# Combined effects of residual cholesterol inflammatory index and triglyceride-glucose-BMI on risk of cardio-cerebrovascular disease: A cohort study

**DOI:** 10.1097/MD.0000000000045395

**Published:** 2025-10-24

**Authors:** Hongfei Yang, Chao Sun, Ya Li, You Zhou, Rui Wang, Yingxue Li

**Affiliations:** a Department of Neurology, Yingshang County People’s Hospital, Fuyang, Anhui, China; b Department of Cardiology, Yingshang County People’s Hospital, Fuyang, Anhui, China.

**Keywords:** body mass index, cardio, cerebrovascular disease, China Health and Retirement Longitudinal Study, glucose, residual cholesterol inflammatory index, triglyceride

## Abstract

This study aimed to analyze the impact of the residual cholesterol inflammatory index (RCII) and the triglyceride–glucose–body mass index (TyG-BMI) on the risk of cardio-cerebrovascular disease (CCVD). Data were obtained from the China Health and Retirement Longitudinal Study, and participants were categorized into 4 groups based on optimal cutoff values of RCII and TyG-BMI. The influence of RCII, TyG-BMI, and their combination on CCVD was assessed using Cox proportional hazards regression and Kaplan–Meier survival models. A total of 7677 individuals were included. Both higher RCII and higher TyG-BMI were independently associated with increased risk of CCVD. Importantly, the combined elevation of RCII and TyG-BMI showed the highest risk (HR = 1.47, 95% CI = 1.21–1.78, *P* < .05), whereas elevation of either marker alone was not statistically significant. Furthermore, no significant multiplicative or additive interaction between RCII and TyG-BMI was observed. These findings suggest that combined assessment of RCII and TyG-BMI may better identify high-risk individuals, although the lack of interaction indicates that the 2 indices contribute independently to CCVD risk.

## 1. Introduction

Cardio-cerebrovascular disease (CCVD) remains a leading cause of mortality and disability worldwide, encompassing conditions such as cardiac arrest, stroke, and coronary heart disease.^[1]^ With 330 million CCVD patients in China in 2023 – including 8.9 million with cardiac arrest, 11.39 million with coronary heart disease, and 13 million with stroke – the burden on public health is enormous.^[2]^

Remnant cholesterol (RC), defined as the cholesterol content of triglyceride-rich lipoproteins after subtracting high-density lipoprotein cholesterol (HDL-C) and low-density lipoprotein cholesterol (LDL-C), has emerged as an important lipid risk factor. Elevated RC levels are associated with both atherosclerotic progression and systemic inflammation.^[3]^ Building on this concept, the residual cholesterol inflammatory index (RCII) – which integrates RC with high-sensitivity C-reactive protein (HS-CRP) – has been developed as a novel biomarker linking lipid metabolism and inflammation. Large population-based studies have shown that individuals with concomitant elevations in RC and HS-CRP exhibit the highest risk of myocardial infarction and all-cause mortality, underscoring the incremental prognostic value of RCII.^[4,5]^

Insulin resistance (IR) is another pivotal determinant of cardiovascular risk. The triglyceride–glucose–body mass index (TyG-BMI), which combines the triglyceride–glucose index with body mass index, serves as a simple and robust surrogate of IR and adiposity-related risk.^[6]^ Recent studies demonstrate that TyG-related indices are strongly associated with cardiovascular morbidity and mortality across diverse populations.^[7,8]^ Moreover, TyG-BMI has shown superior predictive ability compared with the TyG index alone, particularly in relation to stroke severity and adverse outcomes.^[9]^

Despite these advances, few studies have assessed the combined impact of RCII and TyG-BMI on CCVD risk. It remains unclear whether their coexistence confers greater predictive value or whether they interact synergistically. Therefore, the present study aimed to evaluate the independent and combined associations of RCII and TyG-BMI with incident CCVD in a large, nationally representative Chinese cohort (CHARLS). We hypothesized that individuals with concomitantly elevated RCII and TyG-BMI would have the highest CCVD risk, while no statistically significant multiplicative or additive interaction would be observed between the 2 markers.

## 2. Resources and procedures

### 2.1. Population of study and sources of data

Participants were drawn from the China Health and Retirement Longitudinal Study (CHARLS), a nationally representative cohort of Chinese adults aged ≥ 45 years that began in 2011.^[10]^ CHARLS recruited 17,708 participants from 150 counties and 450 villages across 28 provinces, using multistage stratified sampling. Data included demographics, lifestyle, health, insurance, and health care utilization, collected through structured computer-assisted personal interviews at baseline and every 2 to 3 years thereafter. For this study, data from CHARLS 2011, 2013, 2015, 2018, and 2020 waves were used.

Ethical approval was granted by the Institutional Review Board of Peking University (BU; IRB00001052-11015), and all participants provided written informed consent.

**Figure 1.**
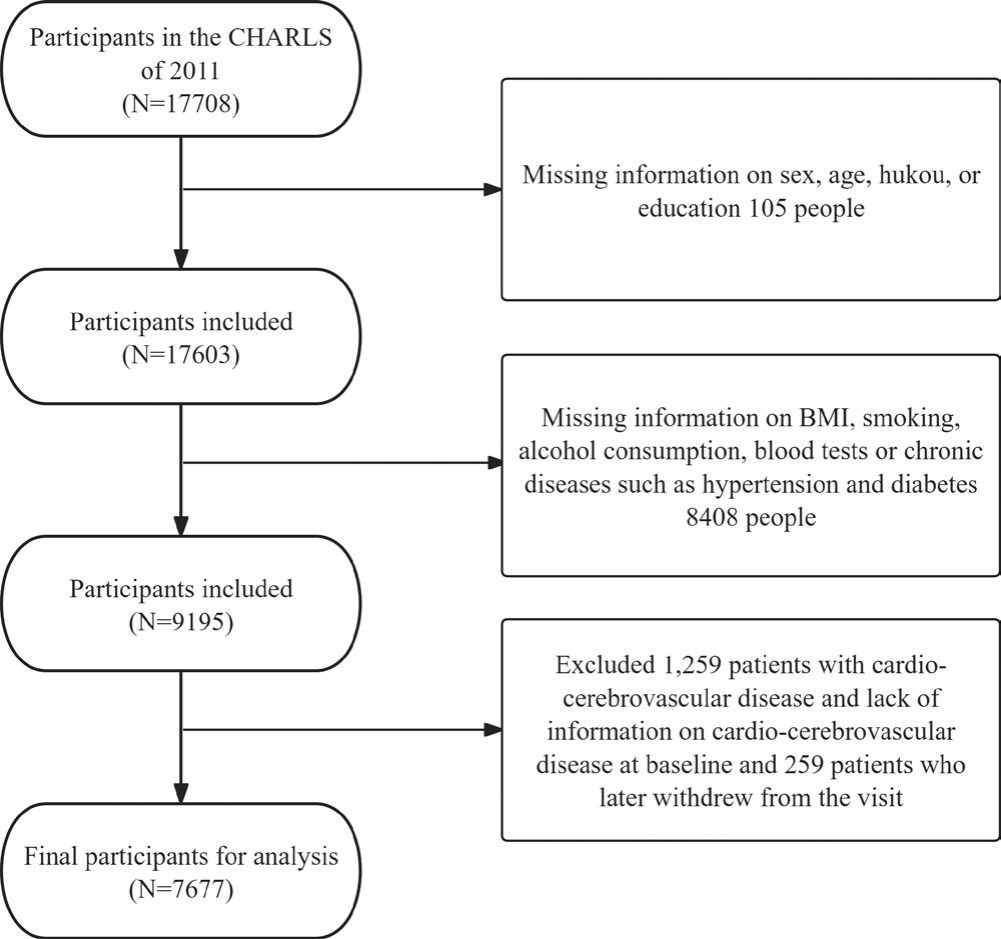
Participant selection flowchart.

Exclusion criteria were: prevalent CCVD at baseline; missing baseline information on CCVD; missing baseline covariates (e.g., BMI, smoking, alcohol use, blood biomarkers); and loss to follow-up (Fig. 1). This ensured a complete-case analysis without imputation.

The flowchart also includes “hukou,” which refers to the Chinese household registration system, distinguishing individuals as agricultural (rural) or nonagricultural (urban) residents, a key socioeconomic determinant in Chinese cohorts.^[11]^

### 2.2. RCII and TyG-BMI assessment

The RCII was calculated as: RCII = (TC (mg/dL)−HDL-C (mg/dL) −LDL-C (mg/dL)) × HS-CRP (mg/L)/10^[12]^; (2) The triglyceride–glucose–body mass index (TyG-BMI) was defined as: TyG-BMI = ln [TG (mg/dL) × FBG (mg/dL)/2] × BMI, where TG is triglycerides, FBG is fasting blood glucose, and BMI is body mass index.^[13]^

### 2.3. Description of CCVD

The primary outcome was incident CCVD, including cardiovascular (e.g., heart disease, angina, myocardial infarction, heart failure) and cerebrovascular events (stroke). CCVD was defined based on self-reported physician diagnosis from follow-up questionnaires: “Has your doctor ever diagnosed you with heart disease, angina, coronary heart disease, heart failure, or other heart problems?” and “Has your doctor ever diagnosed you with a stroke?” (See Supplementary File 1, Supplemental Digital Content, https://links.lww.com/MD/Q438). Incident cases were counted as the first occurrence, and co-occurrence within the same wave was treated as a single event.

Although outcomes relied partly on self-report, trained interviewers and standardized protocols were used to reduce recall bias.

### 2.4. Covariates

This study assessed covariates, including demographics, health condition and performance, and hematological test markers, from 2011 baseline interview data. Covariates included gender (female/male), age, type of household (agricultural, nonagricultural, and other), education level (low level: illiterate, did not finish elementary school, private school, and elementary school graduation; medium level: Junior high school, senior high school, and secondary school graduation; and high level: junior high school graduation, bachelor’s degree, master’s and doctoral degrees, and doctoral degrees). BMI, smoking status, drinking status (alcohol consumption, a small amount of alcohol consumption, no alcohol consumption), hypertension disease, diabetes mellitus, dyslipidemia, cancer, blood indices: FBG, total cholesterol (TC), HDL-C, LDL-C, TG, urea nitrogen (blood urea nitrogen), creatinine (serum creatinine), uric acid, HS-CRP, glycosylated hemoglobin (HbA1c), and platelets (PLTs).

### 2.5. Statistical analysis

Continuous variables were expressed as mean ± standard deviation or median (IQR) depending on distribution (*t* test or Mann–Whitney *U* test). Categorical variables were compared by χ^2^ or Fisher exact test.

To examine dose-response relationships, restricted cubic spline (RCS) regression was applied. Correlations between RCII and TyG-BMI were tested by Spearman correlation.

Participants were divided into high vs. low RCII and TyG-BMI groups based on optimal cutoff values derived from the X-tile program, which determines thresholds that maximize log-rank statistics for survival differences.^[14]^ This method has been widely applied in clinical epidemiology to identify clinically meaningful cut-points.

Subsequently, 4 subgroups were created: Low RCII + Low TyG-BMI; High RCII + Low TyG-BMI; Low RCII + High TyG-BMI; High RCII + High TyG-BMI.

Associations with CCVD were analyzed using multivariable Cox proportional hazards regression models: Model 1: unadjusted; Model 2: adjusted for age, sex, hukou, and education; Model 3: further adjusted for BMI, smoking, drinking, hypertension, diabetes, dyslipidemia, and cancer; Model 4: further adjusted for laboratory biomarkers (FBG, TC, HDL-C, etc).

Multiplicative interactions were assessed by including a product term in the Cox model. Additive interactions were evaluated by 3 indices: synergy index (SI), attributable proportion (AP), and relative excess risk due to interaction (RERI). In this context, S > 1, AP > 0, or RERI > 0 indicates the presence of additive interaction.^[15]^ All analyses were performed in R 4.4.2, SPSS 26.0, and X-tile software, with two-sided *P* < .05 considered statistically significant.

## 3. Outcomes

### 3.1. Fundamental data and baseline characteristics

**Figure 2.**
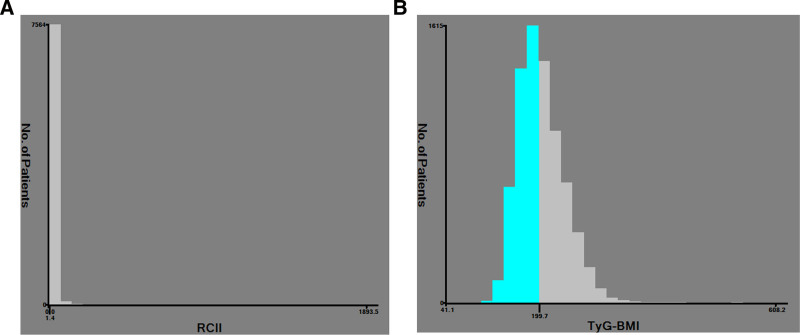
Optimal cutoff values for RCII and TyG-BMI in relation to cardiovascular disease (CCVD) risk. (A) The RCII cutoff value; (B) the TyG-BMI cutoff value. RCII = residual cholesterol inflammatory index, TyG-BMI = triglyceride–glucose–body mass index.

The investigation incorporated 7677 participants, of whom 3568 (46.48%) were men and 4109 (53.52%) were women. The median age was 58.00 (51.00, 65.00). X-tile program revealed that the ideal threshold value for the risk of CCVD was 1.41 (<1.41 was defined as a low-level RCII, n = 3098; ≥1.41 was defined as a high-level RCII, n = 4579), and 199.70 (<199.70 was defined as a low-level TyG-BMI, n = 3971; ≥199.70 was defined as a high-level TyG-BMI, n = 3706). Each was therefore divided into groups of high and low levels. Refer to Figure 2.

The respondents were subsequently categorized into 4 groups according to the combination of the 2 metrics: low RCII and low TyG-BMI groups (n = 2220, 28.92%), high RCII and low TyG-BMI groups (n = 1751, 22.81%), low RCII and high TyG-BMI groups (n = 878, 11.43%), and high RCII and high TyG-BMI groups (n = 2828, 36.44%). Baseline analysis was performed for this subgroup. Notable disparities were observed in the majority of the baseline variables, including age, sex, hukou, BMI, level of education, use of cigarettes, drinking habits, hypertension, diabetes mellitus and dyslipidemia status, in the 4 cohorts (*P* < .05). Furthermore, FBG, TC, HDL-C, TG, blood urea nitrogen, uric acid, HS-CRP, LDL-C, HbA1c, and PLT all demonstrated substantial statistical disparities (*P* < .05). Table 1 displays the comprehensive baseline features of the research cohort.

**Table 1:** Comparison of baseline information between the RCII subgroup and combined TyG-BMI subgroup.

Variables	Total (n = 7677)	Low RCII and low TyG-BMI (n = 2220)	High RCII and low TyG-BMI (n = 1751)	Low RCII and high TyG-BMI (n = 878)	High RCII and high TyG-BMI (n = 2828)	*t/Z*/χ^2^ value	*P* value
Gender, n (%)							
Male	3568 (46.48)	1085 (48.87)	1022 (58.37)	322 (36.67)	1139 (40.28)	182.266	<.05
Female	4109 (53.52)	1135 (51.13)	729 (41.63)	556 (63.33)	1689 (59.72)		
Age (yr) Median (IQR)	58.00 (51.00, 65.00)	58.00 (51.00, 65.00)	60.00 (54.00, 69.00)	55.00 (48.00, 61.00)	57.00 (51.00, 63.00)	250.859	<.05
Hukou, n (%)							
Agricultural hukou	6542 (85.22)	1996 (89.91)	1557 (88.92)	733 (83.49)	2256 (79.77)	126.471	<.05
Nonagricultural hukou and others	1135 (14.78)	224 (10.09)	194 (11.08)	145 (16.51)	572 (20.23)		
Education level, n (%)							
Low level	5398 (70.31)	1598 (71.98)	1339 (76.47)	579 (65.95)	1882 (66.55)	63.463	<.05
Medium level	2191 (28.54)	603 (27.16)	394 (22.50)	287 (32.69)	907 (32.07)		
High level	88 (1.15)	19 (0.86)	18 (1.03)	12 (1.37)	39 (1.38)		
BMI (kg/m^2^) Median (IQR)	23.02 (20.79, 25.59)	21.04 (19.56, 22.39)	20.78 (19.40, 22.01)	25.56 (24.36, 27.12)	25.77 (24.07, 27.86)	4846.009	<.05
Smoking, n (%)							
Yes	2979 (38.80)	898 (40.45)	923 (52.71)	233 (26.54)	925 (32.71)	245.06	<.05
No	4698 (61.20)	1322 (59.55)	828 (47.29)	645 (73.46)	1903 (67.29)		
Drinking, n (%)							
Yes	1991 (25.93)	636 (28.65)	523 (29.87)	202 (23.01)	630 (22.28)	48.295	<.05
A little	601 (7.83)	181 (8.15)	131 (7.48)	69 (7.86)	220 (7.78)		
No	5085 (66.24)	1403 (63.2)	1097 (62.65)	607 (69.13)	1978 (69.94)		
Hypertension, n (%)							
Yes	1592 (20.74)	250 (11.26)	245 (13.99)	186 (21.18)	911 (32.21)	396.456	<.05
No	6085 (79.26)	1970 (88.74)	1506 (86.01)	692 (78.82)	1917 (67.79)		
Diabetes, n (%)							
Yes	353 (4.60)	42 (1.89)	46 (2.63)	48 (5.47)	217 (7.67)	115.045	<.05
No	7324 (95.40)	2178 (98.11)	1705 (97.37)	830 (94.53)	2611 (92.33)		
Dyslipidemia, n (%)							
Yes	546 (7.11)	73 (3.29)	60 (3.43)	73 (8.31)	340 (12.02)	190.279	<.05
No	7131 (92.89)	2147 (96.71)	1691 (96.57)	805 (91.69)	2488 (87.98)		
Cancer, n (%)							
Yes	67 (0.87)	16 (0.72)	14 (0.8)	7 (0.8)	30 (1.06)	1.916	.59
No	7610 (99.13)	2204 (99.28)	1737 (99.2)	871 (99.2)	2798 (98.94)		
FBG (mg/dL) Median (IQR)	102.24 (94.14, 112.86)	98.64 (91.44, 106.38)	99.72 (92.16, 109.26)	102.42 (95.58, 112.14)	107.10 (98.60, 121.86)	628.609	<.05
TC (mg/dL) Median (IQR)	190.59 (167.01, 215.34)	184.02 (161.21, 206.83)	186.34 (163.15, 211.47)	189.43 (165.56, 212.24)	197.94 (173.97, 225.39)	235.081	<.05
HDL-C (mg/dL) Median (IQR)	49.87 (40.98, 60.31)	58.38 (49.87, 68.43)	51.42 (42.91, 61.86)	50.64 (43.69, 59.54)	42.14 (35.57, 50.26)	1709.136	<.05
LDL-C (mg/dL) Median (IQR)	113.66 (92.78, 136.86)	110.95 (92.01, 131.44)	109.79 (88.92, 132.22)	120.04 (99.36, 143.82)	117.14 (93.94, 142.27)	85.252	<.05
TG (mg/dL) Median (IQR)	103.54 (74.34, 151.34)	74.34 (58.41, 93.81)	96.46 (71.68, 130.98)	97.35 (77.00, 121.25)	158.41 (115.93, 222.35)	2898.437	<.05
BUN (mg/dL) Median (IQR)	15.15 (12.52, 18.18)	15.29 (12.60, 18.57)	15.60 (12.86, 18.74)	14.96 (12.46, 18.01)	14.82 (12.32, 17.59)	41.278	<.05
SCR (mg/dL) Median (IQR)	0.76 (0.64, 0.88)	0.75 (0.64, 0.86)	0.78 (0.67, 0.90)	0.71 (0.63, 0.84)	0.76 (0.66, 0.89)	77.808	<.05
UA (mg/dL) Median (IQR)	4.27 (3.56, 5.12)	3.98 (3.35, 4.77)	4.35 (3.61, 5.22)	3.97 (3.32, 4.72)	4.58 (3.79, 5.44)	364.02	<.05
HS-CRP (mg/L) Median (IQR)	0.98 (0.53, 2.08)	0.49 (0.35, 0.74)	1.84 (1.05, 3.82)	0.52 (0.37, 0.74)	1.58 (0.94, 2.88)	3506.706	<.05
HbA1c (%) Median (IQR)	5.10 (4.90, 5.40)	5.10 (4.80, 5.30)	5.10 (4.80, 5.40)	5.10 (4.80, 5.40)	5.20 (4.90, 5.60)	228.584	<.05
PLT (10^9^/L) Median (IQR)	207.00 (162.00, 255.00)	202.00 (161.00, 249.25)	206.00 (158.00, 255.00)	204.00 (161.00, 255.00)	211.00 (164.00, 260.00)	14.474	<.05

BUN = blood urea nitrogen, FBG = fasting blood glucose, HbA1c = glycated hemoglobin, HDL-C = high-density lipoprotein cholesterol, HS-CRP = high-sensitivity C-reactive protein, LDL-C = low-density lipoprotein cholesterol, PLT = platelet, RCII = residual cholesterol inflammatory index, TC = total cholesterol, TG = triglycerides, TyG-BMI = triglyceride–glucose–body mass index, UA = uric acid.

### 3.2. Relationships among the RCII, TyG-BMI, and CCVD

**Figure 3.**
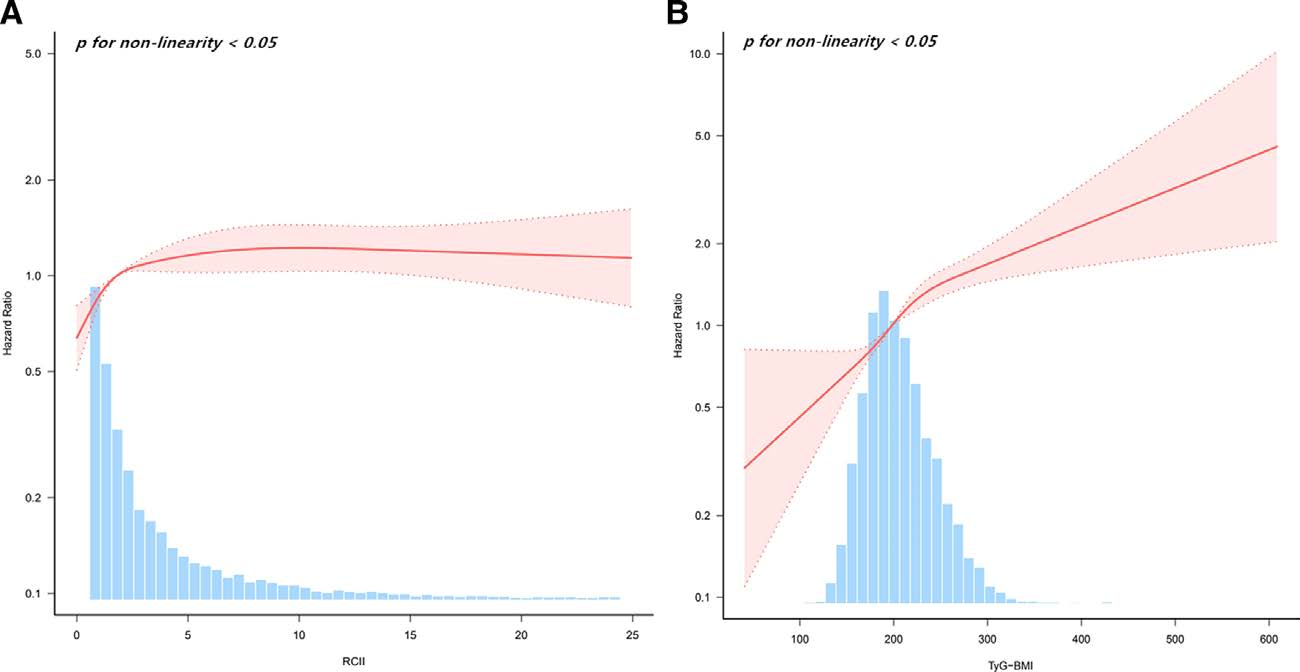
Restricted cubic spline (RCS) plots of RCII and TyG-BMI versus CCVD risk. (A) RCII; (B) TyG-BMI. CCVD = cardio-cerebrovascular disease, RCII = residual cholesterol inflammatory index, TyG-BMI = triglyceride–glucose–body mass index.

**Figure 4.**
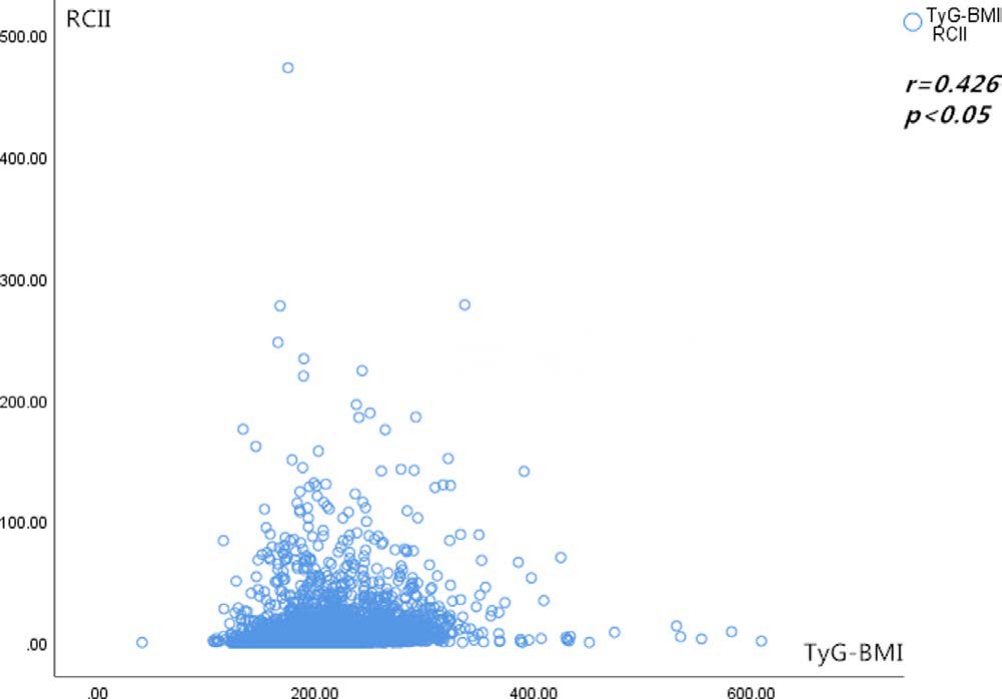
Scatterplot of the Spearman correlation between RCII and TyG-BMI. RCII = residual cholesterol inflammatory index, TyG-BMI = triglyceride–glucose–body mass index.

An aggregate of 1336 individuals had CCVD over the maximum 9-year follow-up duration (960 instances of cardiovascular disease and 437 incidences of cerebrovascular disease, which cooccurred in 61 cases during the same follow-up period, totaling 1336 cases of CCVD). The findings from our RCS investigation indicated a nonlinear relationship (*P* < .05) of the danger of CCVD and both the RCII and TyG-BMI (Fig. 3). The RCII and TyG-BMI were positively associated (*R* = 0.426, *P* < .05) in light of the Spearman correlation study (Fig. 4).

**Figure 5.**
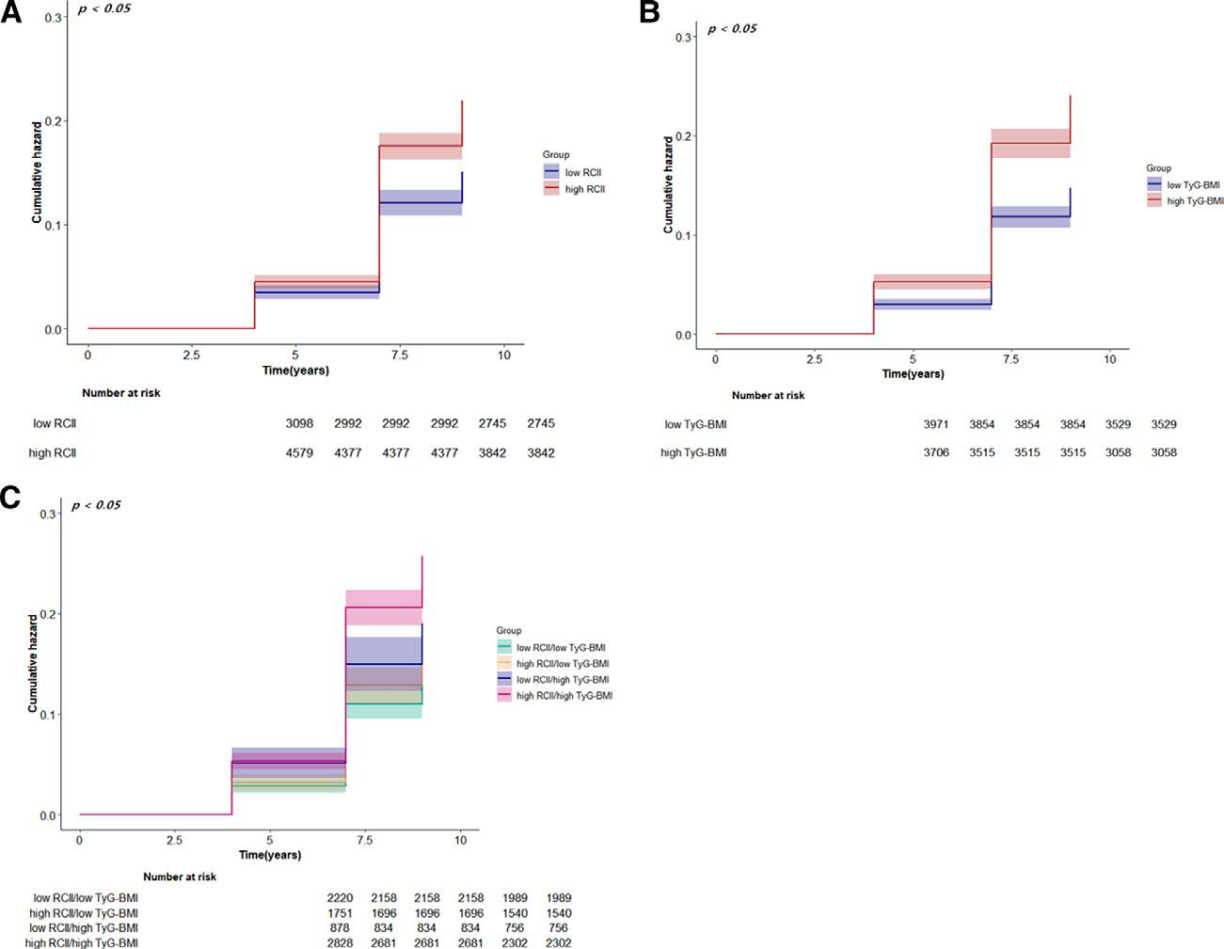
Kaplan–Meier cumulative incidence curves for RCII, TyG-BMI, and CCVD. (A) Groups defined by RCII alone; (B) groups defined by TyG-BMI alone; (C) groups defined jointly by RCII and TyG-BMI. CCVD = cardio-cerebrovascular disease, RCII = residual cholesterol inflammatory index, TyG-BMI = triglyceride–glucose–body mass index.

When grouped by RCII alone, the prevalence of cardiovascular disease and CCVD was 14.01% (434/3098) in the low-level group and 19.70% (902/4579) in the high-level group. When grouped by TyG-BMI alone, the prevalence of CCVD was 13.67% (543/3971) in the low-level group and 21.40% (793/3706) in the high-level group. When the RCII and TyG-BMI were grouped together, the CCVD incidence rates in the 4 groups were as follows: low RCII and low TyG-BMI (12.70%, 282/22 20), high RCII and low TyG-BMI (14.91%, 261/1 751), low RCII and high TyG-BMI (17.31%, 152/878), and high RCII and high TyG-BMI (22.67%, 641/2828). The overall prevalence of CCVD exhibited an upward trend with the escalation of both RCII and TyG-BMI, according to Kaplan–Meier survival curves. With a substantial disparity (*P* < .05), the cumulative incidence of CCVD was greatest in the high RCII and high TyG-BMI cohorts and lowest in the low RCII and low TyG-BMI groups in the combined RCII and TyG-BMI subgroups (Fig. 5).

Cox proportional risk regression model analysis: Following control for all possible confounding variables (Model 4), individuals exhibiting elevated RCII levels demonstrated a 26% heightened risk of CCVD in comparison to their counterparts with lower RCII levels (95% CI: 1.10–1.44, *P* < .05). The risk of CCVD was also 24% greater for those with a high TyG-BMI than for those with a low TyG-BMI (95% CI: 1.06–1.45, *P* < .05). The risk was greater in the high RCII and low TyG-BMI groups, the low RCII and high TyG-BMI groups, and the high RCII and high TyG-BMI groups when the RCII and TyG-BMI were combined, with the low RCII and low TyG-BMI cohorts serving as a reference. However, this difference was statistically significant only in the high RCII and high TyG-BMI cohorts (HR = 1.47, 95% CI = 1.21–1.78; *P* < .05). Refer to Table 2.

**Table 2:** RCII and TyG-BMI and Cox proportional risk regression analysis of CCVD.

Variables	Model 1		Model 2		Model 3		Model 4	
	HR (95% CI)	*P*	HR (95% CI)	*P*	HR (95% CI)	*P*	HR (95% CI)	*P*
RCII								
Low RCII	1.00 (Reference)	<.05	1.00 (Reference)	<.05	1.00 (Reference)	<.05	1.00 (Reference)	<.05
High RCII	1.45 (1.29–1.63)		1.41 (1.26–1.58)		1.20 (1.07–1.36)		1.26 (1.10–1.44)	
TyG-BMI								
Low TyG-BMI	1.00 (Reference)	<.05	1.00 (Reference)	<.05	1.00 (Reference)	<.05	1.00 (Reference)	<.05
High TyG-BMI	1.64 (1.47–1.83)		1.66 (1.49–1.86)		1.26 (1.09–1.45)		1.24 (1.06–1.45)	
RCII and TyG-BMI								
Low RCII and low TyG-BMI	1.00 (Reference)		1.00 (Reference)		1.00 (Reference)		1.00 (Reference)	
High RCII and low TyG-BMI	1.19 (1.00–1.40)	<.05	1.14 (0.96–1.35)	.122	1.13 (0.95–1.33)	.172	1.20 (1.00–1.43)	.05
Low RCII and high TyG-BMI	1.40 (1.15–1.71)	<.05	1.44 (1.18–1.76)	<.05	1.16 (0.94–1.43)	.179	1.15 (0.92–1.43)	.209
High RCII and high TyG-BMI	1.89 (1.64–2.17)	<.05	1.86 (1.62–2.15)	<.05	1.38 (1.17–1.63)	<.05	1.47 (1.21–1.78)	<.05

Model 1: Uncorrected model; Model 2: Corrected for gender, age, hukou and education level; Model 3: Corrected for BMI, smoking, alcohol consumption, hypertension, diabetes mellitus, dyslipidemia, and cancer according to Model 2; Model 4: Corrected for blood test indicators such as FBG, TC, and HDL-C according to Model 3.

CCVD = cardio-cerebrovascular disease, FBG = fasting blood glucose, HDL-C = high-density lipoprotein cholesterol, RCII = residual cholesterol inflammatory index, TC = total cholesterol, TyG-BMI = triglyceride–glucose–body mass index.

### 3.3. Interaction effect of the RCII and TyG-BMI on CCVD

For CCVD (Model 4), the multiplicative interaction between RCII and TyG-BMI failed to reach importance in statistics (OR = 1.07, 95% CI: 0.83–1.37, *P* > .05), nor was the additive interaction (RERI = −0.12, 95% CI: −0.17–0.41; AP = 0.08, 95% CI: −0.11–0.28; SI = 0.35, 95% CI: 0.58–3.14; *P* > .05). The RCII and TyG-BMI did not interact (Table 3).

**Table 3:** The interplay impact of RCII and TyG-BMI in relation to CCVD.

Interactive indices	Model 1		Model 2		Model 3		Model 4	
	Interactive effects (95% CI)	*P* value	Interactive effects (95% CI)	*P* value	Interactive effects (95% CI)	*P* value	Interactive effects (95% CI)	*P* value
Multiplicative effect	1.13 (0.89–1.45)	.31	1.13 (0.89–1.44)	.32	1.06 (0.83–1.35)	.65	1.07 (0.83–1.37)	.61
Additive effect								
RERI	0.30 (−0.01–0.61)	<.05	0.28 (−0.04–0.59)	<.05	0.10 (−0.18–0.37)	.25	0.12 (−0.17–0.41)	.21
AP	0.16 (−0.01–0.33)	<.05	0.15 (−0.02–0.32)	<.05	0.07 (−0.13–0.27)	.25	0.08 (−0.11–0.28)	.21
SI	1.51 (0.87–2.16)	<.05	1.48 (0.85–2.58)	<.05	1.34 (0.49–3.64)	<.05	1.35 (0.58–3.14)	<.05

Model 1: Uncorrected model; Model 2: Corrected for gender, age, hukou and education level; Model 3: Corrected for BMI, cigarette use, drinking, hypertension, diabetes mellitus, dyslipidemia, and cancer according to Model 2; Model 4: Corrected for blood test indicators such as FBG, TC, and HDL-C according to Model 3.

AP = attributable proportion, BMI = body mass index, CCVD = cardio-cerebrovascular disease, FBG = fasting blood glucose, HDL-C = high-density lipoprotein cholesterol, RCII = residual cholesterol inflammatory index, RERI = risk due to interaction, SI = synergy index, TC = total cholesterol, TyG-BMI = triglyceride–glucose–body mass index.

Multiplicative interaction. No evidence of multiplicative interaction was observed between RCII and TyG-BMI across all models (all *P* > .05).

Additive interaction. In the unadjusted model (Model 1) and the model adjusted for age, sex, hukou, and education (Model 2), indices for additive interaction reached statistical significance: the relative excess RERI and attributable proportion (AP) were both greater than zero, and the SI exceeded 1 (all *P* < .05). However, after further adjustment for clinical risk factors (Model 3) and laboratory biomarkers (Model 4), these associations were attenuated and no longer statistically significant (all *P* > .05).

## 4. Discussion

Our study demonstrates that both RCII and TyG-BMI are individually associated with CCVD risk, and that participants with concomitantly elevated RCII and TyG-BMI exhibit the highest incidence of CCVD. However, importantly, no robust multiplicative or additive interaction was observed after full adjustment, suggesting that the 2 indices contribute independently rather than synergistically to CCVD risk. These findings are consistent with prior studies linking RCII to cardiovascular mortality.^[3,4]^ Every SD rise in lnRCII got linked to a 21% increased likelihood of cardiovascular death (HR = 1.21, 95% CI: 1.08–1.35), according to research from the NHANES population.^[16]^ According to another Chinese study,^[12]^ the incidence of first cerebral infarction was 10.6% greater when the RCII standard deviation was elevated (HR = 1.106, 95% CI: 1.048–1.167). After controlling for all factors, high levels of RCII CCVD rose by 26% in the current study when the RCII was examined individually (HR = 1.26, 95% CI: 1.10–1.44, *P* < .05). This is consistent with earlier findings.

Atherosclerosis, the pathological foundation of CCVD, is characterized by lipid buildup, fibrosis, and calcification of the arterial wall, resulting in hemodynamic alterations and vessel lumen narrowing.^[17]^ LDL-C is thought to be the most significant lipid marker for the risk of CCVD in this process.^[18,19]^ However, in recent years, it has been discovered that leftover cholesterol might enter the artery wall, harm the intima, and cause atherosclerosis, which in turn can lead to CCVD.^[20]^ Furthermore, RCS and inflammation can interact. RC has the potential to initiate an inflammatory reaction that adversely affects the endothelium,^[21]^ which increases the incidence of CCVD by accelerating the process of atherosclerosis through engagement with lipid metabolism during plaque formation and causing thrombosis.^[22,23]^ From a pathophysiological perspective, RCII captures the dual influence of lipid metabolism and systemic inflammation, while TyG-BMI reflects IR combined with adiposity. Their coexistence may identify individuals at particularly high risk because both lipid-inflammation and insulin-resistance/obesity pathways are activated. Yet, the lack of statistical interaction in adjusted models indicates that each factor exerts its effect independently, and the observed joint effect likely reflects risk accumulation rather than biological synergy.

When diagnosing IR, TyG demonstrates a strong connection with the gold standard insulin high-normoglycemic clamp technique (HEC).^[24]^ An investigation conducted by Rafiee H et al^[25]^ uncovered an important link between an increased TyG index and an elevated danger of stroke (HR = 1.45; 95% CI: 0.96–2.19; *P* = .042, threshold: 8.92) and cardiovascular disease (HR = 1.48; 95% CI 1.22–1.79; *P* < .001, threshold: 8.91). Conversely, the effectiveness of the TyG-BMI has significantly increased since the introduction of BMI, particularly in regard to evaluating the health risks associated with obesity.^[26]^ The TyG-BMI has been shown to have a substantial correlation with the likelihood of heart disease, NAFLD, and additional metabolic conditions.^[27]^ Upon independent examination of the TyG-BMI in the current investigation, a similar conclusion was drawn: the high-level group exhibited a 24% elevated danger of TyG-BMI (HR = 1.24, 95% CI = 1.06–1.45, *P* < .05).

Since elevated levels of indicators associated with TyG are linked to IR – fasting glucose primarily reflecting IR in the liver and TG primarily reflecting IR in adipocytes – this could be the mechanism by which TyG-related markers increase the risk of CCVD.^[28,29]^ IR causes arterial endothelial damage, nitric oxide inactivation, disruptions in glucose metabolism and lipotoxicity, and PLT aggregation,^[30–33]^ all of which contribute to CCVD. The TyG-BMI could potentially demonstrate greater efficacy compared to the TyG indices at predicting the risk of CCVD because it additionally accounts for data on body fat distribution and obesity, both of which are strongly linked to the buildup of visceral fat. The risk of CCVD is further increased by atherosclerosis, which is caused by endothelial dysfunction, proinflammatory cytokine production, and other processes.^[34]^

No further investigation has been undertaken regarding the combined influence of the RCII and TyG-BMI on CCVD. With the low RCII and low TyG-BMI groups as a reference, the current study revealed that when the RCII and TyG-BMI were combined, the risk was greater in the high RCII and low TyG-BMI groups, the low RCII and high TyG-BMI groups, the high RCII and high TyG-BMI groups, and the high RCII and high TyG-BMI groups. However, only the high RCII and high TyG-BMI groups were significantly different (HR = 1.47, 95% CI = 1.21–1.78, *P* < .05). According to this conclusion, if only one is elevated in the population, the increased risk of CCVD is not substantial; however, if both are elevated, the risk will be much greater. Nevertheless, the 2 impacts on CCVD did not interact. Combined assessment of RCII and TyG-BMI may help stratify high-risk individuals in clinical or public health settings, but these indices should be considered adjuncts to established risk scores rather than replacements. Preventive strategies could focus on individuals with co-elevations, in whom interventions targeting both lipid metabolism and IR may be especially beneficial. However, further studies are needed to confirm whether such combined interventions reduce CCVD incidence.

IR and RC are promoted in both directions; RC causes IR to develop, whereas IR influences RC metabolism.^[35,36]^ According to some studies, IR is linked to both postprandial and fasting RC.^[37,38]^ First, high levels of RC cause the body to produce inflammatory factors that impact insulin signaling and result in IR.^[39]^ Second, RC cholesterol toxicity causes insulin receptor dysfunction in target organs.^[40,41]^ Furthermore, serum HS-CRP levels and IR are positively correlated.^[42]^ Conversely, IR leads to the liver producing more apolipoprotein B (apoB) and triglyceride-rich very low-density lipoprotein particles, and it also causes the liver to have more time to move LRP1 from endosomes to the hepatocyte plasma membrane, which increases RC levels.^[43]^ As a result, RC and IR may reinforce one another in a vicious cycle. In line with Chen C et al’s investigation,^[44]^ the current investigation demonstrated an affirmative relationship between the RCII and TyG-BMI (Spearman *R* = 0.20, *P* < .05).

The increase in CCVD is not substantial, however, if both indicators are evaluated at the same time and only one is elevated while the other stays normal. This could be because IR or problems in lipid metabolism have not progressed to a serious degree. When both signs are high, it indicates that associated metabolic abnormalities are already very noticeable and that there is a notable rise in the likelihood of CCVD.

In light of the aforementioned findings, we put forth the subsequent hypotheses: If only one RCII or TyG-BMI is elevated in the population, timely preventive interventions may interrupt the vicious cycle between the 2 factors and reduce the incidence of CCVD; Timely identification of high-risk groups with both elevated and combined interventions for IR and improvements in lipid metabolism may significantly reduce the risk of CCVD. However, further investigation is necessary to substantiate these outcomes.

## 5. Limitations of the research

This study has several limitations. The study population was restricted to Chinese adults aged ≥ 45 years from the CHARLS cohort; therefore, findings may not be generalizable to younger populations or non-Chinese cohorts. Outcome data relied partly on self-reported physician diagnoses, raising the possibility of recall bias despite standardized interviews. Although we adjusted for multiple demographic, lifestyle, and clinical factors, residual confounding cannot be excluded. We evaluated RCII and TyG-BMI at baseline; changes over time were not analyzed, limiting insight into longitudinal dynamics. Participants with comorbidities such as hypertension and diabetes were not excluded, which may influence associations. Future research should include external validation in diverse populations, prospective assessment of biomarker trajectories, and interventional studies testing whether targeting both lipid-inflammation and IR pathways can reduce CCVD risk.

## 6. Conclusion

Both RCII and TyG-BMI were independently associated with an increased risk of CCVD. Importantly, participants with concomitantly elevated RCII and TyG-BMI had the highest risk, whereas elevation of either marker alone did not confer a statistically significant increase in risk after full adjustment. These findings indicate that the combined assessment of RCII and TyG-BMI may improve the stratification of high-risk individuals. However, our results do not suggest a synergistic interaction between the 2 markers, and their clinical application should be considered as an adjunct to, rather than a replacement for, established risk prediction models.

## Acknowledgments

The authors would like to thank all participants and staff of the CHARLS project for their contributions. Permission has been obtained from all individuals named in the acknowledgments section.

## Author contributions

**Conceptualization:** Hongfei Yang.

**Data curation:** Chao Sun, Ya Li, You Zhou, Rui Wang.

**Formal analysis:** Hongfei Yang, Yingxue Li.

**Methodology:** Hongfei Yang.

**Writing – original draft:** Hongfei Yang.

**Writing – review & editing:** Yingxue Li.

## Supplementary Material


