# Supplementary material for: Combined effects of residual cholesterol inflammatory index and triglyceride-glucose-BMI on risk of cardio-cerebrovascular disease: A cohort study
Source: Medicine (Baltimore). 2025 Oct 24;104(43):e45395. doi: 10.1097/MD.0000000000045395 (PMC12558257; doi:10.1097/MD.0000000000045395)
Supplement: Supplementary file 1 [file medi-104-e45395-s001.doc]

CHINA HEALTH AND RETIREMENT

LONGITUDINAL STUDY – BASELINE

QUESTIONNAIRE

中国健康与养老追踪调查

全国问卷

August 2011

中国经济研究中心

CHINA CENTER FOR ECONOMIC RESEARCH

PEKING UNIVERSITY

**CONTENTS** 目 录

**COVERSCREEN 过滤问卷** [2](#__RefHeading___Toc297046119)

**A HOUSEHOLD ROSTER 家户登记表** [8](#__RefHeading___Toc297046120)

**B 基本信息** [15](#__RefHeading___Toc297046121)

**C FAMILY 家庭** [26](#__RefHeading___Toc297046122)

C1 PARENT CHILDREARING AND SIBLING INFORMATION 父母、子女、兄弟姐妹信息 [26](#__RefHeading___Toc297046123)

CA PARENT INFORMATION 父母信息 [26](#__RefHeading___Toc297046124)

CB CHILDREARING INFORMATION 子女信息 [30](#__RefHeading___Toc297046125)

CC SIBLINGS 兄弟姐妹 [42](#__RefHeading___Toc297046126)

C2 TIME TRANSFER AND TRANSFERS 家庭交往与经济帮助 [44](#__RefHeading___Toc297046127)

CD TIME TRANSFER 与父母、子女间的交往 [44](#__RefHeading___Toc297046128)

CE TRANSFERS 家庭得到及提供的经济帮助 [46](#__RefHeading___Toc297046129)

CF TIME SPENT PROVIDING CARE 提供照料时间 [56](#__RefHeading___Toc297046130)

**D HEALTH STATUS AND FUNCTIONING 健康状况和功能** [59](#__RefHeading___Toc297046131)

DA HEALTH STATUS 健康状况 [59](#__RefHeading___Toc297046132)

DB FUNCTIONAL LIMITATIONS AND HELPERS 身体功能障碍以及辅助者 [76](#__RefHeading___Toc297046133)

DC COGNITION & DEPPRESSION 认知和抑郁 [83](#__RefHeading___Toc297046134)

DE SELF-REPORTED HEALTH &VIGNETTES 自我评价健康和情景选择题 [87](#__RefHeading___Toc297046135)

**E HEALTH CARE AND INSURANCE 医疗保健与保险** [94](#__RefHeading___Toc297046136)

PART I MEDICAL INSURANCE 第一部分：医疗保险 [94](#__RefHeading___Toc297046137)

PART II HEALTH CARE COSTS AND UTILIZATION 第二部分：医疗成本与使用情况 [98](#__RefHeading___Toc297046138)

**F WORK, RETIREMENT AND PENSION 工作、退休和养老金** [112](#__RefHeading___Toc297046139)

FA JOB STATUS 就业状况 [112](#__RefHeading___Toc297046140)

FB WORK HISTORY 工作史 [113](#__RefHeading___Toc297046141)

FC CURRENT PRIMARY JOB/OCCUPATION 当前、主要工作 [115](#__RefHeading___Toc297046142)

FK UNEMPLOYMENT AND JOB SEARCH ACTIVITIES 失业，求职经历 [131](#__RefHeading___Toc297046143)

FL MOST RECENT JOB 最近一份工作 [132](#__RefHeading___Toc297046144)

FM RETIREMENT 退休与退职 [136](#__RefHeading___Toc297046145)

FN PENSION INSURANCE 养老保险 [143](#__RefHeading___Toc297046146)

**G &H INCOME, EXPENDITRUES AND ASSETS 收入、支出与资产** [156](#__RefHeading___Toc297046147)

G1 VIGNETTS ON INCOME 情景选择题 [156](#__RefHeading___Toc297046148)

G2 HOUSEHOLD INCOME AND EXPENDITURES 家户收入与支出 [156](#__RefHeading___Toc297046149)

HA HOUSEHOLD ASSETS 家户资产 [165](#__RefHeading___Toc297046150)

HB INDIVIDUAL ASSETS 个人资产 [176](#__RefHeading___Toc297046151)

I. HOUSING CHARACTERISTICS 住房情况 [184](#__RefHeading___Toc297046152)

HOUSEHOLD CONTACTS 家户联系信息表 [187](#__RefHeading___Toc297046153)

J. INTERVIEWER OBSERVATION 访问员观察 [189](#__RefHeading___Toc297046154)

**APPENDIX 附表** [191](#__RefHeading___Toc297046155)

# **COVERSCREEN 过滤问卷**

[IWER: Find a respondent (he/she does not have to be 45+ years of age, but could not be nanny, short workers or visitors) from the sample house.]

[访员注意：在这个房子中找一人（这人可以不到45岁，但不能是保姆、做工的人或者客人等）回答下列问题.]

**CV000.** Did the household receive “*a letter to the resident”?* 该户是否收到 “致居民的一封信” ？

（1）Yes 是

（2）No 否

**CV001.** How many households live in this dwelling? 你们这座房子里住了几户人？一户人是指一起吃饭并且共同承担其它生活费用的人。_____0..25Households 户

**[PROCEDURE: Ask each household the following questions.]**

**[程序:对每户人家问以下问题：]**

**CV002_1.** Is this the main home for this household? Is there another house located elsewhere in which the family lives most of the time? 这一户大多数时间居住在这个房子里，还是居住在其他地方的房子里？

（1）There’s another house they live most of the time. 大多数时间居住在其他地方

（2）It’s the main home. 大多数时间住在这个房子里 skip to CV002 跳至CV002

**CV002_2.** Is that house located in this neighbourhood? 主要居住的房子是在本村/社区吗？

（1）Yes 是

（2）No 否 **go to next household 询问下一家户**

**[PROCEDURE：If each household answered no in CV002_2，QUIT.]**

**[程序：如果每户对CV002_2题都回答否，退出.]**

**We appreciate your time. However, there is no eligible household for us to interview this time.**

**谢谢您的配合，但您这里没有符合我们调查条件的家户。耽误您时间了，抱歉！**

**[PROCEDURE：If more than one household lives here，ask each one CV002 and CV003.]**

**[程序：如果多户住在这里，针对每户询问CV002 和CV003 .]**

**CV002.** How many persons live in this household? 这一户住了几个人，要包括在外上学或者打工住在集体宿舍的人。 ___________ 0..25Persons 人

**CV003.** How many persons aged 39 + years that belong to your household? (Those who was born before July 1, 1971 based on the solar calendar or May 9, 1971 based on the lunar calendar,)其中，有几个年龄在39岁及以上的人？（阳历（公历）1971年7月1日或者阴历（农历）1971年5月9日之前出生的人) ________0..25Persons 人

[PROCEDURE: List all households with age-eligible members then randomly choose one, continue CV001_1-CV030. If there is no eligible household, end the cover screen. ]

**[程序：列出所有有适龄对象的家户信息，随机抽取一户作为被调查户，继续询问CV001_1- CV030；如果没有适龄受访者，结束调查。]**

**[PROCEDURE：If only one household lives here，ask CV001_1 to CV030 ]**

**[程序：如果只有一户住在这里，询问CV001_1 到 CV030.]**

**[IWER: Find a respondent (Call it RB) (he/she does not have to be 45+ years old, but could not be nanny, short workers and visitors etc) from the chosen eligible household, and ask CV001_1 to CV030.]**

**[访员注意：在抽取的适龄家户中找一人（这人可以不到45岁，但不能是保姆、做工的人或者客人等）回答CV001_1 到CV030]**

We appreciate your participation. The China Health and Retirement Longitudinal Study (CHARLS) is interested in learning about important aspects of people’s lives, such as their health, financial, and family situations, and in providing a high-quality public use dataset on aging for academic and policy research. The survey sample is composed of persons aged 45 and older from about 10,000 households throughout the country.

非常感谢您参与这次调查。中国健康与养老追踪调查旨在收集老百姓的健康、经济和家庭情况等方面的数据，为学术研究以及政府制定、调整社会保障政策提供科学依据。本次调查是在全国随机抽取10,000个家庭并对家庭中45岁以上的人进行调查。非常荣幸，您是我们随机抽取的住户，希望您能支持和配合我们的工作，为国家社会保障政策改善做贡献。非常感谢！

**CV001_1.** How many dwelling units does your household live in this village/community? 你们家在这个村/社区内住了几个这样的房子?____________ 0..25Houses 个

[IWER: Not ask the house property, but the number of houses in which they are living.不是问这户人家有几套房子的产权，而是他们家居住的房子数量，房子是指抽中的住宅单元。]

**CV001_2.** How many people lived with this household for more than six months in the past year(including those attending school/work away from home but returning to this home almost every week, but not including non-family members who live here for school or work reasons but generally return to one’s own home every week.)? （Not yourself but including all other people living with; nannies should be included. Do not include persons who recently left because they got married to a member outside the household and moved to another household, or persons who recently divorced a member and then moved out.） 过去一年，在这个家住了至少6个月，并且现在还住在这里的有几个人？如果平时上班、上学住在外面，但是周末一般回家的人也算在内。如果平时上班、上学住在这里，但周末回自己家的人不算。如果他过去一年在这家住了至少6个月，但由于结婚、离婚而现在不住这里了，不算此类。_________ 0..25Persons 人

[IWER: This type excludes one who lived with this household for more than six months but has gone out of the country, and he/she lived in the resident living district. 访员注意：如果一个人在这个家住了6个月以上，但是已外出打工，且在外是住在居民区，不是住集体宿舍，不算在此类。]

[PROCEDURE: If CV001_2 >0, ask CV001_2_1]

[程序：如果CV001_2 >0, 请提问CV001_2_1]

**CV001_2_1.** What are their names? 他叫什么名字？___________

**CV001_3.** In the past year, how many people lived in this household for less than 6 months, but recently joined this household because they were new born or were adopted, or got married to another household member, or they got divorced from someone outside the household and moved back to this household? （Not Including yourself）过去一年，有多少人来到这个家还不到6个月，比如说新生的孩子，收养的孩子，或刚结婚过来的，或者离婚回来的? __________0..25Persons 人

**[PROCEDURE: If CV001_3 >0, ask CV001_3_1]**

**[程序：如果CV001_3 >0, 请提问CV001_3_1]**

**CV001_3_1.** What are their names? 这些人叫什么名字？____________

**CV001_5.** In the past year, how many people lived in the household for less than 6 months because they lived with more than one household during the year, but still spent more time with this household than any other household, for example old persons or someone needs to be taken after by others? （NOT including the persons above）除了以上这些人，您家里还有没有老人或者其他需要照顾的人，在多个家庭轮流居住，但是过去一年在您家居住时间最长？如果有的话，有几个人？_______0..25Persons 人

**[PROCEDURE: If CV001_5 >0, ask CV001_5_1]**

**[程序：如果CV001_5 >0, 请提问CV001_5_1 ]**

**CV001_5_1.** What are their names? 这些人叫什么名字？**__________**

**CV001_4.** In the past year, how many people who went to school/joined army/went to work away from home lived elsewhere in a dormitory or workplace (or live-in nanny lives in other’s house) but not living in the resident living district? （NOT including the persons above）除了上面这些人，您家是否还有人外出上学、参军、务工等，在外住在集体宿舍或单位住房（或属于住家保姆，住在别人家），而不是住在居民区？ _______ 0..25Persons 人

**[PROCEDURE: If CV001_4 >0, ask CV001_4_1 ]**

**[程序：如果CV001_4 >0, 请提问CV001_4_1]**

**CV001_4_1.** What are their names? 这些人叫什么名字？**___________**

**CV001_4_2.** Other than the people you have already described, how many persons lived in your household for more than 6 months in the past year, but have since left this household and are no longer members of the household (changzhu renkou)? 除了上面这些人，过去一年，在这个家住了至少6个月，但是已经离开这个家不再是这个家的常住人口，有多少人？______0..25Persons 人

**[PROCEDURE: If CV001_4_2>0, ask CV001_4_3]**

**[程序：如果CV001_4_2 >0, 请提问CV001_4_3 ]**

**CV001_4_3.** What are their names? 这些人叫什么名字？___**_______**

[PROCUDURE: get the total number of all the Household members according to charls mentioned by RB (CVTotal), and list them.]

[程序: 得到RB提到家户成员的数目 (CVTotal)，并且列出他们.]

**CV020.** You have described [**CVAll**] persons who are part of your household. They are as follows [LIST OF ALL PERSONS’ name IN HH]. 您刚才说您家现在有[**CVAll**]个人，他们是[列出所有的人的名单]

Are there any other household members in your household? 请问过去一年您家还有其他家户成员吗？

[IWER: probe and judge by yourself according to the definition of CHARLS household member. 访员注意：根据CHARLS对家户成员的定义进行追问和判断。）

[IWER: Confirm that all household members have been listed. If Not all members are listed, please

(1) Yes to add new one.

[访员注意：确定已经列所有家户成员。如果没有列出所有家户成员，请点击 “(1) 有”增加新成员。]

(1) Yes 有

(2) No 没有

**[PROCUDURE: IF CV020 = 1 asked the following question for additional person]**

**[程序: 如果CV020 = 1询问新增加的人的信息]**

**CV001_6_1.** What is his/her name? 他/她叫什么名字？**___________**

**CV010.** What is [CV001_6_1]’s type? [CV001_6_1 ]是什么类型？

(1) Lived with this household for more than six months in the past year. 过去一年，在这个家住了至少6个月，并且现在还住在这里的人。

(2) New household members. 过去一年，新来到这个家还不到6个月。

(3) Rotating parents or other household members.除了以上这些人，您家里的老人或者其他需要照顾的人，他在多个家庭轮流居住，但是过去一年在您家居住时间最长。

(4) Lived elsewhere in a dormitory or workplace but not in residential area. 除了上面这些人，在外住在集体宿舍或单位住房，而不是住在居民区的人

(5) Lived in the household for more than 6 months in the past year but have left the household and are no longer members of the household (changzhu renkou). 过去一年，在这个家住了至少6个月，但是已经离开这个家不再是这个家的常住人口。

**CV025_0.** Does the household have any live-in nanny? 您家有住家保姆吗？

(1)Yes, on the list 是，在名单上

(2)Yes, not on the list 是， 不在名单上 skip to CV030

(3)No 否 skip to CV030

**If CV025_0 =1, ask:**

**CV025.** Please check (Mult choice).如果有，在您说的这些人中，有谁是您家的保姆吗？请选上。（多选）

1-25: the list of household member 给出前面的家户成员的列表

**CV030.** In the following list, who was born before July 1, 1971 based on the solar calendar or May 9, 1971based on the lunar calendar?( Those who’re aged 39 and older) 请问在下列人中，谁是在阳历（公历）1971年7月1日或者阴历（农历）1971年5月9日之前出生的？（年龄在39岁及以上的人）

1-25: the list of household member exclude the nanny 给出前面的家户成员的列表

30: None

**Option 1: No eligible household member 第一种可能：没有适龄的家户成员**

**CONTINUE TO EXIT 退出**

We appreciate your time. There is no age-eligible person for us to interview this time. Thank you for your time!

谢谢您的配合，但这次您家里没有我们需要访问的适龄受访者。耽误您时间了，抱歉！

**Option 2: Have eligible household member(s) 第二种可能：这家有适龄的家户成员**

[Randomly select one age-eligible household member if there is more than one.]

[如果这个家中有不止一个适龄的家户成员，随机抽取一个。]

**[PROCEDURE: Member in Fourth Category is eligible to be main respondent, but Fifth Category is not.]**

**[程序：第四类家户成员可以被选为主要受访者，第五类不能.]**

**CV005.** **[IWER: Record Gender of Main Respondent. 访问员：记录此人的性别.]**

1. Male 男
2. Female 女

**MRbirth.** When was [MR] born? [主要受访者]的出生日期是？

_______1900..2011 Year年(MRbirth_year) ______0..12Month月(MRbirth_mon) ______0..31Day日(MRbirth_day)

[IWER: Mark the year using four digits. 访员注意: 用四位数字表示年份。]

**MRbirth_type.** Is your answer based on the solar or the lunar calendar? 您刚才回答的是公历（阳历）还是农历（阴历）？

1. Solar calendar 公历（阳历）
2. Lunar calendar 农历（阴历）

**[Show Card 出示卡片1]**

**CV033.** What is [preload MR]’s marital status? [主要受访者]目前的婚姻状态？

[IWER: common-law marriage is considered as married. 访员注意：即使没有领结婚证但自称结婚者也可视为已婚.]

(1）Married with spouse present 已婚并与配偶一同居住

(2）Married but not living with spouse temporarily for reasons such as work 已婚，但因为工作等原因暂时没有跟配偶在一起居住

（3）Separated 分居 （不再作为配偶共同生活）

（4）Divorced 离异

（5）Widowed 丧偶

（6）Never married 从未结婚

[PROCEDURE: if married, CV033 =1/2, then ask CV040]

[程序：如果已婚CV033 =1/2，询问CV040 ]

**CV040.** Who is [MR]’s spouse? [主要受访者]的配偶是？

1-25:the list of household member not including himself 给出前面的家户成员的列表，本人除外

30: not in the list，name 不在列表之中，姓名

**CV045.** **[IWER: confirm the gender of Main Respodent’s spouse 访问员：确认MR配偶的性别.]**

1. Male 男
2. Female 女

**Determine the Financial Respondent 选取财务受访者**

**CV031.** I will ask some questions about household financial status. Who would be most knowledgeable about this? Please tell me who will answer the financial module. 我下面将会问一些家庭财务情况的问题，请问在你们家中谁比较了解这方面的情况？

[**Please list all the household members, and pick one member from them. 列出所有家户成员的名单，从中选择]**

**Determine the Family Respondent 选取家庭情况受访者**

[PROCEDURE: If [Married with spouse present or living with a partner as if they were married] ask CV032 ]

[程序：如果对方已婚并与配偶一同居住，提问CV032]

**CV032.** I will ask some questions about family status. Who would be most knowledgeable about this, [MR] or [MR’s spouse]? 下面我将会问一些家庭情况的问题，请问在[主要受访者]和[主要受访者的配偶]谁比较了解这方面的情况？

(1) [MR] [主要受访者]

(2) [MR’s spouse] [主要受访者的配偶]

**[PROCEDURE: If the Main Respondet is 39 to 44, end this interview and begin contact sheet only]**

**[程序：如果选取的主要受访者年龄在39岁到44岁之间，结束访谈，仅开始问卷中“家户联系信息表”部分.]**

We appreciate your time. You are not our sample now, but you will become our sample in 6 years. Can you tell us your contact information so that we can keep in touch with you?

**[IWER：Pls start contact sheet. 访员注意：请填写“家户联系信息”表]**

谢谢您的配合，[主要受访者]不是我们这次调查的样本，但6年后[主要受访者]会成为我们的样本。您能告诉我们[主要受访者]的联系信息吗，这样我们能够在六年后再联系到他/她。

**[PROCEDURE: If the Main Respondet is 45 or older, continue the interview]**

**[程序：如果选取的主要受访者年龄在45岁及以上[(MR_age_year<1966 and MR_age_month<7 and MR_ager=1) or (MR_age_year<1966 and MR_age_month<6 and MR_ager=2)]，继续访谈.]**

[IWER: Coverscreen is completed. Please click <next> to back SMS to continue interview.]

[访员注意：已经做完了过滤问卷这部分。请点击<下一步>回到样本管理系统继续访问]

# **A HOUSEHOLD ROSTER 家户登记表**

[IWER: Please conduct this part of the interview (A Household roster) when the family respondent is at home. Don’t allow a proxy to complete the entire section. 访员注意：当家庭受访者在场时，才提问H家户登记表的问卷。这部分不允许请人完全代填。]

**A001.** [[IWER: Take down the type of this neighbourhood. 调查员记录地区类型]

1. Rural Village 农村
2. Urban Community 城镇社区

**[INTRO: Relatives can have important effects on your life. We’d like to ask you some questions about other members of your household: 引语：您的家人对您的生活可能会有重要影响，因此我们想问问除了您和您配偶外的其他家户成员的情况。]**

[IWER: The names of other household members, not including MR and spouse, are preloaded from the coverscreen information. Ask each household member the following questions: 访员注意：其他家户成员不包括主要受访者及其配偶，其他家户成员的名单要从过滤问卷获得。对于每一个其他家户成员，分别问下面的问题：]

***[[1]](#footnote-2)A002.**  Gender of this household member  [加载家户成员姓名]的性别是？

     （1）Male男

（2）Female女

***A003.** When was [name] born?  [姓名] 的出生日期是？

_______(A003_1)1900..2011 (HBirthyear)Year年 ______(A003_2)0..12 (HBirthmonth)Month月

**[Show Card 出示卡片1]**

***A004.** What is [name]’s marital status? [姓名] 目前的婚姻状态？

**[Notice： Common-law marriage is considered as married. 注意：即使没有领证但自称结婚者也可视为已婚。]**

1. Married with spouse present已婚并与配偶一同居住
2. Married but not living with spouse temporarily for reasons such as work 已婚，但因为工作等原因暂时没有跟配偶在一起居住

(3) Separated 分居 （不再作为配偶共同生活）

(4) Divorced 离异

(5) Widowed 丧偶

(6) Never married 从未结婚

**[PROCEDURE: If A004 =****3,4,5,6 and >=45years old, please ask A005]**

**[程序：如果A004 =3,4,5,6并且年龄大于等于45岁，请提问A005 ]**

**[Intro: It is common that many people who are not currently married often live with partners. Please bear me a question on this. The answer to this question will be kept strictly confidential and will be used for researchpurposes only. 现在有很多人虽然没结婚，但与别人同居。所以，想请您回答下面这个问题。请放心，我们将会对您的回答严格保密，并且仅用于学术研究。]**

**A005.** Is [name] unmarried but Living with a partner? [姓名]现在有没有和别人同居？

(1) Yes 有

(2) No 没有

****[[2]](#footnote-3)A006.** What is the relationship of [name] to you? [姓名]是您的？

1. Mother 母亲 Go to A009 请跳至A009
2. Father 父亲 Go to A009 请跳至A009
3. Mother-in-law 岳母/婆婆 Go to A009 请跳至A009
4. Father-in-law 岳父/公公 Go to A009 请跳至A009
5. Sibling 兄弟姐妹 Go to A009 请跳至A009
6. Brother-in-law, sister-in-law 姐夫妹夫/嫂子弟媳 Go to A009 请跳至A009
7. Child 孩子 Go to A007 请跳至A007
8. Spouse of child 儿媳/女婿 Go to A009 请跳至A009
9. Grandchild 孙子女 Go to A009 请跳至A009
10. Other relative（specify） 其他亲戚（请注明）_______(A006_1) Go to A009 请跳至A009

**[PROCEDURE: If CV033=1/2, please ask A007 ] [程序：如果 CV033 =1/2, 请提问A007]**

******A007.** What is the relationship between [name] and you? [姓名]是您和现在配偶的亲生孩子，还是别的？

1. The biological child of your and your (current) spouse.您和您（现在的）配偶的亲生孩子
2. The biological child of you, but not of your (current) spouse.您自己的亲生孩子，非现在配偶的。
3. The biological child of your (current) spouse, but not of yours.您（现在的）配偶自己的亲生孩子，不是您的。
4. The adopted or foster child of you or your spouse.您或您（现在的）配偶收养或抚养的孩子。

**[PROCEDURE: If CV033=3/4/5/6，please ask A008 ] [程序：如果CV033 =3/4/5/6，请提问A008]**

******A008.** What is the relationship between [name] and you? [姓名]是您自己的亲生孩子吗？

1. Your biological child. 您自己的亲生孩子
2. Your adopted or foster child or step child. 您收养或抚养的孩子或继子女。

****A009.** What is the current hukou status of [name]? [姓名]目前的户口状态是？

(1) Agriculture Hukou 农业 Skip to A012 .跳转至A012

1. Non-Agriculture Hukou 非农业 Skip to A012 .跳转至A012
2. Unified Residency Hukou 统一居民户口

[IWER: for the place where agricultural hukou is abandoned. 访员注意：对取消农村户口的地方]

1. Do not have Hukou 没有户口 Skip to A013 .跳转至A013

F1：“统一居民户口”指的是某些地方实行户口制度改革后，不再区分农业与非农业户口，而是统一为“居民户口”。

****A010.** What is [name]’s Hukou status before he/she has the unified residency hukou? [姓名]在获得统一居民户口之前是什么户口？

(1) Agriculture Hukou 农业

1. Non-Agriculture Hukou 非农业
2. Do not have Hukou 没有户口

F1：“统一居民户口”指的是某些地方实行户口制度改革后，不再区分农业与非农业户口，而是统一为“居民户口”。

****A011.** When did [name] have the unified residence Hukou? [姓名]什么时候获得统一居民户口？

________ 2000..2011year 哪年

F1：“统一居民户口”指的是某些地方实行户口制度改革后，不再区分农业与非农业户口，而是统一为“居民户口”。

****A012.** What is the location of [name]‘s current hukou? [姓名]目前户口所在地？

(1) This household 在这个家里

(2) This village/neighborhood 在本村/社区

(3) Another village/neighborhood in this county/city 在本县/市的其他村/社区

(4) Another county/city in this province在本省的其他县/市，___________(A012_1)city市__________ (A012_2) county县

(5)Another province 外省：_______(A012_3)province 省/自治区/直辖市, _____ _______(A012_4)city市_____ ______(A012_5) county县00000000000000000000000000000000000000000000000000000000000000000000000000000000000000000000000000000000000000000000000000000000 136___别是？0000000000000000000000000000000000000000000000000000000000000000000000000000000000000000000000000000000000000000000000[IWER: Choose from the list of provinces，see appendix 2 请选择省份，见附表二]

(6) Abroad 国外

**[PROCEDURE: If A006=7, ask A021. 程序：如果A006=7(此人为受访者的子女)，请询问A021]**

******A021.** Where was the birth place of CHILD's NAME? [孩子姓名]出生地是？

(1) This village/neighborhood 在本村/社区

(2) Another village/neighborhood in this county/city 在本县/市的其他村/社区

(3) Another county/city in this province在本省的其他县/市，___________(A021_1)city市__________ (A021_2) county县

(4)Another province 外省：_______(A021_3)province 省/自治区/直辖市, _____ _______(A021_4)city市_____ ______(A021_5) county县00000000000000000000000000000000000000000000000000000000000000000000000000000000000000000000000000000000000000000000000000000000 136___别是？0000000000000000000000000000000000000000000000000000000000000000000000000000000000000000000000000000000000000000000000[IWER: Choose from the list of provinces，see appendix 2 请选择省份，见附表二]

(5) Abroad 国外

******A022.** Is [name]’s present hukou status and location the same as his/her first Hukou?  [孩子姓名]目前户口与他出生时获得的户口是否一样？

1. Yes 是 →Skip to precedure before A013 请跳至A013前的程序控制
2. No 否

******A023.** How did [name]’s Hukou status or location change?

[孩子姓名]的户口发生过什么变化？

(1)Both Hukou status and location have changed 户口类型和户口所在地都变化过

(2)Only Hukou status has changed 只有户口类型变化

(3)Only Hukou location has changed 只有户口所在地变化 →Skip to A025 请跳至A025

******A024.** What was [name]’s first HuKou status? [孩子姓名] 第一个户口的类型是？

1. Agricultural Hukou 农业
2. Non-agricultual Hukou 非农业

[If A023=2, skip A025. 如果A023=2，跳过A025]

******A025.** What was the location of [name]‘s first hukou? [孩子姓名]第一个户口所在地？

(1) This village/neighborhood 在本村/社区

(2) Another village/neighborhood in this county/city 在本县/市的其他村/社区

(3) Another county/city in this province在本省的其他县/市，___________(A025_1)city市__________ (A025_2) county县

(4)Another province 外省：_______(A025_3)province 省/自治区/直辖市, _____ _______(A025_4)city市_____ ______(A025_5) county县00000000000000000000000000000000000000000000000000000000000000000000000000000000000000000000000000000000000000000000000000000000 136___别是？0000000000000000000000000000000000000000000000000000000000000000000000000000000000000000000000000000000000000000000000[IWER: Choose from the list of provinces，see appendix 2 请选择省份，见附表二]

(5) Abroad 国外

**[PROCEDURE: If the person is less than 6 years old, skip to the next person 程序：如果这人年龄不到6岁，请接着询问下一个人]**

****A013.** Is [name] still in school now? 请问[姓名]现在还在上学吗？

1. Yes 是
2. No 否 →Skip to A0015 请跳至A015

00000000000000000000000000000000000000000000000000000000000000000000000000000000000000000000000000000000000000000000000000000000 136___别是？0000000000000000000000000000000000000000000000000000000000000000000000000000000000000000000000000000000000000000000000[IWER: Fulltime student, not on-the-job student. 访员注意：指全职读书，在职不算]

****A014.** What level of schooling and grade is [name] currently enrolled in? [姓名]现在读几年级？

(1) Primary school grade 1 小学1年级

(2) Primary school grade 2 小学2年级

(3) Primary school grade 3 小学3年级

(4) Primary school grade 4 小学4年级

(5) Primary school grade 5 小学5年级

(6) Primary school grade 6 小学6年级

(7) Middle school grade 1 初中1年级

(8) Middle school grade 2 初中2年级

(9) Middle school grade 3 初中3年级

(10) Middle school grade 4 初中4年级

(11) High school, grade 1 高中1年级

(12) High school, grade 2 高中2年级

(13) High school, grade 3 高中3年级

(14) Vocational/technical high school year 1 中专1年级

(15) Vocational/technical high school year 2 中专2年级

(16) Vocational/technical high school year 3 中专3年级

(17) College year 1 大专/大学1年级

(18) College year 2 大专/大学2年级

(19) College year 3 大专/大学3年级

(20) College year 4 大专/大学4年级

(21) College year 5 大专/大学5年级

(22) College year 6 大专/大学6年及6年级以上

(23) Master’s degree 硕士

(24) Doctoral degree/ Ph.D. degree 博士

**[PROCEDURE:Skip to procedures befor A016，程序：请跳转至A016 前的程序控制 ]**

**[PROCEDURE: If the person is less than 12 years old, skip to the next person 程序：如果这人年龄不到12岁，请询问下一个人]**

****A015.** What is the highest level of education completed? [姓名]完成的最高学历是？

(1) No formal education (illiterate)  未受过正式教育（文盲）

(2) Did not finish primary school but capable of reading or writing 未读完小学，但能够读、写

(3) Sishu/home school 私塾

(4) Graduate from elementary school 小学毕业

(5) Graduate from middle school 初中毕业

(6) Graduate from high school  高中毕业

(7) Graduate from vocational school 中专（包括中等师范、职高）毕业

(8) Graduate from Two/Three Year College / Associate degree 大专毕业

(9) Graduate from Four Year College / Bachelor’s degree 本科毕业

(10) Graduate from Post-graduate, Master’s degree 硕士毕业

(11) Graduate from Post-graduate, Doctoral degree/Ph.D. 博士毕业

**[PROCEDURE: If the person is less than 16 years old, skip to the next person 程序：如果这人年龄不到16岁，请询问下一个人]**

****A016.** Did [name] spend one or more months away from the household in the past year?

过去一年，[姓名] 在外居住的时间有没有达到或者超过一个月？

(1) Yes 是

(2) No 否 Skip A017, A018, A019 请跳过A017, A018, A019

****A017.** How many months in the past year did [name] live away from home?

过去一年，[姓名]在外居住了几个月？____ 0..12months 月

**[Softcheck： if “0” or missing is reported in A017 and yes is checked in A016 . 如果A017 答案为0或者缺失，并且A016 =1，则进行检查。]**

****A018.** Where is the main place that [name] lived during his/her time away? [姓名]在外居住时主要呆在哪儿呢？

(1) This county/city本县/市

(2) Another county/city in this province在本省的其他县/市，___（A018_1）city市____（A018_2） county县

(3)Another province 外省：________(A018_3)province 省/自治区/直辖市，___________(A018_4)city市___________(A018_5) county县00000000000000000000000000000000000000000000000000000000000000000000000000000000000000000000000000000000000000000000000000000000 136___别是？0000000000000000000000000000000000000000000000000000000000000000000000000000000000000000000000000000000000000000000000

[IWER: Choose from the list of provinces，see appendix 2. 访员注意： 请选择省份，见附表二]

(4) Abroad国外

****A019.** What type of location did [name] live in? [姓名]在外居住的地方属于什么行政级别?

(1) City 城市

(2) County 县城

(3) Town乡镇

(4) Village 村庄

**[PROCEDURE: Go/Proceed to the next person 程序：请询问下一个人]**

**A020.** How often did the respondent receive assistance in answering section A-HOUSEHOLD ROSTER? 受访者填写A部分问卷时是否求助？

[[IWER: If it is answered by a proxy, please record the respondent’s reaction. 访员注意：如果是协助回答，请记录受访者的反应。]

1. Never 从未
2. A few times 偶尔几次

(3) Most or all of the time 大多数

# **B 基本信息**

| NOTE: ONLY THOSE WHO WERE BORN BEFORE JULY 1, 1966, AND THEIR SPOUSES WILL BE INTERVIEWED. 注意：1966年7月1日之前出生的人及其配偶需要做下列问卷。 |
| --- |

**[Show Card 出示卡片2]**

**BA001.** What is your Chinese Zodiac sign? 您的属相是？_________________

[IWER: Choose from the list of Chinese Zodiac signs，see Appendix 1请选择属相，选项见附表一。]

**BA002.** When were you born? 您的出生日期是？

_______ 1900..2011 (BA002_1 )year 年 ______0..12 (BA002_2 ) month月______0..31 (BA002_3 )day 日

[IWER：The year must be a number in the range [1900 – 2011]. Mark the year using four digits. Take down the month as its actual number. For example, write January as “1” not “01”, December as “12”. If do not remember month and day, fill ‘0’. 访员注意：年份填写范围[1900－2011]。用4位数表示年，按照实际的月份填写月。例：1月写作“1”，而不是“01”,12月写作“12”。如果记不住月份和日期，请填入’0’。]

[CAPI: Check date of birth by Zodiac.]

[If the person does not know the date of birth, BA002 =DK or BA002 =RF, skip BA003. 如果受访者不知道出生日期即BA002 选择不知道或拒绝回答，跳过BA003 .]

**BA003.** Is your answer to BA002 based on the solar or the lunar calendar? 您刚才回答的是公历（阳历）还是农历（阴历）？

1. Solar calendar 公历（阳历）
2. Lunar calendar 农历（阴历）

[If the person does not know the date of birth, BA002 =DK or BA002 =RF, ask BA004 如果受访者不知道出生日期即BA002 选择不知道或拒绝回答，问BA004 .]

**BA004.** What is your age? 您今年多大年纪？________1…120years old 岁

[IWER: You can refer to the year for some major events or born in which year during the republic of China. (begin in 1912) 访员注意：可以提示受访者一些大事件发生的时间或者民国出生哪一年(民国元年为1912年)。]

F1：“民国元年”为1912年。民国年数+1911=公元年

**BA005.** Do you attend school/work away from home but return to this home almost every week? 您是不是平时上学/上班，但通常每周末回这个家？

(1) Yes 是

(2) No 否

**[INTRO: Next are some questions about your birth place, some changes in your housing location, your Hukou and education. 接下来我们将问到您的出生地、居住地的变化情况，以及您的户口和受教育程度。]**

**BB001.** Where were you born? 您的出生地是哪里？

1. This village/neighborhood 本村/社区 Skip to BB006 请跳至BB006
2. Another village/neighborhood in this county/city 本县/市的其他村/社区 Skip to BB005 请跳至BB005
3. Another county/city in this province在本省的其他县/市，_____(BB001_1)city市_____(BB001_2) county县
4. Another province 外省：_____(BB001_3)province 省/自治区/直辖市，_______(BB001_4)city市________(BB001_5)county县00000000000000000000000000000000000000000000000000000000000000000000000000000000000000000000000000000000000000000000000000000000 136___别是？0000000000000000000000000000000000000000000000000000000000000000000000000000000000000000000000000000000000000000000000

[IWER: Choose from the list of provinces，see Appendix 2 请选择省份，见附表二]

1. Abroad 国外

**BB002.** What is the type of your birth place? Is it rural village or urban community? 您出生地的类型是什么？是农村还是城镇社区？

1. Rural Village 农村
2. Urban Community 城镇社区

**BB003.** When did you first live in this county/city? 您第一次搬到本县/市是在什么时候？

_________ 1900..2011 Year 年

[IWER: Mark the year using four digits. 请用4位数表示年。]

**BB004.** When you first moved to this county/city, did you live in the same village/community as you currently do? 您第一次搬到本县/市，就是住在当前村/社区吗？

1. Yes 是 skip BB005 跳过BB005
2. No 否

**BB005.** In what year did you first live in this village/neighborhood? 您第一次搬来本村/社区居住是在什么时候？___________ 1900..2011Year 年

[IWER: Mark the year using four digits. 请用4位数表示年。]

**BB006.** Where did you mainly live before you were 16 years old? Is it in village or city/town? 16岁以前，您主要住在农村还是城镇？

[IWER: City or Village when the person was living there. 受访者当时住的时候是农村还是城镇。]

1. Village 农村
2. City/Town 城镇

**[PROCEDURE: If BB001 =1 or BB001 =2 go to BB009 ; else go to BB007] [程序：如果BB001 =1 or BB001 =2，跳至BB009 ；否则请继续BB007]**

**BB007.** Ever since you first came to this county/city, have you ever lived outside your present county/city for more than 6 months? 自从您第一次搬到这个县/市以来，您有没有在本县/市以外的地方连续住过六个月以上？

1. Yes是
2. No否 → Go to BC001 请跳至BC001

**BB008.** Ever since you first came to this county/city, how long have you lived outside this county/city in total? 自从您第一次搬到这个县/市以来，您在其他县/市一共住了多长时间？ ________(BB008_1)years 年________(BB008_2)months 月

**Skip to BB011 请跳至BB011**

**BB009.** Have you ever lived outside this county/city for more than 6 months? 您曾在本县/市以外的地方连续住过六个月以上吗？

1. Yes是
2. No否 →Skip to BC001 请跳至BC001

**BB010.** How long have you lived outside this county/city in total? 您在本县/市以外的地方一共住了多长时间？

________(BB010_1)years 年________(BB010_2)months 月 [hard check: years <= 2011-BB003 此处的年份应该<= BB003 ]

**BB011.** When did you most recently live in this county/city (after being away for 6 months or longer)? 您最近一次搬到本县/市居住是在什么时候（指最近一次六个月或以上外出之后）？______ 1900..2011year 年[IWER: Mark the year using four digits. 访员注意：用4位数表示年。]

[hard check: year >=BB003 此处的年份应该>= 2011-BB003 ]

**[Show Card 出示卡片3]**

**BB012.** Before moving to the current county/city, where else did you live for at least six months? (Not less than six months, otherwise ask about previous locations where the respondent lived for at least six months) 搬到本县/市之前，您连续住过六个月以上的地方是哪里（必须至少六个月，否则问上一个超过六个月的居住地点）？

1. Birth place 出生地
2. Another county/city in this province在本省的其他县/市，______(BB012_1)city市______(BB012_2) county县
3. Another province 外省：______(BB012_3)province 省/自治区/直辖市，___________(BB012_4city)市___________(BB012_5)county县00000000000000000000000000000000000000000000000000000000000000000000000000000000000000000000000000000000000000000000000000000000 136___别是？0000000000000000000000000000000000000000000000000000000000000000000000000000000000000000000000000000000000000000000000

[IWER: Choose from the list of provinces，see appendix 2 请选择省份，见附表二]

1. Abroad国外

**BC001 .** What is your current HuKou status? 目前您的户口类型是？

1. Agricultual Hukou 农业 Skip to BC004 请跳至BC004
2. Non-agricultural Hukou 非农业 Skip to BC004 请跳至BC004
3. Unified Residence Hukou 统一居民户口

[IWER: for the place where agricultural hukou is abandoned. 访员注意：对取消农村户口的地方]

1. Do not have Hukou没有户口 Skip to BC009 跳至BC009

F1：“统一居民户口”指的是某些地方实行户口制度改革后，不再区分农业与非农业户口，而是统一为“居民户口”。

[If BC001 =3, ask BC002 to BC003. 如果受访者为统一居民户口，询问BC002 到BC003]

**BC002.** What is your Hukou status before you have the unified residence Hukou? 您在获得统一居民户口之前是什么户口？

(1) Agricultural Hukou 农业户口

(2) Non-agricultual Hukou 非农业户口

(3) Do not have Hukou 没有户口

F1：“统一居民户口”指的是某些地方实行户口制度改革后，不再区分农业与非农业户口，而是统一为“居民户口”。

**BC003.** When did you have the unified residence Hukou? 您什么时候获得统一居民户口？

________(BC003_1)1900..2011year 哪年________(BC003_2)0..12 month 月

F1：“统一居民户口”指的是某些地方实行户口制度改革后，不再区分农业与非农业户口，而是统一为“居民户口”。

Go to BC005 请跳至BC005

**BC004.** When did you get your current Hukou? 您是什么时候得到目前的这个户口？

_____1900..2011(BC004_1)year 年________0..12 (BC004_1)month 月 [hard check: enforce year>=BA002_1 此处的年份应该≥BA002_1 (出生年份)]

[IWER: You must fill year[1900 - 2011]. Mark the year using four digits. Take down the month as its actual number. For example, write January as “1” not “01”, December as “12”. If do not remember month, fill ‘0’. 访员注意: 年份填写范围[1900 - 2011]。用4位数表示年，按照实际的月份填写月。例：1月写作“1”，而不是“01”,12月写作“12”。如果记不住月份，请填入’0’。]

**BC005.** Where is your current HuKou? 目前您的户口所在地是？

[IWER: If birthplace is in this village/neighborhood, pls choose the second choice. 如果出生地是本村或社区，此处请选择第二个选项]

1. Same as birthplace 和出生地一样
2. This village/neighborhood 本村/社区
3. Another village/neighborhood in this county/city 本县/市的其他村/社区
4. Another county/city in this province在本省的其他县/市，_______(BC005_1)city市______(BC005_2) county县
5. Another province 外省：________(BC005_3)province 省/自治区/直辖市，___________(BC005_4)city市___________(BC005_5)county县00000000000000000000000000000000000000000000000000000000000000000000000000000000000000000000000000000000000000000000000000000000 136___别是？0000000000000000000000000000000000000000000000000000000000000000000000000000000000000000000000000000000000000000000000

[IWER: Choose from the list of provinces，see appendix 2 请选择省份，见附表二]

[Procedure：If (BC005 =1 and BB001 =3. 4.5) or BC005 =4 or BC005 =5，please ask BC006 . 如果(BC005 =1且 BB001 =3.4.5) 或BC005 =4或BC005 =5，请询问BC006 ]

**BC006.** How long have you been away from your current Hukou county/city?

您离开目前户口所在县/市的时间有多久了？ ______（BC006_1）Years年_________（BC006_2）Months 月

[hard check: impose that years<=2011-BA002_1 (birthyear) 此处的年份应该≤2011-BA002_1 (出生年份)]

**BC007.** Is your first Hukou same as your current Hukou?（Not including the change of unified Residence Hukou.） 您的第一个户口就是您目前的户口吗？（统一居民户口的变化不算变化。）

(1) Yes 是 Go to BD001 请跳至BD001

(2) No 否

**BC008.** Were there any other Hukou between your first Hukou and your current Hukou?(Including changes of hukou type and place. Hukou type change only means changes between Agricultural Hukou and non-agricultural hukou.) 第一个户口与目前户口之间，您还有过其他户口吗？（户口变化指户口类型或户口所在县市的变化，其中户口类型变化只指农业与非农业户口的变化。）

1. Yes 有
2. No 没有

**BC009.** Has your Hukou type or place ever changed since your first Hukou? (Hukou type change only means changes between Agricultural Hukou and non-agricultural hukou.) 从第一个户口开始，您的户口类型或户口所在地发生过什么变化？（户口类型变化指农业与非农业户口的变化。）

(1)Both Hukou type and place have changed 户口类型和户口所在地都变化过

(2)Only Hukou type has changed 只有户口类型变化

(3)Only Hukou place has changed 只有户口所在地变化

[If BC009 =1or 2, ask BC010, if BC009 =3. skip BC010. 如果BC009 =1或2, 提问BC010 .如果BC009 =3，跳过BC010 ]

**BC010.** What was your first HuKou status? 您拿到的第一个户口的类型是？

1. Agricultural Hukou 农业
2. Non-agricultual Hukou 非农业

[If BC008 =1 and (BC009 =1 or 3), ask BC011 ,else skip BC011. 如果BC008 =1且BC009 =1或3，提问BC011 , 否则跳过BC011]

**BC011.** Where was your last HuKou? 您上一个户口所在地是？

[IWER: If last Hukou is same as birthplace and is in another village/neighborhood in this county/city, then choose 1. 访员注意：如果上一个户口所在地与出生地一样，都在本县市其他村/社区，那么请选1。]

1. Same as birthplace 与出生地一样

(2) Another village/neighborhood in this county/city 本县/市的其他村/社区

(3) Another county/city in this province在本省的其他县/市，____(BC011_1)city市____(BC011_2) county县

(4)Another province外省：___ (BC011_3)province 省/自治区/直辖市，___(BC011_4)city市___ _(BC011_6) county县

00000000000000000000000000000000000000000000000000000000000000000000000000000000000000000000000000000000000000000000000000000000 136___别是？0000000000000000000000000000000000000000000000000000000000000000000000000000000000000000000000000000000000000000000000[IWER: Choose from the list of provinces，see appendix 2 请选择省份，见附表二]

[If BC011 =2 or 3 or 4, ask BC012 , else go to the procedure before BC013. 如果BC011 =2 或 3 或 4，提问BC012，否则跳至BC013 前的程序控制。]

**BC012.** Why the location of current Hukou is different from your first Hukou? 为什么上一个户口所在地与出生地不一样？

1. marriage 结婚
2. go to school 上学
3. employment 就业
4. retirement/ revolutionary retirement 退休/离休
5. escape of famine 逃荒
6. sent down to the countryside to do manual labor上山下乡
7. migration of the whole village 整村搬迁
8. others, pls specify____ 其它，请注明____（BC012_1）

[If BC009 =1, ask BC013, then go to procedure before BC015; If BC009 =3, ask BC013 and BC014. If BC009 =2, go to procedure before BC015. 如果BC009 =1, 提问 BC013，然后跳至BC015前的程序控制；如果BC009 =3, 提问BC013 和BC014 . 如果BC009 =2, 跳至BC015 前的程序控制。]

**BC013.** What was the location of your first HuKou? 您第一个户口的所在地是？

[IWER: If first Hukou is same as birthplace or current Hukou, and is same as other choices, then choose 1 or 2. 访员注意：如果第一个户口所在地与出生地或目前户口所在地一样，而且同时也可以说在本村/社区、本县市其他村/社区，那么请选1或2。]

1. Same as birthplace 与出生地一样
2. Same as current Hukou 与目前户口所在地一样
3. This village/neighborhood 本村/社区
4. Another village/neighborhood in this county/city 本县/市的其他村/社区
5. Another county/city in this province在本省的其他县/市，___________(BC013_1)city市___________(BC013_2) county县
6. Another province 外省：_______(BC013_3)province 省/自治区/直辖市，_________ (BC013_4)city市________( BC013_5) county县00000000000000000000000000000000000000000000000000000000000000000000000000000000000000000000000000000000000000000000000000000000 136___别是？0000000000000000000000000000000000000000000000000000000000000000000000000000000000000000000000000000000000000000000000

[IWER: Choose from the list of provinces，see appendix 2 请选择省份，见附表二]

[If (BC009 =1 or 3 and BC013 =3 or 4 or 5 or 6) or (BC009 =1 or 3 and BC013 =1 and BC005 ≠1), ask BC014 , else go to the procedure before BC015. 如果(BC009 =1或3且BC013 =3或4或5或6) 或 (BC009 =1或3且 BC013 =1且BC005 ≠1)，提问BC014, 否则跳至BC015前的程序控制。]

**BC014.** Why the location of current Hukou is different from your first Hukou? 为什么第一个户口所在地与目前户口所在地不一样？

1. marriage 结婚
2. go to school 上学
3. employment 就业
4. retirement/ revolutionary retirement 退休/离休
5. sent down to the countryside to do manual labor上山下乡
6. migration 搬迁
7. migration of the whole village 整村搬迁
8. escape of famine 逃荒
9. others, pls specify____ 其它，请注明____(BC014_1)

[If BC001 =1 and BC009 =1 or 2, ask BC015 to BC016 , then skip to BD001 .如果受访者为农业户口且户口类型发生过变化，询问BC015 到BC016 ，然后跳至BD001 .]

**BC015.** What is the reason that you had the non-agricultural Hukou? 您有过非农业户口是因为什么情况？

1. sent down during 1967-1977 and did not come back to city 1967-1977年之间下乡后来没回城
2. was a student 上学
3. marriage 结婚
4. employment 就业
5. migration of the whole village 整村搬迁
6. escape of famine 逃荒
7. others,pls specify ______ 其它,请注明______(BC015_1)

**BC016.** When did you have the non-agricultural Hukou? 您什么时候有非农业户口？

From________(BC016_1)1900..2011year to ________(BC016_2)1900..2011year 从哪年到哪年

[If BC001=2 and BC009=1 or 2, ask BC017 to BC018, 如果受访者为非农业户口且户口类型发生过变化，询问BC017 到BC018.]

**BC017.** What is the reason that you had the agricultural Hukou? 您有过农业户口是因为什么情况？因为在农村长大还是其他？

1. sent down during 1967-1977 and then come back to city 1967-1977年之间下乡后来回城
2. live in rural areas before go to college student 得到非农户口前在农村居住
3. marriage 结婚
4. employment 就业
5. Land is acquired by the government 土地被征用
6. migration of the whole village 整村搬迁
7. escape of famine 逃荒
8. others ,pls specify ______ 其它,请注明______(BC017_1)

**BC018.** When did you have the agricultural Hukou? 您什么时候有农业户口？

From________(BC018_1)1900..2011year to ________(BC018_2)1900..2011year 从哪年到哪年

**BD001.** What is the highest level of education you have attained? 您获得的最高学历是？

1. No formal education (illiterate)  未受过教育（文盲） → Skip to BD007 请跳至 BD007
2. Did not finish primary school but capable of reading and/or writing 未读完小学，但能够读、写
3. Sishu/home school 私塾
4. Elementary school 小学毕业
5. Middle school 初中毕业
6. High school  高中毕业
7. Vocational school 中专（包括中等师范、职高）毕业
8. Two-/Three-Year College/Associate degree 大专毕业
9. Four-Year College/Bachelor’s degree 本科毕业
10. Master’s degree 硕士毕业
11. Doctoral degree/Ph.D. 博士毕业

**[Procedure: If BD001 =2, ask BD002. 如果BD001 =2，提问BD002 ]**

**BD002.** What is the highest grade did you finish in primary school ？ 您小学读到____ 1..6年级？

Skip to BD005 请跳至BD005

**[Procedure: If BD001 >=4, ask BD003. 如果BD001 >=4，提问BD003 .]**

**BD003.** How many additional years of schooling did you complete after [THE ANSWER CHOSEN IN BD001 ]? 您在[BD001的答案]之后又上过几年级学？______ years 年

**[PROCEDURE: If BD001 >7, ask BD004 ] [程序：如果BD001 >7，问BD004 ]**

**BD004.** When did you go to college? 您从哪一年开始读大学/大专?_______ 1900..2011year年

[IWER：if there are skipped years, fill in the years needed for the grade rather than the actual number of years; if there are repeated years, fill in the years needed for the grade rather than the actual number of years.访员注意：如果有跳级或者留级的情况，填完成相应学位需要的时间而不是实际的年数。]

**BD005.** At what age did you begin formal schooling? 您从几岁开始上小学/私塾？

_____1…120years old 岁 [soft check, <6, >11]

**BD006.** At what age did you finish schooling? 您几岁读完书？_____1…120years old 岁

[IWER：It asks age when R finishes schooling, not age when R finished elementary school.访员注意：此处询问的是停止上学的年龄，并非读完小学/私塾的年龄。]

**BD007.** Have you ever attended school for adult education? 您曾经参加过成人教育（如电大，夜校，自考，函授，扫盲班，速成班）吗? （可多选）

- 1. None 没有 Go to BE001 请跳至 BE001
  2. TV University 电大
  3. Night School 夜校
  4. Zikao (examinations for self-taught students)自考
  5. Hanshou/Correspondence course/Distance learning 函授
  6. Literacy course 扫盲班
  7. Accelerated education course 速成班
  8. Other (explain: )其他，请注明__________ (BD007_1 )

**BD008.** How many years did you spend in adult education? 您成人教育一共读了几年？

_______ year 年 [soft check >9]

**BD009.** Did you get a diploma or degree from the adult education program you attended? 您从成人教育中获得了学位或文凭吗？

1. Yes 是
2. No　否 Go to BE001 跳至BE001

**BD010.**When did you receive the diploma? 您什么时候获得该学位或者文凭？________1900..2011year年

**BD011.** What is the highest level of schooling you obtained from the adult education program? 您获得最高的成人教育学位或文凭是什么？

1. Vocational school 中专
2. Two/Three Year College / Associate degree 大专
3. Four Year College / Bachelor’s degree 本科
4. Others 其他

**[Show Card 出示卡片1]**

**BE001.** RMaritalStatus: What is your marital status? 您目前的婚姻状态？

[IWER: common-law marriage is considered as married 即使没有领结婚证但自称结婚者也可视为已婚]

1. Married with spouse present 已婚与配偶一同居住
2. Married but not living with spouse temporarily for reasons such as work 已婚，但因为工作等原因暂时没有跟配偶在一起居住

(3) Separated 分居 （不再作为配偶共同生活）

(4) Divorced 离异

(5) Widowed 丧偶

(6) Never married 从未结婚

**[PROCEDURE: If BE001 =3/4/5/6, please ask BE002]**

**[程序：如果BE001 =3/4/5/6, 请提问BE002 ]**

**[Intro: It is common that many people who are not currently married often live with partners. Please bear me a question on this. The answer to this question will be kept strictly confidential and will be used for researchpurposes only. 现在有很多人虽然没结婚，但与别人同居。所以，想请您回答下面这个问题。请放心，我们将会对您的回答严格保密，并且仅用于学术研究。]**

**BE002.** Are you unmarried but Living with a partner? 那您现在有没有和别人同居？

(1) Yes 有

(2) No 没有

**[PROCEDURE: If the person has never married (BE001 =6(never married), then skip to BF008 ] [如果受访者未婚(BE001 =6)，请跳至BF008 ]**

**[INTRO: Many people have more than one marrige through whole life. Please bear me a few more questions on this.很多人一生中不止结过一次婚，所以，我们想请您回答以下这些婚姻题目。]**

**BE003.** How many times have you been married? 您到目前为止结过几次婚？_______ times 次 [soft check >2]

**[PROCEDURE:If the person married only once, continue with BE004-BE005 , then go to the procedure before BF001 ] [如果受访者只结过一次婚，继续回答BE004-BE005，然后跳至BF001 前的程序控制]**

**[If the person married more than once, continue with BE006 and BE009, 如果受访者不只结过一次婚，继续回答BE006 和BE009 ]**

**BE004**.When did you get married? 你什么时候结的婚？

_____1900..2011 (BE004_1) year 年________ 0..12 (BE004_2 )month 月[soft check: year>BA002_1 +16 此处的年份应该>BA002_1 +16]

[IWER: You must fill year [1900 - 2011]. Mark the year using four digits. Take down the month as its actual number. For example, write January as “1” not “01”, December as “12”. If do not remember month, fill ‘0’. 访员注意: 年份填写范围[1900 - 2011]。用4位数表示年，按照实际的月份填写月。例：1月写作“1”，而不是“01”,12月写作“12”。如果记不住月份，请填入’0’。]

**BE005.** When you were married, what was the total value of cash and goods (including housing) that your parents gave to you and your spouse? 您结婚时，您父母总共给了您和您的配偶多少价值的现金和物品（包括房子）？___________Yuan 元

**Skip to procedure before BF001 跳至BF001 前的程序控制**

**BE006.** When did you marry the first time? 你第一次是什么时候结的婚？

_____ 1900..2011 (BE006_1) year 年_______________0..12(BE006_2) month月[soft check: year>BA002_1 +16此处的年份应该>BA002_1 +16]

[IWER: You must fill year [1900 - 2011]. Mark the year using four digits. Take down the month as its actual number. For example, write January as “1” not “01”, December as “12”. If do not remember month, fill ‘0’. 访员注意: 年份填写范围[1900 - 2011]。用4位数表示年，按照实际的月份填写月。例：1月写作“1”，而不是“01”,12月写作“12”。如果记不住月份，请填入’0’。]

**BE007.** In what year did your first marriage end? 您的第一次婚姻是什么时候结束的？ 1900..2011Year [hard check: enforce >=BE006 此处的年份应该>=BE006 ]

**BE008.** Why did your first marriage end? 为什么会结束第一次婚姻？

1. death of spouse 配偶去世了
2. divorce 离婚

**BE009 .**When was your most recent marriage? 你最近一次是什么时候结的婚？

_____1900..2011(BE009_1) year 年________ 0..12 (BE009_2)month 月

[hard check: enforce year >=BE007 此处的年份应该>=BE007 ]

[IWER: You must fill year[1900 - 2011]. Mark the year using four digits. Take down the month as its actual number. For example, write January as “1” not “01”, December as “12”. If do not remember month, fill ‘0’. 访员注意: 年份填写范围[1900 - 2011]。用4位数表示年，按照实际的月份填写月。例：1月写作“1”，而不是“01”,12月写作“12”。如果记不住月份，请填入’0’。]

**[PROCEDURE: Based on the answer to BE001, If the person is married with spouse present, skip to BF008. ] [程序：根据BE001 的答案编程]如果受访者目前有配偶(BE001=1 / 2)，跳至BF008 ]**

**[If the person is married but not living with spouse for separated, divorced, or widowed, then ask them to answer BF001 to BF007 ].[如果受访者与配偶分居、离异或者丧偶(BE001=3/4/5)，回答BF001 至BF007 ]**

[IWER: I’d like to ask you a few questions about your current (or most recent) spouse/partner:

访员注意：这里有几个关于受访者配偶的问题。若受访者属于结婚多次，则问最近一次婚姻的的配偶]

**[INTRO: An important part of this study is understanding how people make decisions during different stages of life, we also need to a general age range for your spouse.了解人们在不同的人生阶段如何进行决策是我们研究的一个重要方面，因此接下来我们会问到您配偶的年龄。]**

**[Show Card 出示卡片2]**

**BF001.** What is your spouse/partner’s Chinese Zodiac sign? 您配偶的属相是？______

[IWER: Choose from the list of Chinese Zodiac signs，see Appendix 1 请选择属相，选项见附表一。]

**BF002.** When was your spouse/partner born? 您配偶的出生日期是？

_______1900..2011 (BF002_1 )year 年______0..12 (BF002_2 )month 月[preload spouse‘s birthdate]

[IWER: Mark the year using four digits. Take down the month as its actual number. For example, write January as “1” not “01”, December as “12”. If do not remember month, fill ‘0’. 访员注意: 用4位数表示年，按照实际的月份填写月。例：1月写作“1”，而不是“01”,12月写作“12”。如果记不住月份，请填入’0’。]

**BF003.** Is your spouse/partner‘s date of birth based on the solar calendar or the lunar calendar? 您刚才答的是公历（阳历）还是农历（阴历）？

1. Solar calendar 公历（阳历）
2. Lunar calendar 农历（阴历）

**BF004.** What is the highest level of education your spouse/partner has attained? 您配偶获得的最高学历是？[Note: Please refer to my corrections/suggestions in the previous section with similar questions]

1. No formal education (illiterate)  未受过教育（文盲）
2. Did not finish primary school but capable of reading or writing 未读完小学，但能够读、写
3. Home School 私塾
4. Elementary school 小学毕业
5. Middle school 初中毕业
6. High school  高中毕业
7. Vocational school 中专（包括中等师范、职高）毕业
8. Two/Three Year College / Associate degree 大专毕业
9. Four Year College / Bachelor’s degree 本科毕业
10. Post-graduate, Master’s degree 硕士毕业
11. Post-graduate, Doctoral degree/Ph.D. 博士毕业

**[If the person is separated, ask BF005 如果受访者与配偶分居，问BF005 ]**

**[If the person is divorced, ask BF006 如果受访者离异，问BF006 ]**

**[If the person is widowed, ask BF007 如果受访者丧偶，问BF007 ]**

**BF005.** When did you separate? 你们什么时候分居的？______1900..2011 (BF005_1)year 年 ______0..12 (BF005_2 )month 月[hard check: enforce year>year married (BE004 if _BE003 =1. BE009 if BE003 >1)]

[IWER: Mark the year using four digits. Take down the month as its actual number. For example, write January as “1” not “01”, December as “12”. If do not remember month, fill ‘0’. 访员注意: 用4位数表示年，按照实际的月份填写月。例：1月写作“1”，而不是“01”,12月写作“12”。如果记不住月份，请填入’0’。]

**Skip toBF008 请跳至BF008**

**BF006.** When did you divorce? 你们什么时候离婚的？______1900..2011 (BF006_1)year 年 ______0..12 (BF006_2)month 月[hard check: enforce year>year married (BE004 if _BE003=1. BE009 if BE003 >1)]

[IWER: Mark the year using four digits. Take down the month as its actual number. For example, write January as “1” not “01”, December as “12”. If do not remember month, fill ‘0’. 访员注意: 用4位数表示年，按照实际的月份填写月。例：1月写作“1”，而不是“01”,12月写作“12”。如果记不住月份，请填入’0’。]

**Skip toBF008 请跳至BF008**

**BF007.** When did your spouse pass away? 您配偶是什么时候去世的?

_______1900..2011 (BF007_1)year 年______0..12 (BF007_2)month 月

[Hard check: enforce year>year married (BE004 if BE003 =1. BE009 if BE003 >1)]

[IWER: Mark the year using four digits. Take down the month as its actual number. For example, write January as “1” not “01”, December as “12”. If do not remember month, fill ‘0’. 访员注意: 用4位数表示年，按照实际的月份填写月。例：1月写作“1”，而不是“01”,12月写作“12”。如果记不住月份，请填入’0’。]

**BF008.** How often did the respondent receive assistance in answering section A-Demographics? 受访者填写该部分问卷时是否求助？

[IWER: If it is answered by a proxy, please record the respondent’s reaction. 访员注意：如果是协助回答，请记录受访者的反应。]

1. Never 从未
2. A few times 偶尔几次
3. Most or all of the time 大多数
4. The section was completed by a proxy respondent（the respondent is absent） 受访者不在场，完全请人代填 →Skip to BF009 请跳至BF009

**BF009.** [IWER: What is the proxy’s relationship to R? If unknown, please ask the proxy. 访员注意：代填问卷的人和受访者是什么关系。如果不清楚，请问代填者。]

What is your relationship to R? 您和受访者是什么关系？

1. Spouse 配偶
2. Mother 母亲
3. Father 父亲
4. Mother-in-law 岳母/婆婆
5. Father-in-law 岳父/公公
6. Sibling 兄弟姐妹
7. Brother-in-law, sister-in-law 姐夫妹夫/嫂子弟媳
8. Child 孩子
9. Spouse of child 孩子的配偶
10. Grandchild 孙子女
11. Other relative 其他亲戚
12. Helper or other non-relative 帮忙的人或者其他非亲属

**BF010.** [IWER: Please record the reason for proxy 访员注意：请记录代答的原因。]

What is the main reason for proxy（the respondent is absent）？受访者不在场，完全请人代填的主要原因是什么？

（1）The respondent has serious physical handicaps 受访者有严重身体障碍

（2）The respondent has serious mental handicaps 受访者有严重精神障碍

（3）The respondent has rejected this interview. 受访者拒访

（4）Other (BF010_1)其他

# **C FAMILY 家庭**

[IWER: Please conduct this part of the interview (B Family) when the family respondent is at home. Don’t allow a proxy to complete the entire section. 访员注意：当家庭受访者在场时，才提问B家庭部分的问卷。这部分不允许请人完全代填。]

## C1 PARENT CHILDREARING AND SIBLING INFORMATION 父母、子女、兄弟姐妹信息

**In the following three parts: bb parent information, bc childrering information and bd sibling information, I’d like to ask you some questions about your family. 接下来的三部分：父母、子女、兄弟姐妹信息，我们想问您一些有关您这些家人的问题。**

## CA PARENT INFORMATION 父母信息

First, I’d like to ask you some questions about your parents. 首先我们想问您一些关于您父母的问题

**[PROCEDURE: If respondent’s father is a household member, skip to CA003. 程序：如果父亲是家户成员跳至CA003 ]**

**CA001.** Is your father still living? 您父亲还健在吗？

(1) Yes 是

(2) No   否 →Skip to CA003 请跳至 CA003

[IWER: If the respondent reports more than one father, ask about the father who raised the respondent. 访员注意：如果受访者报告有多个父亲，询问抚养受访者的那个。]

**CA002.** What is his name? 您父亲叫什么名字？ __________

[IWER: Mark 'father' if respondent doesn't want to give the name. 访员注意：如果受访者不愿给出，请填写“父亲”。]

*****[[3]](#footnote-4)CA003.** Is your father your….您父亲是你的亲生父亲、继父、养父或者其他…?

(1) Biological father亲生父亲

(2) Adoptive father养父

(3) Stepfather继父

(4) A different biological relative who raised you抚养你长大的有血缘的亲戚

(5) Another individual who raised you其他抚养过您的人

**[PROCEDURE: If CA001=1 or is a household member (means living, but was not asked/interviewed CA001), then ask CA004, CA005 ; otherwise, skip them.][程序：如果CA001 =1或者是家户成员，询问CA004 , CA005 ；否则跳过它们]**

*********CA004.** Where was your father born? 您父亲是在哪里出生的？

(1) This village/neighborhood 本村/社区

(2) Another village/neighborhood in this county/city 本县/市的其他村/社区

(3) Another county/city in this province在本省的其他县/市，_______(CA004_1)city市________(CA004_2) county县

(4)Another province 外省：__(CA004_3)province 省/自治区___ (CA004_4)city市____(CA004_5)county县

00000000000000000000000000000000000000000000000000000000000000000000000000000000000000000000000000000000000000000000000000000000 136___别是？0000000000000000000000000000000000000000000000000000000000000000000000000000000000000000000000000000000000000000000000[IWER: Choose from the list of provinces，see appendix 2 请选择省份，见附表二]

1. Abroad 国外

*****CA005.**Did your father grow up in an urban area or a rural area? 您父亲是在城镇还是农村长大的？

(1) City 城镇

(2) Village 农村

**[PROCEDURE: If respondent’s father is a household member, skip to CA012 . 如果父亲是家户成员跳至CA012 ]**

**[PROCEDURE: If CA001 =1 ask CA006 ; otherwise, skip it] [程序：如果CA001 =1，询问CA006；否则跳过]**

**[Show Card 出示卡片2]**

*****CA006.** What is your father’s Chinese Zodiac sign? 您父亲的属相是__________

[IWER: Choose from the list of Chinese Zodiac signs，see Appendix 1访员注意: 请选择属相，选项见附表一]

*****CA007.** In what year was your father born? 您父亲是哪一年出生的？_______ 1850..1950Year 年

[IWER: Mark the year using four digits. 访员注意: 用4位数表示年。]

[IWER: Fill in -9999 if the respondent cannot recall the date. 访员注意:记不清日期用-9999表示。]

**[PROCEDURE: If CA001 =2, ask CA008 ; otherwise, skip them] [程序: 如果CA001 =2，询问CA008 ; 否则跳过它们]**

*****CA008.** In what year did your father pass away?  您父亲哪年去世的？________1900..2011 (CA008_1 )Year 年 or______1…120 (CA008_2 )Years old岁

[IWER: Mark the year using four digits. 访员注意: 用4位数表示年。]

[IWER: Fill in -9999 if the respondent cannot recall the date. 访员注意:记不清日期用-9999表示。]

**[Softcheck if the number entered for the year of death is smaller than the number entered for the year of birth. 如果CA008 中的年份小于CA007 中的年份，则进行检查。]**

*****CA009.** What is the highest level of education your father has completed? 您父亲的最高学历是？

(1) No formal education (illiterate)  未受过正规教育（文盲）

(2) Did not finish primary school but capable of reading or writing 未读完小学，但能够读、写

(3) Sishu/home school 私塾毕业

(4) Elementary school 小学毕业

(5) Middle school 初中毕业

(6) High school  高中毕业

(7) Vocational school 中专（包括中等师范、职高）毕业

(8) Two-/Three-Year College / Associate degree 大专毕业

(9) Four-Year College / Bachelor’s degree 本科毕业

(10) Post-graduate, Master’s degree 硕士毕业

(11) Post-graduate, Doctoral degree/Ph.D. 博士毕业

**[PROCEDURE: If CA001 =1 ask CA010 ; otherwise, skip it] [程序：如果CA001 =1，询问CA010；否则跳过]**

*********CA010. What is your father’s current marital status?** 您父亲目前的婚姻状态？[**Notice： Common-law marriage is considered as married. 注意：即使没有领证但自称结婚者也可视为已婚。]**

1. Married， with my mother 已婚，配偶是我母亲
2. Married，but not with my mother已婚，但配偶不是我母亲

(3) Separated 分居 （不再作为配偶共同生活）

(4) Divorced 离异

(5) Widowed 丧偶

(6) Never married 从未结婚

**[PROCEDURE: If CA010 = 3, 4, 5, 6 and >=45years old, please ask CA011 ]**

**[程序：如果CA010 =3,4,5,6并且年龄大于等于45岁，请提问CA011 ]**

**[Intro: It is common that many people who are not currently married often live with partners. Please bear me a question on this. The answer to this question will be kept strictly confidential and will be used for researchpurposes only. 现在有很多人虽然没结婚，但与别人同居。所以，想请您回答下面这个问题。请放心，我们将会对您的回答严格保密，并且仅用于学术研究]**

**CA011.** Is the householdmember unmarried but Living with a partner? 您父亲现在有没有和别人同居？

(1) Yes 有

(2) No 没有

[PROCEDURE: If CA001 =2, skip to procedure before CB001 ] [程序: 如果CA001=2, 跳至procedure before CB001 ]

*****CA012.** Does your father work currently (work includes agricultural work, earning wage work, self-employed activities, and unpaid family business work, et. al.)? 请问您父亲现在工作吗 (工作包括务农、挣工资工作、从事个体、私营活动或不拿工资为家庭经营活动帮工等)

1. Yes 在工作
2. No 没工作

*****CA013.** How is your father’s health? Very good, good, fair, poor or very poor? 请问您父亲身体情况怎么样？很好，好，一般，不好还是很不好？

1. Very good 很好
2. Good 好
3. Fair 一般
4. Poor 不好
5. Very poor 很不好

*****CA014.** Which is/was the highest occupation of your father? 您父亲的最高职业是什么？

(1) Managers 国家机关、党群组织、企业、事业单位负责人

(2) Professionals and technicians 专业技术人员

(3) Clerks 办事人员和有关人员

(4) Commercial and service workers 商业、服务业人员

(5) Agricultural, forestry, husbandry and fishery producers 农、林、牧、渔、水利业生产人员

(6) Production and transportation workers 生产、运输设备操作人员及有关人员

(7)Can’t be specified 无法分类的工作

[PROCEDURE: If CA012 =2, skip CA015 ] [程序: 如果CA012 =2, 跳过CA015 ]

*****CA015 .**What is your father’s average income at present? 目前您父亲的收入有多少？（不包括子女给的钱）

____yuan / year or ____yuan/month ____(CA015_1 )元/年或___(CA015_2 )元/月

[PROCEDURE: If respondent’s father is a household member, skip to procedure before CB001. 程序：如果父亲是家户成员，跳至CB001 前的程序控制。]

**[Show Card 出示卡片4]**

*****CA016.** Where does your father normally live? 您父亲一般住在哪里？

1. The same or an adjacent dwelling/courtyard with me 与我同一个院子（公寓）或者相邻的院子（公寓）
2. Another household in this village/neighborhood 本村/社区的其他房子里
3. Another village/neighborhood in this county/city, how far away ____km 本县/市的其他村/社区，离这里有多远: ______(CA016_1)公里
4. Another county/city in this province在本省的其他县/市，_____(CA016_2)city市______(CA016_3) county县, 离这里有多远____________(CA016_4 )公里
5. Another province 外省：_____(CA016_5)province 省/自治区/直辖市，__________(CA016_6)city市_________(CA016_7)county县00000000000000000000000000000000000000000000000000000000000000000000000000000000000000000000000000000000000000000000000000000000 136___别是？0000000000000000000000000000000000000000000000000000000000000000000000000000000000000000000000000000000000000000000000, 离这里有多远___________(CA016_8 )公里

[IWER: Choose from the list of provinces，see appendix 2 请选择省份，见附表二]

1. Abroad 国外

[PROCEDURE: If CA016 =3-5, ask CA01 . 程序：如果CA016 =3-5，询问CA017 ]

*****CA017.** What kind of location does your father live in? 您父亲现在居住地的类型是？

(1) City 城市

(2) County 县城

(3) Town 乡镇

(4) Village 农村

*****CA018.** Is his hukou in the same place as his current residence? 他的户口是在他现在住的地方吗？

(1) Yes 是 Skip to CA020 跳至CA020

(2) No 否

(3) Does not have Hukou 没有户口 Skip to CA021 跳至CA021

*****CA019.** What is the location of your father’s current hukou? 您父亲目前的户口所在地是？

(1) This village/neighborhood 本村/社区

(2) Another village/neighborhood in this county/city 本县/市的其他村/社区

(3) Another county/city in this province在本省的其他县/市，_________(CA019_1)city市_______(CA019_2) county县

(4) Another province 外省：_______(CA019_3)province 省/自治区/直辖市，________(CA019_4)city市__________(CA019_5) county县00000000000000000000000000000000000000000000000000000000000000000000000000000000000000000000000000000000000000000000000000000000 136___别是？0000000000000000000000000000000000000000000000000000000000000000000000000000000000000000000000000000000000000000000000

(5) Abroad 国外

[IWER: This village/neighborhood, this county/city and this province each means the respondent’s one访员注意:本村/社区、本县/市、本省指受访者所在的村/社区、县/市、省]

*****CA020.** What is your father’s current hukou status? 你父亲目前的户口状态是？

(1) Agriculture Hukou 农业户口

(2) Non-Agriculture Hukou 非农业户口

(3) Unified Residency Hukou 统一居民户口

（4）Do not have Hukou 没有户口

F1：“统一居民户口”指的是某些地方实行户口制度改革后，不再区分农业与非农业户口，而是统一为“居民户口”。

*****CA021.** Does your father own a house? 您父亲有房产吗？

(1) Yes 有

(2) No 没有 skip CA022 and CA022_a 跳过CA022和CA022_a

*****CA022.** Do you know the present value of your father’s house? 您父亲的房产现在值多少钱？

_______________10000Yuan 万元

*****CA022_a.** Does your father shares the house with others? 您父亲的房产是与其他人共有的吗？

(1) Yes 是

(2) No 不是

**[PROCEDURE: Add one more section for respondent’s mother, with questions identical to those asked about the respondent’s father. 程序：询问受访者有关其母亲的问题，问题与刚才问到的父亲问题相同。]**

**[PROCEDURE: Add two more sections for respondent’s father-in-law and mother-in-law, with questions identical to those asked about the respondent’s father. 程序：询问受访者有关其岳父/公公和岳母/婆婆的问题，问题与刚才问到的父亲和母亲的问题相同。]**

**[PROCEDURE: if married(B047 =1/2) ask CA023 first:] [程序: 如果受访者已婚(B047 =1/2)，首先询问CA023：]**

**If the spouse is at home, let him/her answer the questions about his/her parents. 如果配偶在，请配偶回答他/她父母的问题。**

**CA023 .** Who answers the questions about the spouse’s parents? 配偶父母的问题由谁回答？

[IWER: Record 访问员记录：]

- 1. The family respondent 家庭模块回答人
  2. The spouse 配偶

**[PROCEDURE: if widow (CV033 = 5), ask CA024] [程序: 如果受访者丧偶(CV033= 5), 询问CA024]**

**CA024.** Do you keep in contact with your spouse’s parents? 您还和您岳父母/公公、婆婆保持联系吗？

1. Yes 是
2. No 否

**[PROCEDURE: If married (B047 =1/2) OR widowed but keeping in contact with spouse’s parents (CA024 =1), then ask information about spouse’s parents] [程序: 如果受访者已婚 (CV033 =1/2)，或者丧偶但是和岳父母/公公、婆婆保持联系 (CA024 =1)，询问受访者岳父母/公公、婆婆的信息]**

## CB CHILDREARING INFORMATION 子女信息

**INTRODUCTION: Next, we will ask some questions about your fostering of your children/about raising your children. 下面，我将询问一些关于您抚养您子女的问题。**

**[PROCEDURE: if the respondent is currently unmarried (BE001= 3 - 6), ask CB001-CB016] [程序: 如果受访者现在婚姻状态不是已婚 (BE001 = 3 - 6), 询问CB001 -CB016]**

**CB001**. Have you ever given birth to any child? If yes, how many are currently living, who are not living with you? 您生过孩子吗？如果生过，没和您住在一起的总共有多少个健在？_______ 0..25人

[IWER: Non-HHmember child.Mark “0” if none 访员注意：这里询问非家户成员子女，如果没有，请填0。]

**[CAPI lists the names of HHmember children.] [CAPI提取属于家户成员子女的姓名]**

**[PROCEDURE: If not mark “0”, ask CB002][程序：如果填的不是0，询问CB002 题]**

**CB002.** What are the name of your biological children who are not living with you？没和您住在一起的亲生子女的姓名是？

[IWER: List names of those children 访员注意：列出此类亲生子女姓名。]

**CB003.** How many biological children do you have who have passed away? 您的亲生子女中，有去世的吗？如果有，多少个去世了？_______ 0..25Persons 人 [IWER: Mark “0” if none.访员注意：如果没有请填0。]

**[PROCEDURE: Repeat questions CB004 ~CB008 for each child who have passed away. 程序：请针对每个去世的孩子询问CB004 ~CB008 ]**

******CB004.**  When was this child born该孩子的出生日期是？______(CB004_1)1900..2011Year年 _____(CB004_2)0..12Month月

[Procedure: If CB004 =DK or RF, skip CB005 . 如果CB004 =不知道或拒绝回答，跳过CB005]

******CB005.** Is your answer to CB020 based on the solar or lunar calendar? 您刚才回答的是公历（阳历）还是农历（阴历）？

（1）Solar calendar公历（阳历）

（2）Lunar calendar农历（阴历）

******CB006.** Sex of this child该孩子的性别是？

     （1）Male男

（2）Female女

******CB007.** When did this child pass away该孩子去世的日期是？_______(CB007_1)1900..2011Year年 ______(CB007_2)0..12Month月

[Procedure: If CB007 =DK or RF, skip CB008 . 如果CB007 不知道或拒绝回答，跳过CB008 ]

**CB008.** Is your answer to BC000_1_3 based on the solar or lunar calendar? 您刚才回答的是公历（阳历）还是农历（阴历）？

（1）Solar calendar公历（阳历）

（2）Lunar calendar农历（阴历**）**

**[Softcheck if the number entered for the year of death is smaller than the number entered for the year of birth. 如果CB004 中的年份小于CB007 中的年份，则进行检查。]**

**CB009.** Have you ever adopted or fostered any child or step child? If yes, how many are currently living who’re not living with you? 您收养或者抚养过子女或继子女吗？如果有，没和您住在一起的总共有多少个健在？_______ 0..25Persons 人

[IWER: Non-HHmember child.Mark “0” if none 访员注意：这里询问非家户成员子女，如果没有，请填0。]

**[CAPI lists the names of HHmember children.] [CAPI提取属于家户成员子女的姓名]**

**[PROCEDURE: If not mark “0”, ask CB010 ][程序：如果填的不是0，询问CB010 题]**

**CB010.** What are the name of your adopted or fostered or step children who are not living with you？没和您住在一起的， 您收养的或抚养的子女或继子女的姓名是？

[IWER: List names of those children 访员注意：列出此类子女姓名。]

**CB011.** How many adopted or foster children or step children do you have who have passed away? 您收养或抚养的子女或继子女中有去世的吗？如果有，多少个已经去世了？ _______ 0..25Persons 人 [IWER: Mark “0” if none.访员注意：如果没有请填0。]

**[PROCEDURE: Repeat questions CB012 ~CB016 for each child who have passed away. 程序：请针对每个去世的孩子询问CB012 ~CB016]**

******CB012.**  When was this child born该孩子的出生日期是？______(CB012_1)1900..2011Year年 _____(CB012_2) 0..12Month月

[Procedure: If CB012 =DK or RF, skip CB013. 如果CB012 =不知道或拒绝回答，跳过CB013]

******CB013.** Is your answer to BC000_2 based on the solar or lunar calendar? 您刚才回答的是公历（阳历）还是农历（阴历）？

（1）Solar calendar公历（阳历）

（2）Lunar calendar农历（阴历）

******CB014.** Sex of this child该孩子的性别是？

     （1）Male男

（2）Female女

******CB015.** When did this child pass away该孩子去世的日期是？_______(CB015_1)1900..2011Year年 ______(CB015_2)0..12Month月

[Procedure: If CB015 =DK or RF, skip CB016. 如果CB015 不知道或拒绝回答，跳过CB016]

**CB016.** Is your answer to CB015 based on the solar or lunar calendar? 您刚才回答的是公历（阳历）还是农历（阴历）？

（1）Solar calendar公历（阳历）

（2）Lunar calendar农历（阴历**）**

**[Softcheck if the number entered for the year of death is smaller than the number entered for the year of birth. 如果CB012 中的年份小于CB015 中的年份，则进行检查]**

**PROCEDURE: if the respondent is currently married (BE001 = 1/2), ask CB017 -CB043 ][程序: 如果受访者现在婚姻状态是已婚 (BE001 = 1/2), 询问CB017 - CB043 ]**

**CB017.** How many biological children do you and your （current ） spouse have together who are currently living and not living with you? 您跟您（现在的）配偶生过孩子吗？如果有，没和您住在一起的总共有多少个？_______ 0..25Persons 人

[IWER: Non-HHmember child.Mark “0” if none 访员注意：这里询问非家户成员子女，如果没有，请填0。]

**[CAPI lists the names of HHmember children.] [CAPI提取属于家户成员子女的姓名]**

**[PROCEDURE: If not mark “0”, ask CB018 ][程序：如果填的不是0，询问CB018 题]**

**CB018.** What are the names of those children who are not living with you？没和您住在一起的，这些子女的姓名是？

[IWER: List names of all those biological children. 访员注意：列出所有此类子女姓名。]

**CB019.** How many biological children do you and your （current）spouse have together who have passed away? 您跟您（现在的）配偶的亲生子女中，有去世的吗？如果有，多少个已经去世？_______ 0..25Persons 人[IWER: Mark “0” if none.访员注意：如果没有请填0。]

**[PROCEDURE: Repeat questions CB020 ~CB024 for each child who have passed away. 程序：请针对每个去世的孩子询问CB020 ~CB024]**

******CB020.**  When was this child born该孩子的出生日期是？______(CB020_1)1900..2011Year年 _____(CB020_2)0..12Month月

[Procedure: If CB020 =DK or RF, skip CB021. 如果CB020 =不知道或拒绝回答，跳过CB021]

******CB021.** Is your answer to CB020 based on the solar or lunar calendar? 您刚才回答的是公历（阳历）还是农历（阴历）？

（1）Solar calendar公历（阳历）

（2）Lunar calendar农历（阴历）

******CB022.** Sex of this child该孩子的性别是？

     （1）Male男

（2）Female女

******CB023.** When did this child pass away该孩子去世的日期是？_______(CB023_1)1900..2011Year年 ______(CB023_2 )0..12Month月

[Procedure: If CB023 =DK or RF, skip CB024. 如果CB023 不知道或拒绝回答，跳过CB024]

**CB024.** Is your answer to CB023 based on the solar or lunar calendar? 您刚才回答的是公历（阳历）还是农历（阴历）？

（1）Solar calendar公历（阳历）

（2）Lunar calendar农历（阴历**）**

**[Softcheck if the number entered for the year of death is smaller than the number entered for the year of birth. 如果CB020 中的年份小于CB023 中的年份，则进行检查。]**

**[PROCEDURE: If A039 =1, skip to CB033 ][程序：如果A039 =1，跳至CB033 题]**

**CB025.** How many additional biological children do you have who are currently living and not living with you? 跟您（现在的）配偶一起生的子女之外，您自己的还有其他亲生子女吗，如果有，没和您住在一起的总共有多少个健在？ _______ 0..25Persons 人

[IWER: Non-HHmember child.Mark “0” if none 访员注意：这里询问非家户成员子女，如果没有，请填0。]

**[CAPI lists the names of HHmember children.] [CAPI提取属于家户成员子女的姓名]**

**[PROCEDURE: If not mark “0”, ask CB026 ][程序：如果填的不是0，询问CB026 题]**

**CB026.** What are the names of those children who are not living with you？没和您住在一起的，这些子女的姓名是？

[IWER: List names of all those children 访员注意：列出此类子女姓名。]

**CB027.** How many additional biological children do you have who have passed away? 除了跟您（现在的）配偶一起生的子女之外，您自己的亲生子女中，有去世的吗？如果有，多少个去世了？_______ 0..25Persons 人 [IWER: Mark “0” if none.访员注意：如果没有请填0。]

**[PROCEDURE: Repeat questions CB028 ~CB032 for each child who have passed away. 程序：请针对每个去世的孩子询问CB028 ~CB032]**

******CB028.**  When was this child born该孩子的出生日期是？______(CB028_1)1900..2011Year年 _____(CB028_2)0..12Month月

[Procedure: If CB028 =DK or RF, skip CB029. 如果CB028 =不知道或拒绝回答，跳过CB029]

******CB029.** Is your answer to CB028 based on the solar or lunar calendar? 您刚才回答的是公历（阳历）还是农历（阴历）？

（1）Solar calendar公历（阳历）

（2）Lunar calendar农历（阴历）

******CB030.** Sex of this child该孩子的性别是？

     （1）Male男

（2）Female女

******CB031.** When did this child pass away该孩子去世的日期是？_______(CB031_1)1900..2011Year年 ______(CB031_2) 0..12Month月

[Procedure: If CB031 =DK or RF, skip CB032. 如果CB031 不知道或拒绝回答，跳过CB032]

**CB032.** Is your answer to CB031 based on the solar or lunar calendar? 您刚才回答的是公历（阳历）还是农历（阴历）？

（1）Solar calendar公历（阳历）

（2）Lunar calendar农历（阴历**）**

**[Softcheck if the number entered for the year of death is smaller than the number entered for the year of birth. 如果CB028 中的年份小于CB031 中的年份，则进行检查]**

**CB033.** How many additional biological children does your （current ） spouse have who are currently living and not living with you? 跟您一起生的子女之外，您（现在的）配偶还有自己的亲生子女吗，如果有，没和您住在一起的总共有多少个健在？_______ 0..25Persons 人

[IWER: Non-HHmember child.Mark “0” if none 访员注意：这里询问非家户成员子女，如果没有，请填0。]

**[CAPI lists the names of HHmember children.] [CAPI提取属于家户成员子女的姓名]**

**[PROCEDURE: If not mark “0”, ask CB034 ][程序：如果填的不是0，询问CB034 题]**

**CB034.**What are the names of those children who are not living with you？没和您住在一起的，这些子女的姓名是？

[IWER: List names of all those children 访员注意：列出此类子女姓名。]

**CB035.** How many additional biological children does your spouse have who have passed away? 除了您和您（现在的）配偶一起生的子女之外，您配偶的亲生子女中，有去世的吗？如果有，有多少个去世了？_______ 0..25Persons 人

[IWER: Mark “0” if none.访员注意：如果没有请填0。]

**[PROCEDURE: Repeat questions CB036 ~CB040 for each child who have passed away. 程序：请针对每个去世的孩子询问CB036 ~CB040]**

******CB036.**  When was this child born该孩子的出生日期是？______(CB036_1)1900..2011Year年 _____(CB036_2)0..12Month月

[Procedure: If CB036 =DK or RF, skip CB037 . 如果CB036 =不知道或拒绝回答，跳过CB037]

******CB037.** Is your answer to CB036 based on the solar or lunar calendar? 您刚才回答的是公历（阳历）还是农历（阴历）？

（1）Solar calendar公历（阳历）

（2）Lunar calendar农历（阴历）

******CB038.** Sex of this child该孩子的性别是？

     （1）Male男

（2）Female女

******CB039.** When did this child pass away该孩子去世的日期是？_______(CB039_1)1900..2011Year年 ______(CB039_2)0..12Month月

[Procedure: If CB039 =DK or RF, skip CB040. 如果CB007 不知道或拒绝回答，跳过CB008]

**CB040.** Is your answer to CB039 based on the solar or lunar calendar? 您刚才回答的是公历（阳历）还是农历（阴历）？

（1）Solar calendar公历（阳历）

（2）Lunar calendar农历（阴历**）**

**[Softcheck if the number entered for the year of death is smaller than the number entered for the year of birth. 如果CB036 中的年份小于CB039 中的年份，则进行检查。]**

**CB041.** How many adopted or foster children or step children do you or your （current ）spouse have who are currently living and not living with you? 您和您（现在的）配偶有其他收养或者抚养的子女或继子女吗？如果有，没和您住在一起的总共有多少个健在？_______ 0..25Persons 人

[IWER: Non-HHmember child..Mark “0” if none 访员注意：这里询问非家户成员子女，如果没有，请填0。]

**[CAPI lists the names of HHmember children.] [CAPI提取属于家户成员子女的姓名]**

**[PROCEDURE: If not mark “0”, ask CB042 ][程序：如果填的不是0，询问CB042 题]**

**CB042.** What are the names of those children who are not living with you？没和您住在一起的，这些子女的姓名是？

[IWER: List names of those children 访员注意：列出此类子女姓名。]

**CB043.** How many adopted or foster children do you or your （current）spouse have who have passed away? 您或您（现在的）配偶收养或抚养的子女中有去世的吗？如果有，多少个已经去世了？ _______10..25Persons 人 [IWER: Mark “0” if none.访员注意：如果没有请填0。]

**[PROCEDURE: Repeat questions CB044 ~CB048 for each child who have passed away. 程序：请针对每个去世的孩子询问CB044 ~CB048]**

******CB044.**  When was this child born该孩子的出生日期是？______(CB044_1)1900..2011Year年 _____(CB044_2)0..12Month月

[Procedure: If CB044 =DK or RF, skip CB045. 如果CB044 =不知道或拒绝回答，跳过CB045 ]

******CB045.** Is your answer to BC000_2 based on the solar or lunar calendar? 您刚才回答的是公历（阳历）还是农历（阴历）？

（1）Solar calendar公历（阳历）

（2）Lunar calendar农历（阴历）

******CB046.** Sex of this child该孩子的性别是？

     （1）Male男

（2）Female女

******CB047.** When did this child pass away? 该孩子去世的日期是？_______(CB047_1)1900..2011Year年 ______（CB047_2）0..12Month月

[Procedure: If CB047 =DK or RF, skip CB048. 如果CB047 不知道或拒绝回答，跳过CB048 ]

**CB048.** Is your answer to BC000_1_3 based on the solar or lunar calendar? 您刚才回答的是公历（阳历）还是农历（阴历）？

（1）Solar calendar公历（阳历）

（2）Lunar calendar农历（阴历**）**

**[Softcheck if the number entered for the year of death is smaller than the number entered for the year of birth. 如果CB044 中的年份小于CB047 中的年份，则进行检查。]**

**[PROCEDURE: repeat questions CB049 ~CB079 for each living child. 程序：请针对每个健在的孩子询问CB049 ~CB079]**

**[PROCEDURE: If the child is a household member, skip to CB064 ; for non-HHmember child, ask the following question。Ask the HHmember child first. We can get the number of hhmember child from CV. 程序：如果孩子是家户成员，跳至CB064;如果有非家户成员的孩子，请问下面的问题CB049 -CB079 ]**

**[PROCEDURE: Preload all the names of the children from the family roster and confirm with the respondent. 程序：请从家户登记表提取所有孩子的信息，并跟受访者进行确认。]**

**Intro: We have asked some information of HHmember child in A section. Now we will ask the other children who are not living with you now. 引语：我们在A 家户登记表中已经问过家户成员的孩子的部分信息。接下来，我们先问没和您住在一起的孩子的相关信息。**

[IWER: Names of HHmember child have already been preloaded from A in CAPI. We only have to ask the non-hhmember childern’s name. 访员注意：家户成员孩子的姓名在系统中，已经预先存到A家户登记表中，此处只需要询问非家户成员的孩子信息]

******CB049 .** Is [CHILD's NAME] a boy or a girl? [孩子姓名]是男孩还是女孩？

(1) Boy (Son) 男孩（儿子）

(2) Girl (Daughter) 女孩（女儿）

**[Show Card 出示卡片2]**

******CB050.** What is the Chinese Zodiac sign for CHILDn's? [孩子姓名]的属相是____________

[IWER: Choose from the list of Chinese Zodiac signs，see Appendix1. 访员注意：请选择属相，选项见附表一]

******CB051.** Birth month and year for CHILDn's NAME? [孩子姓名]的出生年月？_____1900..2011 (CB051_1 )year 年_____0..12 (CB051_2 )month 月

[IWER: Mark the year using four digits. Take down the month as its actual number. For example, write January as “1” not “01”, December as “12”. If do not remember month, fill ‘0’. 访员注意: 用4位数表示年，按照实际的月份填写月。例：1月写作“1”，而不是“01”,12月写作“12”。如果记不住月份，请填入’0’。]

******CB052.** Is your answer to CB051 based on the solar or lunar calendar? 您刚才回答的是公历（阳历）还是农历（阴历）？

（1）Solar calendar公历（阳历）

（2）Lunar calendar农历（阴历）

**[Show Card 出示卡片4]**

******CB053.** Where does this CHILDn's NAME normally live now? 目前，[孩子姓名]在哪里常住？

1. This household, but economicly independent. 这个家里，但是经济上独立
2. The same or adjacent dwelling/courtyard with me 与我同一个院子（公寓）或者相邻的院子（公寓）
3. Another household in this village/neighborhood 本村/社区的其他房子里
4. Another village/neighborhood in this county/city; distance from here: ____km 本县/市的其他村/社区，离这里有多远: ______(CB053_1)_公里
5. Another county/city in this province在本省的其他县/市，___________（CB053_2）city市______（CB053_3）_____ county县, 离这里有多远____________(CB053_4)公里
6. Another province 外省：_______(CB053_5)province 省/自治区/直辖市，_________(CB053_6)city市_________(CB053_7)county县00000000000000000000000000000000000000000000000000000000000000000000000000000000000000000000000000000000000000000000000000000000 136___别是？0000000000000000000000000000000000000000000000000000000000000000000000000000000000000000000000000000000000000000000000, 离这里有多远________(CB053_8 )公里

[IWER: Choose from the list of provinces，see appendix 2. 访员注意：请选择省份，见附表二]

1. Abroad 国外

******CB054.** In what type of location does CHILDn's NAME live? [孩子姓名]现居住地的类型是？

(1) City 城市

(2) County 县城

(3) Town乡镇

(4) Village 农村

**ou live with [name] for one month or more during ]pouse] What is birth order of child among living children of respondent or s**

******CB055.** What is the current hukou status of CHILDn's NAME? [孩子姓名]]目前的户口状态是？

(1) Agriculture Hukou 农业

(2) Non-Agriculture Hukou 非农业

(3) Unified Residency Hukou 统一居民户口

(4) Does not have Hukou 没有户口 Skip to CB058 跳至CB058

F1：“统一居民户口”指的是某些地方实行户口制度改革后，不再区分农业与非农业户口，而是统一为“居民户口”。

******CB056.** Is CHILDn's NAME’s hukou location the same as his/her place of residence? [孩子姓名]的户口所在地与居住地相同吗?

1. Yes 是 Skip to CB081 跳至CB081
2. No 否

******CB057.** Where is the current hukou place of CHILDn's NAME? [孩子姓名]目前的户口所在地是？

(1) This household 这个家里

(2) This village/neighborhood 本村/社区

(3) Another village/neighborhood in this county/city 本县/市的其他村/社区

(4) Anothercity in this province, specify 本省其他县：_________ (CB057_1)

(5) Another province, province: (preloaded list) 外省（选择省份）_________ (CB057_2)

[IWER: Choose from the list of provinces，see appendix 2 请选择省份，见附表二]

(6) Other(specify) 其他(请注明) (CB057_3)_________

**** **CB081.** Where was the birth place of CHILD's NAME? [孩子姓名]出生地是？

(1) This village/neighborhood 在本村/社区

(2) Another village/neighborhood in this county/city 在本县/市的其他村/社区

(3) Another county/city in this province在本省的其他县/市，___________(CB081_1)city市__________ (CB081_2) county县

(4)Another province 外省：_______( CB081_3)province 省/自治区/直辖市, ____________(CB081_4)city市__________( CB081_5) county县 [IWER: Choose from the list of provinces，see appendix 2 请选择省份，见附表二]

(5) Abroad 国外

******CB082.** Is [name]’s present hukou status and location the same as his/her first Hukou?  [孩子姓名]目前户口与他出生时获得的户口是否一样？

1. Yes 是 →Skip to precedure before CB058 请跳至CB058前的程序控制
2. No 否

****** CB083.** How did [name]’s Hukou status or location change?

[孩子姓名]的户口发生过什么变化？

(1)Both Hukou status and location have changed 户口类型和户口所在地都变化过

(2)Only Hukou status has changed 只有户口类型变化

(3)Only Hukou location has changed 只有户口所在地变化 →Skip to CB085 请跳至CB085

****** CB084.** What was [name]’s first HuKou status? [孩子姓名] 第一个户口的类型是？

1. Agricultural Hukou 农业
2. Non-agricultual Hukou 非农业

[If A083=2, skip CB085. 如果A083=2，跳过CB085]

****** CB085.** What was the location of [name]‘s first hukou? [孩子姓名]第一个户口所在地？

(1) This village/neighborhood 在本村/社区

(2) Another village/neighborhood in this county/city 在本县/市的其他村/社区

(3) Another county/city in this province在本省的其他县/市，________ (CB085_1)city市__________ county县(CB085_2)

(4)Another province 外省：_______province 省/自治区/直辖市(CB085_3), _______ity市(CB085_4) ______ county县(CB085_5)00000000000000000000000000000000000000000000000000000000000000000000000000000000000000000000000000000000000000000000000000000000 136___别是？0000000000000000000000000000000000000000000000000000000000000000000000000000000000000000000000000000000000000000000000[IWER: Choose from the list of provinces，see appendix 2 请选择省份，见附表二]

(5) Abroad 国外

**[PROCEDURE: If person less than 6, go to next person 程序：如果这人年龄不到6岁，请询问下一个人]**

******CB058.** Is CHILDn's NAME still in school now? 请问[孩子姓名]现在还在上学吗？

1. Yes 是
2. No 否 Skip to CB060 请跳至CB060

******CB059.** What level of schooling and grade is CHILDn's NAME currently enrolled in? [孩子姓名]现在读几年级？

(1) Primary school grade 1 小学1年级

(2) Primary school grade 2 小学2年级

(3) Primary school grade 3 小学3年级

(4) Primary school grade 4 小学4年级

(5) Primary school grade 5 小学5年级

(6) Primary school grade 6 小学6年级

(7) Middle school grade 1 初中1年级

(8) Middle school grade 2 初中2年级

(9) Middle school grade 3 初中3年级

(10) Middle school grade 4 初中4年级

(11) High school, grade 1 高中1年级

(12) High school, grade 2 高中2年级

(13) High school, grade 3 高中3年级

(14) Vocational/technical high school year 1 中专1年级

(15) Vocational/technical high school year 2 中专2年级

(16) Vocational/technical high school year 3 中专3年级

(17) College year 1 大专/大学1年级

(18) College year 2 大专/大学2年级

(19) College year 3 大专/大学3年级

(20) College year 4 大专/大学4年级

(21) College year 5 大专/大学5年级

(22) College year 6 大专/大学6年及6年级以上

(23) Master’s degree 硕士

(24) Doctoral degree/Ph.D. degree 博士

**Skip to CB063 请跳至CB063**

**[PROCEDURE: If person less than 12, go to next person 程序：如果这人年龄不到12岁，请询问下一个人]**

******CB060.** What is the highest level of education CHILDn's NAME completed? [孩子姓名]的最高学历是？

(1) No formal education (illiterate)  未受过正规教育（文盲） Skip to CB063 . 请跳至CB063 。

(2) Did not finish primary school but capable of reading or writing 未读完小学，但能够读、写 Skip to CB061 请跳至CB061

(3) Sishu/home school 私塾

(4) Elementary school 小学毕业 Skip to CB062 请跳至CB062

(5) Middle school 初中毕业 Skip to CB062 请跳至CB062

(6) High school  高中毕业 Skip to CB062 . 请跳至CB062

(7) Vocational school 中专毕业（包括中等师范、职高） Skip to CB062 请跳至CB062

(8) Two-/Three-Year College / Associate degree 大专毕业 Skip to CB062 请跳至CB062

(9) Four-Year College / Bachelor’s degree 本科毕业 Skip to CB062 请跳至CB062

(10) Post-graduate, Master’s degree 硕士毕业 Skip to CB062 请跳至CB062

(11) Post-graduate, doctoral degree/Ph.D. 博士毕业 Skip to CB063 请跳至CB063

******CB061.** How many years did CHILDn's NAME spend in primary school? [孩子姓名]上过几年小学？ Skip to CB063 请跳至CB063

******CB062.** How many additional years of schooling did CHILDn's NAME receive after [THE ANSWER CHOSEN IN CB060]? [孩子姓名]在[CB060的答案]之后又上过几年级学？

**[PROCEDURE: If person less than 16, go to next person 程序：如果这人年龄不到16岁，请询问下一个人]**

******CB063.** What is CHILDn's NAME marital status? [孩子的姓名]的婚姻状况？

（1）Married with spouse present 已婚并与配偶一同居住

（2）Married but not living with spouse temporarily for reasons such as work 已婚，但因为工作等原因暂时没有跟配偶在一起居住

（3）Separated 分居 （不再作为配偶共同生活）

（4）Divorced 离异

（5）Widowed 丧偶

（6）Never married 从未结婚

**[Procedure: ask CB064 if child has any college education, i.e. CB059 =17-24 or CB060 =8,9,10 or 11 如果孩子接受过大专或以上教育( 即CB059 =17-24或者CB060 =8，9，10或11)请提问CB064 ]**

******CB064.** How much did you and your spouse spend to support this CHILDn's NAME’s college education？ 您和您的配偶花了多少钱供[孩子的姓名]上大学（大专或以上）？_________Yuan 元

**[PROCEDURE: If the person has never married, please skip CB065 -CB068. 程序：如果这人从未结婚，那么跳过CB065 -CB068]**

******CB065.** How many sons does CHILDn's NAME have? [孩子的姓名]有多少个儿子？______ 0..25Persons 个

******CB066.** How many adult sons（above age 16）does CHILDn's NAME have? [孩子的姓名]有多少个已成年(16岁以上) 的儿子？______ 0..25Persons 个

**[Softcheck if the number of sons is smaller than the number of adult sons. 如果CB065 <CB066, 则进行检查]**

******CB067.** How many daughters does CHILDn's NAME have? [孩子的姓名]有多少个女儿？ _ _____ 0..25Persons 个

******CB068.** How many adult daughters（above age 16） does CHILDn's NAME have? [孩子的姓名]有多少个已成年(16岁以上)的女儿？______ 0..25个

**[Softcheck if the number of daughters is smaller than the number of adult daughters. 如果CB067 <CB068, 则进行检查。]**

******CB069.** Which category did the total income of CHILDn's NAME (and his/her spouse) in the past year belong to? [孩子的姓名](和他/她配偶)去年的总收入属于下面哪一类？

1. None 没有收入
2. under 2,000 yuan 少于2千元
3. 2，000 – 5，000 yuan 2千与5千之间
4. 5，000 – 10，000 yuan 5千与1万之间
5. 10,000 - 20,000 yuan 在1万与2万之间
6. 20,000 - 50,000 yuan 在2万与5万之间
7. 50,000 - 100,000 yuan 在5万与10万之间
8. 100,000 - 150,000 yuan 在10万与15万之间
9. 150,000 - 200,000 yuan 在15万与20万之间
10. 200,000 - 300,000 yuan 在20万与30万之间
11. Above 300,000 yuan 高于30万

******CB070.** Is [Child’s Name] working now (work includes agricultural work, earning wage work, self-employed activities, and unpaid family business work, et. al.)? [孩子的姓名]现在工作吗？(工作包括务农、挣工资工作、从事个体、私营活动或不拿工资为家庭经营活动帮工等)

(1) Yes 是

(2) No 否 Skip to CB074 跳至CB074

**[Softcheck if the age of the child is less than 16 and the child is reported to be working. 如果孩子年龄小于16岁，而且CB070 =1，则进行检查。]**

**[Softcheck if the child is reported to be in school and also is reported to be working. 如果这孩子既在上学又在工作，则进行检查。]**

**[Show Card 出示卡片5]**

******CB071.** What is [CHILDn’s NAME]’s main occupation? [孩子的姓名]现在主要干什么工作？

(1) Managers 国家机关、党群组织、企业、事业单位负责人

(2) Professionals and technicians 专业技术人员

(3) Clerks 办事人员和有关人员

(4) Commercial and service workers 商业、服务业人员

(5) Agricultural, forestry, husbandry and fishery producers 农、林、牧、渔、水利业生产人员

(6) Production and transportation workers 生产、运输设备操作人员及有关人员

******CB072.** What is the highest administrative level that CHILDn's NAME has attained? [孩子的姓名]现在有什么行政职务吗？

(1) Team Leader 组长（股长）

(2) Section Chief 科长

(3) Director of a division 处长

(4) Director-General of a bureau and above 局长及以上

(5) Township Leader 乡镇干部

(6) None 无行政职务

F1：行政职务：是国家行政机关为实施行政管理而设置的国家公职。

******CB073.** What is/was your CHILDn's NAME highest professional/technical level? [孩子的姓名]现在有什么专业/技术职称吗？

(1) Technician 技术员

(2) Primary level 初级职称

(3) Intermediate level 中级职称

(4) Advanced level 高级职称

(5) None 无专业/技术职称

F1：“专业/技术职称”指专业技术人员的专业技术与学识水平和工作能力的等级称号。

**[PROCEDURE: IF CB070 =1, skip CB074 , CB075.] [程序: 如果 CB070 =1, 跳过 CB074, CB075.]**

******CB074.** Has [Child’s Name] ever worked before? (Work includes agricultural work, earning wage work, self-employed activities, and unpaid family business work, et. al.) [孩子的姓名]以前工作过吗？ (工作包括务农、挣工资工作、从事个体、私营活动或不拿工资为家庭经营活动帮工等)

(1) Yes 工作过

(2) No 没工作过 Skip to CB076 跳至CB076

**[Show Card 出示卡片5]**

******CB075.** What sort of work did CHILDn's NAME mainly do? [孩子的姓名]主要做什么工作？

(1) Managers 国家机关、党群组织、企业、事业单位负责人

(2) Professionals and technicians 专业技术人员

(3) Clerks办事人员和有关人员

(4) Commercial and service workers商业、服务业人员

(5) Agricultural, forestry, husbandry and fishery producers 农、林、牧、渔、水利业生产人员

(6) Production and transportation workers 生产、运输设备操作人员及有关人员

******CB076.** Did CHILDn's NAME live with others, away from you, before age 16 for more than six months? [孩子的姓名]在16岁之前有没有与您分开、与别人在一起生活过超过6个月？

(1) Yes 是

(2) No 否 →Repeat questions for additional children 返回问另一个子女

******CB077.** What was the earliest age that CHILDn’s NAME lived separately with others for more than six months? [孩子的姓名]最早是在几岁的时候与您分开与别人住六个月以上？_____ 1…16years of age 岁

**[Softcheck if the age reported here is older than the current age of the child. 如果CB077 大于孩子现在的年龄，则进行检查。]**

******CB078.** With whom did CHILDn's NAME live for the longest period of time when not living with you or your spouse? 在不和你们夫妻住在一起的日子里，[孩子的姓名]和谁一起生活的时间最长？

(1) Your parents 你的父母

(2) Your spouse’s parents 你配偶的父母

(3) Your brother’s or sister’s family 你兄弟姐妹的家庭

(4) Other family 其他家庭

(5) Dormitory 学校宿舍

(6) Other 其他

******CB079.** Cumulatively, how long did CHILDn's NAME live separately with others before age 16? [孩子的姓名]16岁以前总共与您分开与别人住六个月以上的时间有多少年？______ 0.00..16.00 years 年_______(CB079_1) 0..11months月

**[PROCEDURE: Repeat questions for additional children 返回问另一个子女]**

**[Intro: Now we will ask information about your grand children. 引语：下面我们想了解一下您孙子女的一些信息。]**

**[Procedure: For all HHMember, if the relationship is grandchild, A006 =9, Loop CB080. 程序：对于家户成员的孙子女(A006 =9)，循环提问CB080]**

**CB080.** Who are [preload grand children who is HHmember]’s parents?(circle all that applies) [CAPI lists the names of grandchildren who are household members.] [孙子]的父母是谁？[CAPI提取家户成员的孙子女的姓名]（可多选）

(1)-(25)[CAPI lists the names of all the children.] [CAPI提取所有孩子的姓名列表]

(26) 其他： (CB080_1)

## CC SIBLINGS 兄弟姐妹

**[INTRO: Next I have some questions about your brothers and sisters. 接下来我们会问一些有关您兄弟姐妹的问题。]**

**CC001.** How many of your siblings are still alive? 您有多少个兄弟姐妹还健在？___ 0..25siblings 个

**[PROCEDURE: If CC001 =0 skip to CC003 . 程序：如果CC001 =0, 跳至CC003 ]**

**CC002.** How many of your living siblings are: 现在活着的兄弟姐妹中，

Older brothers 哥哥有几个______0..25 (CC002_1 ), Younger brothers 弟弟有几个_______ 0..25 (CC002_2 )

Older sisters 姐姐有几个______0..25 (CC002_3 ), Younger sisters 妹妹有几个______0..25 (CC002_4 )

**[Softcheck if the total number of brothers and sisters reported in BD006 is not equal to the total number of siblings reported in CC001 . 所有活着的兄弟姐妹总数(CC002_1 + CC002_2 + CC002_3 +CC002_4 ) ≠CC001 .]**

**CC003.** How many of your biological siblings have died? 您有兄弟姐妹去世了吗?如果有，多少个 _______ 0..25 siblings 个

**[PROCEDURE: If CC003 =0, skip to CC005. 程序：如果CC003=0, 跳至CC005 ]**

*******CC004 .** How many of your deceased siblings were: 您去世的兄弟姐妹中，

Older brothers 哥哥有几个______0..25 (CC004_1 ), Younger brothers 弟弟有几个_______ 0..25 (CC004_2 )

Older sisters 姐姐有几个______0..25 (CC004_3 ), Younger sisters 妹妹有几个______0..25 (CC004_4 )

**[Softcheck if the total number of brothers and sisters reported in CC004 is not equal to the total number of siblings reported in CC003 . 所有去世的兄弟姐妹总数(CC004_1 + CC004_2 + CC004_3 + CC004_4 ) ≠CC003 .]**

**[PROCEDURE: Repeat all sibling questions for the spouse’s siblings. 程序：对配偶的兄弟姐妹重复所有兄弟姐妹问题]**

**If the respondent is separated or divorced, skip the questions about his/her spouse’s siblings. (Note: Even if the respondent is widowed, still ask BD000-1). 如果受访者婚姻状态为分居或者离异，不问他/她配偶兄弟姐妹的问题（注意：即使受访者婚姻状态为丧偶，仍要问他/她配偶兄弟姐妹的问题）**

**If the spouse is at home, let him/her answer the questions about his/her siblings 如果配偶在家请配偶回答他/她兄弟姐妹的问题。**

**CC005.** Who answers the questions about the spouse’s siblings? 配偶兄弟姐妹的问题由谁回答？

[IWER: Record. 访员记录：]

1. The family respondent 家庭模块受访者
2. The spouse 配偶

**CC006.** How many of your spouse’s siblings are still alive? 您配偶有多少个兄弟姐妹还健在？___ 0..25个

**[PROCEDURE:If CC006 =0 skip to CC008. 程序：如果CC006 =0, 跳至CC008 ]**

**CC007.** How many of your spouse’s living siblings are: 在您配偶现在活着的几个兄弟姐妹中，

Older brothers 哥哥有几个______0..25 (CC007_1 ), Younger brothers 弟弟有几个_______0..25 (CC007_2)

Older sisters 姐姐有几个______0..25 (CC007_3), Younger sisters 妹妹有几个______0..25 (CC007_4)

**[Softcheck if the total number of brothers and sisters reported in BD016 is not equal to the total number of siblings reported in BD011. 配偶的所有活着的兄弟姐妹总数(CC007_1 + CC007_2 + CC007_3 + CC007_4 )≠CC006 ]**

**CC008.** How many biological siblings do your spouse or did your spouse have who died? 您配偶有多少个有血缘关系的兄弟姐妹去世了? _______ 0..25个

**[PROCEDURE: If CC008=0, skip to the procedure before CE001. 程序：如果CC008 =0, 跳至CE001 前面的程序控制]**

********CC009.** How many of your spouse’s deceased siblings were: 您配偶去世的兄弟姐妹中，

Older brothers 哥哥有几个______0..25 (CC009_1 ), Younger brothers 弟弟有几个_______ 0..25 (CC009_2)

Older sisters 姐姐有几个______0..25 (CC009_3 ), Younger sisters 妹妹有几个______0..25 (CC009_4 )

**[Softcheck if the total number of brothers and sisters reported in BD012 is not equal to the total number of siblings reported in CC009. 配偶的所有去世的兄弟姐妹总数(CC009_1 + CC009_2 + CC009_3 + CC009_4 )≠CC008 ]**

## C2 TIME TRANSFER AND TRANSFERS 家庭交往与经济帮助

**Introduction: In the following three parts: be time transfer, bf transfer and bg time spent providing care we will ask you how you contact with parents and children, and economic trancfers. 接下来的三部分，我们将询问您与父母、子女间的交往和家庭得到及提供的经济帮助。**

## CD TIME TRANSFER 与父母、子女间的交往

### Contact with parents 与父母的交往

**[PROCEDURE: Skip to CD003 if father/mother/father-in-law/mother-in-law is not alive OR father/mother/father-in-law/mother-in-law is household member. 如果父亲/母亲/岳父(公公)/岳母(婆婆)去世了，或者父亲/母亲/岳父(公公)/岳母(婆婆)是家户成员，跳至CD003 。]**

**[LOOP: Repeat each living and non-resident father/mother/father-in-law/mother-in-law for CD001 -CD002 . CD001-CD002 循环提问健在的没住在一起的父亲/母亲/岳父(公公)/岳母(婆婆)。]**

********[[4]](#footnote-5)CD001.** [If the father/mother/father-in-law/mother-in-law is alive] Whom does your father/mother/father-in-law/mother-in-law live with? [如果父亲/母亲/岳父(公公)/岳母(婆婆)健在] 您父亲/母亲/岳父(公公)/岳母(婆婆)和谁住在一起？

1. By him/herself  自己单独住或者与配偶单独住
2. With my / my spouse’s older brother 和我/配偶哥哥住
3. With my / my spouse’s younger brother 和我/配偶弟弟住
4. With my / my spouse’s older sister 和我/配偶姐姐住
5. With my / my spouse’s younger sister和我/配偶妹妹住
6. Take turns in children’s homes在子女家轮流住
7. Nursing home 住养老院
8. Other 其他

**[Show Card 出示卡片6]**

********CD002.** How often do you/your spouse see your father/mother/father-in-law/mother-in-law? 您或您的配偶多长时间去看望一次您的父亲/母亲/岳父(公公)/岳母(婆婆)？

1. Almost every day差不多每天
2. 2-3 times a week每周2-3次
3. Once a week每周一次
4. Every two weeks每半个月一次
5. Once a month每月一次
6. Once every three months 每三个月一次
7. Once every six months半年一次
8. Once a year每年一次
9. Almost never几乎从来没有
10. Other 其他

**Contact with Children 与子女的交往**

**[ PROCEDURE:If respondent has no non-cohabiting children, skip to CF001. 程序：如果受访者没有不在一起生活的子女，跳至CF001]**

For each of the non-coresident child, ask the following two questions (CD003 and CD004). 对每一个不住在一起的子女，询问以下两个问题（CD003 和CD004）。

**[Show Card 出示卡片6]**

******CD003.** How often do you see CHILDn’s NAME? 您多长时间见到[孩子姓名]?

1. Almost every day差不多每天
2. 2-3 times a week每周2-3次
3. Once a week每周一次
4. Every two weeks每半个月一次
5. Once a month每月一次
6. Once every three months 每三个月一次
7. Once every six months半年一次
8. Once a year每年一次
9. Almost never几乎从来没有
10. Other 其他

**[ PROCEDURE: If CD003=1,2,3, skipCD004. 程序：如果CD003=1,2,3，跳过CD004]**

**[Show Card 出示卡片6]**

******CD004.** How often do you have contact with CHILDn’s NAME either by phone, text mess-age, mail, or email, when you didn’t live with CHILDn’s NAME? 您和[孩子姓名]不在一起住的时候，您多长时间跟[孩子姓名]通过电话、短信、信件或者电子邮件联系?

1. Almost every day差不多每天
2. 2-3 times a week每周2-3次
3. Once a week每周一次
4. Every two weeks每半个月一次
5. Once a month每月一次
6. Once every three months 每三个月一次
7. Once every six months半年一次
8. Once a year每年一次
9. Almost never几乎从来没有
10. Other 其他

## CE TRANSFERS 家庭得到及提供的经济帮助

**Introduction:** **Families sometimes help one another in a variety of ways, and each type of help can be important. The next questions are about help you (and your spouse) have given to or received from your non-coresident family members in the past year. 有时候，家庭之间会有多种形式的互相帮助，而每一种形式的帮助都很重要。所以，接下来我们想了解一下，您和您配偶有没有从其他人那里得到或者给其他人什么经济帮助。**

### Receipt of Economic Assistance including Cash and in-kind Transfers 得到经济帮助（包括现金和实物等）

**[PROCEDURE: Skip to the procedure before CE004 if both of father and mother died before 2010, or both of them are househould member, or one died before 2010, the other is househouldmember, 如果父母双方都在2010年之前去世了，或者父母双方都是家户成员，或者父母一方在2010年以前去世，另一方是家户成员，则跳至CE004 前的程序控制。]**

**CE001.** In the past year, did you or your spouse receive any economics supports from your non-coresident parents? 过去一年，您或您的配偶从您的不住在一起的父母那里收到过任何经济支持吗？

(1) Yes 是

(2) No 否             → Skip to CE004 请跳至CE004

**CE002.** How much did you receive from your non-coresident parents in the past year?( specify the amount of each type of economics transfers). 过去一年，你们从您没住在一起的父母那里获得了多少帮助？（请回答每一类经济帮助的数额）

(1) Regular monetary or in-kind support (e.g., money or in-kind support every month/quarter/half year/year, at fixed time) 定期给钱或实物（比如按月、按季度、按半年、按年给钱或实物，时间上大致固定）

Regular monetary support 定期给钱______ Yuan 元（CE002_1）

Regular in-kind support 定期给实物______ Yuan 元（CE002_2）

(2) Non-regular monetary or in-kind support (e.g., money or in-kind support at Spring Festival or/and Mid-Autumn Festival or/and birthday or/and wedding or/and funeral or/and others)不定期给钱或实物 (比如逢年过节、各种节假日、生日、婚丧事、教育、医疗等情况下不定期给钱或实物)

Non-regular monetary support 不定期给钱______ Yuan 元（CE002_3）

Non-regular in-kind support 不定期给实物______ Yuan 元（CE002_4）

**CE003:** add unfolding brackets （100/200/400/800/1600yuan）for each type of money.请在此处给每种费用添加分级展开问题（100/200/400/800/1600 元）。

[PROCEDURE: Skip to the procedure before CE007 if both of father-in-law and mother-in-law died before 2010, or both of them are househould member, or one died before 2010, the other is househouldmember, 如果岳父母（公公婆婆）双方都在2010年之前去世了，或者岳父母（公公婆婆）双方都是家户成员，或者岳父母（公公婆婆）一方在2010年以前去世，另一方是家户成员，则跳至CE007前的程序控制。]

**CE004 .** In the past year, did you or your spouse receive any economics supports from your non-coresident parents-in-law? 过去一年，您或您的配偶从您的没住在一起的岳父母（公公婆婆）那里收到过任何经济支持吗？

(1) Yes 是

(2) No 否             → Skip to CE007 请跳至CE007

**CE005.** How much did you receive from your non-coresident parents-in-law in the past year?( specify the amount of each type of economics transfers). 你们从您的没住在一起的岳父母（公公婆婆）那里获得了多少帮助？（请回答每一类经济帮助的数额）

(1) Regular monetary or in-kind support (e.g., money or in-kind support every month/quarter/half year/year, at fixed time) 定期给钱或实物（比如按月、按季度、按半年、按年给钱或实物，时间上大致固定）

Regular monetary support 定期给钱______ Yuan 元（CE005_1）

Regular in-kind support 定期给实物______ Yuan 元（CE005_2）

(2) Non-regular monetary or in-kind support (e.g., money or in-kind support at Spring Festival or/and Mid-Autumn Festival or/and birthday or/and wedding or/and funeral or/and others) 不定期给钱或实物 (比如逢年过节、各种节假日、生日、婚丧事、教育、医疗等情况下不定期给钱或实物)

Non-regular monetary support 不定期给钱______ Yuan 元（CE005_3）

Non-regular in-kind support 不定期给实物______ Yuan 元（CE005_4）

**CE006 :** add unfolding brackets （100/200/400/800/1600yuan）for each type of money.请在此处给每种费用添加分级展开问题（100/200/400/800/1600 元）。

[PROCEDURE: Skip to the procedure before CE011 if the respondent and spouse have no non-coresident children.] [程序：如果受访者及配偶没有不住在一起的孩子，则跳至CE011 前的程序控制.]

**CE007.** In the past year, did you or your spouse receive any economics supports from your non-coresident children? 过去一年，您或您的配偶从您的没住在一起的孩子那里收到过任何经济支持吗？

(1) Yes 是

(2) No 否             → Skip to CE011 请跳至CE011

**CE008 .** Which child（ren）?那是哪一个孩子（哪些孩子）？ (choose all that apply可多选)

**[Provide the list of children]**

**For each of the child checked in CE008 , ask CE009 . 对CE008 里选中的每一个孩子，询问CE009 。**

*********CE009.** How much of the following did you receive from this child [CHILDNAME] in the past year?( specify the amount of each type of economics transfers). 过去一年你们从这个孩子【孩子名】那里获得了多少以下各种帮助？（请回答每一类经济帮助的数额）

(1) Regular monetary or in-kind support (e.g., money or in-kind support every month/quarter/half year/year, at fixed time) 定期给钱或实物（比如按月、按季度、按半年、按年给钱或实物，时间上大致固定）

Regular monetary support 定期给钱______ Yuan 元（CE009_1）

Regular in-kind support 定期给实物______ Yuan 元（CE009_2）

(2) Non-regular monetary or in-kind support (e.g., money or in-kind support at Spring Festival or/and Mid-Autumn Festival or/and birthday or/and wedding or/and funeral or/and others) 不定期给钱或实物 (比如逢年过节、各种节假日、生日、婚丧事、教育、医疗等情况下不定期给钱或实物)

Non-regular monetary support 不定期给钱______ Yuan 元（CE009_3）

Non-regular in-kind support 不定期给实物______ Yuan 元（CE009_4）

**CE010** : add unfolding brackets （100/200/400/800/1600yuan）for each type of money.请在此处给每种费用添加分级展开问题（100/200/400/800/1600 元）。

[PROCEDURE: Skip to CE015 if the respondent and spouse have no non-coresident grandchildren or grandchildren are younger than 10 years.] [程序：如果受访者及配偶没有不住在一起的孙子女或孙子女年龄小于10岁，则跳至CE015 .]

**CE011.** In the past year, did you or your spouse receive any economics supports from your non-coresident grandchildren? 过去一年，您或您的配偶从您的没住在一起的孙子女那里收到过任何经济支持吗？

(1) Yes 是

(2) No 否             → Skip to CE015 请跳至CE015

**CE012.** Which child was the parent of the grandchildren? 是哪个孩子的孩子？ (choose all that apply可多选)

**[Provide the list of children]**

**For each of the child checked in CE012 , ask CE013 .对CE012 里选中的每一个孩子，询问CE013 。**

*********CE013.** How much of the following did you receive from this child’s [CHILDNAME] children in the past year? (specify the amount of each type of economics transfers). 过去一年你们从这个孩子【孩子名】的孩子那里获得了多少以下各种帮助？（请回答每一类经济帮助的数额）

(1) Regular monetary or in-kind support (e.g., money or in-kind support every month/quarter/half year/year, at fixed time) 定期给钱或实物（比如按月、按季度、按半年、按年给钱或实物，时间上大致固定）

Regular monetary support 定期给钱______ Yuan 元（CE013_1）

Regular in-kind support 定期给实物______ Yuan 元（CE013_2）

(2) Non-regular monetary or in-kind support (e.g., money or in-kind support at Spring Festival or/and Mid-Autumn Festival or/and birthday or/and wedding or/and funeral or/and others) 不定期给钱或实物 (比如逢年过节、各种节假日、生日、婚丧事、教育、医疗等情况下不定期给钱或实物)

Non-regular monetary support 不定期给钱______ Yuan 元（CE013_3）

Non-regular in-kind support 不定期给实物______ Yuan 元（CE013_4）

**CE014 :** add unfolding brackets （100/200/400/800/1600yuan）for each type of money.请在此处给每种费用添加分级展开问题（100/200/400/800/1600 元）。

**CE015.** In the past year, did you or your spouse receive any economics supports from your non-coresident other relatives? 过去一年，您或您的配偶从您的没住在一起的其他亲戚那里收到过任何经济支持吗？

(1) Yes 是

(2) No 否             → Skip to CE018 请跳至CE018

**CE016.** How much did you receive from your non-coresident other relatives in the past year?( specify the amount of each type of economics transfers). 过去一年，你们从您的没住在一起的其他亲戚那里获得了多少帮助？（请回答每一类经济帮助的数额**）**

(1) Regular monetary or in-kind support (e.g., money or in-kind support every month/quarter/half year/year, at fixed time) 定期给钱或实物（比如按月、按季度、按半年、按年给钱或实物，时间上大致固定）

Regular monetary support 定期给钱______ Yuan 元（CE016_1）

Regular in-kind support 定期给实物______ Yuan 元（CE016_2）

(2) Non-regular monetary or in-kind support (e.g., money or in-kind support at Spring Festival or/and Mid-Autumn Festival or/and birthday or/and wedding or/and funeral or/and others) 不定期给钱或实物 (比如逢年过节、各种节假日、生日、婚丧事、教育、医疗等情况下不定期给钱或实物)

Non-regular monetary support 不定期给钱______ Yuan 元（CE016_3）

Non-regular in-kind support 不定期给实物______ Yuan 元（CE016_4）

**CE017 :** add unfolding brackets （100/200/400/800/1600yuan）for each type of money.请在此处给每种费用添加分级展开问题（100/200/400/800/1600 元）。

**CE018.** In the past year, did you or your spouse receive any economics supports from your non-coresident non-relatives (e.g. friends)? 过去一年，您或您的配偶从您的没住在一起的其他非亲戚(如朋友)那里收到过任何经济支持吗？

(1) Yes 是

(2) No 否             → Skip to CE021 请跳至CE021

**CE019.** How much did you receive from your non-coresident non-relatives in the past year?( specify the amount of each type of economics transfers). 过去一年你们从您的没住在一起的非亲戚那里获得了多少帮助？（请回答每一类经济帮助的数额）

(1) Regular monetary or in-kind support (e.g., money or in-kind support every month/quarter/half year/year, at fixed time) 定期给钱或实物（比如按月、按季度、按半年、按年给钱或实物，时间上大致固定）

Regular monetary support 定期给钱______ Yuan 元（CE019_1）

Regular in-kind support 定期给实物______ Yuan 元（CE019_2）

(2) Non-regular monetary or in-kind support (e.g., money or in-kind support at Spring Festival or/and Mid-Autumn Festival or/and birthday or/and wedding or/and funeral or/and others) 不定期给钱或实物 (比如逢年过节、各种节假日、生日、婚丧事、教育、医疗等情况下不定期给钱或实物)

Non-regular monetary support 不定期给钱______ Yuan 元（CE019_3）

Non-regular in-kind support 不定期给实物______ Yuan 元（CE019_4）

**CE020 :** add unfolding brackets （100/200/400/800/1600yuan）for each type of money.请在此处给每种费用添加分级展开问题（100/200/400/800/1600 元）。

### Provision of Economic Assistance including Cash and in-kind Transfers

### 提供经济帮助（包括现金和实物等）

[PROCEDURE: Skip to the procedure before CE024 if both of father and mother died before 2010, or both of them are househould member, or one died before 2010, the other is househouldmember, 如果父母双方都在2010年之前去世了，或者父母双方都是家户成员，或者父母一方在2010年以前去世，另一方是家户成员，则跳至CE024 前的程序控制。]

**CE021.** In the past year, did you or your spouse provide any economics supports to your non-coresident parents? 过去一年， 您或您的配偶给您的不住在一起的父母过任何经济支持吗？

(1) Yes 是

(2) No 否             → Skip to CE024 请跳至CE024

**CE022.** How much did you provide to your non-coresident parents in the past year?( specify the amount of each type of economics transfers). 你们给您没住在一起的父母多少帮助？（请回答每一类经济帮助的数额）

(1) Regular monetary or in-kind support (e.g., money or in-kind support every month/quarter/half year/year, at fixed time) 定期给钱或实物_（比如按月、按季度、按半年、按年给钱或实物，时间上大致固定）

Regular monetary support 定期给钱______ Yuan 元（CE022_1）

Regular in-kind support 定期给实物______ Yuan 元（CE022_2）

(2) Non-regular monetary or in-kind support (e.g., money or in-kind support at Spring Festival or/and Mid-Autumn Festival or/and birthday or/and wedding or/and funeral or/and others) 不定期给钱或实物 (比如逢年过节、各种节假日、生日、婚丧事、教育、医疗等情况下不定期给钱或实物)

Non-regular monetary support 不定期给钱______ Yuan 元（CE022_3）

Non-regular in-kind support 不定期给实物______ Yuan 元（CE022_4）

**CE023**: add unfolding brackets （100/200/400/800/1600yuan）for each type of money.请在此处给每种费用添加分级展开问题（100/200/400/800/1600 元）。

[PROCEDURE: Skip to the procedure before CE027 if both of father-in-law and mother-in-law died before 2010, or both of them are househould member, or one died before 2010, the other is househouldmember, 如果岳父母（公公婆婆）双方都在2010年之前去世了，或者岳父母（公公婆婆）双方都是家户成员，或者岳父母（公公婆婆）一方在2010年以前去世，另一方是家户成员，则跳至CE027 前的程序控制。]

**CE024.** In the past year, did you or your spouse provide any economics supports to your non-coresident parents-in-law? 过去一年，您或您的配偶给您的没住在一起的岳父母（公公婆婆）过任何经济支持吗？

(1) Yes 是

(2) No 否             → Skip to CE027 请跳至CE027

**CE025.** How much did you provide to your non-coresident parents-in-law in the past year?( specify the amount of each type of economics transfers). 你们给您的没住在一起的岳父母（公公婆婆）多少帮助？（请回答每一类经济帮助的数额）

(1) Regular monetary or in-kind support (e.g., money or in-kind support every month/quarter/half year/year, at fixed time) 定期给钱或实物（比如按月、按季度、按半年、按年给钱或实物，时间上大致固定）

Regular monetary support 定期给钱______ Yuan 元（CE025_1）

Regular in-kind support 定期给实物______ Yuan 元（CE025_2）

(2) Non-regular monetary or in-kind support (e.g., money or in-kind support at Spring Festival or/and Mid-Autumn Festival or/and birthday or/and wedding or/and funeral or/and others) 不定期给钱或实物 (比如逢年过节、各种节假日、生日、婚丧事、教育、医疗等情况下不定期给钱或实物)

Non-regular monetary support 不定期给钱______ Yuan 元（CE025_3）

Non-regular in-kind support 不定期给实物______ Yuan 元（CE025_4）

**CE026 :** add unfolding brackets（100/200/400/800/1600yuan）for each type of money.请在此处给每种费用添加分级展开问题（100/200/400/800/1600 元）。

[PROCEDURE: Skip to the procedure before CE031 if the respondent and spouse have no non-coresident children, 如果受访者及配偶没有不住在一起的孩子，则跳至CE031 前的程序控制。]

**CE027.** In the past year, did you or your spouse provide any economics supports to your non-coresident children? 过去一年，您或您的配偶给您的没住在一起的孩子过任何经济支持吗？

(1) Yes 是

(2) No 否             → Skip to CE031 请跳至CE031

*********CE028.** Which child（ren）?那是哪一个孩子（哪些孩子）？ (choose all that apply可多选)

**[Provide the list of children]**

**For each of the child checked in CE028 , ask CE029 .对CE028 里选中的每一个孩子，询问CE029 。**

*********CE029.** How much of the following did you provide to this child [CHILDNAME] in the past year? (specify the amount of each type of economics transfers). 过去一年你们给[孩子名]多少以下各种帮助？（请回答每一类经济帮助的数额）

(1) Regular monetary or in-kind support (e.g., money or in-kind support every month/quarter/half year/year, at fixed time) 定期给钱或实物（比如按月、按季度、按半年、按年给钱或实物，时间上大致固定）

Regular monetary support 定期给钱______ Yuan 元（CE029_1）

Regular in-kind support 定期给实物______ Yuan 元（CE029_2）

(2) Non-regular monetary or in-kind support (e.g., money or in-kind support at Spring Festival or/and Mid-Autumn Festival or/and birthday or/and wedding or/and funeral or/and others) 不定期给钱或实物 (比如逢年过节、各种节假日、生日、婚丧事、教育、医疗等情况下不定期给钱或实物)

Non-regular monetary support 不定期给钱______ Yuan 元（CE029_3）

Non-regular in-kind support 不定期给实物______ Yuan 元（CE029_4）

**CE030 :** add unfolding brackets （100/200/400/800/1600yuan）for each type of money.请在此处给每种费用添加分级展开问题（100/200/400/800/1600元）。

[PROCEDURE: Skip to CE035 if the respondent and spouse have no non-coresident grandchildren, 如果受访者及配偶没有不住在一起的孙子女，则跳至CE035]

**CE031.** In the past year, did you or your spouse provide any economics supports to your non-coresident grandchildren? 过去一年，您或您的配偶给您的没住在一起的孙子女过任何经济支持吗？

(1) Yes 是

(2) No 否             → Skip to CE035 请跳至CE035

*********CE032.** Which child was the parent of the grandchildren? 是哪个孩子的孩子？ (choose all that apply可多选)

**[Provide the list of children]**

**For each of the child checked in CE032 , ask CE033 .对CE032 里选中的每一个孩子，询问CE033 。**

*********CE033.** How much of the following did you provide to this child’s [CHILDNAME] children in the past year?( specify the amount of each type of economics transfers). 过去一年，你们给这个孩子【孩子名】的孩子多少以下各种帮助？（请回答每一类经济帮助的数额）

(1) Regular monetary or in-kind support (e.g., money or in-kind support every month/quarter/half year/year, at fixed time) 定期给钱或实物（比如按月、按季度、按半年、按年给钱或实物，时间上大致固定）

Regular monetary support 定期给钱______ Yuan 元（CE033_1）

Regular in-kind support 定期给实物______ Yuan 元（CE033_2）

(2) Non-regular monetary or in-kind support (e.g., money or in-kind support at Spring Festival or/and Mid-Autumn Festival or/and birthday or/and wedding or/and funeral or/and others) 不定期给钱或实物 (比如逢年过节、各种节假日、生日、婚丧事、教育、医疗等情况下不定期给钱或实物)

Non-regular monetary support 不定期给钱______ Yuan 元（CE033_3）

Non-regular in-kind support 不定期给实物______ Yuan 元（CE033_4）

CE034 : add unfolding brackets （100/200/400/800/1600yuan）for each type of money.请在此处给每种费用添加分级展开问题（100/200/400/800/1600 元）。

**CE035.** In the past year, did you or your spouse provide any economics supports to your non-coresident other relatives? 过去一年，您或您的配偶给您的没住在一起的其他亲戚过任何经济支持吗？

(1) Yes 是

(2) No 否             → Skip to CE038 请跳至CE038

**CE036.** How much did you provide to your non-coresident other relatives in the past year?( specify the amount of each type of economics transfers). 过去一年你们给您的没住在一起的其他亲戚多少帮助？（请回答每一类经济帮助的数额）

(1) Regular monetary or in-kind support (e.g., money or in-kind support every month/quarter/half year/year, at fixed time) 定期给钱或实物（比如按月、按季度、按半年、按年给钱或实物，时间上大致固定）

Regular monetary support 定期给钱______ Yuan 元（CE036_1）

Regular in-kind support 定期给实物______ Yuan 元（CE036_2）

(2) Non-regular monetary or in-kind support (e.g., money or in-kind support at Spring Festival or/and Mid-Autumn Festival or/and birthday or/and wedding or/and funeral or/and others) 不定期给钱或实物 (比如逢年过节、各种节假日、生日、婚丧事、教育、医疗等情况下不定期给钱或实物)

Non-regular monetary support 不定期给钱______ Yuan 元（CE036_3）

Non-regular in-kind support 不定期给实物______ Yuan 元（CE036_4）

CE037: add unfolding brackets （100/200/400/800/1600yuan）for each type of money.请在此处给每种费用添加分级展开问题（100/200/400/800/1600 元）。

**CE038.** In the past year, did you or your spouse provide any economics supports to your non-coresident non-relatives (e.g. friends)? 过去一年，您或您的配偶给您的没住在一起的其他非亲戚(如朋友)过任何经济支持吗？

(1) Yes 是

(2) No 否             → Skip to CE041 请跳至CE041

**CE039.** How much did you provide to your non-coresident non-relatives in the past year?( specify the amount of each type of economics transfers). 过去一年你们给您的没住在一起的非亲戚多少帮助？（请回答每一类经济帮助的数额）

(1) Regular monetary or in-kind support (e.g., money or in-kind support every month/quarter/half year/year, at fixed time) 定期给钱或实物（比如按月、按季度、按半年、按年给钱或实物，时间上大致固定）

Regular monetary support 定期给钱______ Yuan 元（CE039_1）

Regular in-kind support 定期给实物______ Yuan 元（CE039_2）

(2) Non-regular monetary or in-kind support (e.g., money or in-kind support at Spring Festival or/and Mid-Autumn Festival or/and birthday or/and wedding or/and funeral or/and others) 不定期给钱或实物 (比如逢年过节、各种节假日、生日、婚丧事、教育、医疗等情况下不定期给钱或实物)

Non-regular monetary support 不定期给钱______ Yuan 元（CE039_3）

Non-regular in-kind support 不定期给实物______ Yuan 元（CE039_4）

CE040: add unfolding brackets （100/200/400/800/1600yuan）for each type of money.请在此处给每种费用添加分级展开问题（100/200/400/800/1600 元）。

**CE041.** Have you or your spouse ever given a large amount of money or major assets (worth more than 5000 yuan) to any of your children, not including transfers made for the child’s college education（Not including the large amount of money or major assets (worth more than 5000 yuan) which was mentioned before）. This can include cash, land, housing, or other assets, given as inheritance, at the time of the child’s marriage, to support medical expenses or other emergencies, to support grandchildren’s education or welfare, to avoid taxation, or for any other reason. 除了孩子上大学，您或您的配偶还给过他们一大笔钱或值钱的大物品（超过5000元）吗（不包括前面已回答的超过5000元的大笔财物）？这笔钱或物品包括现金、土地、房屋或其他资产、给他们作为结婚礼物或遗产继承、帮助他们支付医疗费用或其他紧急事件、为了避税或其他原因而帮助他们供孩子（即自己的孙子女/外孙子女）上学、消费。

1. Yes 是
2. No 否 skip to CE046 请跳至 CE046

**CE042 .** To which children did you give the money or assets? [CAPI: preload list of children’s names] 您或您的配偶把这一大笔钱或值钱的大物品给了哪个/些孩子？[CAPI:提取所有孩子的姓名]

[For each selected child, ask:] [分别针对CE042 中每个选中的孩子提问]

*********CE043.** What was the value of the money or assets to [CHILDNAME]? 给[孩子的姓名]的钱或值钱的大物品一共价值多少？_________ yuan 元

*********CE044.** In what year was the (largest) gift made to [CHILDNAME]? 最大的那笔钱或值钱大物品，您或您的配偶是哪年给[孩子的姓名]的？_______ 1900..2011year 年

*******CE045. What was the reason for the gift to [CHILDNAME]? 为什么给[孩子的姓名]？

1. pay for medical care or other emergency expense, 支付医疗费用或其他紧急事件
2. pay for house 买房
3. avoid taxation 避税
4. Inheritance 继承
5. support grandchildren’s education or welfare 供孙子女/外孙子女上学或消费
6. other(explain) ____其他(请注明)________(CE045_1)

**CE046 .**Have you or your spouse ever received a large amount of money or major assets (worth more than 5000 yuan) including cash, land, or property from any of your children（Not including the large amount of money or major assets (worth more than 5000 yuan) which was mentioned before）? This includes paying for medical expenses or other emergecies, paying for housing, or for any other reason. 您或您的配偶从孩子那里收到过一大笔钱或值钱的大物品（超过5000元）吗（不包括前面已回答的超过5000元的大笔财物）？这笔钱或物品包括现金、土地、房屋或其他资产，可以用于医疗或其他紧急事件、买房或其他原因。

1. Yes 是
2. No 否 skip to CE051 请跳至 CE051

**CE047 .**From which children did you receive the money or assets? [CAPI: preload list of children’s names] 您或您的配偶是从哪个/ 哪些孩子这里收到过一大笔钱或值钱的大物品？[CAPI:提取所有孩子的姓名]

[For each selected child, ask:] [分别针对CE047 中每个选中的孩子提问]

***********CE048. What was the value from [CHILDNAME]? [孩子的姓名]给您或您的配偶的钱或值钱的大物品一共价值多少？_________ yuan 元

***********CE049. In what year was the (largest) gift made from [CHILDNAME]? 最大的那笔钱或值钱大物品， [孩子的姓名]是哪年给您或您的配偶？_______ 1900..2011year 年

*******CE050. What was the reason for the gift from [CHILDNAME]? [孩子的姓名]为什么给您或您的配偶?

1. pay for medical care or other emergency expense 支付医疗费用或其他紧急事件
2. pay for house 买房
3. avoid taxation 避税
4. other(explain) ____其他(请注明)________(CE050_1)

*********CE051.** Have you or your spouse ever given a large amount of money or major assets (worth more than 5000 yuan) to your parents（Not including the large amount of money or major assets (worth more than 5000 yuan) which was mentioned before）？This can include cash, land, housing, or other assets. 您或您的配偶还给过父母一大笔钱或值钱的大物品（超过5000元）吗（不包括前面已回答的超过5000元的大笔财物）？这笔钱或物品包括现金、土地、房屋或其他资产.

(1)Yes 是

(2）No 否 skip to CE055 请跳至 CE055

*********CE052.** What was the value of the money or assets to your parents? 给父母的钱或值钱的大物品一共价值多少？_________ yuan 元

*********CE053.** In what year was the (largest) gift made to your parents? 最大的那笔钱或值钱大物品，您或您的配偶是哪年给父母的？_______ 1900..2011year 年

*******CE054. What was the reason for the gift to your parents? 为什么给父母？

1. pay for medical care or other emergency expense, 支付医疗费用或其他紧急事件
2. pay for house 买房
3. avoid taxation 避税
4. Inheritance 继承
5. other(explain) ____其他(请注明)________(CE054_1)

*********CE055.** Have you or your spouse ever received a large amount of money or major assets (worth more than 5000 yuan) to your parents（Not including the large amount of money or major assets (worth more than 5000 yuan) which was mentioned before）？This can include cash, land, housing, or other assets. 您或您的配偶从您父母这里收到过一大笔钱或值钱的大物品（超过5000元）吗（不包括前面已回答的超过5000元的大笔财物）？这笔钱或物品包括现金、土地、房屋或其他资产。

(1)Yes 是

(2）No 否 skip to CE059 请跳至 CE059

*********CE056.** What was the value of the money or assets from your parents? 给您或您的配偶的钱或值钱的大物品一共价值多少？_________ yuan 元

*********CE057.** In what year was the (largest) gift made from your parents? 最大的那笔钱或值钱大物品，是哪年给您或您的配偶的？_______ 1900..2011year 年

*******CE058. What was the reason for the gift from your parents? 为什么给您或您的配偶？

1. pay for medical care or other emergency expense, 支付医疗费用或其他紧急事件
2. pay for house 买房
3. avoid taxation 避税
4. Inheritance 继承
5. other(explain) ____其他(请注明)______

*********CE059 .** Have you or your spouse ever given a large amount of money or major assets (worth more than 5000 yuan) to your parents-in-law（Not including the large amount of money or major assets (worth more than 5000 yuan) which was mentioned before）？This can include cash, land, housing, or other assets. 您或您的配偶还给过您的岳父母/公公婆婆一大笔钱或值钱的大物品（超过5000元）吗（不包括前面已回答的超过5000元的大笔财物）？这笔钱或物品包括现金、土地、房屋或其他资产.

(1) Yes 是

(2）No 否 skip to CE063 请跳至 CE063

*********CE060.** What was the value of the money or assets to your parents-in-law? 给您的岳父母/公公婆婆的钱或值钱的大物品一共价值多少？_________ yuan 元

*******CE061. In what year was the (largest) gift made to your parents-in-law? 最大的那笔钱或值钱大物品，您或您的配偶是哪年给您的岳父母/公公婆婆的？_______ 1900..2011year 年

*******CE062. What was the reason for the gift to your parents-in-law? 为什么给您的岳父母/公公婆婆？

1. pay for medical care or other emergency expense, 支付医疗费用或其他紧急事件
2. pay for house 买房
3. avoid taxation 避税
4. Inheritance 继承
5. other(explain) ____其他(请注明)________ (CE062_1)

*********CE063.** Have you or your spouse ever received a large amount of money or major assets (worth more than 5000 yuan) to your parents-in-law（Not including the large amount of money or major assets (worth more than 5000 yuan) which was mentioned before）？This can include cash, land, housing, or other assets. 您或您的配偶从您的岳父母/公公婆婆这里收到过一大笔钱或值钱的大物品（超过5000元）吗（不包括前面已回答的超过5000元的大笔财物）？这笔钱或物品包括现金、土地、房屋或其他资产。

(1)Yes 是

（2）否 skip to CF001 请跳至 CF001

*********CE064.** What was the value of the money or assets from your parents-in-law? 给您或您的配偶的钱或值钱的大物品一共价值多少？_________ yuan 元

*********CE065.** In what year was the (largest) gift made from your parents-in-law? 最大的那笔钱或值钱大物品，是哪年给您或您的配偶的？_______ 1900..2011year 年

*******CE066. What was the reason for the gift from your parents-in-law? 为什么给您或您的配偶？

1. pay for medical care or other emergency expense, 支付医疗费用或其他紧急事件
2. pay for house 买房
3. avoid taxation 避税
4. Inheritance 继承
5. other(explain) ____其他(请注明)______(CE066_1)

## CF TIME SPENT PROVIDING CARE 提供照料时间

**[PROCEDURE: Based on BC019-BC022, if the respondent has any grandchildren under 16, ask CF001 -CF003 ; otherwise, skip to CF004 . 如果受访者有16岁以下的未成年孙子/孙女，提问CF001 - CF003 ; 否则，请跳至CF004 .]**

**CF001.** Did you spend any time taking care of your grandchildren last year? 过去一年，您或您配偶是否花时间照看了您的孙子/孙女？

(1) Yes 是

(2) No   否                   → Skip to CF004 请跳至CF004

**********[[5]](#footnote-6)CF002.** For which child’s children did you provide care? 您或您配偶照看哪一个子女的孩子？

[IWER: Please list all the children including coresident ones, and add a choice “deceased children” in CAPI list. Select from list displayed by CAPI (child’s name) 访员注意：请列出所有人，包括在一起住的孩子和去世的子女名单，从孩子名单中选出]

**[PROCEDURE: Repeat question CF003 according to the list of names in CF002 . 程序: 根据在CF002 名单重复问CF003]**

**********CF003.** Approximately how many weeks and how many hours per week did you spend last year taking care of this child’s children? 过去一年，您和您配偶大约花几周，每周花多少时间来照看这个子女的孩子？

Myself 我______(CF003_1)weeks 周；__________(CF003_2) 0.00..168.00hours per week 小时/周

My spouse 我爱人____（CF003_3）weeks 周；______（CF003_4） 0.00..168.00hours per week 小时/周

[IWER: Mark '1' if the period is less than 7 days. 访员注意：如果小于7天填写“1”]

**[Softcheck: if more than 52 weeks are reported or more than 140 hours are reported. 如果答案大于52周或者大于140小时。]**

**CF004.** Did you or your spouse take care of your parents or parents-in-law during the last year in assisting them in their daily activities or other activities (e.g., household chores, meal preparation, laundry, going out, grocery shopping, financial management, etc.)? 过去一年，您或您配偶有没有在日常活动（或其他活动）方面给您父母或您配偶的父母提供帮助（例如家务劳动，做饭，洗衣，外出，购物和财务管理）？

(1) Yes 是

(2) No   否                             → Skip to CG001 请跳至CG001

**CF005.** Approximately how many weeks and how many hours per week did you yourself spend last year taking care of your parents or parents-in-law? 过去一年，您自己大约花几周，每周花多少时间来照看您的父母和岳父母（或公公婆婆）？

(1) Your father 您父亲 ____(CF005_1)weeks周; _____(CF005_2)0.00..168.00hours per week小时/周

(2) Your mother 您母亲 ____(CF005_3)weeks周; _____(CF005_4)0.00..168.00hours per week小时/周

（3）Your father-in-law 您的岳父（或公公） ____(CF005_5)weeks周; ___(CF005_6)0.00..168.00hours per week小时/周

（4）Your mother-in-law 您的岳母（或婆婆） ____(CF005_7)weeks周; ___(CF005_8)0.00..168.00hours per week小时/周

**[Softcheck: if (1) is checked in CF004 and 0 or missings are reported in CF005.如果CF004 =1,而且CF005 =0或答案缺失]**

**[Softcheck: if more than 52 weeks are reported or more than 140 hours are reported. 如果答案大于52周或者大于140小时]**

**CF006.** Approximately how many weeks and how many hours per week did your spouse spend last year taking care of your parents or parents-in-law? 过去一年，您的配偶大约花几周，每周花多少时间来照看您的父母和岳父母（或公公婆婆）？

(1) Your father 您父亲 ____(CF006_1)weeks周; _____(CF006_2)0.00..168.00hours per week小时/周

(2) Your mother 您母亲 ____( CF006_3) weeks周; _____(CF006_4)0.00..168.00hours per week小时/周

（3）Your father-in-law 您的岳父（或公公）____(CF006_5)weeks周; ___(CF006_6)0.00..168.00hours per week小时/周

（4）Your mother-in-law 您的岳母（或婆婆 ____(CF006_7)weeks周; ___(CF006_8)0.00..168.00hours per week小时/周

**[Softcheck: if (1) is checked in CF004 and 0 or missings are reported in CF006 .如果CF004 =1,而且CF006 =0或答案缺失。]**

**[Softcheck: if more than 52 weeks are reported or more than 140 hours are reported. 如果答案大于52周或者大于140小时。]**

### CG Living Arrangements preferences 居住安排偏好:

[PROCEDURE: Main respondent and spouse both answers CG001 and CG002] [程序：主要受访者及其配偶都要回答CG001 和CG002 ]

**CG001.** Suppose an elderly person has a spouse and adult children, and has good relationship with them What do you think is the best living arrangement for the elderly person? 假定一个老年人有配偶和成年子女，而且与子女关系融洽，您觉得怎么样的居住安排对他最好?

- 1. Live with adult children 与成年子女一起住
  2. Don’t live with them in the same house, but live in the same community or village. 不与子女一起住，但是跟子女住在同一个村/社区
  3. Don’t live with them in the same house and the same community or village. 不与子女一起住，也不跟子女住在同一个村/社区

(4) Live in a nursing house 住养老院

(5) Other 其他

**CG002.**  Suppose an elderly person has no spouse but has adult children, and has good relationship with them. What do you think is the best living arrangement for him/her? 假定一个老年人没有配偶，但是有成年子女，而且与子女关系融洽，您觉得怎么样的居住安排对他最好?

- 1. Live with adult children 与成年子女一起住
  2. Don’t live with them in the same house, but live in the same community or village. 不与子女一起住，但是跟子女住在同一个村/社区
  3. Don’t live with them in the same house and the same community or village.不与子女一起住，也不跟子女住在同一个村/社区

(4) Live in a nursing house 住养老院

(5) Other 其他

**CG003.** How often did the respondent receive assistance in answering section C -FAMILY? [IWER: If it is answered by a proxy, please record the respondent’s reaction.] 受访者填写C 部分问卷时是否求助？[访员注意：如果是协助回答，请记录受访者的反应。]

(1) Never 从未

(2) A few times 偶尔几次

(3) Most or all of the time 大多数

# **D HEALTH STATUS AND FUNCTIONING 健康状况和功能**

## DA HEALTH STATUS 健康状况

SKIP PATTERN CHECKPOINT: SELF-REPORTED HEALTH STATUS

TWO SCALES ARE USED TO MEASURE SELF-REPORTED HEALTH STATUS. R WILL BE ASKED TO RATE THEIR HEALTH STATUS TWICE, ONCE AT THE BEGINNING OF THIS SECTION AND AGAIN AT THE END OF THE SECTION. QUESTION ORDER WILL BE ASSIGNED RANDOMLY.

IF R IS RANDOMLY ASSIGNED TO ORDER 1(SEC_DA _LIST=1), SKIP TO DA001

IF R IS RANDOMLY ASSIGNED TO ORDER 2(SEC_DA _LIST=2), SKIP TO DA002

跳转检查：自我报告健康状况

在这个部分中，我们将要求受访者完成两次自我健康状况评价。第一次是在这个部分的开头；第二次则放在这个部分的结尾处。在问卷中自我评价的问题以随机的形式出现。

如果受访者被随机分在第一组（DA 部分=1），跳至 DA001

如果受访者被随机分在第二组（DA 部分=2），跳至 DA002

### PART I: GENERAL HEALTH STATUS AND DISEASE HISTORY 第一部分：一般健康状况和疾病史

**DA001.** Next, I have some questions about your health. Would you say your health is excellent, very good, good, fair, or poor? 接下来我们问一些与您健康状况相关的问题。您觉得您的健康状况怎么样？是极好，很好，好，一般，还是不好？

[IWER：Interviewer should read all the following options 访员注意：必须读出所有的选项]

1. Excellent 极好
2. Very good 很好
3. Good 好
4. Fair 一般
5. Poor 不好

**DA002.** Next, I have some questions about your health. Would you say your health is very good, good, fair, poor or very poor? 下面我将问到一些关于您的健康状况的问题。您认为您的健康状况怎样？是很好，好，一般，不好，还是很不好？

[IWER：Interviewer should read all the following options 访员注意：必须读出所有的选项]

1. Very good 很好
2. Good 好
3. Fair 一般
4. Poor 不好
5. Very poor 很不好

[IWER: Please do not ask proxy the following questions from CA005-CA006. 访员注意：DA003 -DA004 题不允许请代理人回答。]

**DA003.**  Do you ever feel pain on the left side of your chest? 您左侧胸部曾感到过疼痛吗？

(1) Yes 是

(2) No 否

**DA004.** Do you ever feel chest pains when climbing stairs/uphill or walking quickly? 当您爬楼梯、登山或快速走的时候，曾感到过胸部疼痛吗？

(1) Yes 是

(2) No 否

(3) Not applicable 不适用

**DA005.** Do you have one of the following disabilities? 您是否有下列残疾问题？

(1) Physical disabilities 躯体残疾

(2) Brain damage/mental retardation 大脑受损/智力缺陷

(3) Vision problem 失明或半失明

(4) Hearing problem 聋或半聋

(5) Speech impediment 哑或严重口吃

**[Skip pattern：If respondent says “no” to all of the above, skip to DA007 跳转检查点：如果没有任何问题，请跳至DA007]**

**DA006.** In what year did you become disabled? 您是什么时候开始患有[preload DA005 answer]？____ 1900..2011Year年

[IWER: Mark the year using four digits. 访员注意：用4位数表示年]

**[Show Card 出示卡片7]**

**DA007.** Have you been diagnosed with [conditions listed below, read one by one] by a doctor? 是否有医生曾经告诉过您有以下这些慢性病？

[IWER: Read one by one. 1=yes, 2=no. 访问员注意：请逐项读出以下答案，并让受访者逐一回答，1=是，

2=否。]

(1) Hypertension 高血压病

(2) Dyslipidemia (elevation of low density lipoprotein, triglycerides (TGs),and total cholesterol, or a low high density lipoprotein level)血脂异常（包括低密度脂蛋白、甘油三酯、总胆固醇的升高或（和）高密度脂蛋白的下降）

(3) Diabetes or high blood sugar糖尿病或血糖升高（包括糖耐量异常和空腹血糖升高）

(4) Cancer or malignant tumor (excluding minor skin cancers) 癌症等恶性肿瘤（不包括轻度皮肤癌）

(5) Chronic lung diseases, such as chronic bronchitis , emphysema ( excluding tumors, or cancer) 慢性肺部疾患如慢性支气管炎或肺气肿、肺心病（不包括肿瘤或癌）

(6) Liver disease (except fatty liver, tumors, and cancer) 肝脏疾病

（除脂肪肝、肿瘤或癌外）

(7) Heart attack, coronary heart disease, angina, congestive heart failure, or other heart problems 心脏病（如心肌梗塞、冠心病、心绞痛、充血性心力衰竭和其他心脏疾病）

(8) Stroke 中风

(9) Kidney disease (except for tumor or cancer) 肾脏疾病（不包括肿瘤或癌）

(10) Stomach or other digestive disease (except for tumor or cancer) 胃部疾病或消化系统疾病（不包括肿瘤或癌）

(11) Emotional, nervous, or psychiatric problems 情感及精神方面问题

(12) Memory-related disease 与记忆相关的疾病 （如老年痴呆症、脑萎缩、帕金森症）

(13) Arthritis or rheumatism 关节炎或风湿病

(14) Asthma 哮喘

**[RPOGRAM：Ask DA008 only if CA010 = 1, 5, 11, and CA010 = no.] [ 程序: 对于每个DA007 的第1,5, 11选项中回答否的选项,问DA008.]**

**DA008.** Do you know if you have [preload the current choice in DA007 ]? 您是否知道自己患有？[依次显示所有DA007 中第1,5, 11选项中回答否的选项的疾病名称]

(1) Yes 是

(2) No 否

**CAPI: If diagnosed or known by R: 如果医生诊断或者自己知道患有：**

**Ask the following 2 questions for each condition (item 1- 14 in DA007 ) he/she named. Do not allow anser to these questions if no disease named in DA007**

**对于他/她患的 DA007 中1-14的每种疾病，重复询问以下两个问题。如果他/她没有DA007 中提到的疾病，那么不需要让他/她回答以下两个问题。**

**DA009.** When was the condition first diagnosed or known by yourself ? 第一次诊断出或者自己知道患有[…]在_____（DA009_1）1900..2011Year年或______（DA009_2） 1…120Age岁？

[IWER: Mark the year using four digits.访员注意: 用4位数记录年份]

**[PROCEDURE: Answer DA010 if DA007 = 2, 5, 6, 7, 9, 10, 12, 13 or DA008=1,5,11.]**

**[程序：如果DA007 =2, 5, 6, 7, 9, 10, 12, 13或者DA008=1,5,11回答DA010 .]**

**DA010.** Are you now taking any of the following treatments to treat […] or its complications (Check all that apply)? Taking Chinese traditional medicine, taking Western modern medicine, other treatments? 您目前有没有正在采用以下方式来治疗[…]及其并发症(选择所有采用的方式)？服用中药，服用西药，吃药以外的其他治疗方法？（可多选）

[IWER: Read one by one. 访问员注意：请逐项读出以下答案，并让受访者逐一回答。]

1. Taking Chinese traditional medicine 服用中药(DA010_1)
2. Taking Western morden medicine 服用西药(DA010_2)
3. Other treatments 吃药以外的其他治疗方法(DA010_3)
4. None of the above 以上都没有(DA010_4)

**[ROCEDURE: If DA007 =1 then answerDA011.] [如果DA007 =1，回答DA011 .]**

**DA011.** Are you now taking any of the following treatments to treat or control your hypertension?(Check all that apply) Taking Chinese traditional medicine, taking Western modern medicine?您目前有没有正在采用以下方式来治疗控制高血压？服用中药，服用西药？（可多选）

[IWER: Read one by one. 1=yes,2=no. 访问员注意：请逐项读出以下答案，并让受访者逐一回答，1=是，

2=否。]

1. Taking Chinese traditional medicine 服用中药
2. Taking Western morden medicine 服用西药
3. None of the above 以上都没有

**DA012.** During last year (last 12 months), how many times have you had blood pressure examination? 在过去一年（12个月）中，您检查过几次血压？

└─┴─┴─┘0...999Times 次

**DA013.** Have your care providers ever given you health education/advice on the following (check all that apply)? Weight control, exercise, diet and/or smoking control? 有没有医生建议您注意以下问题？（可多选）控制体重，身体锻炼，饮食调理，控烟？

[IWER: Read one by one. 1=yes, 2=no. 访问员注意：请逐项读出以下答案，并让受访者逐一回答，1=是，

2=否。]

1. Weight control 控制体重
2. Exercise 身体锻炼
3. Diet 饮食调理
4. Smoking control 控烟
5. None of the above 以上都没有

**[PROCEDURE: IfDA007 =3 then answerDA014. 如果DA007 =3，回答DA014 ]**

**DA014.** Are you now taking any of the folloing treatments to treat or control your diabetes?(Check all that apply) Taking Chinese traditional medicine, taking Western morden medicine ,taking insulin injections?您目前有没有正在采用以下方式来治疗控制糖尿病？服用中药，服用西药，注射胰岛素？（可多选）

[IWER: Read one by one. 1=yes, 2=no. 访问员注意：请逐项读出以下答案，并让受访者逐一回答，1=是，

2=否。]

1. Taking Chinese traditional medicine 服用中药
2. Taking Western modern medicine 服用西药
3. Taking insulin injections 注射胰岛素
4. None of the above 以上都没有

**DA015.** During last year (last 12 months), how many times have you had the following? 过去一年（12个月）中，下列各种检测您分别做了几次？

1. Blood glucose test 血糖检测 (DA015_1)└─┴─┴─┘0..999Times 次
2. Urine glucose test 尿糖检测 (DA015_2)└─┴─┘0..99Times 次
3. fundus examination 眼底检查 (DA015_3 )└─┴─┘0..99 Times 次
4. micro-albuminuria test 微蛋白尿检查 (DA015_4)└─┴─┘0..99 Times 次
5. None of the above 以上都没有

**DA016.** Have your care providers ever given you health education/advice on the following? (check all that apply)有没有医生建议您注意以下问题？控制体重，身体锻炼，饮食调理，控烟，足部自我护理？（可多选）

[IWER: Read one by one. 1=yes, 2=no. 访问员注意：请逐项读出以下答案，并让受访者逐一回答，1=是，

2=否。]

1. Weight control 控制体重
2. Exercise 身体锻炼
3. Diet 饮食调理
4. Smoking control 控烟
5. Foot self-care 足部自我护理
6. None of the above 以上都没有

**[PROCEDURE: If DA007 =4 “Cancer or a malignant tumor, excluding minor skin cancers” then answer DA018.] [程序：如果”DA007 =4癌症或恶性肿瘤（不包括轻度皮肤癌）”，回答DA017 及DA018.]**

**[Show Card 出示卡片8]**

**DA017.** In which organ or part of your body do you have cancer? Including the origins and metastasis of tumor.（circle all that apply）身体的哪个器官或部位患有或曾经患有癌症？包括原发和已转移的肿瘤。（可多选）

[IWER: Read one by one. We should still ask R even if he/she has already been cured. 访员注意：依次读出所有选项，若受访者所患癌症已痊愈，仍需记录。]

- - 1. Brain 大脑
    2. Oral cavity 口腔
    3. Larynx 喉
    4. Other pharynx 咽
    5. Thyroid 甲状腺
    6. Lung 肺
    7. Breast 乳房
    8. Oesophagus 食管
    9. Stomach 胃
    10. Liver 肝脏
    11. Pancreas 胰腺
    12. Kidney 肾脏
    13. Prostate 前列腺
    14. Testicle 睾丸
    15. Ovary 卵巢
    16. Cervix 子宫颈
    17. Endometrium 子宫内膜
    18. Colon or rectum 结肠或直肠
    19. Bladder 膀胱
    20. Skin 皮肤
    21. Non-Hodgkin lymphoma 非何杰金淋巴瘤（非霍奇金淋巴瘤）
    22. Leukemia 白血病
    23. Other organ 其他器官(DA017_1)

**DA018.** Have you taken any of the following treatments to treat your canser or relieve its/their symptoms (e.g., pain, nausea, etc.) in the past two years? (Check all that apply) Taking Chinese traditional medicine ,taking Western morden medicine ,chemotherapy ,surgery ,radiation therapy?在过去的两年中，您有没有采用以下方式来治疗肿瘤或缓解肿瘤所引起的疼痛、恶心等症状(可多选)？服用中药，服用西药，化学疗法，手术治疗，放射疗法？

[IWER: Read one by one. 访问员注意：请逐项读出以下答案，并让受访者逐一回答

。]

1. Taking Chinese traditional medicine 服用中药
2. Taking Western morden medicine 服用西药
3. Chemotherapy 化学疗法
4. Surgery 手术治疗
5. Radiation therapy 放射疗法
6. None of the above 以上都没有

F1：（1）“化学疗法”指用化学合成药物治疗疾病的方法。化学药物治疗(简称化疗)是目前治疗肿瘤及某些身免疫性疾病的主要手段之一。

（2）“手术治疗”是最早应用的治疗癌症的方法，也是目前许多早期癌症治疗的首选疗法。

（3）“放射治疗”是用各种不同能量的射线照射肿瘤，以抑制和杀灭癌细胞的一种治疗方法。

**PROCEDURE: If DA007 =8, then answer DA019. 如果DA007 =8，回答DA019。**

**DA019.**  Are you now taking any of the folloing treatments because of your stroke?(Check all that apply) Taking Chinese traditional medicine ,taking Western morden medicine , physical therapy, acupuncture and moxibustion , occupational therapy?您目前有没有正在采用以下方式来治疗或控制由于中风引起的痉挛或者其它并发症？服用中药，服用西药，物理治疗，针灸治疗，职业治疗？（可多选）

[IWER: Read one by one. 1=yes, 2=no. 访问员注意：请逐项读出以下答案，并让受访者逐一回答，1=是，

2=否。]

1. Taking Chinese traditional medicine 服用中药
2. Taking Western morden medicine 服用西药
3. Physical therapy 物理治疗
4. Acupuncture and moxibustion 针灸治疗
5. Occupational therapy 职业治疗
6. None of the above 以上都没有

F1：（1）“物理治疗”指主要借着自然界中的物理因子（声光水冷电热力）、运用人体生理学原理法则等，针对人体局部或全身性的功能障碍或病变，施予适当的非侵入性、非药物性治疗来处理患者身体不适和病痛治疗方式，使其尽可能地恢复其原有的生理功能。

（2）“针灸治疗”指针法和灸法的合称。针法是把毫针按一定穴位刺入患者体内，运用捻转与提插等针刺手法来治疗疾病。灸法是把燃烧着的艾绒按一定穴位熏灼皮肤，利用热的刺激来治疗疾病。

（3）“职业治疗”指透过悉心选择而适用于病者的康复活动，帮助在精神、情绪、体能或智能上有障碍的人恢复/增强身体及心智功能，阻止/减轻伤残所带来的影向，使他们能适应工作，社会及家庭上的要求，达至正常和独立的生活。

**PROCEDURE: If DA007 =11 or DA008 =11 then answer DA020 如果DA007 =11或DA008=11，回答DA020.**

**DA020.** Are you now taking any of the folloing treatments for your emotional, nervous, or psychiatric problems?(Check all that apply) Receiving psychiatric or psychological treatment, taking anti depressants ,taking tranquilizers or sleeping pills?您目前有没有正在采用以下方式来治疗精神、心理和情感方面的疾病？（可多选）

接受精神科治疗或心理治疗，服用抗抑郁药物，服用镇静或安眠药物？

[IWER: Read one by one. 1=yes, 2=no. 访问员注意：请逐项读出以下答案，并让受访者逐一回答，1=是，

2=否。]

1. Receiving psychiatric or psychological treatment 接受精神科治疗或心理治疗
2. Taking anti depressants 服用抗抑郁药物
3. Taking tranquilizers or sleeping pills 服用镇静或安眠药物
4. None of the above 以上都没有

[IWER: Please do not ask proxy the following questions from DA021 -DA078 . 访员注意：DA021 -DA078 题不允许请代理人回答。]

**DA021.** Have you ever been in a traffic accident or any other kind of major accidental injury and received medical treatment? 您是否经历过交通事故，或任何的重大意外伤害，并接受了治疗？

(1)Yes 是

(2)No 否 → Skip to DA023 跳至DA023

**DA022.** Does your injury caused by the accident limit your daily activities? 事故导致的伤害是否影响您现在的日常活动？

- 1. Yes 是
  2. No 否

**DA023.** Have you fallen down in the last two years? 您过去两年有没有摔倒？

1. Yes 有
2. No 没有 → Skip to DA025 跳至DA025

F1：“过去两年”指从被访日起往前推24个月到受访日这段时间。

**DA024.** How many times have you fallen down in the last two years seriously enough to need medical treatment?? 您过去两年中有多少次摔倒受伤严重到需要接受治疗?_________ times 次

F1：“过去两年”指从被访日起往前推24个月到受访日这段时间。

**DA025.** Have you ever fractured your hip? 您有没有过髋骨骨折？

1. Yes 有
2. No 没有

F1：“髋骨”指人体腰部的骨骼，共左右两块。幼年时，髋骨分为髂骨, 坐骨和耻骨以及软骨连接。成年后，它们之间的软骨会骨化，成为一个整体，即髋骨。

SKIP PATTERN CHECKPOINT: PROSTATE ILLNESS/INCONTINENCE 跳转格式检查: 前列腺疾病/大小便失禁

IF R IS MALE, SKIP TO DA029. 男性跳至DA029

**DA026.**  When did you begin the menarche? 您什么时候开始来月经的？

_____1900..2011 (DA026_1)Year年[IWER: Mark the year using four digits. [访员注意: 用4位数表示年。]

Or 或Age年龄______1…120 (DA026_2) Years岁

**DA027.** Have you started menopause? 您绝经了吗？

1. Yes 是
2. No 否 →Skip to DA032 跳至DA032

**DA028.** When did you begin the menopause? 您什么时候开始绝经的？

_____1900..2011 (DA028_1)Year年；[IWER: Mark the year using four digits. 访员注意: 用4位数表示年。]

Or 或Age年龄______1…120 (DA028_2) Years岁 →Skip to DA032 跳至DA032

**DA029.** Have you ever been diagnosed with a prostate illness, such as prostate hyperplasia（excluding [prostatic](app:ds:prostatic) [cancer](app:ds:cancer)）? 有没有医生诊断您得了前列腺疾病如前列腺增生(排除前列腺癌)？

1. Yes 是
2. No 否 →Skip to DA032 跳至DA032

F1：“前列腺增生”主要症状有排尿困难，轻者夜里起床小便次数增多，有尿不净或尿完后还有少量排出的现象；严重者出现尿流变细，甚或排不出的现象；同时常伴有腰酸腰痛、四肢无力、遗精等症状。

**DA030.** When was the condition first diagnosed? 第一次诊断出您有前列腺疾病是在什么时候？

_____1900..2011 (DA030_1 )Year年；[IWER: Mark the year using four digits. 访员注意: 用4位数表示年。]

Or 或Age年龄______1…120 (DA030_2 ) Years岁

**DA031.** Are you now taking medication or other treatment for your prostate illness? 您目前是否正在服药或以其他方式治疗前列腺疾病？

1. Yes 是
2. No 否

**DA032.** Now I have some questions about your eyesight. Do you usually wear glasses or corrective lenses? 下面问您一些关于视力的问题。您通常是否戴眼镜（包括矫正视力镜片）？

1. Yes 是
2. Legally blind 失明 → Skip to DA038 跳至DA038
3. No 否

**DA033.** How good is your eyesight for seeing things at a distance, like recognizing a friend from across the street (with glasses or corrective lenses if you wear them)? Would you say your eyesight for seeing things at a distance is excellent, very good, good, fair, or poor? 您看远处的东西怎么样？比如说能不能隔着马路认出朋友(包括戴着眼镜)。是极好，很好，好，一般还是不好？

1. Excellent 极好
2. Very good 很好
3. Good 好
4. Fair 一般
5. Poor 不好

**DA034.** How good is your eyesight for seeing things up close, like reading ordinary newspaper print (with glasses or corrective lenses if you wear them)? Would you say your eyesight for seeing things up close is excellent, very good, good, fair, or poor? 您看近处的东西怎么样？比如说戴着眼镜能不能看报纸？是极好，很好，好，一般还是不好？

1. Excellent 极好
2. Very good 很好
3. Good 好
4. Fair 一般
5. Poor 不好

**DA035.** Have you ever had cataract surgery? 您是否做过白内障手术？

1. Yes 是
2. No 否 → Skip to DA037 跳至DA037

**DA036.** Have you had cataract surgery on both eyes or just one? 白内障手术是做的一只眼还是两只眼？

1. One eye only 一只眼
2. Both eyes 两只眼

**DA037.** Has a doctor/nurse/paramedical/ doctor of traditional Chinese medicine doctor ever treated you for glaucoma? 是否有医生诊断过您得了青光眼？

(1)Yes 是

(2)No 否

**DA038.** Now I have some questions about your hearing. Do you ever wear a hearing aid? 现在有几个关于听力的问题。您有没有戴过助听器？

1. Yes 是
2. No 否

**DA039.** Is your hearing very good, good, fair, poor, or very poor (with a hearing aid if you normally use it and without if you normally don’t)? Would you say your hearing is excellent, very good, good, fair, or poor? 您的听力如何？(如果您经常戴助听器，那么戴助听器时听力如何？如果您不经常戴助听器，那么不戴助听器时听力如何？)是极好，很好，好，一般还是不好？

1. Excellent 极好
2. Very good 很好
3. Good 好
4. Fair 一般
5. Poor 不好

**DA040.**  Have you lost all of your teeth? 您的牙齿是否已经掉光？

1. Yes 是
2. No 否

**DA041.** Are you often troubled with any body pains? 您经常为身体疼痛而感到苦恼吗？

(1) Yes 有

(2) No 没有 skip to DA044 请跳至DA044

**[Show Card 出示卡片9]**

**DA042.** On what part of your body do you feel pain? Please list all parts of body you are currently feeling pain. 身体哪些部位感到疼痛？请列出所有部位。

1. Head (Headache) 头
2. Shoulder 肩膀
3. Arm 胳膊
4. Wrist 手腕
5. Fingers 手指
6. Chest 胸
7. Stomach (Stomachache) 胃
8. Back 背
9. Waist 腰
10. Buttocks 臀部
11. Leg 腿
12. Knees 膝盖
13. Ankle 脚踝
14. Toes 脚趾
15. Neck 脖子

**DA043.** How bad is your pain (if more than one type of pain, ask about the most severe one among them)? Mild, Moderate or Severe? 疼痛程度（如果有多于一种类型的疼痛，回答最严重的疼痛程度）轻微，中度，严重？

1. Mild 轻微
2. Moderate 中度
3. Severe 严重

**[Show Card 出示卡片10]**

**DA044.** Have you been diagnosed with any of the following infectious diseases? 是否有医生曾经告诉过您得过以下传染病？

（1）Tuberculosis结核病

（2）hepatitis B 乙型肝炎

（3）Malaria疟疾

（4）Influenza流感

（5）Rabies狂犬病

（6）Schistosomiasis血吸虫病

（7）AIDS patients and infecious ones艾滋病患者及感染者

（8）Encephalitis B 乙型脑炎

（9）Dysentery 痢疾

(10) Measles麻疹

(11) Brucellosis布氏杆菌病

(12) Gonorrhea淋病

(13) Syphilis梅毒

(14) Others其他 Pls specify 请说明(DA044_1)

(15) None 一个都没有

**DA045.** Are there any other medical diseases or conditions that are important to your health now that we have not talked about? 是否还有其他严重的疾病或身体不适我没问到？

1. Yes 是 →Skip toDA046 跳至DA046
2. No 否 →Skip toDA047 跳至DA047

**DA046.**. What illness is that? 这些疾病是什么？_______________________________

**DA047.** Have you gained or lost 5 or more kilograms in the last year? (excluding pregnancy) 过去一年有没有增重10斤以上，或减轻10斤以上？（怀孕除外）

1. Yes, I only gained weight 是，只增重。
2. Yes, I only lost weight 是，只减轻。
3. Yes, I first gained and then lost weight 是，先增重再减轻。
4. Yes, I first lost and then gained weight 是，先减轻再增重。
5. No 没有
6. I don’t know 我不知道

**DA048.** How would you evaluate your health during childhood, up to and including age 15? Excellent, very good, good,fair, poor? 您15岁之前（包括15岁）的身体状况怎么样？极好，很好，好，一般，不好？

(1) Excellent 极好

(2) Very Good 很好

(3) Good 好

(4) Fair 一般

(5) Poor 不好

### PART II: LIFESTYLE AND HEALTH BEHAVIORS 第二部分：生活方式和健康行为

**DA049.** During the past month, how many hours of *actual sleep* did you get at night (average hours for one night)? (This may be shorter than the number of hours you spend in bed.) 过去一个月内，您平均每天晚上真正睡着的时间大约是几小时？（可能短于您在床上躺着的时间）_____0.00..24.00hours小时

**DA050.** During the past month, how long did you take a nap after lunch? 过去一个月内，您通常午睡多长时间？ _____minutes分钟

[ IWER：If R didn’t take a nap,please record for ”0”. 访员注意：如果受访者不午睡，请记录为0.]

**[PROCEDURE: DA051 will be presented ONLY to a random subsample of households (half). Main respondent and spouse in the selected households should answer DA051 ] [程序：仅仅在二次抽样被选中的家庭中询问DA051 题。选中家庭中的主要受访者及其配偶都要回答DA051 题.]**

**DA051.** Now we would like to ask about the amount of time you spend on different types of physical activities in a usual week. 下面有一些问题是有关您通常每周花了多少时间做这些活动？

| **PHYSICAL ACTIVITIES身体活动**  **(KKTYPE)** | | **DA051.** | **DA052.** |  | |
| --- | --- | --- | --- | --- | --- |
|  |  | During **a usual week**, did you do any [….] for at least 10 minutes continuously? 您通常每周有没有至少持续做[...]十分钟？ | During a usual week, on how many days did you do [….] for at least 10 minutes?您通常每周有多少天做[…]至少十分钟？ | How much time did you usually spend doing [….] on one of those days? 在做[…]的这些天里，您一天花多少时间做[…]？ | |
| **A.** | Now, think about all the **vigorous activities** requiring hard/high-intensity physical effort that you do in **a usual week**. Vigorous activities make you breathe much harder than normal and may include heavy lifting, digging, plowing, aerobics, fast bicycling, and cycling with a heavy load. Think only about those physical activities that you did for at least 10 minutes at a time.下面请回忆下您通常每周做的非常消耗体力的激烈活动。激烈的活动会让你呼吸急促，比如搬运重物、挖地、耕作、有氧运动、快速骑车、骑车载货等。只需回忆您每次运动了至少十分钟的活动。 | 3. No否🡻  1. Yes是🡺 | **└──┘**1..7 days天 | **DA053**  1. < 2 hours小时 | **DA054**  1. < 30 minutes分钟  2. ≥ 30 minutes分钟 |
|  |  |  |  | 2. ≥ 2 hours小时 | **DA055**  3. < 4 hours小时  4. ≥ 4 hours小时 |
| **B.** | . Now think about activities which take **moderate physical effort** that you do in a usual week. Moderate physical activities make you breathe somewhat harder than normal and may include carrying light loads, bicycling at a regular pace, or mopping the floor. Again, think about only those physical activities that you did for at least 10 minutes at a time.下面请回忆下您通常每周做的中等强度的体力活动。中等体力的活动让您的呼吸比平时快一些，比如搬运轻便的东西、常规速度骑自行车、拖地、打太极拳、疾走。只要回忆您每次持续了至少十分钟的活动。 | 3. No否🡻  1. Yes是🡺 | **└──┘**1..7days天 | **DA053**  1. < 2 hours小时 | **DA054**  1. < 30 minutes分钟  2. ≥ 30 minutes分钟 |
|  |  |  |  | 2. ≥ 2 hours小时 | **DA055**  3. < 4 hours小时  4. ≥ 4 hours小时 |
| **C.** | Now think about the time you spend **walking** in a usual week. This includes at work and at home, walking to travel from place to place, and any other walking that you might do solely for recreation, sport, exercise, or leisure.下面回忆一下您通常每周走路花了多少时间。走路包括工作或者在家的时候从一个地方走到另一个地方，以及其他您为了休闲、运动、锻炼或娱乐而散步。 | 3. No否🡻  1. Yes是🡺 | **└──┘**1..7 days天 | **DA053**  1. < 2 hours小时 | **DA054**  1. < 30 minutes分钟  2. ≥ 30 minutes分钟 |
|  |  |  |  | 2. ≥ 2 hours小时 | **DA055**  3. < 4 hours小时  4. ≥ 4 hours小时 |

**ACTIVITIES IN LAST MONTH 过去一个月的活动**

**[Show Card 出示卡片11]**

**DA056.** Have you done any of these activities in the last month? (Code all that apply) 您过去一个月是否进行了下列社交活动？(可多选)

(1) Interacted with friends 串门、跟朋友交往

(2) Played Ma-jong, played chess, played cards, or went to community club 打麻将、下棋、打牌、去社区活动室

(3) Provided help to family, friends, or neighbors who do not live with you and who did not pay you for the help 无偿向与您不住在一起的亲人、朋友或者邻居提供帮助

(4) Went to a sport, social, or other kind of club 去公园或者其他场所跳舞、健身、练气功等

(5) Took part in a community-related organization 参加社团组织活动

(6) Done voluntary or charity work 志愿者活动或者慈善活动

(7) Cared for a sick or disabled adult who does not live with you and who did not pay you for the help无偿照顾与您不住在一起的病人或残疾人

(8) Attended an educational or training course 上学或者参加培训课程

(9）Stock investment 炒股（基金及其他金融证券）

（10）Used the Internet 上网

（11）other 其他

(12) None of these 以上均没有

**[CHECK: You cannot select 'None of these' together with any other answer. Please change your answer. 检查点：您不能同时选择“以上答案均不是”和其他选项。请重新选择。]**

***LOOP cnt = 1 to 10* 循环点1－10**

**|*IF cnt IN* DA056 *(ACTIVITIES IN LAST MONTH)* 如果DA056 题答案为1－10（过去一个月的活动）**

**DA057.** Frequency of activity in the last month 过去一个月的活动频率

How often in the last month [did/have][you] [do voluntary or charity work/cared for a sick or disabled adult/provided help to family, friends or neighbors/attended an educational or training course/ Interacted with friends /go to a sport,social or other kind of club/taken part in a community-related organization]? Almost daily, almost every week, or not regularly? 过去一个月，您每隔多长时间会做刚才说的这些活动（志愿者活动或者慈善活动、照顾病人或残疾人、给家里人、朋友或者邻居提供帮助、接受教育或者参加培训课程、与朋友来往、去运动娱乐、社交或者其他类型的俱乐部、参加社团组织活动、炒股、上网）？差不多每天，差不多每周或不经常？

(1) Almost daily 差不多每天

(2) Almost every week 差不多每周

(3) Not regularly 不经常

**DA058.**  How many meals do you normally eat every day? More than 4 meals per day,4 meals per day,3 meals per day ,2 meals per day,1 meal per day or <1 meal per day?您一般每天吃几顿饭？每天多于4顿,每天4顿,每天3顿,每天2顿,每天一顿,每天少于一顿?[IWER:Read each choice of the answers. 访员注意：请读出各选项。]

1. More than 4 meals per day 每天多于4顿
2. 4 meals per day 每天4顿
3. 3 meals per day 每天3顿
4. 2 meals per day 每天2顿
5. 1 meal per day 每天一顿
6. <1 meal per day 每天少于一顿

**INTRO. Next, I would like to ask whether you have had the habit of smoking cigarettes/smoking a pipe/chewing tobacco, now or in the past. By smoking we mean smoking more than 100 cigarettes in your life) 下面我想知道您现在或者过去是否有抽烟、用烟管吸烟或咀嚼烟草的习惯（吸烟指的是一生吸烟100支以上）？**

**DA059.** Have you ever chewed tobacco, smoked a pipe, smoked self-rolled cigarettes, or smoked cigarettes/cigars? 您吸过烟吗？(包括香烟、旱烟、用烟管吸烟或咀嚼烟草)

(1) Yes 是

(2) No 否 Skip to DA067 请跳至DA067

**DA060.** Which products did/do you normally use? 吸烟时，一般抽什么烟？

(1) Smoking a pipe 用烟管吸烟（烟袋、旱烟）

(2) Smoking self-rolled cigarettes 自己卷烟抽

(3) Filtered cigarette带滤咀香烟

(4) Unfiltered cigarette不带滤咀香烟

(5) Cigar雪茄

(6) Water cigarettes 水烟

**DA061.** Do you still have the habit or have you totally quit? 您现在还在吸烟还是戒烟了？

(1) Still have 仍然抽烟 Skip DA062 请跳过DA062

(2) Quit 戒烟

**DA062 .** At what age did you totally quit smoking? 您这次成功戒烟是在多少岁或那一年？

Age年龄______1…120 (DA062_1) years岁 Or 或_____1900..2011 (DA062_2) Year年

[IWER: Mark the year using four digits. 访员注意: 用4位数表示年。]

**If DA060 =3 or 4 askDA063 -DA064. 如果DA060 =3或4，提问DA063 -DA064**

**DA063.** In one day about how many cigarettes do/did you consume [preload:now/before totally quitting]? 您[系统加载：现在/戒烟前]平均一天抽多少支香烟？________ cigarettes 支

**If DA060=3 or 4 ask DA064 . 如果DA060=3或4，提问DA064**

**DA064.** How much does/did it cost per pack = 20 cigarettes? 您[系统加载：现在/戒烟前]前，抽的香烟多少钱一包(一包20支) ？_____Yuan 元

[IWER: Prompt R: we’re asking price at that time, not current price][访员注意：提示受访者，此处询问的是当时价格，非现在的价格]

**DA065.** At what age did you start to smoke on a regular basis? 您是多少岁开始经常抽烟的？

Age年龄______ 1…120 (DA065_1) years岁 Or 或_____1900..2011 (DA065_2) Year年

[IWER: Mark the year using four digits. 访员注意: 用4位数表示年。]

**DA066.** How soon after you wake up did/do you smoke your first cigarette, cigar, or pipe? 您[系统加载：现在/戒烟前]前一般睡醒后多久就开始抽烟？

1. Within 5 minutes 5分钟之内
2. Within 6-30 minutes 6－30分钟之内
3. Within 31-60 minutes 31－60分钟之内
4. More than 1 hour 1个小时以后

**DA067.** Did you drink any alcoholic beverages, such as beer, wine, or liquor in the past year? How often? 在过去的一年，您喝酒吗，包括啤酒、葡萄酒或白酒等？喝酒频率如何？

(1) Drink more than once a month. 喝酒，每月超过一次

(2) Drink but less than once a month 喝酒，但每月少于一次 Skip to DA069 请跳至DA069

(3) None of these 什么都不喝 Skip to DA069 请跳至DA069

**DA068.** What type of alcoholic beverages did you drink? Liquor, wine, or beer？(code all that apply)您平时喝哪种酒？烈性酒、葡萄酒、啤酒还是其他？（可多选）

(1) Liqor, including white liquor, whisky, and others 烈性酒，包括白酒、威士忌和其他酒 Skip to DA071 请跳至DA071

(2) Beer 啤酒 Skip to DA071 请跳至DA071

(3) Wine or rice wine 葡萄酒、米酒或黄酒 Skip to DA071 请跳至DA071

**DA069.**  Did you ever drink alcoholic beverages in the past? How often? 您以前喝酒吗？喝酒频率如何？

1. I never had a drink.我从不或极少喝酒 →Skip to DA079 请跳至DA079
2. I used to drink less than once a month. 我很少喝酒，每月少于一次→Skip to DA079 请跳至DA079
3. I used to drink more than once a month. 我以前喝酒，每月超过一次

**DA070.** When did you quit or reduce drinking? 请问您什么时候戒酒或减少喝酒次数的？

___________1900..2011 (DA070 _1)Year 年 ；

[IWER: Record year in 4 digits. 访员注意：年请用4位数表示]

Or age 或年龄: __________1…120 (DA070_2) Years 岁

**DA071.** When did you start drinking? 请问您什么时候开始饮酒？

___________1900..2011 (DA071_1)Year 年；

[IWER: Record year in 4 digits. 访员注意：年请用4位数表示]

Or age 或年龄: __________1…120 (DA071_2) Years 岁

**SKIP PATTERN CHECKPOINT: DRINKING 跳转格式检查：喝酒**

**Now, I am going to ask you how often and how much you drank during the past year. Please tell me how often you drank per month, and how much you drank at a time on average. I will repeat the questions for different types of alcoholic beverages. 现在我想了解过去一年您喝酒的情况。请回答您平均每个月喝多少次酒，一次大概喝多少。我会针对不同的酒类，分别提问。**

**[CAPI: If DA068 answered 1, ask DA072 :] [程序：如果DA068 选择了1，询问DA072 .]**

**DA072.** How often did you drink liquor, including white liquor, whisky, and others per month in the last year？过去一年内您平均一个月喝几次烈性酒，包括白酒、威士忌？

1. Once a month 每月一次
2. 2-3 times a month 每月2-3次
3. Once a week 每周一次
4. 2-3 times a week 每周2-3次
5. 4-6 times a week 每周4-6次
6. Once a day 每天一次
7. Twice a day 一天两次
8. More than twice a day 一天超过两次

**DA073.** The last time you drank liquor last year, how many liang of liquor did you drink? (1 liang=50cc/50 ml)请问您过去一年内最近一次喝烈性酒，喝了多少两？（1两=50毫升）______ liang 两

**[CAPI: If DA068=2, ask DA074 :] [程序：如果DA068 选择了2，询问DA074 .]**

**DA074.** How many times per month did you drink beer in the last year？过去一年内您平均一个月喝几次啤酒?

1. Once a month 一个月一次
2. 2-3 times a month 一个月2-3次
3. Once a week 每周一次
4. 2-3 times a week 每周2-3次
5. 4-6 times a week 每周4-6次
6. Once a day 每天一次
7. Twice a day 一天两次
8. More than twice a day 一天超过两次

**DA075.** The last time you drank beer last year, how many bottles of beer did you drink? (1bottle=2.5 mugs, 1mug=220cc) 请问您过去一年内最近一次喝啤酒，喝了多少瓶?（1瓶=2.5杯，1杯=220毫升）______ (DA075_1)0..120bottles 瓶 or或者 _______(DA075_2)0..300mugs 杯

**[CAPI: If DA068 answered 3, ask DA076:] [程序：如果DA068 选择了3，询问DA076.]**

**DA076.** How often did you drink wine or rice wine per month in the last year？过去一年内您平均一个月喝几次葡萄酒、米酒或黄酒?

1. Once a month 一个月一次
2. 2-3 times a month 一个月2-3次
3. Once a week 每周一次
4. 2-3 times a week 每周2-3次
5. 4-6 times a week 每周4-6次
6. Once a day 每天一次
7. Twice a day 一天两次
8. More than twice a day 一天超过两次

**DA077.** The last time you drank it last year, how many liang of wine did you drink? (1 liang=50cc) 请问您过去一年内最近一次喝葡萄酒、米酒或黄酒，喝了多少两？（1两=50毫升）________ 0.00..100.00liang 两

**DA078.** In the last year, have you ever taken a drink first thing in the morning to steady your nerves or get rid of a hangover? 过去一年内，请问您是否曾经在早晨做的第一件事就是喝酒，以平缓紧张情绪或为了摆脱宿醉的烦恼？

1. Yes 是
2. No 否

**DA079.** How would you rate your health status? Would you say your health is very good, good, fair, poor or very poor? 您觉得您的健康状况是很好，好，一般，不好，还是很不好？

1. Very good 　很好
2. Good 　　 好
3. Fair 　　　 一般
4. Poor 　　　 不好
5. Very poor 　很不好

**DA080.** Next I have some questions about your health. Would you say your health is excellent, very good, good, fair, or poor? 您觉得您自己的健康状况怎么样？是极好，很好，好，一般还是不好？

[IWER：interviewer should read all the following options. 访员注意：请读出所有的选项。]

1. Excellent 极好
2. Very good 很好
3. Good 好
4. Fair 一般
5. Poor 不好

|  | | INTERVIEWER CHECK AGE OF RESPONDENT?访员请检查受访者的年龄。 | **1、< 65 YEAR岁🡺COLUMN A A栏 6、85– 89YEAR岁🡺 COLUMN F F栏**  **2、65 – 69 YEAR岁🡺COLUMN B B栏 7、90– 94YEAR岁🡺 COLUMN G G 栏**  **3、70 – 74 YEAR岁🡺COLUMN C C栏 9、95– 99YEAR岁🡺 COLUMN H H 栏**  **4、75 – 79 YEAR岁🡺 COLUMN D D栏 10、>= 100 YEAR岁🡺 COLUMN I I 栏**  **5、80– 84YEAR岁🡺 COLUMN E E栏** | | | | | | | | |  |
| --- | --- | --- | --- | --- | --- | --- | --- | --- | --- | --- | --- | --- |
| AGE年龄 | | | A | B | C | D | E | F | G | H | I | |
|  |  |  | 75 years  岁 | 80 years  岁 | 85  years  岁 | 90 years  岁 | 95 years  岁 | 100 years  岁 | 105  years  岁 | 110 years  岁 | 115  years  岁 | |
| DA081 . | Suppose there are 5 steps, where the lowest step represents the smallest chance and the highest step represents the highest chance, on what step do you think is your chance in reaching the age of [...]? 假定有五个级别，最低一级代表可能性最小，最高一级代表可能性最大，您设想您活到这个年龄的可能性有多大。  1 Almost impossible 几乎不可能  2 Not very likely不太可能  3 Maybe有可能  4 Very likely很可能  5 Almost certain 简直一定 | | 1 | 1 | 1 | 1 | 1 | 1 | 1 | 1 | 1 | |
|  |  |  | 2 | 2 | 2 | 2 | 2 | 2 | 2 | 2 | 2 | |
|  |  |  | 3 | 3 | 3 | 3 | 3 | 3 | 3 | 3 | 3 | |
|  |  |  | 4 | 4 | 4 | 4 | 4 | 4 | 4 | 4 | 4 | |
|  |  |  | 5 | 5 | 5 | 5 | 5 | 5 | 5 | 5 | 5 | |

[IWER: Please do not ask proxy the following question DA079 and DA080 . 访员注意：DA079 and DA080 题不允许请代理人回答。]

## DB FUNCTIONAL LIMITATIONS AND HELPERS 身体功能障碍以及辅助者

[CAPI: If R is younger than 50(year of birth is after 1960) and If DA001 =1 or 2 or DA002 =1 or 2 and DA003 =2 and DA004 =2 and DA005 =. and DA007 =2 or DA008 =2 skip DB001 -DB015 ][ 程序：如果受访者年龄小于50岁(出生年份在1960年之后)，并且自我评价健康状况良好、没有残疾、慢性病和身体疼痛，跳过DB001-DB015 ]

**We need to understand difficulties people may have with various activities because of a health or physical problem. Please tell me whether you have difficulty performing any of the following tasks on a regular basis. Exclude any difficulties that you expect to last less than three months.人们可能会由于健康或身体的原因而在多种活动中有困难。请说说您每天做事的时候是否遇到了这些困难（排除那些你预计在三个月内能解决的问题）.每道题目的选项都是一样的，包括没有困难，有困难但仍可以完成，有困难且需要帮助，无法完成。请选择合适的答案。**

**DB001.** Do you have any difficulty with running or jogging about 1 Km? 您现在跑或慢跑1公里，有困难吗？

1. No, I don’t have any difficulty 没有困难 >> Skip to DB004 跳至DB004
2. I have difficulty but can still do it. 有困难但仍可以完成。
3. Yes, I have difficulty and need help. 有困难,需要帮助。
4. I can not do it. 无法完成

**DB002.** Do you have difficulty …Walking 1 km…? 您现在走1公里，有困难吗？

1. No, I don’t have any difficulty 没有困难 >> skip DB003 跳过DB003
2. I have difficulty but can still do it. 有困难但仍可以完成。
3. Yes, I have difficulty and need help. 有困难,需要帮助。
4. I can not do it. 无法完成

**DB003.** Do you have difficulty … Walking 100 metres…? 您走100米，有困难吗？

1. No, I don’t have any difficulty 没有困难
2. I have difficulty but can still do it. 有困难但仍可以完成。
3. Yes, I have difficulty and need help. 有困难,需要帮助。
4. I can not do it. 无法完成

**DB004.** Do you have difficulty …Getting up from a chair after sitting for a long period…? 您在椅子上坐时间久了再站起来，有困难吗？

1. No, I don’t have any difficulty 没有困难
2. I have difficulty but can still do it. 有困难但仍可以完成。
3. Yes, I have difficulty and need help. 有困难,需要帮助。
4. I can not do it. 无法完成

**DB005.** Do you have difficulty …Climbing several flights of stairs without resting…? 您连续不停地爬几层楼，有困难吗？

1. No, I don’t have any difficulty 没有困难
2. I have difficulty but can still do it. 有困难但仍可以完成。
3. Yes, I have difficulty and need help. 有困难,需要帮助。
4. I can not do it. 无法完成

**DB006.** Do you have difficulty …Stooping, kneeling, or crouching…? 弯腰、屈膝或者下蹲，您有困难吗？

1. No, I don’t have any difficulty 没有困难
2. I have difficulty but can still do it. 有困难但仍可以完成。
3. Yes, I have difficulty and need help. 有困难,需要帮助。
4. I can not do it. 无法完成

**DB007.** Do you have difficulty …Reaching or extending your arms above shoulder level…? (he/she is regarded as not having difficulty only if he/she can extend both of his/her arms, otherwise he/she is regarded as having difficulty.) 您把手臂沿着肩向上伸展，有困难吗？（两个手都没困难才算没困难，否则算有困难）

1. No, I don’t have any difficulty 没有困难
2. I have difficulty but can still do it. 有困难但仍可以完成。
3. Yes, I have difficulty and need help. 有困难,需要帮助。
4. I can not do it. 无法完成

**DB008.** Do you have difficulty …Lifting or carrying weights over 10 jin, like a heavy bag of groceries…? 您提10斤重的东西，有困难吗？

1. No, I don’t have any difficulty 没有困难
2. I have difficulty but can still do it. 有困难但仍可以完成。
3. Yes, I have difficulty and need help. 有困难,需要帮助。
4. I can not do it. 无法完成

**DB009.** Do you have difficulty …Picking up a small coin from a table…? 您从桌上拿起一小枚硬币，有困难吗？

1. No, I don’t have any difficulty 没有困难
2. I have difficulty but can still do it. 有困难但仍可以完成。
3. Yes, I have difficulty and need help. 有困难,需要帮助。
4. I can not do it. 无法完成

**[CAPI: IF (DB001=1&DB003 =1&…&DB009 =1), THEN SKIP TO DB016, 如果受访者在DB001 ~DB009 没有任何困难，请跳至DB016]**

**Here are a few more everyday acivities. Please tell me if you have any difficulties with these because of a physical, mental, emotional or memory problem. Again, exclude any that you expect to last less than three months. 下面我们想了解一下您日常生活的情况。请问您目前是否因为身体、精神、情感或者记忆方面的原因导致完成下面我们提到的一些日常行为有困难。我们指的“困难”不包括那些预计三个月内能够解决的困难。**

**DB010.** Because of health and memory problems, do you have any difficulty with dressing? Dressing includes taking clothes out from a closet, putting them on, buttoning up, and fastening a belt. 请问您是否因为健康和记忆的原因，自己穿衣服有困难？穿衣服包括从衣橱中拿出衣服，穿上衣服，扣上钮扣，系上腰带。

1. No, I don’t have any difficulty 没有困难
2. I have difficulty but can still do it. 有困难但仍可以完成。
3. Yes, I have difficulty and need help. 有困难,需要帮助。
4. I can not do it. 无法完成

**DB011.** Because of health and memory problems, do you have any difficulty with bathing or showering? 请问您是否因为健康和记忆的原因，洗澡有困难？

1. No, I don’t have any difficulty 没有困难
2. I have difficulty but can still do it. 有困难但仍可以完成。
3. Yes, I have difficulty and need help. 有困难,需要帮助。
4. I can not do it. 无法完成

**DB012.** Because of health and memory problems, do you have any difficulty with eating, such as cutting up your food? 请问您是否因为健康和记忆的原因，自己吃饭有困难，比如自己夹菜？(Definition: By eating, we mean eating food by oneself when it is ready. 定义：当饭菜准备好以后，自己吃饭定义为用餐。)

1. No, I don’t have any difficulty 没有困难
2. I have difficulty but can still do it. 有困难但仍可以完成。
3. Yes, I have difficulty and need help. 有困难,需要帮助。
4. I can not do it. 无法完成

**DB013.** Do you have any difficulty with getting into or out of bed? 您起床、下床有没有困难？

1. No, I don’t have any difficulty 没有困难
2. I have difficulty but can still do it. 有困难但仍可以完成。
3. Yes, I have difficulty and need help. 有困难,需要帮助。
4. I can not do it. 无法完成

**DB014.** Because of health and memory problems, do you have any difficulties with using the toilet, including getting up and down? 请问您是否因为健康和记忆的原因，上厕所有困难，包括蹲下、站起？

1. No, I don’t have any difficulty 没有困难
2. I have difficulty but can still do it. 有困难但仍可以完成。
3. Yes, I have difficulty and need help. 有困难,需要帮助。
4. I can not do it. 无法完成

**DB015.** Because of health and memory problems, do you have any difficulties with controlling urination and defecation? If you use a catheter (conduit) or a pouch by yourself, then you are not considered to have difficulties. 请问您是否因为健康和记忆的原因，控制大小便有困难？（自己能够使用导尿管或者尿袋算能够控制自理）

1. No, I don’t have any difficulty 没有困难
2. I have difficulty but can still do it. 有困难但仍可以完成。
3. Yes, I have difficulty and need help. 有困难,需要帮助。
4. I can not do it. 无法完成

**DB016.** Because of health and memory problems, do you have any difficulties with doing household chores? 请问您是否因为健康和记忆的原因，做家务活的时候有困难？(Definition: By doing household chores, we mean house cleaning, doing dishes, making the bed, and arranging the house. 定义：做家务，我们指的是房屋清洁，洗碗盘，整理被褥和房间摆设。)

[IWER: If R cannot mop the floor, but can scrub, or R cannot fold heavy bedding, but is able to do light ones, then mark (3). 访员注意：如果受访者不能拖地，但是可以擦洗桌子，或者受访者不能整理重的被褥，但是可以整理一些轻便的，请选择(3)。]

1. No, I don’t have any difficulty 没有困难
2. I have difficulty but can still do it. 有困难但仍可以完成。
3. Yes, I have difficulty and need help. 有困难,需要帮助。
4. I can not do it. 无法完成

**DB017.** Because of health and memory problems, do you have any difficulties with preparing hot meals? 请问您是否因为健康和记忆的原因，做饭有困难？ (Definition: By preparing hot meals, we mean preparing ingredients, cooking, and serving food. 定义：做饭我们定义为准备原材料，做饭菜，端上餐桌。)

[IWER: If another person prepares ingredients or if R can cook rice, but is not able to prepare side dishes, then mark (3). 访员注意：如果由于健康原因，受访者需要别人帮忙洗菜切菜，或者受访者只能自己煮米饭但不能做菜，也就是说，由于健康原因受访者只能完成做饭的一些简单的动作，那么选择（3）。]

1. No, I don’t have any difficulty 没有困难
2. I have difficulty but can still do it. 有困难但仍可以完成。
3. Yes, I have difficulty and need help. 有困难,需要帮助。
4. I can not do it. 无法完成

**DB018.** Because of health and memory problems, do you have any difficulties with shopping for groceries? By shopping, we mean deciding what to buy and paying for it. 请问您是否因为健康和记忆的原因，自己去商店买食品杂货有困难？我们这里说的买东西是指决定买什么和付钱。

1. No, I don’t have any difficulty 没有困难
2. I have difficulty but can still do it. 有困难但仍可以完成。
3. Yes, I have difficulty and need help. 有困难,需要帮助。
4. I can not do it. 无法完成

**DB019.** Because of health and memory problems, do you have any difficulties with managing your money, such as paying your bills, keeping track of expenses, or managing assets? 请问您是否因为健康和记忆的原因，管钱有困难，比如支付账单、记录支出项目、管理财物？

1. No, I don’t have any difficulty 没有困难
2. I have difficulty but can still do it. 有困难但仍可以完成。
3. Yes, I have difficulty and need help. 有困难,需要帮助。
4. I can not do it. 无法完成

**DB020.** Because of health and memory problems, do you have any difficulties with taking medications? By taking medications, we mean taking the right portion of medication right on time. 请问您是否因为健康和记忆的原因，自己吃药有困难？ 吃药是指能记得什么时间吃和吃多少。

1. No, I don’t have any difficulty 没有困难
2. I have difficulty but can still do it. 有困难但仍可以完成。
3. Yes, I have difficulty and need help. 有困难,需要帮助。
4. I can not do it. 无法完成

**SKIP PATTERN CHECKPOINT: FUNCTIONAL DIFFICULTIES 跳转检查点：功能障碍**

**IF R NEEDS ANY HELP IN DB010 ~DB020 (BOTH HELP TO SOME EXTENT, (3), AND IN EVERY RESPECT, (4)), SKIP TO DB021. 如果受访者在DB010 ~DB020 需要任何帮助（包括(3)有困难需要帮助，(4)无法完成），请跳至DB021 。**

**IF R DOES NOT NEED ANY HELP, SKIP TO DB029 如果被调查者不需要任何帮助请跳转至DB029**

**DB021.** Do you use the following auxiliary? (Code all that apply)你使用以下辅助么？（可多选）

1. Walking stick拐杖
2. Travel device代步器
3. Manual wheelchair手动轮椅
4. Electric Wheelchair电动轮椅
5. Not any 一个都不用

**DB022.** Who most often helps you with [make sure we ask this only once for ALL these activities; do not ask for each problem separately] (dressing, bathing, eating, getting out of bed, using the toilet, controlling urination and defecation, doing chores, preparing hot meals, shopping, managing money, making phone calls, taking medications) (May choose up to 3 persons)? 请问在以上困难中，谁帮助您最多(最多可选择3个)？（在穿衣、洗澡、吃饭、起床、入厕、控制大小便、家务、做饭、购物、管理钱物、打电话，吃药等困难中）

(Select from the list displayed by CAPI) （请从CAPI列表中选择）

1 Spouse 配偶

2 Mother 母亲

3 Father 　父亲

4 Mother-in-law 岳母/婆婆

5 Father-in-law 岳父/公公

6 ~ 30 Children 　子女

31 Sibling 　　兄弟姐妹

32 Sibling of spouse 配偶的兄弟姐妹

33 Brother-in-law, sister-in-law 　姐夫/妹夫/嫂子/弟媳

34 Spouse of child 孩子的配偶

35 Grandchild 　孙子女

36 Other relative 其他亲属

37 Paid helper(such as nanny) 雇佣人员（如：保姆）

38 Volunteer or Employee of facility 志愿者或者志愿机构人员

39 Other 其他人员

40 No one helped 　　　没有人提供帮助 →Skip to DB029请跳至 DB029

**PROCEDURE: For each helper 6-30 or 34 chosen in DB022, ask DB022_a. 如果DB022 选择 6-30或34（即子女或孩子配偶提供帮助最多），对每一个人询问DB022_a ：**

**DB022_a.** Whether it is the [helper’s name chosen from DB022] him/herself taking the time? [DB022选择的帮助者]亲自帮助您的吗？

1. Yes 是
2. No 否

**PROCEDURE: For each helper 1-40 chosen in DB022, ask DB023-DB026 对DB022 选择的1-40 的每一位提供帮助的人，提问DB023 -DB026 ：**

**DB023.** During the last month, on about how many days did [helper’s name chosen from DB022] help you?

在过去一个月内，[DB022 中选择的帮助者]帮助了您多少天？_____ 1..31Days天

**DB024.** On the days [helper’s name chosen from DB022 helps you, about how many hours per day is that?

在[DB022 中选择的帮助者]帮助您的那些天，他／她大概每天花多少小时帮助您？_____ 1..24Hours 小时 [IWER: less than an hour=1 访员注意：少于一个小时请记为1］

**DB025.** Is he/she living in your home? 他/她是否住在您家里？

1. Yes 是
2. No 否

**DB026.** Is [helper’s name chosen from DB022 paid to help you? 请问[DB022]中选择的帮助者]帮助您，您要付钱吗？

1. Yes 是
2. No 否

**SKIP PATTERN CHECKPOINT: PAID HELPER 跳转点检查：付费的帮手**

**IF R PAID FOR HELP IN DB026, SKIP TO DB027. 如果在DB026 受访者为获得帮助而支付费用，请跳转至DB027 .**

**IF R DID NOT PAY FOR HELP, SKIP TO DB029. 如果受访者不需要帮助付费，那么跳转至DB029 .**

**DB027.** About how much in total did you pay (including value of the goods you gave them as repayment for their help) for the help during the past month? 在过去一个月内您给这些帮助您的人大约总共付了多少钱？（包括作为报答给他们财物的总价值）_______ Yuan 元

**DB028.** Who contributed most to paying this cost? Please choose one person who paid the most. 这些钱谁出的最多，请选择一个支付的最多的人。(Select from the list displayed by CAPI) （请从CAPI列表中选择）

1 Yourself 您自己

2 Spouse 配偶

3 Mother 母亲

4 Father 　父亲

5 Mother-in-law 岳母/婆婆

6 Father-in-law 岳父/公公

7 ~ 31 Children 　子女

32 Sibling 　　兄弟姐妹

33 Sibling of spouse 配偶的兄弟姐妹

34 Brother-in-law, sister-in-law 　姐夫/妹夫/嫂子/弟媳

35 Spouse of child 孩子的配偶

36 Grandchild 　孙子女

37 Other relative 其他亲属

38 Other 其他人员

**DB029.**Do you use the following auxiliary? (Code all that apply)你使用以下辅助工具么？（可多选）

⑴ Walking stick 拐杖

⑵ Travel device 代步器

⑶ Manual wheelchair 手动轮椅

⑷ Electric Wheelchair 电动轮椅

⑸ catheter, urine collection bag 导尿管，导尿袋

⑹ Toilet Series 座便器

⑺ None of the above 以上都没有

**DB030.** Suppose that in the future, you needed help with basic daily activities like eating or dressing. Do you have relatives or friends (besides your spouse/partner) who would be willing and able to help you over a long period of time? 如果以后您在日常生活方面需要照顾，比如吃饭，穿衣，有亲人（除了配偶以外）或朋友能长期照顾您吗？

1. Yes 是
2. No 否 →Skip to DB032 请跳至DB032

**DB031.** What is the relationship to you of that person or those persons? （Choose all that apply） 他/她是您的什么人？（可多选） (Select from the list displayed by CAPI) （请从CAPI列表中选择）

1 Mother 母亲

2 Father 　父亲

3 Mother-in-law 岳母/婆婆

4 Father-in-law 岳父/公公

5 ~ 29 Children 　子女

30 Sibling 　　兄弟姐妹

31 Sibling of spouse 配偶的兄弟姐妹

32 Brother-in-law, sister-in-law 　姐夫/妹夫/嫂子/弟媳

33 Spouse of child 孩子的配偶

34 Grandchild 　孙子女

35 Other relative 其他亲属

36 Paid helper(such as nanny) 雇佣人员（如：保姆）

37 Volunteer or Employee of facility 志愿者或者志愿机构人员

38 Other 其他人员

**DB032.** How often did the respondent receive assistance in answering this section ?受访者填写该部分问卷时是否求助？

[IWER: If it is answered by a proxy, please record the respondent’s reaction.访员注意：如果是协助回答，请记录受访者的反应。]

1. Never 从不
2. A few times 　有一些时候
3. Most or all of the time 　大多数时候
4. The section was completed by a proxy respondent（the respondent is absent） 受访者不在场，完全请人代填 →Skip to DB033 请跳至DB033

**DB033.** What is your relationship to R? 您和受访者是什么关系？

[IWER: What is the proxy’s relationship to R? If unknown, please ask the proxy. 访员注意：代填问卷的人和受访者是什么关系。如果不清楚，请问代填者。]

1. Spouse 　 配偶
2. Mother 　 母亲
3. Father 　　 父亲
4. Mother-in-law 　岳母/婆婆
5. Father-in-law 　岳父公公
6. Sibling 　　　　兄弟姐妹
7. Brother-in-law, sister-in-law 　姐夫妹夫/嫂子弟媳
8. Child 　　　　　孩子
9. Spouse of child 　　孩子的配偶
10. Grandchild 　　　　孙子女
11. Other relative 　　　其他亲戚
12. Helper or other non-relative 　帮忙的人或者其他非亲属

**DB034.** [IWER: Please record the reason for proxy 访员注意：请记录代答的原因。]

What is the main reason for proxy（the respondent is absent）？受访者不在场，完全请人代填的主要原因是什么？

（1）The respondent has serious physical handicaps 受访者有严重身体障碍

（2）The respondent has serious mental handicaps 受访者有严重精神障碍

（3）The respondent has rejected this interview. 受访者拒访

（4）Other . 其他(DB034_1)

## DC COGNITION & DEPPRESSION 认知和抑郁

[IWER: If DB032 =4, then skip to Section E , health care and insurance. Sections Cc and Cd must not be answered by proxy respondents.] [访员注意：如果DB032 =4，请跳至E 部分医疗保健与保险。DC 、DE 部分不能由别人代答.]

**DC001.**. Now I’m going to ask several simple questions. Some may be easy and some may be hard to answer. Please try to answer as honestly as you can. Are you ready? Please tell me today’s date. （Check all that apply） 现在我将问几个问题，有些问题对于您来说可能很简单、有些问题可能就比较难。请告诉我今天的日期，是哪一年哪一月哪一日？（可多选）

[IWER: R doesn't have to answer in this order. If R is an elderly person and marked the date by lunar calendar, that date is correct if it matches with the solar calendar. You can check the accuracy, using the converter. 受访者不必按顺序回答，如果受访者是老人，并按农历来回答，而且如果日期对应的阳历是准确的，那么您可以按日历转换来检查其准确性。]

- 1. Year is correct 年正确
  2. Month is correct 月正确
  3. Day is correct 日正确

**DC002.** Please tell me the day of the week. Is it Monday, Tuesday, Wednesday, Thursday, Friday,

Saturday, or Sunday? 请告诉我今天是星期几？

- 1. Day of week OK/correct 正确
  2. Day of week not OK/incorrect 错误

**DC003.** What is the current season (among Spring, Summer, Fall, or Winter)? 现在是什么季节？

- 1. Season OK 季节正确
  2. Season not OK 季节不正确

**DC004.** How would you rate your memory at the present time? Would you say it is excellent, very good, good, fair or poor? 您觉得自己现在的记忆力怎么样？是极好，很好，好，一般还是不好？

(1) Excellent 极好

(2) Very good 很好

(3) Good 好

(4) Fair 一般

(5) Poor 不好

We are going to read a list consisting of 10 words and we would like you to memorize as many as you can. We deliberately made the list long to make it difficult for anyone to memorize all of the words; most people will only remember a few of them. Please listen carefully as we read the list because we cannot repeat it. When we finish reading the list, we will ask you to recall and tell us as many words as you can remember, and they don’t have to be in the order that you heard them. Is this explanation clear? 我们将会给您读十个词，请仔细听，我们不会重复读。请尽可能多地记。我们是有意列这么多词以增加记忆的难度。读完后，会请您回忆这些词，不需要按顺序。您明白了吗？

[IWER: Do not allow proxy answers.] [访员注意：请不要让代理人答]

| **(CC006_Version) (CC006_wordlisDC006 .** | **Randomly select a list of words to use. 随机选择一组词：**  **1. ADC006_A 2. BDC006_B 3. CCC006_C 4.DDC006_D** |
| --- | --- |

| **Read the list slowly, with an interval of about 2 seconds between each word. 慢慢地读，每词之间间隔2秒。** |
| --- |

| **LIST A** | **LIST B** | **LIST C** | **LIST D** |
| --- | --- | --- | --- |
| A01. RICE 米饭 | B01. STOOL 凳子 | C01. MOUNTAIN 山 | D01. WATER 水 |
| A02. RIVER 河流 | B02. FOOT 脚 | C02. STONE 石头 | D02. HOSPITAL 医院 |
| A03 DOCTOR 医生 | B03. SKY 天空 | C03. BLOOD 血液 | D03. TREE 树木 |
| A04. CLOTHES 衣服 | B04.MONEY 金钱 | C04. MOTHER 妈妈 | D04. FATHER 爸爸 |
| A05. EGG 鸡蛋 | B05. PILLOW 枕头 | C05. SHOES 鞋子 | D05. FIRE 火 |
| A06.CAT 小猫 | B06. DOG 小狗 | C06. EYE 眼睛 | D06. TOOTH 牙齿 |
| A07. BOWL 饭碗 | B07. HOUSE 房子 | C07. GIRL 女孩 | D07. MOON 月亮 |
| A08. CHILD 小孩 | B08. WOOD 木头 | C08. HOUSE 房子 | D08. VILLAGE 村子 |
| A09. HAND 手 | B09. SCHOOL 小学 | C09. ROAD 马路 | D09. BOY 男孩 |
| A10. BOOK 书 | B10. TEA 茶 | C10. SUN 太阳 | D10. TABLE 桌子 |

| **Now please let us know the words you are able to recall. Give R enough time to recall, approximately up to 2 minutes. 现在请您开始回忆。大约给受访者2分钟时间回忆。** |
| --- |

| (CC006)DC007 . | **Circle all the words mentioned by the R on the column. 请选出受访者回忆出来的选项中有的。** |
| --- | --- |

| **LIST A** | **LIST B** | **LIST C** | **LIST D** |
| --- | --- | --- | --- |
| A01. RICE 米饭DC007_A_1 | B01. STOOL 凳子DC007_B_1 | C01. MOUNTAIN 山DC007_C_1 | D01. WATER 水DC007_D_1 |
| A02. RIVER 河流DC007_A_2 | B02. FOOT 脚  DC007_B_2 | C02. STONE 石头DC007_C_2 | D02. HOSPITAL 医院DC007_D_2 |
| A03 DOCTOR 医生DC007_A_3 | B03. SKY 天空  DC007_B_3 | C03. BLOOD 血液DC007_C_3 | D03. TREE 树木DC007_D_3 |
| A04. CLOTHES 衣服DC007_A_4 | B04.MONEY 金钱DC007_B_4 | C04. MOTHER 妈妈DC007_C_4 | D04. FATHER 爸爸DC007_D_4 |
| A05. EGG 鸡蛋DC007_A_5 | B05. PILLOW 枕头 DC007_B_5 | C05. SHOES 鞋子DC007_C_5 | D05. FIRE 火DC007_D_5 |
| A06.CAT 小猫DC007_A_6 | B06. DOG 小狗DC007_B_6 | C06. EYE 眼睛DC007_C_6 | D06. TOOTH 牙齿DC007_D_6 |
| A07. BOWL 饭碗DC007_A_7 | B07. HOUSE 房子DC007_B_7 | C07. GIRL 女孩DC007_C_7 | D07. MOON 月亮DC007_D_7 |
| A08. CHILD 小孩DC007_A_8 | B08. WOOD 木头DC007_B_8 | C08. HOUSE 房子DC007_C_8 | D08. VILLAGE 村子DC007_D_8 |
| A09. HAND 手DC007_A_9 | B09. SCHOOL 小学DC007_B_9 | C09. ROAD 马路DC007_C_9 | D09. BOY 男孩DC007_D_9 |
| A10. BOOK 书DC007_A_10 | B10. TEA 茶DC007_B_10 | C10. SUN 太阳DC007_C_10 | D10. TABLE 桌子DC007_D_10 |
| **A96. NONE RECALLED 一个都不记得** | **B96. NONE RECALLED 一个都不记得** | **C96. NONE RECALLED一个都不记得** | **D96.NONE RECALLED一个都不记得** |
| **A97. REFUSE TO RECALL 拒绝回忆** | **B97. REFUSE TO RECALL拒绝回忆** | **C97. REFUSE TO RECALL 拒绝回忆** | **D97. REFUSE TO RECALL 拒绝回忆** |

**DC008.**  [CAPI automatically record the current time: hour and minute. CAPI自动输入现在的时间：___(DC008_1)时_____(DC008_2)分（24小时制）]。

Try to remember the words I just read to you. I'll ask you to recall them later. 请尽量记住我刚才念给您听的词，一会我将请您回忆它们。

[IWER: Read once more if R did not recall any of the words, up to 3 times, and then go on. If R does not recall any of the words,assure them that it is OK so that R will feel comfortable/at ease.] [访员注意：如果受访者没回忆出任何一个词汇，请再读1遍，直到读第3遍。如果受访者回忆不出这些词汇，请告诉他们没关系，以便让他们放松。]

The 10 items below refer to how you have felt and behaved during the last week. Choose the appropriate response. 下面10道问题是有关您上周的感觉及行为，每道题目的答案都是一样的，如卡片12所示，包括很少或者根本没有，不太多，有时或者说有一半的时间还是大多数的时间，请您选择合适的答案 。

**[Show Card 出示卡片12]**

DC009. I was bothered by things that don't usually bother me. 我因一些小事而烦恼。

1. Rarely or none of the time (<1 day) 很少或者根本没有（<1天）
2. Some or a little of the time (1-2 days) 不太多 （1-2天）
3. Occasionally or a moderate amount of the time (3-4 days) 有时或者说有一半的时间（3-4天）
4. Most or all of the time (5-7 days) 大多数的时间(5-7天)

DC010. I had trouble keeping my mind on what I was doing. 我在做事时很难集中精力。

1. Rarely or none of the time (<1 day) 很少或者根本没有（<１天）
2. Some or a little of the time (1-2 days) 不太多（１-２天）
3. Occasionally or a moderate amount of the time (3-4 days)　有时或者说有一半的时间（3-4天）
4. Most or all of the time (5-7 days) 大多数的时间（5-7天）

DC011. I felt depressed.我感到情绪低落。

1. Rarely or none of the time (<1 day) 很少或者根本没有（<１天）
2. Some or a little of the time (1-2 days) 不太多（１-２天）
3. Occasionally or a moderate amount of the time (3-4 days)　有时或者说有一半的时间（3-4天）
4. Most or all of the time (5-7 days) 大多数的时间（5-7天）

DC012. I felt everything I did was an effort.我觉得做任何事都很费劲。

1. Rarely or none of the time (<1 day) 很少或者根本没有（<１天）
2. Some or a little of the time (1-2 days) 不太多（１-２天）
3. Occasionally or a moderate amount of the time (3-4 days)　有时或者说有一半的时间（3-4天）
4. Most or all of the time (5-7 days) 大多数的时间（5-7天）

DC013. I felt hopeful about the future. 我对未来充满希望。

1. Rarely or none of the time (<1 day) 很少或者根本没有（<１天）
2. Some or a little of the time (1-2 days) 不太多（１-２天）
3. Occasionally or a moderate amount of the time (3-4 days)　有时或者说有一半的时间（3-4天）
4. Most or all of the time (5-7 days) 大多数的时间（5-7天）

DC014. I felt fearful. 我感到害怕。

1. Rarely or none of the time (<1 day) 很少或者根本没有（<１天）
2. Some or a little of the time (1-2 days) 不太多（１-２天）
3. Occasionally or a moderate amount of the time (3-4 days)　有时或者说有一半的时间（3-4天）
4. Most or all of the time (5-7 days) 大多数的时间（5-7天）

DC015.My sleep was restless.我的睡眠不好。

1. Rarely or none of the time (<1 day) 很少或者根本没有（<１天）
2. Some or a little of the time (1-2 days) 不太多（１-２天）
3. Occasionally or a moderate amount of the time (3-4 days)　有时或者说有一半的时间（3-4天）
4. Most or all of the time (5-7 days) 大多数的时间（5-7天）

DC016. I was happy. 我很愉快。

1. Rarely or none of the time (<1 day) 很少或者根本没有（<１天）
2. Some or a little of the time (1-2 days) 不太多（１-２天）
3. Occasionally or a moderate amount of the time (3-4 days)　有时或者说有一半的时间（3-4天）
4. Most or all of the time (5-7 days) 大多数的时间（5-7天）

DC017. I felt lonely. 我感到孤独。

1. Rarely or none of the time (<1 day) 很少或者根本没有（<１天）
2. Some or a little of the time (1-2 days) 不太多（１-２天）
3. Occasionally or a moderate amount of the time (3-4 days)　有时或者说有一半的时间（3-4天）
4. Most or all of the time (5-7 days) 大多数的时间（5-7天）

DC018. I could not get "going." 我觉得我无法继续我的生活。

1. Rarely or none of the time (<1 day) 很少或者根本没有（<１天）
2. Some or a little of the time (1-2 days) 不太多（１-２天）
3. Occasionally or a moderate amount of the time (3-4 days)　有时或者说有一半的时间（3-4天）
4. Most or all of the time (5-7 days) 大多数的时间（5-7天）

[IWER: Try to persuade R to answer if R refuses at first. Record the exact number R says.] [访员注意：尽力说服受访者作答。正确地记录受访者回答的数字。]

**DC019.** Let's try some subtraction of numbers this time. What does 100 minus 7 equal? 我们将问您一些减法。100减去7等于多少？______________

**DC020.** And 7 from that? 再减去7等于多少？____________

**DC021.** And 7 from that? 再减去7等于多少？____________

**DC022.** And 7 from that? 再减去7等于多少？____________

**DC023.**  And 7 from that? 再减去7等于多少？____________

**DC024.** [IWER: Please indicate whether the respondent used paper and pencil or any other aid when completing the number subtraction.] [访员注意：受访者在回答这些算术题时，是否用了纸、笔或其他辅助工具？]

- 1. used aid 用了辅助工具
  2. did not use aid 没用辅助工具

**[Show Card 出示卡片32]**

**DC025.** Do you see this picture? Please draw that picture on this paper. 你看到这张图片了吗？请在这张纸上把该图片画出来。

[IWER: Show the picture of two pentagons overlapped.] [访员注意：出示一张两个五角星重叠的图片]

- 1. Drew the picture 画出了图片
  2. Failed to draw the picture 不能画出该图片

**DC026.** [CAPI automatically record the current time: hour and minute. 输入现在的时间：___(DC026_1)时_____(DC026_2)分（24小时制）]

**DC027.** A little while ago, I read you a list of words and you repeated the ones you could remember. Please tell me any of the words that you remember now. 刚才我给您读了一些词汇，您也重复了您记得的词汇。请告诉我现在您还记得的词汇。

[IWER: Answers are displayed only for interviewer. Please do not show the screen to R.] [访员注意：只有访员可以看到答案。请勿将显示屏展示给受访者看。]

|  | **Circle all the words mentioned by R in the column and list all of the words mentioned which are not on the list in row 51-55. 请选出受访者回忆出来的选项中有的。** | | | |
| --- | --- | --- | --- | --- |
| **LIST A** | | **LIST B** | **LIST C** | **LIST D** |
| A01. RICE 米饭DC027_A_1 | | B01. STOOL 凳子DC027_B_1 | C01. MOUNTAIN 山DC027_C_1 | D01. WATER 水DC027_D_1 |
| A02. RIVER 河流DC027_A_2 | | B02. FOOT 脚DC027_B_2 | C02. STONE 石头DC027_C_2 | D02. HOSPITAL 医院 DC027_D_2 |
| A03 DOCTOR 医生DC027_A_3 | | B03. SKY 天空DC027_B_3 | C03. BLOOD 血液DC027_C_3 | D03. TREE 树木DC027_D_3 |
| A04. CLOTHES 衣服DC027_A_4 | | B04.MONEY 金钱DC027_B_4 | C04. MOTHER 妈妈DC027_C_4 | D04. FATHER 爸爸DC027_D_4 |
| A05. EGG 鸡蛋DC027_A_5 | | B05. PILLOW 枕头 DC027_B_5 | C05. SHOES 鞋子DC027_C_5 | D05. FIRE 火DC027_D_5 |
| A06.CAT 小猫DC027_A_6 | | B06. DOG 小狗DC027_B_6 | C06. EYE 眼睛DC027_C_6 | D06. TOOTH 牙齿DC027_D_6 |
| A07. BOWL 饭碗DC027_A_7 | | B07. HOUSE 房子DC027_B_7 | C07. GIRL 女孩DC027_C_7 | D07. MOON 月亮DC027_D_7 |
| A08. CHILD 小孩DC027_A_8 | | B08. WOOD 木头DC027_B_8 | C08. HOUSE 房子DC027_C_8 | D08. VILLAGE 村子DC027_D_8 |
| A09. HAND 手DC027_A_9 | | B09. SCHOOL 小学DC027_B_9 | C09. ROAD 马路DC027_C_9 | D09. BOY 男孩DC027_D_9 |
| A10. BOOK 书DC027_A_10 | | B10. TEA 茶DC027_B_10 | C10. SUN 太阳DC027_C_10 | D10. TABLE 桌子DC027_D_10 |
| **A96. NONE RECALLED 一个都不记得** | | **B96. NONE RECALLED 一个都不记得** | **C96. NONE RECALLED一个都不记得** | **D96. NONE RECALLED一个都不记得** |
| **A97. REFUSE TO RECALL拒绝回忆** | | **B97. REFUSE TO RECALL拒绝回忆** | **C97. REFUSE TO RECALL拒绝回忆** | **D97. REFUSE TO RECALL拒绝回忆** |

**DC028.** Please think about your life-as-a-whole. How satisfied are you with it? Are you completely satisfied, very satisfied, somewhat satisfied, not very satisfied, or not at all satisfied? 总体来看，您对自己的生活是否感到满意？是极其满意，非常满意，比较满意，不太满意还是一点也不满意？

（1）Completely satisfied 极其满意

（2）Very satisfied 非常满意

（3）Somewhat satisfied 比较满意

（4）Not very satisfied 不太满意

（5）Not at all satisfied 一点也不满意

##

## DE SELF-REPORTED HEALTH &VIGNETTES 自我评价健康和情景选择题

[IWER: Sections DE must NOT be answered by proxy respondents. 访员注意：DE部分不能由他人代答。]

Vignettes will be presented ONLY to a random subsample of households (half). All selected Rs will answer the first 6 questions, and will then get 2 randomly-selected domains out of the 6 domains (each domain has 3 questions). All eligible members in the selected households get the same 12 questions. Do NOT allow proxy answers. 仅仅在二次抽样被选中的家庭中询问情景选择题。所有选中的受访者应先回答前面6个问题，随后会在这6个问题中抽出2个方面要求受访者作答（每个方面有三个问题）。选中的家庭中所有适龄成员将需要回答同样的12个问题。请不要让代理人作答。

We would now like to ask you some questions about your health. Every subject of the options are the same, as shown in card 13, including none, mild, moderate, severe and extreme, please select the appropriate answer. Please choose one of the five answers for every question.我们现在问一些关于您的健康的问题。每道题目的选项都是一样的，如卡片13所示，包括没有，轻微，中等，严重和非常严重，请您选择合适的答案。

**[Show Card 出示卡片13]**

**DE001.** Overall in the last month, how much bodily aches or pains did you experience? 在过去一个月内，您身体有没有疼痛？程度如何？

1. None 没有
2. Mild 轻微
3. Moderate 中等
4. Severe 严重
5. Extreme 非常严重

**DE002.** In the last month, how much difficulty did you have with sleeping, such as having trouble falling asleep, waking up frequently during the night, or waking up too early in the morning? 在过去一个月内，您在睡眠方面有没有困难？比如难以入睡，夜里经常醒来，或早上过早地醒来。程度如何？

1. None 没有
2. Mild 轻微
3. Moderate 中等
4. Severe 严重
5. Extreme 非常严重

**DE003.** Overall in the last month, how much of a problem did you have with moving around? 在过去一个月内，不借助外界帮助，您走路有没有困难？程度如何？

1. None 没有
2. Mild 轻微
3. Moderate 中等
4. Severe 严重
5. Extreme 非常严重

**DE004.** Overall in the last month, how much difficulty did you have with concentrating or remembering things? 在过去一个月内，您在集中注意力或记东西方面有没有困难？程度如何？

1. None 没有
2. Mild 轻微
3. Moderate 中等
4. Severe 严重
5. Extreme 非常严重

**DE005.**  In the last month, how much of a problem did you have with shortness of breath? 在过去一个月内，您有没有喘不过来气的问题？程度如何？

1. None 没有
2. Mild 轻微
3. Moderate 中等
4. Severe 严重
5. Extreme 非常严重

**DE006.** Overall in the last month, how much of a problem did you have with feeling sad, low, or depressed? 在过去一个月内，您有没有感觉到悲伤、消沉或抑郁？程度如何？

1. None 没有
2. Mild 轻微
3. Moderate 中等
4. Severe 严重
5. Extreme 非常严重

We will now give you some examples of persons with serious and less serious health problems. We would like to know how you evaluate the health of these persons. Please assume that the persons have the same age and background as you. 现在，我们给您说几个健康有问题的人。我们想知道您怎么评价他们的健康状况。请您想象这些人和您具有相同的年龄和背景。

**[PROCEDURE: In DE007 -DE024 , there are 2 first names: one male and one female. The program should randomly choose which gender is used for each question, but use the same gender in a question for all persons within a HH. So if domains pain and sleep are chosen for HH A, then for each of the 6 questions for the 2 domains, 1 gender is randomly selected. That choice is used for the main respondent. 程序：DE007 -DE024 题中有两个名字：男名和女名。程序会随机选择男名或女名，但在一户人家只会选择同性别的名字。所以如果家户A抽到了疼痛和睡眠部分，主要受访者就需要回答每个部分中的6个题目，题目中的人名性别是随机选择的，但一户人家会用同样的男名或女名。]**

**Pain Domain 疼痛部分**

**DE007.** Zhang Jun/Wang Hong has a headache once a month that is relieved after taking a pill. During the headache he/she can carry on with his/her day-to-day affairs. Overall in the last month, how much of bodily aches or pains did Zhang San/Wang Hong have? 张军／王红每月都有一次头痛，吃药之后会得到缓解。头痛时，他／她能继续做日常工作。您认为，在过去一个月内，张三／王红身体有没有疼痛？程度如何？

1. None 没有
2. Mild 轻微
3. Moderate 中等
4. Severe 严重
5. Extreme 非常严重

**DE008.** Zhou Wei/Li Li has pain that radiates down his/her right arm and wrist during his/her day at work. This is slightly relieved in the evenings when he/she is no longer working on his/her computer. Overall in the last month, how much of bodily aches or pains did Zhou Wei/Li Li have? 在过去一个月内，周伟／李丽白天在电脑前工作时，他／她右边的胳膊和手腕都会庝，晚上在家不用打电脑时，疼痛会略微减轻。您认为，周伟／李丽身体有没有疼痛？程度如何？

1. None 没有
2. Mild 轻微
3. Moderate 中等
4. Severe 严重
5. Extreme 非常严重

**DE009.** Zhao Liang/Zhou Yan has pain in his/her knees, elbows, wrists, and fingers, and the pain is present almost all the time. Although medication helps, he/she feels uncomfortable when moving around and holding and lifting things. Overall in the last month, how much of bodily aches or pains did Zhao Liang/Zhou Yan have? 在过去一个月内，赵亮／张燕的膝盖、肘部、腰部和手指都有疼痛，而且疼个不停。他/她吃药以后会减轻，但走路、拎东西和举东西仍然吃力。您认为，赵亮／张燕身体有没有疼痛？程度如何？

1. None 没有
2. Mild 轻微
3. Moderate 中等
4. Severe 严重
5. Extreme 非常严重

**Sleep Domain 睡眠部分**

**DE010.** Zhao Yong/Wang Hua falls asleep easily at night, but two nights a week he/she wakes up in the middle of the night and cannot go back to sleep for the rest of the night. In the last month, how much difficulty did Zhao Yong/Wang Hua have with sleeping, such as falling asleep, waking up frequently during the night, or waking up too early in the morning? 在过去一个月内，赵勇／王花晚上睡觉时很快就能入睡，但每个星期都会有两天半夜醒来，就再也睡不着了。您认为，赵勇／王花的睡眠有没有困难，比如说难以入睡，夜里经常醒来，或早上过早地醒来？程度如何？

1. None 没有
2. Mild 轻微
3. Moderate 中等
4. Severe 严重
5. Extreme 非常严重

**DE011.** Li Gang/Li Hong takes about two hours every night to fall asleep. He/She wakes up once or twice a night feeling panicked and takes more than one hour to fall asleep again. In the last month, how much difficulty did Li Gan/Li Hong have with sleeping, such as falling asleep, waking up frequently during the night, or waking up too early in the morning? 在过去一个月内，李刚／李红每天晚上要花两个小时才能入睡。夜里会惊醒一两次，心里感到惊慌，然后花一个多小时才能再次入睡。您认为，李刚／李红的睡眠有没有困难，比如说难以入睡，夜里经常醒来，或早上过早地醒来？程度如何？

1. None 没有
2. Mild 轻微
3. Moderate 中等
4. Severe 严重
5. Extreme 非常严重

**DE012.** Wang Qiang/Xu Lin wakes up almost once every hour during the night. When he/she wakes up in the night, it takes around 15 minutes for him/her to go back to sleep. In the morning he/she does not feel well-rested. In the last month, how much difficulty did Wang Qiang/Xu Lin have with sleeping, such as falling asleep, waking up frequently during the night, or waking up too early in the morning? 在过去一个月内，王强／徐琳夜里几乎每小时都醒来一次。夜里醒来后，再次入睡要花15分钟。早上起床时，他／她会感到没有休息好。您认为，王强／徐琳的睡眠有没有困难，比如说难以入睡，夜里经常醒来，或早上过早地醒来？程度如何？

1. None 没有
2. Mild 轻微
3. Moderate 中等
4. Severe 严重
5. Extreme 非常严重

**Mobility Domain 行动部分**

**DE013.** Luo Hai/Hong Mei is able to walk distances of up to 200 meters without any problems but feels tired after walking one kilometer or climbing more than one flight of stairs. He/She has no problems with day-to-day activities, such as carrying food from the market. Overall in the last month, how much of a problem did Luo Hai/Hong Mei have with moving around? 在过去一个月内，罗海／洪梅走200米的路毫无困难。但走完一公里或爬完几层楼后，会感到累。他／她的日常活动没有问题，比如从市场上买完菜拎回家。您认为，罗海／洪梅在走路方面有没有困难？程度如何？

1. None 没有
2. Mild 轻微
3. Moderate 中等
4. Severe 严重
5. Extreme 非常严重

**DE014.** Chen Shi/Chen Hong does not exercise. He/She cannot climb stairs or do other physical activities because he/she is obese. He/She is able to carry the groceries and do some light household work. Overall in the last month, how much of a problem did Chen Shi/Chen Hong have with moving around? 陈实／陈红从不锻炼身体。因为身体肥胖，他／她不能爬楼梯或做其他的体力活动。但是能去市场上买回少量菜，也能做一些轻松的家务。您认为，在过去一个月内，陈实／陈红在走路方面有没有困难？程度如何？

1. None 没有
2. Mild 轻微
3. Moderate 中等
4. Severe 严重
5. Extreme 非常严重

**DE015.** Li Bin/Zhang Lan has a lot of swelling in his/her legs due to his/her health condition. He/She has to make an effort to walk around his/her home as his/her legs feel heavy. Overall in the last month, how much of a problem did Li Bin/Zhang Lan have with moving around? 在过去一个月内，李斌／张兰因为健康原因，腿肿得很厉害。因为感到腿沉重，所以在家走动都很费劲。您认为，李斌／张兰在走路方面有没有困难？程度如何？

1. None 没有
2. Mild 轻微
3. Moderate 中等
4. Severe 严重
5. Extreme 非常严重

**Cognition Domain 认知部分**

**DE016**. Zhang Liang/Liu Jia can concentrate while watching TV, reading a magazine, or playing a game of cards or chess. Once a week he/she forgets where his/her keys or glasses are, but finds them within five minutes. Overall in the last month, how much difficulty did Zhang Liang/Liu Jia have with concentrating or remembering things? 在过去一个月内，张良／刘佳在看电视、阅读杂志、打牌或下棋时都能集中注意力。但是每周都有一次会忘记把钥匙或眼镜放在哪里，但每次都能在5分钟之内找到。您认为，张亮／刘佳在集中注意力或记东西方面有没有困难？程度如何？

1. None 没有
2. Mild 轻微
3. Moderate 中等
4. Severe 严重
5. Extreme 非常严重

**DE017.** Liu Jun/Song Li is keen to learn new recipes but finds that he/she often makes mistakes and has to reread a recipe several times before he/she is able to do make a new dish properly. Overall in the last month, how much difficulty did Liu Jun/Song Li have with concentrating and remembering things? 刘军／宋丽喜欢照着菜谱学做新菜，但做菜时经常做错。他/她经常要回去再读好几遍才能学会这个菜的做法。您认为，在过去一个月内，刘军／宋丽在集中注意力或记东西方面有没有困难？程度如何？

1. None 没有
2. Mild 轻微
3. Moderate 中等
4. Severe 严重
5. Extreme 非常严重

**DE018.** Li Wei/Yang Wen cannot concentrate for more than 15 minutes and has difficulty paying attention to what is being said to him/her. Whenever he/she starts a task, he/she never manages to finish it and often forgets what he/she was doing. He/She is able to learn the names of people he/she meets. Overall in the last month, how much difficulty did Li Wei/Yang Wen have with concentrating or remembering things? 在过去一个月内，李伟／杨文集中注意力的时间每次不超过15分钟，别人跟他/她说话的时候他很难专心去听。每次他／她要做一件事的时候，都做不完，常常忘记自己要做的是什么，不过当他/她认识新的人的时候，能记住人家的名字。您认为，李伟／杨文在集中注意力或记东西方面有没有困难？程度如何？

1. None 没有
2. Mild 轻微
3. Moderate 中等
4. Severe 严重
5. Extreme 非常严重

**Breathing Domain 呼吸部分**

**DE019.** Zhou Yang/Huang Rong has no problems with walking slowly. He/She gets out of breath easily when climbing uphill for 20 meters or a flight of stairs. In the last month, how much of a problem did Zhou Yang /Huang Rong have with shortness of breath? 在过去一个月内，周洋／黄蓉慢慢地走路没有问题，但上坡走20米或爬一层楼时，就喘不过来气。您认为，他／她在过去一个月内，有没有喘不过来气的问题？程度如何？

1. None 没有
2. Mild 轻微
3. Moderate 中等
4. Severe 严重
5. Extreme 非常严重

**DE020.**. Peng Lai/Sun Yue suffers from respiratory infections about once every year. He/She is short of breath 3 or 4 times a week and had to be admitted to the hospital twice in the past month with a bad cough that required treatment with antibiotics. In the last month, how much of a problem did Peng Lai/Sun Yue have with shortness of breath? 彭来／孙悦每年都会得一次呼吸道感染。他／她每周都会有三、四次喘不上气。在过去一个月内，他／她因为严重咳嗽不得不两次住进医院接受抗生素治疗。您认为，在过去一个月内，他／她在过去一个月内，有没有喘不过来气的问题？程度如何？

1. None 没有
2. Mild 轻微
3. Moderate 中等
4. Severe 严重
5. Extreme 非常严重

**DE021.** He Ming/Zhang Fang has been a heavy smoker for 30 years and wakes up with a cough every morning. He/She gets short of breath even while resting and does not leave the house anymore. He/She often needs to be put on oxygen. In the last month, how much of a problem did He Ming/Zhang Fang have with shortness of breath? 何明／章芳抽很多烟，已经抽了30年了。在过去一个月内，每天早上醒来都会咳嗽。即使休息的时候也会经常喘不过来气，因此他／她不再出门，还经常需要吸氧气。您认为，他／她在过去一个月内，有没有喘不过来气的问题？程度如何？

1. None 没有
2. Mild 轻微
3. Moderate 中等
4. Severe 严重
5. Extreme 非常严重

**Affect Domain 情绪部分**

**DE022.** Wang Dong/Tang Jing enjoys his/her work and social activities and is generally satisfied with his/her life. He/She gets depressed every 3 weeks for a day or two and loses interest in what he/she usually enjoys but is able to carry on with his/her day-today activities. Overall in the last month, how much of a problem did Wang Dong/Tang Jing have with feeling sad, low, or depressed? 王栋／唐静喜欢她的工作，也有不少朋友，一般来说对自己的生活感到满意。但是每隔三个星期，他／她就会有一两天感到郁闷，对他／她平时喜欢的事物丧失兴趣。这种情况并不妨碍他／她继续做日常活动。您认为，在过去一个月内，他／她有没有感觉到悲伤、消沉或抑郁？程度如何？

1. None 没有
2. Mild 轻微
3. Moderate 中等
4. Severe 严重
5. Extreme 非常严重

**DE023.**Li Feng/Zhang Yan feels nervous and anxious. He/She worries and thinks negatively about the future, but feels better in the company of people or when doing something that really interests him/her. When he/she is alone he/she tends to feel useless and empty. Overall in the last month, how much of a problem did Li Feng/Zhang Yan have with feeling sad, low, or depressed? 在过去一个月内，李峰／张艳感到紧张和焦虑，对未来很悲观。但是，当与别人在一起的时候，或者做一些真正令他／她感兴趣的事情时，心情会好一些。而当他/她一个人呆着的时候，他会感到自己毫无价值，很空虚。您认为，在过去一个月内，他／她有没有感觉到悲伤、消沉或抑郁？程度如何？

1. None 没有
2. Mild 轻微
3. Moderate 中等
4. Severe 严重
5. Extreme 非常严重

**DE024.** Zheng Bo/Wu Na feels depressed most of the time. He/She weeps frequently and feels hopeless about the future. He/She feels that he/she has become a burden on others and that he/she would be better dead. Overall in the last month, how much of a problem did Zheng Bo/Wu Na have with feeling sad, low, or depressed? 在过去一个月内，郑波／吴娜在大多数时候都会感到抑郁。他／她经常哭，并对未来没有希望，觉得自己已经成为别人的负担，而死是他／她最好的选择。您认为，在过去一个月内，他／她有没有感觉到悲伤、消沉或抑郁？程度如何？

1. None 没有
2. Mild 轻微
3. Moderate 中等
4. Severe 严重
5. Extreme 非常严重

# **E HEALTH CARE AND INSURANCE 医疗保健与保险**

**CARD21: Health Facilities 医疗机构**

1. General hospital 综合医院（即全科医院，不包括中医院）
2. Specialized hospital 专科医院（不包括中医院）
3. Chinese medicine hospital 中医院
4. Community healthcare center 社区卫生服务中心
5. Township hospital 乡镇卫生院
6. Health care post 卫生服务站
7. Village clinic/Private clinic 村诊所/私人诊所
8. Other 其他

**CARD20: Health Insurance 医疗保险**

1. Urban employee medical insurance (yi-bao) 城镇职工医疗保险（医保）
2. Urban resident medical insurance 城镇居民医疗保险
3. New cooperative medical insurance (he-zuo-yi-liao) 新型农村合作医疗保险（合作医疗）
4. Urban and rural resident medical insurance 城乡居民医疗保险（合并城镇居民和新型农村合作医疗保险）
5. Government medical insurance (gong-fei) 公费医疗
6. Medical aid 医疗救助
7. Private medical Insurance: Purchased by R’s union 商业医疗保险: 单位购买
8. Private medical Insurance: Purchased by Individual 商业医疗保险: 个人购买
9. Other medical insurance (specify) 其他医疗保险 （请注明）
10. No insurance 没有保险

**CARD23: Health Facilities for Inpatient Care 住院治疗的医疗机构**

(1) General Hospital 综合医院（即全科医院，不包括中医院）

(2) Specialized hospital 专科医院（不包括中医院）

(3) Chinese Medicine Hospital 中医院

(4) Community Healthcare Center 社区卫生服务中心

(5) Township Hospital 乡镇卫生院

(6) Health care post 卫生服务站

(7) Other 其他

## PART I MEDICAL INSURANCE 第一部分：医疗保险

**Now we would like to know about health insurance or benefits that you might have. 现在我们想了解一下您享受的健康保险或福利。**

**[Show Card 出示卡片14]**

**EA001.** Are you the policy holder/primary beneficiary of any of the types of health insurance listed below? (circle all that apply) 您本人目前是否参加了以下医疗保险？ (可多选)

(1) Urban employee medical insurance (yi-bao) 城镇职工医疗保险(医保)

(2) Urban resident medical insurance 城镇居民医疗保险

(3) New cooperative medical insurance (he-zuo-yi-liao) 新型农村合作医疗保险（合作医疗）

(4) Urban and rural resident medical insurance 城乡居民医疗保险（合并城镇居民和新型农村合作医疗保险）

(5) Government medical insurance (gong-fei) 公费医疗

(6) Medical aid 医疗救助

(7) Private medical Insurance: Purchased by R’s union 商业医疗保险: 单位购买

(8) Private medical Insurance: Purchased by Individual 商业医疗保险: 个人购买

(9)Other medical insurance (specify) 其他医疗保险, 请注明_______ (EA001_1)

(10)No insurance 没有保险

[Soft check: If pick 10, cannot pick any other, “you chose no insurance and a specific type of insurance, this is not possible” 如果选10, 不能选其他项, “您不能既选择没有保险，又选择有某一种保险”]

F1：（1）“城镇职工医疗保险”指依法对城镇职工的基本医疗权利给予保障的社会医疗保险制度。

（2）“城镇居民医疗保险”指2007年7月国务院决定在全国79个城市实行城镇居民基本医疗保险试点。参保范围：城镇非从业居民，包括：不属于城镇职工基本医疗保险制度覆盖范围的中小学阶段的学生（包括职业高中、中专、技校学生）、少年儿童和其他非从业城镇居民。

（3）“新型农村合作医疗保险“指由政府组织、引导、支持，农民自愿参加，个人、集体和政府多方筹资，以大病统筹为主的农民医疗互助共济制度。采取个人缴费、集体扶持和政府资助的方式筹集资金。

（4）“城乡居民医疗保险”指一些地区率先将城镇居民医疗保险与新型农村合作医疗合并，推行统一的城乡居民医疗保险制度。

（5）“外来务工人员医疗保险”指在某些地区在城镇职工基本医疗保险以外，专为外来务工人员设置的一种医疗保险制度。

（6）“公费医疗”指国家为保障国家工作人员身体健康而实行的一项社会保障制度。其享受对象主要是各级国家机关、党派、团体以及文化、教育、科研、卫生、体育等事业单位的工作人员和离退休人员，二等乙级以上革命伤残军人以及在校大学生等。

（7）“医疗救助”指在政府的支持下，依靠社会力量建立的旨在向困难群体提供某些或全部基本医疗服务的制度。

（8）“商业医疗保险”是相对于社会保险而言的。商业医疗保险由商业保险公司经办，以营利为目的，企业或职工自愿参加。

（9）“其他医疗保险”如中国职工保险互助会《在职职工重大疾病互助保障计划》和《生育保险》。

（10）“医疗救助”在部分区县试点，救助标准是由各区县根据自身经济情况制定，导致救助标准有高有低。主要三种，一是提供社会医疗救助金，给救助对象以经济补偿；二是给医疗机构一定的经济补贴，使后者直接减免救助对象的部分医疗费；三是由社会医疗救助机构举办专门医疗机构，免费为救助对象提供医疗服务。

（11）“医疗保险”是指基本医疗保险，是为补偿疾病所带来的医疗费用的一种保险。职工因疾病、负伤、生育时，由社会或企业提供必要的医疗服务或物质帮助的社会保险。如中国的公费医疗、劳保医疗。

**For each circled type of insurance (1-11), ask the following questions EA002 --- EA008 .**

**对以上选择的1-11的保险类型，回答以下问题 EA002 --- EA008**

**EA002.** Do you have supplemental insurance to this plan? 你有没有参加补充医疗保险？ （例如大病医疗等）

（1） Yes 有

（2） No 无

F1：“补充医疗保险”指由于国家的基本医疗保险只能满足参保人的基本医疗需求，超过基本医疗保险范围之外的医疗需求可以通过补充医疗保险予以补充。是相对于基本医疗保险而言的，包括企业补充医疗保险、商业医疗保险、社会互助和社区医疗保险等多种形式。

**EA003.** Where did you set up your insurance account/policy? 您的保险是在哪里办的?

(1)This county 本县/市

(2) (if it is not in this county) the place of your HuKou (如户口不在本县/市）户籍所在地

(3) other其它_______(EA003_1)province省_________(EA003_2)county市/县

**EA004.** Method of reimbursement 报销方式如何？

(1) Get reimbursement immediately 看病当时即可报销

(2) You pay first and get reimbursed later. 自己当时先垫付以后报销

**EA005.** Through which agency did you purchase your primary plan? 您的主要保险是通过什么机构办理的？

(1) Community committee/ village committee 居委会/村委会

(2) R’s union 单位

(3) Agency of Social insurance 社保经办机构

(4） Private insurance company 商业保险机构

(5) Other 其他

**EA006.** What’s your out-of-pocket yearly premium? 您每年要自己缴纳多少保险费(包括主保险费和补充医疗保险费)？____Yuan 元 [soft check upper limit: 15,000 for choice 1, 3,000 choice 2, 1,000 choice 3, 1,500 choice 4, 4,000 choice 7, 2,500 choice 9 程序检查：上限：选项1，15,000；选项2，3,000；选项3，1,000；选项4，1,500；选项7，4,000；选项9，2,500]

**EA007.** Who pay the premium for you? (choose all that apply) 谁给您交保费？(可多选)

- 1. Myself 自己
  2. Children 子女
  3. Relatives 亲属
  4. Government 政府
  5. R’s union 单位（包括村集体）
  6. Loan 借款
  7. Donate 捐赠
  8. Others 其他 Specify 请注明(EA007_1)

**EA008.** When did this benefit begin? 您是什么时候参加这份保险的？

[IWER: Mark the year using four digits. Take down the month as its actual number. For example, write January as “1” not “01”, December as “12”. If do not remember month, fill ‘0’. 访员注意: 用4位数表示年，按照实际的月份填写月。例：1月写作“1”，而不是“01”,12月写作“12”。如果记不住月份，请填入’0’。]

1900..2011Year 年(EA008_1)└─┴─┴─┴─┘，0..12Month 月(EA008_2)└─┴─┴

**Skip to EC001 请跳至EC001**

##

## PART II HEALTH CARE COSTS AND UTILIZATION 第二部分：医疗成本与使用情况

[IWER: Please do not allow proxy to answer Part II. 访员注意： “医疗成本与使用情况” 这部分不允许请别人代为回答]

**EC001.** When did you take the last physical examination? 您最近一次常规体检时什么时候？

[IWER: Mark the year using four digits. Take down the month as its actual number. For example, write January as “1” not “01”, December as “12”. If do not remember month, fill ‘0’. 访员注意: 用4位数表示年，按照实际的月份填写月。例：1月写作“1”，而不是“01”,12月写作“12”。如果记不住月份，请填入’0’。]

1900..2011Year 年(EC001_1)└─┴─┴─┴─┘0..12Month 月(EC001_2)└─┴─┴

**EC002.** Who paied the physical examination cost? 谁付的体检费？

1. Myself 自己
2. Children 子女
3. Relatives 亲属
4. Government 政府
5. R’s union 单位（包括村集体）
6. R’s insurance 保险
7. Loan 借款
8. Donate 捐赠
9. Others 其他____ Specify 请注明____(EC002_1)

**The next questions pertain to medical facilities or medical providers you may have visited for outpatient care during the past 1 month (excluding hospitalization). 下面我们要问问您过去一个月，到门诊看病或者接受治疗的情况（不包括住院）。**

**ED001.** In the last month have you visited a public hospital, private hospital, public health center, clinic, or health worker’s or doctor’s practice, or been visited by a health worker or doctor for outpatient care? 过去一个月里，您是否去医疗机构看过门诊或者接受过上门医疗服务？

(1) Yes 是

(2) No 否

**ED002.** Have you been ill in the last month? 您在过去一个月内生过病吗？

(1) Yes 是

(2) No 否

If ED001 =2 and ED002 =2 skip to EE001 如果ED001 =2 并且ED002 =2，请跳至EE001

If ED001 =1 skip to ED004 如果ED001 =1，请跳至ED004

If ED002 =1 and ED001=2 go to ED003. 如果ED002=1并且ED001 =2，请跳至ED003

**ED003.** What’s the main reason for not seeking medical treatment? 您没有去看病的主要原因是什么？

- - 1. Already under treatment. 之前已经看过医生了
    2. Illness is not serious. Don’t need treatment 病情不严重，不需要看医生
    3. Poor 没有钱看病
    4. No time 没有时间看病
    5. Inconvenient traffic 去医院交通不方便
    6. Poor service 医院服务态度不好
    7. No available treatment 认为看医生没有用
    8. Other 其他

**Skip to EE003 请跳至EE003**

**[Show Card 出示卡片15]**

**ED004.** Which types of medical facilities have you visited in the last 4 weeks for outpatient treatment?（circle all that apply）过去一个月，您去过哪些医疗机构接受门诊治疗？（可多选）

1. General hospital 综合医院（即全科医院，不包括中医院）
2. Specialized hospital 专科医院（不包括中医院）
3. Chinese medicine hospital 中医院
4. Community healthcare center 社区卫生服务中心
5. Township hospital 乡镇卫生院
6. Health care post 卫生服务站
7. Village clinic/Private clinic 村诊所/私人诊所
8. Other 其他

**PROCEDURE: For each item 1-7 checked in ED004 , ask ED005对ED004 选择的 1-7 的每一种医疗机构，提问ED005：**

**ED005.** How many times did you visit / been visited by [...] during the last month? 过去一个月中，您去这家医疗机构看过几次门诊？└─┴─┘ Times 次

**If sum(ED005)>1, then ask ED006; otherwise, skip ED006。如果ED005中的次数总和>1, 则问ED006；否则，就跳过ED006。**

**ED006.** How much did all the visits to [ED004 answer] cost during the last month? 您过去一个月去[ED004 answer]看病的总费用大概是多少?

IWER: If possible, please check the list of cost. 访员注意：如果可能，查看一下明细单

A. 1. Total cost 总费用_____(ED006_1)Yuan 元; [soft check upper bound: 30,000 上限：30,000]

2. Didn’t pay anything 没有付任何钱

**PROCEDURE: IF ED006 = 1, ASK ED007**

**ED007.**

B. 1. Self-paid part 其中自付部分_____ (ED007_1)Yuan 元

2. Didn’t pay anything. 没有付任何钱

**Now I’d like to ask you some questions about your most recent visit to a health care provider in the last month. 下面我想了解一下您过去一个月最近一次就诊的情况。**

**ED008.**  Which health care provider did you visit most recently during the past month? 过去一个月，您最近一次去了哪种医疗机构就诊？

[CAPI：Preload the health care providers in ED004. 程序：加载ED004填的医疗机构。]

**ED009.**  Is this facility public or private? 这家机构是公立的还是私立的？

1. Public 公立的
2. Private 私立的

**If ED008 =1-3, ask ED010 如果ED008 =1-3，请回答ED010**

**ED010.** What’s the level of this facility? 这家医疗机构是什么级别的？

1. County/district 县/市/区级
2. Regional /city 地/市
3. Provincial/ affiliated to a ministry 省/部属
4. Military 军队
5. Others 其他
6. Not applicable 不适用

**ED011.** What is the name of this health care provider? 这家医疗机构的名称是什么？______

[IWER: If unknown, please write “unknown”.访员注意：如果不知道医疗机构的名称，请填“不知名”。]

**ED012.** Did the provider visit you at home? 这次是不是医护人员上门服务？

（1） Yes 是 🡪 skip ED013 to ED016 and ED022，跳过ED013 到 ED016 和ED022

（2） No 否

**ED013.** How many kilometers is it from the medical facility to your residence? 从您家到这家医疗机构有多远？└─┴─┴─┘.└─┴─┘ Km 公里 [softcheck upper bound: 200 上限：200]

**ED014.** What is the travel time (one-way) to that facility? 您上次去这家医疗机构单程花多少时间？

└─┴─┘(ED014_1)

01. Minute 分钟 [softcheck range:1-59 范围：1-59]

02. Hour 小时 [softcheck upper limit: 10 上限：10]

方式？

(ED014_2)

1 Walk 步行

2 Bus 公交车

3 Car 私家车或出租车

4.Ambulance 救护车

5 Bicycle or other manual vehicles 自行车或其他人力车

6.Electric bicycle/electric tricycle 电动自行车/电动三轮车

7. Motorcycle 摩托车

8. Tractor 拖拉机

9. Train 火车

10. Animal or animal-pulled cart 骑牲畜或坐畜力车

**If ED014 =1, skip ED015 如果ED014 =1，跳过ED015**

**ED015.** What was the total transportation cost to the facility (including fuel cost, one way trip)? 您上次去这家医疗机构的单程交通成本是多少？（包括燃料费）

└─┴─┴─┘,└─┴─┴─┘RMB 元 [softcheck upper limit: 600 上限：600]

**ED016.** Where is the health care provider located? 这家医疗机构的具体地址是？

1. province 省(ED016_1)

A. This province 本省

B. Other province, (preload province) 外省（选择省份(ED016_1_1)

[IWER: Choose from the list of provinces，see appendix 2 请选择省份，见附表二]

2. county/city县/市(ED016_2)

A. This county / city 本县/市

B. Other county / city, specify 外县/市, (ED016_2_1) city市,______(ED016_2_2)county县

3. township/district 乡/区(ED016_3)

A. This township/district 本乡/ 区

B. Other township/district, specify 外乡/区 (ED016_3_1) 乡/区

4. village/street 村/街道(ED016_4)

A. This village/street 本村/街道

B. Other village/street, specify 外村/街道 (ED016_4_1) 村/街道

**ED017.** What was the purpose of your visit? (circle all that apply) 您上次去这家医疗机构的目的是什么？(可多选)

1. Immunization 免疫接种
2. Consultation 咨询
3. Medical check-up 体检
4. Treatment for Illness 看病
5. Other 其他

**If ED017 =4, then ask ED018 —ED021, ELSE ASK ED022.**

**如果ED017 答案中包含 4“看病”，继续问ED018 —ED021 的问题，否则提问ED022**

**ED018.** Could you tell me the disease name? 您能告诉我您得了什么病吗？_________

**ED019.** Was the visit a first visit or a follow-up visit for the symptom? 这次看病是第一次就诊还是复诊？

（1） First 第一次就诊

（2） Follow-up 复诊

**ED020 .** Was the visit for ordinary outpatient service or an emergency? 这次看病是普通门诊还是急诊？

（1）普通门诊

（2）急诊

**[Show Card 出示卡片16]**

**ED021.** What kind of treatment did you receive? (circle all that apply) 您这次去看病接受了以下哪种治疗？(可多选)

1. Injection 注射
2. Laboratorium test 化验
3. Surgery 手术
4. X-ray, CT, B ultrasonic, MRI X-光、CT、B超、核磁共振等检查
5. Medications and purchase medical 用药、拿药
6. IV (Drip Infusion) 输液
7. Traditional treatment ,eg massage, acupuncture 中医治疗，如按摩、针灸等

(8) Other 其他

**ED022.** Upon arrival, how long did you have to wait to be examined? 最近一次就诊，您排队花了多长时间？└─┴─┘

01. Minute 分钟 [range 范围： 1-59]

02. Hour 小时 [softcheck upper limit 上限: 6]

(ED022_1)

**ED023.** What was the total cost of this visit, including both treatment and medication cost (includes prescriptions you received)? 这次就诊(包括药费和诊疗费，药费包括在这家医疗机构或其他药店购买医生开的处方药)总共花了多少钱？

1.└─┴─┘,└─┴─┴─┘,└─┴─┴─┘ RMB 元(ED023_1) [softcheck upper limit 上限: 30,000]

2. There was no cost 没有费用

**PROCEDURE: IF ED023 = 1, ASK ED024**

**ED024.**  how much did you pay out of pocket, after reimbursement from insurance?？ 这次就诊所花的费用里，您自己支付了多少？

1.└─┴─┘,└─┴─┴─┘,└─┴─┴─┘ RMB 元(ED024_1) [soft check upper limit: 30,000, also ED025 <=ED023_1, else “Pay out of pocket cannot be more than total cost” 上限：30,000，而且ED025 <=ED023_1，因为“自己支付的费用不可能多于总费用”]

2. Did not pay anything 没有付任何钱 Go to ED026 请跳至ED026

**ED025.** Who contribute most for paying the out-of-pocket cost? 自付部分谁给您交得最多？

1. Myself 自己
2. Children 子女
3. Relatives 亲属
4. Government 政府
5. R’s union 单位（包括村集体）
6. Loan 借款
7. Donate 捐赠
8. Others 其他 Specify 请注明

**ED026.** What was the total medication cost for this visit, including prescriptions you received? 这次就诊所付的药费(包括在这家医疗机构或其他药店购买医生开的处方药)一共是多少钱？

1.________ RMB 元(ED026_1) [soft check upper limit: 5,000, and must be no more than ED023_1. 上限：5,000，而且不能多于ED023_1]

2. Doctor did not write a prescription 没有开药 skip ED027 跳过ED027

3. Didn’t fill prescription 开了方子，没有去取药 skip ED027 跳过ED027

**ED027.** How much will you eventually pay out of pocket for the medications from this visit, including prescriptions you received? 这次就诊所花的药费(包括在这家医疗机构或其他药店购买医生开的处方药)里，您自己支付了多少钱？

1.________ RMB 元(ED027_1) [softcheck upper limit: 5,000 and ED027_1 must be no more than ED026_1 and no more than ED023_1. 上限：5,000 而且ED027_1 必须少于ED026_1，少于ED023_1]

2. Didn’t pay anything 没有自付费用

**[Show Card 出示卡片14]**

**ED028.** What insurance did you use or will you use?(circle all that apply) 这次就诊的费用（包含诊疗费和药费）您使用或将使用哪种医疗保险报销？（可多选）

1. Urban employee medical insurance (yi-bao) 城镇职工医疗保险（医保）
2. Urban resident medical insurance 城镇居民医疗保险
3. New cooperative medical insurance (he-zuo-yi-liao) 新型农村合作医疗保险（合作医疗）
4. Urban and rural resident medical insurance 城乡居民医疗保险（合并城镇居民和新型农村合作医疗保险）
5. Government medical insurance (gong-fei) 公费医疗
6. Medical aid 医疗救助
7. Private medical Insurance: Purchased by R’s union 商业医疗保险: 单位购买
8. Private medical Insurance: Purchased by Individual 商业医疗保险: 个人购买
9. Other medical insurance (specify) 其他医疗保险,请注明_______
10. Reimbursed by R’s union 单位报销
11. No insurance 没有保险
12. Not revelent to me .不适用

F1：（1）“城镇职工医疗保险”指依法对城镇职工的基本医疗权利给予保障的社会医疗保险制度。

（2）“城镇居民医疗保险”指2007年7月国务院决定在全国79个城市实行城镇居民基本医疗保险试点。参保范围：城镇非从业居民，包括：不属于城镇职工基本医疗保险制度覆盖范围的中小学阶段的学生（包括职业高中、中专、技校学生）、少年儿童和其他非从业城镇居民。

（3）“新型农村合作医疗保险“指由政府组织、引导、支持，农民自愿参加，个人、集体和政府多方筹资，以大病统筹为主的农民医疗互助共济制度。采取个人缴费、集体扶持和政府资助的方式筹集资金。

（4）“城乡居民医疗保险”指一些地区率先将城镇居民医疗保险与新型农村合作医疗合并，推行统一的城乡居民医疗保险制度。

（5）“外来务工人员医疗保险”指在某些地区在城镇职工基本医疗保险以外，专为外来务工人员设置的一种医疗保险制度。

（6）“公费医疗”指国家为保障国家工作人员身体健康而实行的一项社会保障制度。其享受对象主要是各级国家机关、党派、团体以及文化、教育、科研、卫生、体育等事业单位的工作人员和离退休人员，二等乙级以上革命伤残军人以及在校大学生等。

（7）“医疗救助”指在政府的支持下，依靠社会力量建立的旨在向困难群体提供某些或全部基本医疗服务的制度。

（8）“商业医疗保险”是相对于社会保险而言的。商业医疗保险由商业保险公司经办，以营利为目的，企业或职工自愿参加。

（9）“其他医疗保险”如中国职工保险互助会《在职职工重大疾病互助保障计划》和《生育保险》。

（10）“医疗救助”在部分区县试点，救助标准是由各区县根据自身经济情况制定，导致救助标准有高有低。主要三种，一是提供社会医疗救助金，给救助对象以经济补偿；二是给医疗机构一定的经济补贴，使后者直接减免救助对象的部分医疗费；三是由社会医疗救助机构举办专门医疗机构，免费为救助对象提供医疗服务。

（11）“医疗保险”是指基本医疗保险，是为补偿疾病所带来的医疗费用的一种保险。职工因疾病、负伤、生育时，由社会或企业提供必要的医疗服务或物质帮助的社会保险。如中国的公费医疗、劳保医疗。

（12）“单位报销”指一些福利较好的单位可以对本单位职工发生的除基本医疗保险报销部分以外的医疗费用进行全额或一定额度的报销。

**ED029.**  Did you give any “red envelopes” to the doctors for this visit? 您最近一次就诊有没有给医生送红包？

（1）Yes 是

（2）No 否

**The following questions pertain to hospitalization (inpatient care) that you have had during the past year. 下面我们想了解一下您过去一年接受住院治疗的情况。**

**EE001.** In the past year, did a doctor suggest that you needed inpatient care but you did not get hospitalized? 过去一年，是否有医生说您应该住院而没有住院的情况？

1. Yes 有
2. No 没有 Skip to EE003 请跳至EE003

**EE002.** What’s the main reason for not seeking hospitalization? 您没去住院的主要原因是什么？

1. Not enough money 没有钱
2. Not willing to go to the hospital 不愿意住院
3. Felt that hospital was unlikely to cure problem—hospital quality poor 住院也没有用，医院医疗条件差
4. Felt that care was unlikely to cure the problem—problem too serious 觉得问题很严重，住院也没用
5. No ward available. 医院没有床位
6. Other 其他

**EE003.** Have you received inpatient care in the past year? 过去一年内，您住过院吗？

1. Yes 是
2. No 否 Skip to EF001 请跳至EF001

**EE004.**  How many times have you received inpatient care during the past year? 过去一年，您接受过几次住院治疗？└─┴─┘ Times 次 [If EE004 =1, skip to EE007. 如果EE004 =1，跳至EE007]

**EE005.** What was the medical cost for all the hospitalizations you received during the past year? (Only include fees paid to the hospital, including ward fees but excluding wages paid to a hired nurse, transportation costs, and accommodation costs for yourself or family members.) 过去一年住院的总费用大概是多少? （只包括付给医院的费用，不包括陪护的工资、自己或家人的交通费和住宿费，但包括医院病房费。）

A. 1. Total cost 总费用_________(EE005_1)Yuan 元;

2. Didn’t pay anything. 没有付任何钱。[softcheck upper limit 上限: 300,000]

**PROCEDURE: IF EE005 = 1 ASK EE006**

**EE006.**

B. 1. Self-paid part 其中自付部分_________(EE006_1)Yuan 元

2. Didn’t pay anything. 没有付任何钱 [softcheck upper limit: 300,000 and EE005 B.1<=EE005 A.1.上限：300,000，而且EE005 B.1<=EE005 A.1.]

**We want details about the last hospitalization you had in the past year. 我们想了解您在过去一年中，最后一次住院的详细情况。**

**If ED001 = 1 ask EE007. 如果ED001 = 1问EE007.**

**EE007.** Is this the same facility as mentioned in ED011 for outpatient care? 这家医院是[ ED011 ]吗？

(1) Yes 是 Skip to EE016 跳至EE016

(2) No 否

**If EE007= 2 or EC003= 2 ask EE008 -EE015. 如果EE007 = 2 或者EC003 = 2 问EE008-EE015。**

**[Show Card 出示卡片17]**

**EE008.** What is the type of health or service facility which you visited for last inpatient care (hospital admissions)/for your most recent hospitalization in the past year? 过去一年中，您最后一次接受住院治疗所在的医疗机构的类型是什么？

(1) General Hospital 综合医院（即全科医院，不包括中医院）

(2) Specialized hospital 专科医院（不包括中医院）

(3) Chinese Medicine Hospital 中医院

(4) Community Healthcare Center 社区卫生服务中心

(5) Township Hospital 乡镇卫生院

(6) Health care post 卫生服务站

(7) Other 其他

**EE009.** Is this facility public or private? 这家医疗机构是公立的还是私立的？

1. Public 公立的
2. Private 私立的

**If EE008 =1-3, ask EE010, 如果EE008 =1-3，请回答EE010**

**EE010.** What’s the level of this facility? 这家医疗机构是什么级别的？

1. County/district 县/市/区级
2. Regional /city 地/市
3. Provincial/ affiliated to a ministry 省/部属
4. Military 军队
5. Others 其他

**EE011.** What is the name of this facility? 这家医疗机构的名称是什么？____________

**EE012.**. What is the location of this facility? 这家医疗机构的具体的地址是什么？

1. province 省(EE012_1 )

A. This province 本省

B. Other province, (preload province) 外省（选择省份）(EE012_1_1 )

[IWER: Choose from the list of provinces，see appendix 2 访员注意：请选择省份，见附表二]

2. county/city县/市(EE012_2_1 )

A. This county / city 本县/市

B. Other county / city, specify 外县/市 县/市(EE012_2_2 )

3.township/district 乡/区(EE012_3_1 )

A. This township/district 本乡/ 区

B. Other township/district, specify 外乡/区 (EE012_3_2) 乡/区

4. village/street 村/街道(EE012_4_1 )

A. This village/street 本村/街道

B. Other village/street, specify 外村/街道 (EE012_4_2 )

**EE013.** How many kilometers is it from the medical facility to your residence? 从您家到这家医疗机构有几公里？└─┴─┴─┘.└─┴─┘ Km 公里 [softcheck upper limit 上限: 3000]

**EE014.** What is the travel time (one-way) to that facility? 您上次去这家医疗机构接受住院治疗单程花多少时间？

(EE014_1)

└─┴─┘

01. Minute 分钟 [check range 范围: 1-59]

02. Hour 小时 [softcheck upper limit 上限: 20]

(EE014_2)

1 Walk 步行

2 Bus 公交车

3 Car 私家车或出租车

4.Ambulance 救护车

5 Bicycle or other manual vehicles 自行车或其他人力车

6.Electric bicycle/electric tricycle 电动自行车/电动三轮车

7. Motorcycle 摩托车

8. Tractor 拖拉机

9. Train 火车

10. Animal or animal pulled cart 骑牲畜或坐畜力车

**IF EE014_2 = 2-10, ask EE015**

**EE015.** What was the total transportation cost to the facility (including fuel cost, one way trip)? 您上次去这家医疗机构接受住院治疗的单程交通成本是多少钱？（包括燃料费） └─┴─┴─┘,└─┴─┴─┘RMB 元 [soft check upper limit 上限: 600]

**EE016.** How many nights were you hospitalized there? 您在那里住了几晚？

└─┴─┴─┘ Nights 个晚上 [softcheck upper limit 上限: 40]

**EE017.** What was the starting date of your hospital stay? 您从哪一天开始住院？

______1900..2011 (EE017_1)Year 年_________0..12 (EE017_2)Month 月_______0..31 (EE017_3)Day 日

[IWER: Mark the year using four digits. Take down the month as its actual number. For example, write January as “1” not “01”, December as “12”. If do not remember month and day, fill ‘0’. 访员注意: 用4位数表示年，按照实际的月份填写月。例：1月写作“1”，而不是“01”,12月写作“12”。如果记不住月份和日期，请填入’0’。]

**EE018.** What was your date of exit? 您哪天出院？

(1) ______1900..2011 (EE018_1)Year 年_________0..12 (EE018_2)Month 月________0..31(EE018_3)Day

[IWER: Mark the year using four digits. Take down the month as its actual number. For example, write January as “1” not “01”, December as “12”. If do not remember month and day, fill ‘0’. 访员注意: 用4位数表示年，按照实际的月份填写月。例：1月写作“1”，而不是“01”,12月写作“12”。如果记不住月份和日期，请填入’0’。]

(2) Still there 仍然在住院

**[soft check: date of exit should be not before starting date, “exit date is before starting date, please ask R again”, also exit date should be within 1 year of today 出院日期应该比住院日期要晚，“出院日期早于住院日期，请再问一次受访者”，出院日期应该在从今天往前数的一年之内。]**

**EE019.**. Why were you hospitalized? （Choose one choice） 您住院的原因是什么？(单选)

(1) Sickness 生病

(2) Accident 意外

(3) Violence 暴力

(4) Other 其他

**EE020 .** Could you tell me the name of the disease? 您能告诉我们您是得了什么病住院的吗？_________

**[Show Card 出示卡片18]**

**EE021.** During hospitalization, what kind of treatment did you receive? (circle all that apply) 在最近的一次住院期间，您接受了哪些治疗？ (可多选)

[IWER: Read one by one. 访问员注意：请逐项读出以下答案。]

1. Medical check-up/consultation 体检或者咨询
2. Injection 注射
3. Laboratory test 化验
4. Surgery 手术
5. X-ray, CT, B ultrasonic, MRI X-光、CT、B超、核磁共振等检查
6. Medications 用药、拿药
7. IV (Drip Infusion) 输液
8. Traditional treatment, e.g., massage, acupuncture 中医治疗，如按摩、针灸等

(9) Delivery 分娩

(10) Other 其他

**[PROCEDURE: If EE018 =2, please skip EE022 . 程序：如果EE018 =2，请跳过EE022]**

**EE022.** Under what conditions did you leave the hospital? 您这次是在什么情况下出院的？

(1) Fully recovered from illness, received doctor’s approval 病完全好了，医生同意出院 Skip EE023 跳过EE023

(2) Didn’t recover from illness, but received doctor’s suggestion to leave 病没有完全好，医生建议出院 Skip EE023 跳过EE023

(3) Didn’t recover from illness, requested to leave without doctor’s suggestion 病没有完全好，自己要求出院

(4) Other reasons 其他原因 Skip EE023 请跳过EE023

**EE023.** Why did you want to leave the hospital before you were recovered? 您要求提前出院的原因是什么？

(1) Can’t recover from illness 久病不愈

(2) Poor 经济困难

(3) No space in the hospital 没有床位

（4）Limited hospital conditions 医院医疗条件所限

(5) Poor quality and service from health care providers 医护人员服务态度不好

(6) Other reasons 其他原因

**EE024.** What was the total medical cost of hospitalization? (Only include the fees paid to the hospital, excluding the wage of hired nurse, the fare or rent, but including the ward fees.) 这次住院一共花了多少钱？（只包括付给医院的费用，不包括陪护的工资、自己或家人的交通费和住宿费，但包括医院病房费。）

1.└─┴─┴─┘,└─┴─┴─┘,└─┴─┴─┘RMB 元 (EE024_1)[softcheck upper limit 上限: 100,000]

2. There was no cost 没有费用

**EE025.** What was the total cost for hired nurse? 雇佣陪护照顾病人花了多少钱?

1.└─┴─┴─┘,└─┴─┴─┘,└─┴─┴─┘RMB 元(EE025_1)

2. There was no cost 没有费用

**EE026.** What was the total cost for transportation, food and accommodation of patient and relatives? 自己和家人往返医院交通费、伙食费以及家人或陪护的住宿费一共花了多少钱?

1.└─┴─┴─┘,└─┴─┴─┘,└─┴─┴─┘RMB 元(EP026_1)

2. There was no cost 没有费用

**EE027.** How much did you or will you eventually pay out of pocket for the total costs of hospitalization? 这次住院，您最后自己总共付了（将要付）多少钱？

1.└─┴─┴─┘,└─┴─┴─┘,└─┴─┴─┘RMB 元(EE027_1) [softcheck upper limit: 100,000 and EE027_1 must be no more than EE024_1 上限：100,000，而且EE027_1必须小于等于EE024_1 ]

2. Didn’t pay anything. 没有付任何钱

**EE028.** Who contributes most for paying the out-o- pocket cost? 自付部分谁给您交得最多？

1. Myself 自己
2. Children 子女
3. Relatives 亲属
4. Government 政府
5. R’s union 单位（包括村集体）
6. Loan 借款
7. Donate 捐赠
8. Others 其他 Specify 请注明

**EE029.** What was the total medication cost during this visit? 这次住院药费一共花了多少钱？

1.└─┴─┴─┘,└─┴─┴─┘,└─┴─┴─┘ RMB 元(EE029_1) [softcheck upper limit: 60,000 and EE029_1 should be no more than EE024_1上限：60,000，而且EE029_1 应该小于等于EE024_1 ]

2. Didn’t receive 没有用药 Skip EE030 请跳过EE030

**EE030.** How much did you pay out of pocket for medication costs during this visit? 这次住院，您自己一共付了多少钱的药费？

1. └─┴─┴─┘,└─┴─┴─┘,└─┴─┴─┘ RMB 元(EE030_1) [softcheck upper limit: 60,000 and EE030_1 should be no more than EE029_1 and EE024_1 上限：60,000，而且EE031_1 应该小于等于EE029_1 和EE024_1]

2. Didn’t pay anything. 没有付任何钱

**[Show Card 出示卡片14]**

**EE031.** **(Preload from EA001 or EB002)** What insurance did you use or will you use? (circle all that apply)您使用或将使用哪种医疗保险报销？(可多选)

1. Urban employee medical insu rance (yi-bao) 城镇职工医疗保险（医保）
2. Urban resident medical insurance 城镇居民医疗保险
3. New cooperative medical insurance (he-zuo-yi-liao) 新型农村合作医疗保险（合作医疗）
4. Urban and rural resident medical insurance 城乡居民医疗保险（合并城镇居民和新型农村合作医疗保险）
5. Government medical insurance (gong-fei) 公费医疗公费医疗
6. Medical aid 医疗救助
7. Private medical Insurance: Purchased by R’s union 商业医疗保险: 单位购买
8. Private medical Insurance: Purchased by Individual 商业医疗保险: 个人购买
9. Other medical insurance (specify) 其他医疗保险, 请注明_______
10. Reimbursed by R’s union 单位报销
11. No insurance 没有保险
12. Not revelent to me 不适用

F1：（1）“城镇职工医疗保险”指依法对城镇职工的基本医疗权利给予保障的社会医疗保险制度。

（2）“城镇居民医疗保险”指2007年7月国务院决定在全国79个城市实行城镇居民基本医疗保险试点。参保范围：城镇非从业居民，包括：不属于城镇职工基本医疗保险制度覆盖范围的中小学阶段的学生（包括职业高中、中专、技校学生）、少年儿童和其他非从业城镇居民。

（3）“新型农村合作医疗保险“指由政府组织、引导、支持，农民自愿参加，个人、集体和政府多方筹资，以大病统筹为主的农民医疗互助共济制度。采取个人缴费、集体扶持和政府资助的方式筹集资金。

（4）“城乡居民医疗保险”指一些地区率先将城镇居民医疗保险与新型农村合作医疗合并，推行统一的城乡居民医疗保险制度。

（5）“外来务工人员医疗保险”指在某些地区在城镇职工基本医疗保险以外，专为外来务工人员设置的一种医疗保险制度。

（6）“公费医疗”指国家为保障国家工作人员身体健康而实行的一项社会保障制度。其享受对象主要是各级国家机关、党派、团体以及文化、教育、科研、卫生、体育等事业单位的工作人员和离退休人员，二等乙级以上革命伤残军人以及在校大学生等。

（7）“医疗救助”指在政府的支持下，依靠社会力量建立的旨在向困难群体提供某些或全部基本医疗服务的制度。

（8）“商业医疗保险”是相对于社会保险而言的。商业医疗保险由商业保险公司经办，以营利为目的，企业或职工自愿参加。

（8）“其他医疗保险”如中国职工保险互助会《在职职工重大疾病互助保障计划》和《生育保险》。

（10）“医疗救助”在部分区县试点，救助标准是由各区县根据自身经济情况制定，导致救助标准有高有低。主要三种，一是提供社会医疗救助金，给救助对象以经济补偿；二是给医疗机构一定的经济补贴，使后者直接减免救助对象的部分医疗费；三是由社会医疗救助机构举办专门医疗机构，免费为救助对象提供医疗服务。

（11）“医疗保险”是指基本医疗保险，是为补偿疾病所带来的医疗费用的一种保险。职工因疾病、负伤、生育时，由社会或企业提供必要的医疗服务或物质帮助的社会保险。如中国的公费医疗、劳保医疗。

（12）“单位报销”指一些福利较好的单位可以对本单位职工发生的除基本医疗保险报销部分以外的医疗费用进行全额或一定额度的报销。

**EE032.** Did you pay any “Red Envelopes” to the doctors for this visit? 这次住院期间有没有给医生送红包？

(1) Yes 是

(2) No 否 .

**Now we’d like to know whether you have treated yourself during the past month. 下面我们想了解在过去一个月，您进行自我治疗的情况。**

**[Show Card 出示卡片19]**

**EF001.** How did you treat yourself during the past month? (circle all that apply) 过去一个月，你是否进行了以下自我治疗（注意：此处不包括凭处方取药的情况）？（可多选） [check, if choose 7 cannot choose other options 如果选择7，就不能选其他选项。]

1. Consumed over-the-counter modern medicines 自己买非处方西药
2. Consumed prescription medicines自己买处方西药
3. Consumed traditional herbs or traditional medicines as treatment 用传统中草药或者传统方法治疗
4. Tonic/Health supplement 吃维生素/补品
5. Use health care equipment 使用保健设备
6. Other 其他
7. None 没有 Skip to EF006 跳至EF006

**For each circled self-treatment method (1-5), ask questions EF002 , EF003 , EF005 对每一种选择的 1-5 的自我治疗方式，均回答EF002 , EF003 , EF005**

**EF002.** What is the approximate total cost to [preload EF001 ] during the last month? 过去一个月，自己[preload EF001 ]的总成本大概是多少？

1.└─┘,└─┴─┴─┘,└─┴─┴─┘ RMB 元(EF002_1) [softcheck upper limits: 2,000 上限检查：2,000]

2. There was no cost. 没有费用。

**PROCEDURE: IF EF002 = 1 ASK EF003**

**EF003.** How much did you pay out-of-pocket? 除了报销的部分，您自己支付了多少？

1.└─┘,└─┴─┴─┘,└─┴─┴─┘ RMB 元(EF003_1) [softchecks upper limits: 2,000 and EF003_1 should be no more than EF002_1 . 上限检查：2,000，而且EF003_1 应该小于等于EF002_1 ]

2. Didn’t pay anything. 没有付任何钱 Go to EF005 请跳至EF005

**EF004.** Who contribute most for paying the out-o- pocket cost? 自付部分谁给您交得最多？

1. Myself 自己
2. Children 子女
3. Relatives 亲属
4. Government 政府
5. R’s union 单位（包括村集体）
6. Loan 借款
7. Donate 捐赠
8. Others 其他 Specify 请注明

**[Show Card 出示卡片14]**

**EF005.** What insurance did you use? (circle all that apply) 您使用了哪种医疗保险报销？（可多选）

1. Urban employee medical insurance (yi-bao) 城镇职工医疗保险（医保）
2. Urban resident medical insurance 城镇居民医疗保险
3. New cooperative medical insurance (he-zuo-yi-liao) 新型农村合作医疗保险（合作医疗）
4. Urban and rural resident medical insurance 城乡居民医疗保险（合并城镇居民和新型农村合作医疗保险）
5. Government medical insurance (gong-fei) 公费医疗公费医疗
6. Medical aid 医疗救助
7. Private medical Insurance: Purchased by R’s union 商业医疗保险: 单位购买
8. Private medical Insurance: Purchased by Individual 商业医疗保险: 个人购买
9. Other medical insurance (specify) 其他医疗保险, 请注明_______
10. Reimbursed by R’s union 单位报销
11. No insurance 没有保险
12. Not revelent to me .不适用

F1：（1）“城镇职工医疗保险”指依法对城镇职工的基本医疗权利给予保障的社会医疗保险制度。

（2）“城镇居民医疗保险”指2007年7月国务院决定在全国79个城市实行城镇居民基本医疗保险试点。参保范围：城镇非从业居民，包括：不属于城镇职工基本医疗保险制度覆盖范围的中小学阶段的学生（包括职业高中、中专、技校学生）、少年儿童和其他非从业城镇居民。

（3）“新型农村合作医疗保险“指由政府组织、引导、支持，农民自愿参加，个人、集体和政府多方筹资，以大病统筹为主的农民医疗互助共济制度。采取个人缴费、集体扶持和政府资助的方式筹集资金。

（4）“城乡居民医疗保险”指一些地区率先将城镇居民医疗保险与新型农村合作医疗合并，推行统一的城乡居民医疗保险制度。

（5）“外来务工人员医疗保险”指在某些地区在城镇职工基本医疗保险以外，专为外来务工人员设置的一种医疗保险制度。

（6）“公费医疗”指国家为保障国家工作人员身体健康而实行的一项社会保障制度。其享受对象主要是各级国家机关、党派、团体以及文化、教育、科研、卫生、体育等事业单位的工作人员和离退休人员，二等乙级以上革命伤残军人以及在校大学生等。

（7）“医疗救助”指在政府的支持下，依靠社会力量建立的旨在向困难群体提供某些或全部基本医疗服务的制度。

（8）“单位报销”指一些福利较好的单位可以对本单位职工发生的除基本医疗保险报销部分以外的医疗费用进行全额或一定额度的报销。

（9）“商业医疗保险”是相对于社会保险而言的。商业医疗保险由商业保险公司经办，以营利为目的，企业或职工自愿参加。

（10）“其他医疗保险”如中国职工保险互助会《在职职工重大疾病互助保障计划》和《生育保险》。

（11）“医疗救助”在部分区县试点，救助标准是由各区县根据自身经济情况制定，导致救助标准有高有低。主要三种，一是提供社会医疗救助金，给救助对象以经济补偿；二是给医疗机构一定的经济补贴，使后者直接减免救助对象的部分医疗费；三是由社会医疗救助机构举办专门医疗机构，免费为救助对象提供医疗服务。

（12）“医疗保险”是指基本医疗保险，是为补偿疾病所带来的医疗费用的一种保险。职工因疾病、负伤、生育时，由社会或企业提供必要的医疗服务或物质帮助的社会保险。如中国的公费医疗、劳保医疗。

（14）“单位报销”指一些福利较好的单位可以对本单位职工发生的除基本医疗保险报销部分以外的医疗费用进行全额或一定额度的报销。

**EF006.** How often did the respondent receive assistance in answering section D-Health care and insurance?受访者填写该部分问卷时是否求助？

[IWER: If it is answered by a proxy, the respondent’s reaction. 访员注意：如果是协助回答，请记录受访者的反应。]

1. Never 从未
2. A few times 偶尔几次
3. Most or all of the time 大多数
4. The section was completed by a proxy respondent（the respondent is absent） 受访者不在场，完全请人代填 → Skip to EF007 请跳至EFA007

**EF007.** What is your relationship to R? 您和受访者是什么关系？

[IWER: What is the proxy’s relationship to R? If unknown, please ask the proxy. 访员注意：代填问卷的人和受访者是什么关系。如果不清楚，请问代填者。]

1. Spouse 配偶
2. Mother 母亲
3. Father 父亲
4. Mother-in-law 岳母/婆婆
5. Father-in-law 岳父/公公
6. Sibling 兄弟姐妹
7. Brother-in-law, sister-in-law 姐夫妹夫/嫂子弟媳
8. Child 孩子
9. Spouse of child 孩子的配偶
10. Grandchild 孙子女
11. Other relative 其他亲戚
12. Helper or other non-relative 帮忙的人或者其他非亲属

**EF008.** [IWER: Please record the reason for proxy 访员注意：请记录代答的原因。]

What is the main reason for proxy（the respondent is absent）？受访者不在场，完全请人代填的主要原因是什么？

（1）The respondent has serious physical handicaps 受访者有严重身体障碍

（2）The respondent has serious mental handicaps,受访者有严重精神障碍

（3）The respondent has rejected this interview. 受访者拒访

（4）Other . 其他 (EF008_1)

# **F WORK, RETIREMENT AND PENSION 工作、退休和养老金**

## FA JOB STATUS 就业状况

**FA001.** Did you engage in agricultural work (including farming, forestry, fishing, and husbandry for your own family or others) for more than 10 days in the past year? 过去一年，您有没有从事10天以上的农业生产经营活动（包括为自家或其他农户种地、管理果树、采集农林产品、养鱼、打鱼、养牲畜以及去市场销售自家生产的农产品等）？

1. Yes 是 → Skip to FB001 请跳至FB001
2. No 否

**FA002.** Did you work for at least one hour last week? We consider any of the following activities to be work: earn a wage, run your own business and unpaid family business work, et. al. Work does not include doing your own housework or doing activities without pay, such as voluntary work. 上周您工作了至少一个小时吗？挣工资工作、从事个体、私营经济活动或不拿工资为家庭经营活动帮工都算是工作，但不包括家务劳动、义务的志愿劳动。

1. Yes 是 → Skip to FB001 请跳至FB001
2. No 否

**FA003 .** Do you have a job but are temporarily laid-off, or on sick or other leave, or in-job training? 您是不是有工作但是目前正处在临时放假、休病假，或其他假期中，或者正在在职培训？

1. Yes 是
2. No 否 → Skip to FA007 请跳至FA007

**FA004.** In what month and year did you leave or attend training? 您是什么时候开始休假或参加培训的？

______1900..2011 (FA004_1) Year年_______0..12 (FA004_2) Month 月

[IWER: Mark the year using four digits. Take down the month as its actual number. For example, write January as “1” not “01”, December as “12”. If do not remember month, fill ‘0’. 访员注意: 用4位数表示年，按照实际的月份填写月。例：1月写作“1”，而不是“01”,12月写作“12”。如果记不住月份，请填入’0’。]

[Double check those stop work for a long time. (Interview Year – FA004_1 ) + (Interview month – FA004_2 )/12 > 1?]

**FA005.** Do you expect to go back to this job at a definite time in the future or within 6 months? 您能够在确定的时间或者6个月以内，回到原来的工作岗位么？

1. Yes 是 → Skip to FB001 请跳至FB001
2. No 否

**FA006.** Do you still receive any salary from this job? 这个单位是否仍然给你发工资?

(1) Yes 是 →Skip to FB001 请跳至FB001

(2) No 否

**FA007.** Have you worked for at least three months during your lifetime (work includes agricultural work,earning wage work, self-employed activities, and unpaid family business work, et. al.)? 您这辈子是否曾经工作了至少三个月的时间？(工作包括务农、挣工资工作、从事个体、私营活动或不拿工资为家庭经营活动帮工等)

1. Yes 是  →Skip to FB001 请跳至FB001
2. No 否

**FA008.** Work includes all kinds of labour excluding doing your own housework, whether you earn wages or not. Are you sure that you didn’t work at least three months during your lifetime? 我们所说的工作指所有除家务劳动以外的所有干活的劳动，不管是不是有工资。您确定这辈子没有工作过至少三个月的时间？

- - 1. Yes, never worked before 是的，从未工作过
    2. No, ever worked. 不是，以前工作过 →Skip to FB001 请跳至FB001

**FA009.** What is the main reason for you not to work in your lifetime? 请问您不工作的主要原因是什么？

- - - - 1. Disabled (physical or psychological) 残疾（包括生理和心理上的残疾）
        2. Homemaker 操持家务
        3. My family is too rich that I don’t need to work 我家太有钱了，我不需要工作
        4. Taking care of siblings 照看兄弟姐妹
        5. Other 其它

**[PROCEDURE: If FA007 = 2[never worked], then go to FN001 [pension section]. 如果受访者回答从未工作FA007 = 2，请跳至养老保险部分FN001]**

## FB WORK HISTORY 工作史

**FB001.** At what age (or in which year) did you start working (or farming), excluding part-time job during school time? 您从多少岁或者哪年开始工作(或者务农)的（不包括上学期间的兼职工作）？

Age 年龄___ 1…120 (FB001_1)Years岁or或 ___1900..2011 (FB001_1) Year年

[IWER: Mark the year in four digits.访员注意：用4位数表示年。]

**FB002.** What type of work unit was your first job, A government organization, “shiye danwei,” firm, NGO, inidivdual farmer, or household enterprise? 您第一份工作的工作单位属于以下哪一种？

1. Government政府部门
2. Institutions 事业单位
3. NGO 非营利机构（如社团、协会、学会等）
4. Firm 企业
5. Individual firm 个体户
6. Farmer 农户
7. Individual household 居民户
8. Other 其他

**[PROCEDURE: If FB002 = 4, then ask FB003, otherwise go on to FB004 . 如果FB002 =4，请提问FB003，否则请跳至FB004]**

**[Show Card 出示卡片20]**

**FB003.** What is the ownership type of the business? 您单位的所有制类型是？

1. 100% State owned firm 国有企业
2. State-controlled firm国有控股企业
3. 100% Collective-owned firm 集体所有制企业
4. Collective-controlled firm集体控股企业
5. 100% Private firm 私营/个体
6. Private-controlled firm私人控股企业
7. 100% foreign-owned 外商独资
8. Joint venture中外合资
9. Other joint-ownership 其他联营企业
10. Other 其他

**FB004.** Where was your first workplace located? 您第一份工作的地点在哪里？

- 1. this village/community 本村/社区
  2. other village/community in this county/city 本县/市的其他村/社区

(3)Another county/city in this province在本省的其他县/市，________ (FB004_1)city市_______ (FB004_2) county县

(4)Another province 外省：________(FB004_3)province 省/自治区/直辖市，___________(FB004_4)city市__________(FB004_5) county县00000000000000000000000000000000000000000000000000000000000000000000000000000000000000000000000000000000000000000000000000000000 136___别是？0000000000000000000000000000000000000000000000000000000000000000000000000000000000000000000000000000000000000000000000

[IWER: Mark 0 if there is no fixed address. 访员注意：如果没有具体地址，请标明“0”。]

[IWER: Choose from the list of provinces，see appendix 2. 访员注意：请选择省份，见附表二]

**[PROCEDURE: If FB002 = 4 and FB003= 1/ 2/3/4, then skip FB005 and FB006. 如果FB002= 4而且FB003 =1/ 2/3/4, 跳过FB005和FB006 .]**

**FB005.** Over your career did you ever have employment in a state owned, state controlled or collectively owned enterprise? 您一生中有没有在国有企业、国有控股企业、集体所有制企业或集体控股企业工作过？

1. Yes 有
2. No 没有 Go to FB007 请跳至FB007

**FB006.** Specific ownership type 请问是在哪种类型的企业工作过？

1. 100% State owned firm 国有企业
2. State-controlled firm国有控股企业
3. 100% Collective-owned firm 集体所有制企业
4. Collective-controlled firm集体控股企业

**FB007.** Not counting current non-employment or retirement, did you stop working for an extended period of time （more than 1 year once）due to reasons of family, health, school, etc.? 您是否曾经因为家庭、健康、上学等原因中断过工作（一次中断时间在1年以上），服兵役、灵活就业、退休后不工作或者目前失业的时间不算？

1. Yes 是
2. No 否 →Skip to FB011 请跳至FB011

**FB008.** How long were the interruptions in all? 一共中断了多长时间？

____ 1…120 (FB008_1) Years 年___0..11(FB008_2)Months 月

[IWER: Mark the month and year using integert; if 0, please fill ‘0’. 访员注意：用整数表示年月，如果刚好是整年，月份处填“0”。 ]

**FB009** When was the longest time period that you stopped working? 最长的一次中断是从什么时候到什么时候？From从____ 1900..2011 (FB009_2)year年____ 0..12 (FB009_3)month to月到____ 1900..2011 (FB009_5)year年___0..12 (FB009_6)month月

[IWER: Mark the year using four digits. Take down the month as its actual number. For example, write January as “1” not “01”, December as “12”. If do not remember month, fill ‘0’. 访员注意: 用4位数表示年，按照实际的月份填写月。例：1月写作“1”，而不是“01”,12月写作“12”。如果记不住月份，请填入’0’。]

**[Soft Check: Reprompt for FB008 and FB009 if longest time period is greater than Total from FB008 or (FB009_3 +FB009_4 /12)-(FB009_1 +FB009_2 /12) > FB008_1 +FB008_2 /12.]**

**[Another check to avoid that interruption began before starting work. FB009_1 < FB001_1 or FB009_1 < CV009_a + FB001_1 ]**

**FB010.** The reason was: 中断工作的原因是：

(1) Family 家庭（例如照顾家人，结婚生子）

(2) Health 健康（例如生病）

(4) School 上学

(5) Unemployment/layoff 失业/下岗

(6) Other 其他

**FB011.** Have you completed retirement procedures （including early retirement） or internal retirement（Note: Retirement from government departments, enterprises and institutions, not including retirement in the sense of getting agricultural insurance）? 您是否办理了退休手续（包括提前退休）或内退（注意：退休指从机关、事业单位、企业的退休，不包括获取农保的退休）？

(1) Yes 是 → skip FB012 跳过FB012

(2) No 否

**FB012.** Have you completed receding position procedures？您是否办理了退职手续？

(1) Yes 是

(2) No 否

F1：“退职”指职工因病残完全丧失劳动能力，但在年龄、工龄或个人缴费年限方面又不具备退休条件的，经医院证明并经劳动鉴定委员会确认、组织批准后退出生产或工作岗位，并按国家有关规定给予一定的物质帮助和被补偿，进行休养。或者是已到退休年龄，但工龄不够。

**[PROCEDURE: If FA007=1, please skip to FK001. 程序：如果FA007 =1，跳至FK001.]**

**[PROCEDURE: If FA008=2, please skip to FL001. 程序：如果FA008 =2，跳至FL001.]**

## FC CURRENT PRIMARY JOB/OCCUPATION 当前、主要工作

**SKIP PATTERN: If R did farming last year (FA001=1), ask FC001; if R did not do farming last year, but worked last week (FA001 =2 and (FA002 =1 OR [FA002 =2 & FA003 =1 & FA005 =1]), ask FC019.**

**如果受访者过去一年从事了农业劳动：请回答FC001；若受访者有非农就业，不管其过去一周是否工作（FA001 =2 and (FA002 =1 OR [FA002 =2 & FA003 =1 & FA005 =1]），都跳至FC019 。**

**FARM ENPLOYED 农业打工**

**FC001.** Did you work for other famers in wage for at least ten days in the past year？（Agricultural work in wages）过去一年，您有没有为其他农户打工至少10天？（指从事农业劳动挣钱）

1. Yes 是
2. No 否 →Skip to FC008 请跳至FC008

F1：“为其他农户打工”指为其他农户从事农业生产劳动挣钱。

**FC002.** How many employers did you work for in the past month? 过去一个月，您一共为多少个农户打工？

_________________

**FC003.** Where is your workplace for most time?  大多数时间里，您工作的地点是？

(1)this village/community 本村/社区

(2)other village/community in this county/city 本县/市的其他村/社区

(3)Another county/city in this province在本省的其他县/市，_______ (FC003_1)city市________ (FC003_2)county县

(4)Another province 外省：________(FC003_3)province 省/自治区/直辖市，___________(FC003_4)city市___________(FC003_5) county县00000000000000000000000000000000000000000000000000000000000000000000000000000000000000000000000000000000000000000000000000000000 136___别是？0000000000000000000000000000000000000000000000000000000000000000000000000000000000000000000000000000000000000000000000

[IWER: Mark 0 if there is no fixed address. 如果没有具体地址，请标明“0”。]

[IWER: Choose from the list of provinces，see appendix 2. 请选择省份，见附表二]

**FC004.** How many months did you work on cropping (forestry), livestock, and fishing in wage for other famers in the past year? 过去一年中，您有几个月在为其他农户从事[ 种植业及林业（包括种地、管理果树、采集农林产品）]或[畜牧业(饲养牲畜) ]或 [渔业（养殖水产品）]？ _____ 0..12Months 月 挣钱？

**FC005.** How many days did you work in wage for other famers per week on average during a normal work month in the past year? 过去一年中，在您为其他农户从事[种植业及林业（包括种地、管理果树、采集农林产品）] 或[畜牧业(饲养牲畜) ]或[渔业（养殖水产品）]的月份里，您一般每周干几天？ ______ 0..7Days 天

**FC006.** How many hours did you usually work in wage for other famers per day during a normal work day in the past year? 过去一年中，在您为其他农户从事[种植业及林业（包括种地、管理果树、采集农林产品）] 或 [畜牧业(饲养牲畜) ] 或 [渔业（养殖水产品）]的日子里，您一般每天要干几个小时？ _____ 0..16 Hours 小时

[Soft Check: Verify if number of hours per day is unreasonable, e.g., FC006 >16]

**FC007.** What is the average monthly wage did you get in your working months in the past year? 过去一年里，您工作的月份里平均每个月挣多少钱？___ Yuan 元

**HOUSEHOLD AGRICULTURAL WORK 自家农业生产活动**

**FC008.** Did you work for your own household for at least ten days in the past year？过去一年，您有没有为自家从事农业生产经营活动至少10天？

（1）Yes 是

（2）No 否 →Skip to FC013 请跳至FC013

**FC009.** How many months did you work on [cropping (forestry), livestock, and fishing] for your own householdin the past year? 过去一年中，您有几个月为自家从事[种植业及林业（包括种地、管理果树、采集农林产品）]或[畜牧业(饲养牲畜) ] 或 [渔业（养殖水产品）]？ _____ 0..12Months 月

**FC010.** How many days did you work for your own household per week on average during a normal work month in the past year? 过去一年中，在您为自家从事[种植业及林业（包括种地、管理果树、采集农林产品）] 或[畜牧业(饲养牲畜) ] 或[渔业（养殖水产品）]的月份里，您一般每周干几天？ _____0..7 Days 天

**FC011.** How many hours did you usually work for your own household per day during a normal work day in the past year? 过去一年中，在您为自家从事[种植业及林业（包括种地、管理果树、采集农林产品）] 或 [畜牧业(饲养牲畜) ]或 [渔业（养殖水产品）]的日子里，您一般每天要干几个小时？ ______ 0..24Hours 小时

[ A check similar to the one for EP062. FC011 > 16]

**FC012.** Where is your workplace for most time?  大多数时间里，您工作的地点是？(preload sampling community ID)

(1)this village/community 本村/社区

(2)other village/community in this county/city 本县/市的其他村/社区

(3)Another county/city in this province在本省的其他县/市,_______ (FC012_1)city市______(FC012_2)county县

(4)Another province 外省：_______(FC012_3)province 省/自治区/直辖市，__________(FC012_4)city市________(FC012_5)county县00000000000000000000000000000000000000000000000000000000000000000000000000000000000000000000000000000000000000000000000000000000 136___别是？0000000000000000000000000000000000000000000000000000000000000000000000000000000000000000000000000000000000000000000000

[IWER: Mark 0 if there is no fixed address. 如果没有具体地址，请标明“0”。]

[IWER: Choose from the list of provinces，see appendix 2. 请选择省份，见附表二]

**FC013.** How many days of work did you miss last year due to health problems? 过去一年您有多少天因为健康原因干不了活？______ 0..366 Days 天

[IWER: Mark 0 if you didn't miss any work days. 访员注意：如果没有，请注明“0”。]

**FC014.** Besides agricultural work, did you work for at least one hour last week in wage or self-employed work or umpaid family business? 除农业生产经营活动外，上周您有没有工作（包括挣工资工作、从事个体、私营活动或不拿工资为家庭经营活动帮工等）至少一个小时？

（1）Yes 是 →Skip to FC019 请跳至FC019

（2）No 否

**FC015.** Do you have wage or self-employed work but are temporarily laid-off or are on sick, seasonal, or other leave or in-job training? 您是否有其他工作（包括挣工资工作、从事个体、私营活动或不拿工资为家庭经营活动帮工等），但是目前正暂时放假、由于生病或者季节原因休假、歇业或者处于其他假期中或正在在职培训？

(1) Yes 是

(2) No 否 →Skip to FC018 请跳至FC018

**FC016.** In what month and year did leave or attend training? 您是什么时候开始休假/歇业/在职培训的？

______1900..2011 (FC016_1) Year年_______0..12 (FC016_2) Month 月

[IWER: Mark the year using four digits. Take down the month as its actual number. For example, write January as “1” not “01”, December as “12”. If do not remember month, fill ‘0’. 访员注意: 用4位数表示年，按照实际的月份填写月。例：1月写作“1”，而不是“01”,12月写作“12”。如果记不住月份，请填入’0’。]

**[Same check as the one for FA004 . (Interview Year –FC016_1 + (Interview month – FC016_2 )/12 > 1?]**

**FC017.** Do you expect to go back to this job at a definite time in the future or within 6 months? 您能够在确定的时间或者6个月以内，回到原来的工作岗位么？

1. Yes 是 →Skip to FC019 请跳至FC019
2. No 否

**FC018.** At what age do you plan to stop working? Stopping work in this context shall refer to having stopped all income-related activities, unpaid family business and having no intention of engaging in anything more serious than small pastime work. 您计划在多大年龄时停止工作，即停止从事一切以挣钱为目的的活动，也不再为家庭经营活动帮工，将来也不打算从事比消遣性工作更劳累的工作?______ 1…120Years old 岁

[IWER: Please tell me the approximate age. Mark 0 if you plan to keep working until you are physically able. 访员注意：请告知大概年龄。如果您计划只要健康允许，就一直工作，请标明“0”。]

F1：“消遣性工作”指不是以挣钱为目的的活动，以及不再为家庭经营活动帮工的工作。

→Skip to pension and social security section (FN001) if have not processed retirement and receding (FB011 ==2 and FB012 ==2); or skip to retirement section (FM001) if processed retirement or receding (FB011 ==1 or FB012 ==1)如果还没办理退休手续也没有办理退职手续（FB011 ==2 和 FB012 ==2），请跳至社会保障和养老金部分FN001 ；如果已经办理了退休手续或退职手续(FB011 ==1 或 FB012 ==1)，请跳至退休和退职部分FM001

**FC019.** Besides agricultural work, do you currently hold more than one non-agricultural job? 除农业生产经营外，您目前有两份（及以上）的非农业工作吗？

[IWER: non-agricultural job includes paid jobs, self-employed activities, unpaid family business work, et. al. Activities without pay, such as voluntary work, are not included. 访员注意：工作包括挣工资工作、从事个体、私营活动或不拿工资为家庭经营活动帮工等，但没有工资的比如志愿者之类的工作不包括在内；此处务农不算工作，即如果一个人除了务农外只有一份工作，此处选2。]

(1) Yes 是 Skip to FC020 跳至FC020

(2) No 否 Skip to FC021 跳至FC021

**FC020.** Among all your jobs, which one is your main job? [Main job is defined as the job at which you work the longest hours] Do you earn a wage or do you run your own business or work for unpaid family business? 在这几份工作中，您目前的主要工作[主要工作的定义：工作时间最长的工作]是什么？是挣工资工作，从事个体或者私营经济活动，还是不拿工资为家庭经营活动帮工？

(1) Employed 挣工资工作 →Skip to FD001 请跳至FD001

(2) Self-employed从事个体或者私营经济活动 →Skip to FH001 请跳至FH001

(3) unpaid family business不拿工资为家庭经营活动帮工 →Skip to FH001 请跳至FH001

**FC021.** How do you describe your non-agricultural job? Do you earn a wage or do you run your own business or work for unpaid family business? 您怎么描述您的这份工作？是挣工资工作，从事个体或者私营经济活动还是不拿工资为家庭经营活动帮工？

(1) Employed 挣工资工作 →Skip to FD001 请跳至FD001

(2) Self-employed从事个体或者私营经济活动 →Skip to FH001 请跳至FH001

(3) unpaid family business不拿工资为家庭经营活动帮工 →Skip to FH001 请跳至FH001

### FD EMPLOYED 受雇

**FD001.** Do you receive wages from your current workplace or receive them from a dispatch/contract company? 您的工资是从工作单位直接拿到还是要通过某个派遣单位拿？

1. Place of work 工作单位
2. Labor dispatch company 劳务派遣单位

F1：“工作单位”是指受访者当前从事劳动的单位。“派遣单位”是指劳动者签订合同，或者人事所在的单位，比如劳务输出公司。

[CAPI: For dispatched/contract workers (FD001 =2), mention the following for questions FD002 -FD016 对派遣工人(FD001=2)，对FD002-FD016均提示] The next few questions pertain to the situation at your current workplace, and not to the company that has dispatched/contracted you out. 下面几个问题是关于您为之工作的单位的情况，而不是派遣单位。

**FD002.** Do you work for a government organization, institution, firm, NGO, individual farmer, or resident household? 您是为政府机构、事业单位、企业单位、非赢利结构（如社团、协会、学会等）、个体户、农户，还是居民户工作？

(1) Government政府部门

(2) Institutions 事业单位 → Skip FD006 跳过FD006

(3) NGO 非营利机构（如社团、协会、学会等） → Skip FD006 , FD010 跳过FD006 , FD010

(4) Firm 企业 → Skip FD006 跳过FD006

(5) Individual firm 个体户 → Skip FD006 , FD010 跳过FD006 , FD010

(6) Farmer 农户 → Skip FD005 ,FD006 , FD010 跳过FD005 ,FD006 ,FD010

(7) Individual household 居民户 → Skip FD005 ,FD006 , FD010 跳过FD005 ,FD006 , FD010

(8) Other 其他 → Skip FD006 , FD010 跳过FD006 , FD010

**[INTRO: We will ask your work history later.In order to distinguish the work units, we need the full name and address of your employer.我们在后面的问卷还会问有关工作史的问题，为了便于区分不同的工作单位，我们需要单位名称。]**

**FD003.** What is the name of your workplace/employer? Please state specifically the name of your company or institution. 您的工作单位或者雇主是什么名称？（请标明单位名称）__________________________

[IWER: Write the name of the household head if R works for a family. 访员注意：如果雇主是家庭，请写户主的姓名。]

**FD004.** Where is your workplace located?  您工作单位的地点是？(preload sampling community ID)

(1)this village/community 本村/社区

(2)other village/community in this county/city 本县/市的其他村/社区

(3)Another county/city in this province在本省的其他县/市，___________(FD004_1)city市___________(FD004_2) county县

(4)Another province 外省：________(FD004_3)province 省/自治区/直辖市，_______ (FD004_4)city市_____ ______ (FD004_5) county县00000000000000000000000000000000000000000000000000000000000000000000000000000000000000000000000000000000000000000000000000000000 136___别是？0000000000000000000000000000000000000000000000000000000000000000000000000000000000000000000000000000000000000000000000

[IWER: Mark 0 if there is no fixed address. 访员注意：如果没有具体地址，请标明“0”。]

[IWER: Choose from the list of provinces，see appendix 2. 访员注意：请选择省份，见附表二]

**FD005.** What kind of business or industry do you work in—that is, what does your workplace primarily make or do? 该单位主要是做什么的，也就是，他们制造什么产品或者从事什么活动？_________________________________

[IWER: Type of business访员注意：产业或行业类型]

**If government employee (FD002 =1), ask: 如果是政府雇员，问：**

**FD006.** Are you a civil servant? 您是公务员吗？

1. Yes 是
2. No 否

**FD007.** Are you a formal employee of an establishment? 您是正式编制内的员工吗？

1. Yes 是
2. No 否

**Skip to FD011 请跳至FD011**

**If institution (FD002 =2), ask: 如果是事业单位，问：**

**FD008.** Is your institution operated as a firm or as a government unit? 您这个单位是比照企业还是政府机构运行？

- 1. As a firm 比照企业
  2. As a government unit 比照政府机构

**FD009.** Are you regular worker? 您是正式编制内的员工吗？

1. Yes 是
2. No 否

**If firm (FD002=4), ask: 如果是企业，问：**

**[Show Card 出示卡片20]**

**FD010.** What is the ownership type of the business? 您单位的所有制类型是？

(1) 100% State owned firm 国有企业

(2) State-controlled firm 国有控股企业

(3) 100% Collective-owned firm 集体所有制企业

(4) Collective-controlled firm 集体控股企业

(5) 100% Private firm 私营/个体

(6) Private-controlled firm私人控股企业

(7) 100% foreign-owned 外商独资

(8) Joint venture中外合资

(9) Other joint-ownership 其他联营企业

(10) Other 其他

**FD011.** When did you start working for this employer? 您什么时候开始在这个单位工作的？

______1900..2011 (FD011_1)Year年_______ 0..12 (FD011_2 )Month 月

[IWER: Mark the year using four digits. Take down the month as its actual number. For example, write January as “1” not “01”, December as “12”. If do not remember month, fill ‘0’. 访员注意: 用4位数表示年，按照实际的月份填写月。例：1月写作“1”，而不是“01”,12月写作“12”。如果记不住月份，请填入’0’。]

**[Soft Check: Consistency of employment start date. Make Sure that the individual was at least a minimum age when he/she started working for this employer (e.g., prompt to check if under 16?). Specifically (FD011_1+ FD011_2 /12)- (CV009_a+ CV009_b/12) <16 prompts a soft check.]**

**[Replace 16 in the above soft check with FB001. (FD011_1+ FD011_2 /12)- (CV009_a+ CV009_b/12) < FB001_1 or FD011_1 <FB001_2 ]**

**FD012.** What sort of work do you do? (E.g., cleaner, accountant, teacher, etc.) 您具体从事什么工作？(如清洁工、会计、教师等) ______ _____________________________

[IWER: Ask about the specific work that R does. 访问员注意：需要了解具体的工作内容]

**FD013.** What is your current position? 您目前的职务是什么？

1. Clerk/worker 普通职工
2. Team Leader 组长（股长）
3. Section Chief科长

(4) Director of a division 处长

(5) Director-General of a bureau and above 局长及以上

(6) Village Leader 村干部

(7) Township Leader 乡镇干部

(8) Division manager单位部门经理

(9) Overall/General manager单位总经理

(10) Others 其他

**FD014.** What is your current professional/technical level? 你目前的专业/技术职称是什么？

(1) Technician 技术员

(2) Primary level 初级职称

(3) Intermediate level 中级职称

(4) Advanced level 高级职称

(5) No professional/technical level 无职称

F1：“专业/技术职称”指专业技术人员的专业技术与学识水平和工作能力的等级称号。

**[PROCEDURE:Skip this question if answer to FD013 is 2-9. 程序：如果FD013 选择2-9，跳过这个问题。]**

**FD015.** Are you in a position to supervise others? 您是否管理别人？

1. Yes 是
2. No 否                      → Skip to FD017 请跳至FD017

**FD016.** How many people are there under your supervision? 您管着多少人？

(1) 1~5 people 1~5人

(2) 6~10 people 6~10人

(3) 11~15 people 11~15人

(4) 16~30 people 16~30人

(5) 31~99 people 31~99人

(6) More than 100 people 超过100人

[CAPI: For dispatched worker(FD001 =2), prompt for FD017 -FD030 对派遣工人(FD001 =2)，对FD017 -FD030 均提示]

**The next few questions are about your dispatch work unit.下面几个问题是关于您派遣单位的情况。**

**[PROCEDURE:Do not ask FD017 to FD019 if FD013 =3-5 （government officials）. 程序：如果FD013 =3-5，跳过FD017 , FD018 , FD019]**

**FD017.** What is your employment type at your current workplace? 您目前的受聘类型是？

1. Regular worker 劳务派遣工
2. Contract worker 合同制员工
3. Casual/Part-time worker 日工/钟点工

**FD018.** Do you have a personnel file? 您有档案吗？

1. Yes 是
2. No 否 → Skip to FD020 请跳至FD020

**FD019.** Where is your personnel file kept? 您的档案存放在哪里？

1. With my current employer 当前雇主处
2. Other work unit but not current employer 其他工作单位(非当前雇主处)
3. With the Job Service Center in this city 本县/市人才服务中心
4. HuKou place in other city户口所在地
5. Other city 其他城市

**FD020.** Did you receive a labor contract (or employment contract) in written form from your current workplace(or labor dispatch company)? 您与当前单位/劳务派遣单位签了书面的劳动合同吗？

1. Yes 是
2. No 否 Skip to FD024 请跳至FD024

**FD021.** What is the agreed period of employment (labor contract period)? 您的劳动合同的期限是多长？

1. Defined period固定期限 _______ 0..100 (FD021_1)Years 年 _______0..11(FD021_2)Months 月[IWER: if do not remember months, please fill '0'. 访员注意：如果记不住多少个月，请填“0”。 ]
2. Not defined 无固定期限            → Skip to FD024 请跳至 FD024
3. Same as the term of the project 以完成一定工作任务为期限的劳动合同

**FD022.** Has the current employment contract ever been renewed? 现在的劳动合同是续签过的吗？

1. Yes  是
2. No (Current contract is the first contract)  否(当前的合同是第一份合同)

→ Skip to FD024 请跳至FD024

**FD023.** How many times has the contract been renewed? 您的劳动合同是第几次续签？____ 1..50times 次

**FD024.** How long do you expect to work at your current workplace? 您预期还会在现在的单位干多久？

1. Less than one year 不到1年
2. One to two years 1-2年
3. Two to three years  2-3年       Skip FD025 请跳过FD025
4. More than three years  超过3年       Skip FD025 请跳过FD025

**FD025.** Why do you expect so? 你为什么这么觉得呢？

1. Because the predefined contract period will expire 因为之前签的合同快到期了
2. Because typically the contract expires (although there’s no written contract) 因为之前约定的时间快到了（尽管没有书面合同）
3. Because I was hired under the condition that I would resign upon the request of my employer 因为估计雇主会让我辞职
4. Because the current job/project will be completed 因为目前的工作/项目即将结束
5. Because the person I am substituting/replacing will return to work 因为我顶替的那个人要回来了
6. Because I can only work during certain seasons 因为我只能在某些时间段工作
7. Because I plan to find another job that better suits my job aptitude, abilities, and preferences 因为我想找一份更适合我的工作
8. Because I will reach retirement age as set by regulations/practice 因为我快到退休年龄了
9. Because of family care responsibilities, poor health, etc. 因为家庭或健康方面的原因
10. Other其他原因

**[If R has not processed retirement (FB011 =2) ask: 如果被访者还没有办理退休手续，问:]**

**FD026.** Is this work unit going to process retirement for you? 您目前的工作单位将给您办理退休手续吗？

1. Yes 是
2. No  否                          →Skip to FD029 请跳至FD029

**FD027.** At what age will you process retirement? 您将在多少岁办理退休手续？____ 45..120 Years old 岁

**Skip to FD029 请跳至FD029**

**[Soft Check for Reasonable Age: If (FD027 < 50 & CV004==2) or (FD027 < 55 & CV004==1) prompt for verification.]**

**[Add another check to see if the reported age above is smaller than actual age. To be specific, FD027 < (Interview Year – CV009_a)]**

**If R has processed retirement (FB011 =1) ask: （如果被访者已经办理退休手续问：）**

**FD028.** Did you process retirement through this work unit? 您是在这个单位办的退休手续吗？

1. Yes 是
2. No 否

**FD029.** Except for national/public holidays, how many days of paid vacation do you have this year at your current workplace? 除国家法定节假日以外，今年您可以享受几天的带薪休假？_____ 0..366Days 天

[IWER: Mark 0 if there is no paid vacation. 访员注意：如果没有带薪休假，请标明“0”。]

**[Soft Check for reasonable range: If FD029 >30?, prompt for verification]**

**FD030.** How many days of work did you miss at this current job in the past year due to health problems? 在过去的一年中，您由于健康原因，在这个单位请假多少天？_______ 0..366Days 天

[IWER: Mark 0 if you didn't miss work. 访员注意：如果您没有不在职的情况，请标明“0”。]

**[PROCEDURE: If FD030 =0, skip FD031] [程序：如果FD030 =0，跳过FD031]**

**FD031.** In these days, how many did not deduct wage or bonus? 在这些天里，有多少天是不扣工资或奖金的？______ 0..366Days 天

**[Soft Check for reasonable range: If FD031 > FD030, prompt for verification]**

### FE QUESTIONS ABOUT LABOR SUPPLY 劳动力供给：

[CAPI: For dispatched worker (FD001 =2), prompt for FE001 - FE003 :] The next few questions about labor supply are about the situation of your work place, not dispatch work unit. [CAPI: 对派遣工人(FD001 =2)，对FE001 -FE003 均提示：] **下面几个关于劳动力供给的问题是指您工作单位的情况，而不是派遣单位。**

**FE001.** Counting paid vacations and sick leave not deducting wage as work, how many months did you work in the past year? 过去一年中，您工作了几个月（带工资的假期和不扣工资的病假算作工作时间）？ ______ 0..12Months 月

**FE002.** How many days a week did you work on average in the past year? 过去一年中，您一般每周工作几天？_______ 0..7Days 天

**FE003.** How many hours did you work per day on average in the past year, excluding meal breaks but including any paid or unpaid overtime? 过去一年中，您一般每天工作多少个小时？工作时间不包括午休时间，但包括加班时间（不管是否有报酬）______ 0..24Hours 小时

[Soft Check: Verify if number of hours per day is unreasonable, e.g., FE003 >16]

### FF QUESTIONS ABOUT WAGES: 工资问题

[CAPI: For dispatched worker (FD001 =2), prompt for FF001 -FG014 : 对派遣工人(FD001=2)，对FF001 -FG014 均提示：] **The following questions about salary and benefits refer to what you receive from the dispatch company. 下面几个问题是关于您从派遣单位获得工资和福利的情况。**

**FF001.** How is your wage paid mainly? Is it regularly paid, contract-based, performance-based, or other? If it is regularly paid, please tell me how often you receive your wages. Do you have a yearly contract, monthly, weekly, daily, or hourly? Please select one. 您的工资主要是怎么支付的？是定期支付、按项目支付、按绩效支付还是其他？如果是定期支付，请回答您是多久领一次工资？您是否按年、月、周、日还是小时领取工资？请选择一个。

1. Yearly salary 年薪
2. Monthly salary 月薪 → Skip to FF004 请跳至FF004
3. Weekly salary 周薪 → Skip to FF006 请跳至FF006
4. Daily salary 日薪 → Skip to FF008 请跳至FF008
5. Hourly salary 小时工资 → Skip to FF010 请跳至FF010
6. Contract-based 按项目 → Skip to FF012 请跳至FF012
7. Performance-based 按绩效 → Skip to FF012 请跳至FF012
8. Other 其他 → Skip to FF012 请跳至FF012

**FF002.** What is the after-tax salary including bonus in the last year? 把奖金等各种收入都算在内，您过去一年从单位拿到多少钱（税后）？_______ Yuan 元

**[Soft Check: Prompt for clarification if under a low threshold, e.g. under 1200 RMB annual. Specifically, prompt to clarify if FF002 <1200RMB.]**

**FF003.** [IWER: If R is unwilling to answer or does not remember, ask unfolding bracket questions here. 访员注意：如果受访者不愿回答或者忘记了，在此处分级展开提问]10,000 /30,000/50,000/100,000/200,000 yuan 元

**Skip to FF014 请跳至FF014**

**FF004.** What is the after-tax salary including bonus last month? 把奖金等各种收入都算在内，您上个月从单位拿到多少钱（税后）？___ Yuan 元

**Soft Check: Prompt for clarification if under a low threshold, e.g. under 100 RMB per month. Specifically, prompt to clarify if FF004 <100RMB.**

**FF005.** [IWER: If R is unwilling to answer or does not remember, ask unfolding bracket questions here. 访员注意：如果受访者不愿回答或者忘记了，在此处分级展开提问]: 500 /1,000 /2,500/5,000 /10,000 yuan 元

**Skip to FF014 请跳至FF014**

**FF006.** What is the wage including bonus last week? 把奖金等各种收入都算在内，您上周从单位拿到多少钱（税后）？_____ Yuan 元

**[Soft Check: Prompt for clarification if under a low threshold, e.g. under 25 RMB per week. Specifically, prompt to clarify if FF006 <25RMB.]**

**FF007.** [IWER: If R is unwilling to answer or does not remember, ask unfolding bracket questions here. 访员注意：如果受访者不愿回答或者忘记了，在此处分级展开提问]: 200/500 /10,00/25,00/5,000 yuan 元

**Skip to FF012 请跳至FF012**

**FF008.** What is the usual daily wage? 您一般每天挣多少钱？_____ Yuan 元.

**Soft Check: Prompt for clarification if under a low threshold, e.g. under 5 RMB per day. Specifically, prompt to clarify if FF008 <5 RMB.**

**FF009.** [IWER: If R is unwilling to answer or does not remember, ask unfolding bracket questions here. 访员注意：如果受访者不愿回答或者忘记了，在此处分级展开提问]: 20 /50 /100 /200/500 yuan 元

**Skip to FF012 请跳至FF012**

**FF010.** What is your hourly wage? 您每小时的工资是多少？____Yuan 元

**Soft Check: Prompt for clarification if under a low threshold, e.g. under 1 RMB per hour. Specifically, prompt to clarify if FF010 <1 RMB.**

**FF011.** [IWER: If R is unwilling to answer or does not remember, ask unfolding bracket questions here. 访员注意：如果受访者不愿回答或者忘记了，在此处分级展开提问]: 10 /30/50/100/200 yuan 元

**Skip to FF012 请跳至FF012**

**FF012.** How much on average do you receive last month after taxes (including bonus)? 把奖金等各种收入都算在内, 您上个月税后拿到多少钱？_____Yuan 元

**Soft Check: Prompt for clarification if under a low threshold, e.g. under 100 per month. Specifically, prompt to clarify if FF012 <100 RMB.**

**FF013.** [IWER: If R is unwilling to answer or does not remember, ask unfolding bracket questions here. 访员注意：如果受访者不愿回答或者忘记了，在此处分级展开提问]: 1,000/3,000/5,000/10,000/20,000 yuan 元

**FF014.** What is the value of all other bonuses (not paid at same time as regular wage) received in the past year? 过去一年中，您从单位拿到的其他所有奖金（不和薪酬一起按期支付，比如年终奖等）一共有多少钱（税后）？______Yuan 元

**Soft Check: Prompt on bonus and monthly earnings if bonus is more than five times monthly net income, e.g., re-ask some questions if FF014 >5*FF012 | FF014 >5*FF012**

**[JGuo: delete the end part of the above sentence?]**

**FF015.** [IWER: If R is unwilling to answer or does not remember, ask unfolding bracket questions here. 访员注意：如果受访者不愿回答或者忘记了，在此处分级展开提问]1,000/3,000 /5,000/10,000/20,000 yuan 元

### FG Fringe Benefits 单位福利

**[Show Card 出示卡片21]**

**FG001.** The following are fringe benefits which may be provided by a company. Please answer if the following are provided by your current workplace and whether you benefit from the following. (Check all that apply) 下面我们将列举一些福利。请回答您目前公司的福利情况以及您是否享受以下福利？(可多选)

1. Free lunch 免费午餐
2. Free breakfast 免费早餐
3. Free dinner 免费晚餐
4. Meal cash subsidy 餐费补贴
5. Transportation cash subsidizations 交通费补贴
6. Free housing 免费住宿
7. Subsidization of housing 住房补贴
8. Company car 单位配车
9. Company bus 单位班车
10. Other subsidies 其他补贴（包括实物折合）
11. None 没有 Skip to FG003 请跳至FG003

**For each choice, ask: 对上题的每一个答案，都分别提问FG002**

**FG002.** How much is the value of the subsidy per month? 每个月这种补助有多少（值多少钱）？__ Yuan 元

[CAPI: If FD006 = 1 or FD007 = 1 or FD009 =1, skip FG003 -FG014. 如果是公务员、政府机构的其他正式编制的员工、或事业单位的正式编制的员工FD006 = 1 or FD007 = 1 or FD009 =1，跳过FG003 -FG014]

**[PROCEDURE: If R has not processed retirement（FB011 =2）, ask: FG003 -FG014. 程序：如果受访者没有退休（FB011 =2），问：FG003 -FG014 ]**

**[Show Card 出示卡片22]**

**FG003.** Does your employer provide pension insurance, health insurance, unemployment insurance, worker’s injury insurance and maternity insurance ？(Choose all that apply) 您的雇主是否提供养老保险、医疗保险、失业保险、工伤保险和生育保险？（可多选）

1. Pension 养老保险 → Skip to FG009 请跳至FG009
2. Health insurance 医疗保险 → Skip to FG009 请跳至FG009
3. Unemployment insurance 失业保险 → Skip to FG009 请跳至FG009
4. Worker’s injury insurance工伤保险 → Skip to FG009 请跳至FG009
5. Maternity insurance生育保险 → Skip to FG009 请跳至FG009
6. None没有

**FG004.** Why are you not covered by the above mentioned social insurance through your employer?（Choose all that apply）? 单位为什么没有给你上社会保险（包括养老保险、医疗保险、失业保险、工伤保险、生育保险）?（可多选）

(1) There is no social insurance through employment in my local area 本地没有职工社会保险 → Skip to FG014 请跳至FG014

(2) Social insurance through work is available in my local area but my employer does not provide it to me 本地有这些项目但是我的单位不给我上保险 → Skip to FG014 请跳至FG014

(3) My employer offers it but I am unwilling to join单位虽然提供但是我不愿意参加 → Skip to FG014 请跳至FG014

(4) I am in the trial period 我在试用期中间→ Skip to FG014 请跳至FG014

(5) I have social insurance from elsewhere我在其他地方上了保险 → Skip to FG005 请跳至FG005

(6) I have passed retirement age 我已经过了退休年龄 → Skip to FG014 请跳至FG014

**FG005.** From where do you have/receive the above mentioned social insurance? 您在哪儿上的社会保险（包括养老保险、医疗保险、失业保险、工伤保险、生育保险）？

(1) I am covered through another work unit 通过另一个工作单位 → Skip to FG006 请跳至FG006

(2) I contribute through the job service center 通过人才交流中心 → Skip to FG008 请跳至FG008

**FG006.** Why do you contribute the above mentioned social insurance fees through another work unit? 您为什么要通过另一个工作单位缴纳社会保险（包括养老保险、医疗保险、失业保险、工伤保险、生育保险）费？

1. I was laid off by this work unit but my employment contract has not terminated 我在这家工作单位下岗，但劳动关系没有终止。
2. I am on leave from this employer 我从这家单位请假。
3. I keep my position but do not receive pay from this work unit 我在这家工作单位办了停薪留职。
4. A friend in this work unit is helping me this way 我在这家单位有个朋友帮我。
5. Other 其他

**FG007.** Where is this work unit located? 这个单位在哪？

- 1. Same city 就在这个城市
  2. Different city, same province 省内的其他城市
  3. Different province 其他省

**Skip to FG009 请跳至FG009**

**FG008.** Who pays for your contribution through the job service center? 您通过人才交流中心上社会保险（包括养老保险、医疗保险、失业保险、工伤保险、生育保险），请问由谁出钱？

1. My employer 雇主
2. Myself 我自己
3. My employer and myself雇主和我各负担一部分

**All those paying social insurance through whatever means, ask（FG003=1-4 or FG004=5）：只要上了社会保险的人，不管通过什么渠道上的，都请回答：FG009 – FG013**

**FG009.** Is the salary you told me about earlier the net amount after paying for the above benefits? 您刚才告诉我的您的收入，扣除了这些上交的保险金额吗？

1. Yes 是
2. No 否

**FG010.** On what income base is the contribution to the above mentioned social insurance determined? 请问您缴纳社会保险（包括养老保险、医疗保险、失业保险、工伤保险、生育保险）费时是根据多少工资计算的？_______________ Yuan/month元/月

**FG011.** Do you know how much you or your employer (another work unit) contributes to the above benefits? 您知道您或者您的雇主（另一个工作单位）给您缴纳了多少社会保险（包括养老保险、医疗保险、失业保险、工伤保险、生育保险）吗？

1. I know the amount by myself and the employer 我知道我和雇主各缴了多少
2. I know the amount by myself but not the employer我只知道我自己缴纳了多少，但不知道雇主缴了多少。→ Skip to FG013 请跳至FG013
3. I know neither my contribution nor my employer’s contribution.我既不知道我缴了多少，也不知道雇主缴了多少。 Skip to FG014 请跳至FG014

**FG012.** How much is your employer’s contribution in total? 您的雇主每月给您缴了多少？

______ Yuan/month 元/月

**Soft Check Income Report and FG012 if Contribution is Greater than Monthly Income**

**FG013.** How much is your own contribution in total? 您个人每个月缴纳了多少社会保险（包括养老保险、医疗保险、失业保险、工伤保险、生育保险）？___Yuan/month 元/月

**Soft Check Income Report and FG013 if Contribution is Greater than Monthly Income**

**FG014.** Does your employer provide funding for public housing? 您的雇主是否提供住房公积金？

1. Yes 是
2. No 否

F1：“住房公积金”指单位及其在职职工缴存的长期住房储金，是住房分配货币化、社会化和法制化的主要形式。

**Soft Check: Verify if FG014 > than some threshold.**

**FG015.** At what age do you plan to stop working? Stopping work in this context shall refer to having stopped all income-related activities and unpaid family business and having no intention of engaging in anything more serious than small pastime work. 您计划在多大年龄时停止工作，即停止从事一切以挣钱为目的的活动以及不再为家庭经营活动帮工，将来也不打算从事比消遣性工作更劳累的工作?_____ 0..120Years old 岁

F1：“消遣性工作”指不是以挣钱为目的的活动，以及不再为家庭经营活动帮工的工作。

[IWER: Please tell me the approximate age. Mark 0 if you plan to keep working until you are physically able. 访员注意：请告知大概年龄。如果您计划只要健康允许，就一直工作，请标明“0”。]

**Soft Check: Prompt for correction if current age is greater than response, e.g. (Interview Year+ Interview month/12) – (CV009_a+CV009_b/12) > FG015**

**→ Skip to FJ001 请****跳至FJ001**

### FH NON-FARM SELF-EMPLOYED AND UNPAID FAMILY BUSINESS 非农自雇和为家庭经营活动帮工

**FH001.** How many months did you work in the past year? 过去一年中，您工作了几个月？______ 0..12 Months 月

**FH002.** How many days did you work per week on average in the past year? 过去一年中，您一般每周工作几天？ _____ 0..7Days 天

**FH003.** How many hours did you work per day on average in the past year, excluding meal breaks but including any paid or unpaid overtime on a normal work month?过去一年中，您一般每天工作多少个小时？不包括午餐和其他休息时间，但是包括加班时间，不管是否有报酬 ______ 0..24hours 小时

**Soft Check: Verify if number of hours per day is unreasonable, e.g., FH003 >16**

**FH004.** How many days of work did you miss in the past year due to health problems? 过去一年中，您由于健康原因，有多少天没有上班？______0..366Days 天

[IWER: Mark 0 if you didn't miss any work days. 访员注意：如果没有，请注明“0”。]

**Next are some questions about the main self-employed work. 下面是关于您从事的主要个体或者私营经济活动的问题**

**IF FC020 =2 or FC021 =2[Self-employed], ask如果FC020 =2 或者 FC021 =2，受访者自雇，询问:**

**FH005.** What is the name of your company or workplace? 您的企业或者生意的名称是？

[IWER: If there is more than one company, ask about the main one. Mark 0 if there is no name. 访员注意：如果在几个企业工作或者做了多项生意，问主要工作的企业或者主要从事的生意。如果没有名称，请标明“0”。] ___________________________________________

**FH006.** Where is your company or workplace located? 您的公司/生意的地址是？

(1)this village/community 本村/社区

(2)other village/community in this county/city 本县/市的其他村/社区

(3)Another county/city in this province在本省的其他县/市，_____ (FH006_1)city市________(FH006_2) county县

(4)Another province 外省：________(FH006_3)province 省/自治区/直辖市，___________(FH006_4)city市__________(FH006_5) county县00000000000000000000000000000000000000000000000000000000000000000000000000000000000000000000000000000000000000000000000000000000 136___别是？0000000000000000000000000000000000000000000000000000000000000000000000000000000000000000000000000000000000000000000000

[IWER: Mark 0 if there is no fixed address. 如果没有具体地址，请标明“0”。]

[IWER: Choose from the list of provinces，see appendix 2. 请选择省份，见附表二]

**FH007.** What kind of business or industry do you work in—that is, what does your company do or make? 您主要做什么生意，即生产什么产品或者从事什么经营活动？______________________________________

[IWER: Type of business 产业或行业类型]

**FH008.** When did you start working at the current company or workplace? 您什么时候开始经营这个企业/这项生意？______ 1900..2011 (FH008_1)Year年_______0..12 (FH006_2) Month 月

[IWER: Mark the year using four digits. Take down the month as its actual number. For example, write January as “1” not “01”, December as “12”. If do not remember month, fill ‘0’. 访员注意: 用4位数表示年，按照实际的月份填写月。例：1月写作“1”，而不是“01”,12月写作“12”。如果记不住月份，请填入’0’。]

[**A check similar to the one for FD011**. **(FD011_1 + FD011_2 /12)- (CV009_a+ CV009_b/12) < 16 or (FD011_1 + FD011_2 /12)-(CV009_a+ CV009_b/12) < FB001_1 or FH008_1 < FB001_2** ]

**FH009.** Do any other household members work in the same self-employed activity? 您是否还有其他家人也从事同样的个体或者私营经济活动？

1. Yes 是 Skip to FI001 跳至FI001
2. No 否

**[IWER: Income from self-employment if R is the single operator （FH009 =2）. If other family member involved, income is asked in household section. 访员注意：如果受访者是自己一个人开公司、做生意等，那么接下来问收入；如果还有其他家人一起做事，那么跳至FI001 , 在家庭问卷中问收入。]**

**FH010.** Not including spending on fixed capital, what is your best estimate of net income earned from this activity in the last year? Remember to consider the following types of costs: energy, housing or equipment rental, raw materials, transportation, marketing, wages, taxes, and other fees. 不包括在固定资本上的投入，您能否估计一下过去一年您开这个公司/做这项生意挣了多少钱？我们指的是刨除能源、住房和设备租用费、原材料、交通费、营销、工资、税收和杂费之后的净收入。________Yuan元

**Soft Check: Prompt for verification if net income is less than 1200 RMB / year. Or, prompt if FH010 <1200RMB**

**FH011.** [IWER: If R is unwilling to answer or does not remember, ask unfolding bracket questions here. 访员注意：如果受访者不愿回答或者忘记了，在此处分级展开提问]: 5,000 /10,000/50,000/100,000/200,000 yuan 元

**Next are some questions about your unpaid family business. 下面是关于您不拿工资为家庭经营活动帮工的问题**

**IF FC020 =3 or FC021 =3[Unpaid family business], ask:**

**如果FC020 =3 或者 FC021 =3[受访者不拿工资为家庭经营活动帮工], 询问:**

**FH012.** What is the name of company or workplace that you work in without wage? 您无偿帮忙的企业或者生意的名称是？[IWER:Mark 0 if there is no name. 如果没有名称，请标明“0”.]

____________________________________________

**FH013.** Where is this company or workplace loacted? 这个公司/生意的地址是？

(1)this village/community 本村/社区

(2)other village/community in this county/city 本县/市的其他村/社区

(3)Another county/city in this province在本省的其他县/市，___________(FH013_1)city市_______ (FH013_2)county县

(4)Another province 外省：________(FH013_3)province 省/自治区/直辖市，___________(FH013_4)city市___________（FH013_5）county县00000000000000000000000000000000000000000000000000000000000000000000000000000000000000000000000000000000000000000000000000000000 136___别是？0000000000000000000000000000000000000000000000000000000000000000000000000000000000000000000000000000000000000000000000

[IWER: Mark 0 if there is no fixed address. 如果没有具体地址，请标明“0”。]

[IWER: Choose from the list of provinces，see appendix 2 请选择省份，见附表二]

**FH014.** What kind of business or industry do you work in—that is, what does this company do or make? 他们主要做什么生意，即生产什么产品或者从事什么经营活动？________________________________

[IWER: Type of business 产业或行业类型]

**FH015.** What sort of work did you do? (E.g., cleaner, accountant, etc.) 您具体从事什么工作？(例如清洁工、会计、出纳等) _______________________________________

[IWER: Ask the specific work. 访问员注意：询问具体工作内容。 ]

**FH016.** How many family members, relatives or friends who work without payment are there including yourself? 您有多少家人、亲戚或朋友在这个公司/生意不拿工资做事的？（包括您本人）

___ People 人

**FH017.** When did this company or workplace start operation？这个企业/生意是什么时候开办/经营的？

______ 1900..2011 (FH017_1)Year年_______0..12 (FH017_2) Month 月

**FH018.** When did you start working at the current company or workplace? 您什么时候开始在这个企业/生意帮忙的？______1900..2011 (FH018_1)Year年_______0..12 (FH018_2) Month 月

**FH019.** Do any other household members work in the same company or workplace? 您是否还有其他家人也在这个企业/生意工作？

（1）Yes 是 Skip to FI001 跳至FI001

（2）No 否

[IWER: Income from self-employment if no other household members work in（FH019 =2）. If other family member involved, income is asked in household section. 访员注意：如果没有其他家人在企业/生意工作，那么接下来问收入；如果还有其他家人一起做事，那么跳至FI001 , 在家庭问卷中问收入。]

**FH020.** Not including spending on fixed capital, what is your best estimate of net income earned from this activity in the last year? Remember to consider the following types of costs: energy, housing or equipment rental, raw materials, transportation, marketing, wages, taxes, and other fees. 不包括在固定资本上的投入，您能否估计一下过去一年这个公司/做这项生意挣了多少钱？我们指的是刨除能源、住房和设备租用费、原材料、交通费、营销、工资、税收和杂费之后的净收入。________ Yuan元

**Soft Check: Prompt for verification if net income is less than 1200 RMB / year. Or, prompt if FH020 <1200RMB**

**FH020_bracket.** [IWER: If R is unwilling to answer or does not remember, ask unfolding bracket questions here. 访员注意：如果受访者不愿回答或者忘记了，在此处分级展开提问]: 5,000 /10,000/50,000/100,000/200,000 yuan 元

### FI Social insurance questions for the self-employed. 自雇部分中的社会保险问题

**[PROCEDURE: If R has not processed retirement（FB011 =2）, ask: FI001 -FI011 . 程序：如果受访者没有退休（FB011 =2），问：FI001 -FI011。]**

**[Show Card 出示卡片22]**

**FI001.** Next are questions about your social insurance. Are you covered by pension insurance, health insurance, unemployment insurance, worker’s injury insurance or maternity insurance through your own self-employment？(Choose all that apply) 下面是一些关于社会保险的问题。您是否以个体或者私营业主的身份缴纳了养老保险、医疗保险、失业保险、工伤保险或生育保险？（可多选）

1. Pension养老保险 → Skip to FI005 请跳至FI005
2. Health insurance医疗保险 → Skip to FI005 请跳至FI005
3. Unemployment insurance失业保险 → Skip to FI005 请跳至FI005
4. Worker’s injury insurance工伤保险 → Skip to FI005 请跳至FI005
5. Maternity Insurance生育保险 → Skip to FI005 请跳至FI005
6. None无

**FI002.** Why are you not covered by the above mentioned social insurance through self-employment? 您为什么没有给自己上社会保险（包括养老保险、医疗保险、失业保险、工伤保险、生育保险）?

1. It is not possible for the self-employed to participate in social insurance in my local area 在本地自雇经营的人没有办法上社会保险 → Skip to FI012 请跳至FI012
2. Social insurance is available to the self-employed but I do not participate我可以上保险但是我不愿意参加 → Skip to FI012 请跳至FI012
3. I have social insurance from elsewhere 我在其他地方上了保险
4. I have passed retirement age 我已经过了退休年龄 → Skip to FI012 请跳至FI012

**FI003.** From where do you have the above mentioned social insurance? 您在哪上的社会保险（包括养老保险、医疗保险、失业保险、工伤保险、生育保险）？

1. I am covered through another work unit 我通过另一个工作单位上的
2. I contribute through job service center 我通过人才交流中心上的 → Skip to FI005 请跳至FI005

**FI004.** You contribute through another work unit, why? 请问您为什么通过另一个工作单位缴纳社会保险（包括养老保险、医疗保险、失业保险、工伤保险、生育保险）费？

1. I was laid-off by this work unit but employment contract is not terminated 我在这家工作单位下岗，但工作关系没有中止
2. I am on leave from this employer 我从这家单位请假，工作关系没有变动
3. I keep my position but do not receive pay from this work unit 我在这家工作单位办了停薪留职，没有转工作关系
4. A friend in this work unit is helping me this way 我在这家单位有个朋友帮我
5. Other 其他

**Skip to FI005 . 请跳至FI005**

**[PROCEDURE: If R has social insurance, either from self-employment or by job service center, ask: FI005 – FI011. 程序：如果受访者上了社会保险，无论是通过自雇还是人才交流中心，请提问：FI005 – FI011]**

**FI005.** How much is your own contribution to the above mentioned social insurance last year? 您过去一年上社会保险（包括养老保险、医疗保险、失业保险、工伤保险、生育保险）个人缴纳多少钱？____Yuan 元

**Soft Check: Prompt if FI005 =0 or FI005 > FH010 /2.**

**If FH009 =2，skip FI006 . 如果FH009 =2，跳过FI006**

**FI006.** Is the income you told us about earlier net after paying for the above benefits? 您刚才告诉我们的净收入，扣除了这些上交的保险金额吗？

1. Yes 是
2. No 否

**FI007.** On what income base is the contribution to the above mentioned social insurance determined? 请问您缴纳社会保险（包括养老保险、医疗保险、失业保险、工伤保险、生育保险）费时是根据多少收入计算的？_______________ Yuan/month元/月

**[PROCEDURE: If R participates in social insurance through another work unit （FI003 =1）, ask: FI008 -FI010 . 程序：如果被访者通过另一个单位缴纳社会保险（FI003 =1），提问： FI008 -FI010]**

**FI008.** Do you know how much you or that work unit contributes to the above benefits? 您知道您或者那个工作单位给您缴纳了多少社会保险（包括养老保险、医疗保险、失业保险、工伤保险、生育保险）吗？

1. I know the amount by myself and the work unit 我知道我和单位各缴了多少→ Skip to FI009 请跳至FI009
2. I know the amount by myself but not the work unit我只知道我自己缴纳了多少，但不知道单位缴了多少。 → Skip to FI010 请跳至FI010
3. I do not know either my contribution or the work unit’s contribution at all. 我既不知道我缴了多少，也不知道单位缴了多少。 → Skip to FI011 请跳至FI011

**FI009.** How much is work unit’s contribution in total? 单位每月给您缴了多少？ ______ Yuan/month 元/月

**Soft Check: Prompt for Verification if Contribution is Greater than Monthly Earnings, e.g., FI009 >FH010**

**FI010.** How much is your own contribution to pension in total? 您个人每月缴多少社会保险（包括养老保险、医疗保险、失业保险、工伤保险、生育保险）？ ______ Yuan/month 元/月

**Soft Check: Prompt for Verification if Contribution is Greater than Monthly Earnings, e.g., FI010 >FH010**

**FI011.** Do you expect to process retirement at this business/organization? 您预期将来在这个单位办理退休手续吗？

1. Yes 是
2. No 否

**FI012.** At what age do you plan to stop working? Stopping work in this context shall refer to having stopped all income-related activities and unpaid family business and having no intention of engaging in anything more serious than small pastime work. 您计划在多大年龄停止工作？即停止从事一切以挣钱为目的的活动以及不再为家庭经营活动帮工，将来也不打算从事比消遣性工作更劳累的工作?

______ 0..120Years old 岁

F1：“消遣性工作”指不是以挣钱为目的的活动，以及不再为家庭经营活动帮工的工作。

[IWER: Please tell me the approximate age. Mark 0 if you plan to keep working until you are physically able. 访员注意：请告知大概年龄。如果您计划只要健康允许，就一直工作，请标明“0”。]

**Soft Check: Prompt for correction if current age is greater than response, e.g. (Interview Year+ Interview month/12) – (CV009_a+CV009_b/12) > FI012**

### FJ SIDE JOB (EMPLOYED OR SELF-EMPLOYED) 非主要职业（受雇或自雇）

**If FC019 =1 (more than one job), proceed with the following section.如果 FC019 =1（工作超过一份）问这部分**

**FJ001.** How many jobs do you currently hold, excluding your main job? 除了主要工作，您目前还从事其他几种工作？ ______ 1..20种

**FJ002.** How many hours a week do you work on average at your side job(s), not considering your main job? 除了主要工作，您每周在其他工作上平均工作多少个小时？

________ 0..168 Hours per week 小时/周

**FJ003.** What is the average monthly income or wage that you get from side job(s) other than your main job? 除了主要工作的收入外，您从事其他工作每月的平均收入是？___________ Yuan per month 元/月

**FJ004.** [IWER: If R is unwilling to answer or does not remember, ask unfolding bracket questions here. 访员注意：如果受访者不愿回答或者忘记了，在此处分级展开提问]: 500 /1,000/2,500/5,000/10,000 yuan 元

**END OF CURRENT JOB 结束当前工作部分**

## FK UNEMPLOYMENT AND JOB SEARCH ACTIVITIES 失业，求职经历

**For those not currently working but once employed (FA007 =1) 当前没有工作但是曾经工作过的人回答(FA007 =1)：**

**FK001.** Next are some questions about circumstances about your non-employment and job search activities. In what month and year did you last work? 下面是一些关于您停止工作前后的一些情况和找工作的问题。您什么时候开始不工作的？

______ 1900..2011 (FK001_1)Year年_______0..12 (FK001_2) Month 月

[IWER: Mark the year using four digits. Take down the month as its actual number. For example, write January as “1” not “01”, December as “12”. If do not remember month, fill ‘0’. 访员注意: 用4位数表示年，按照实际的月份填写月。例：1月写作“1”，而不是“01”,12月写作“12”。如果记不住月份，请填入’0’。]

[**Compare with the time when started work. FK001_1 < FB001_1 or (FK001_1 – CV009_a) < FB001_1** ]

**FK002.** Did you search for a new job during the last month? 过去一个月您是否找过工作？

1. Yes 是
2. No 否                            Skip to FL001 请跳至FL001

**FK003.** At what age do you plan to stop working? Stopping work in this context shall refer to having stopped all income-related activities and unpaid family business and having no intention of engaging in anything more serious than small pastime work. 您计划在多大年龄停止工作？即停止从事一切以挣钱为目的的活动以及不再为家庭经营活动帮工，将来也不打算从事比消遣性工作更劳累的工作?

____0..120Years old 岁

F1：“消遣性工作”指不是以挣钱为目的的活动，以及不再为家庭经营活动帮工的工作。

[IWER: Please tell me the approximate age. Mark 0 if you plan on working until you are physically capable. 访员注意：请告知退休的大约年纪。如果您计划只要身体健康就一直工作，请注明“0”。]

**[Soft check: Compare with current age. FK003 < (Interview Year – CV009_a)**

## FL MOST RECENT JOB 最近一份工作

–– RESPONDENT NOT CURRENTLY WORKING

——目前没有工作的人，但曾经工作过的人回答

Ask if FA007 =1 or (FA007=2 and FA008 =2)

IWER：The next questions are about the last main job you had, which could be farming, earning a wage, running your own business or working for unpaid family business. It does not include doing your own housework or doing activities without pay, such as voluntary work. If you have more than one job, we are interested in the job at which you worked the longest hours. We’re interested in your situation near the termination of this job. 下面的问题是关于您最近一份工作的情况，可以是务农、挣工资工作、从事个体、私营经济活动、或者不拿工资为家庭经营活动帮工，但不包括家务劳动或者志愿者劳动等没有报酬的工作。如果您之前同时从事不止一份工作，我们想了解您的主要工作，即占用您时间最多的那份工作。

**FL001.** Did you work for someone else(including work for unpaid family business), were you self-employed, did you farm, or were you otherwise employed? 您之前是为别人工作（包括不拿工资为家庭经营活动帮工）还是自己从事个体或者私营经济活动，或者务农？

1. Employed 受雇（包括非农打工和在外地的农业打工等）
2. Self-employed自雇（从事个体或者私营经济活动）
3. Unpaid family business 不拿工资为家庭经营活动帮工
4. Farming 农业生产活动（农业生产经营活动，包括为自家或其他农户种地、管理果树、采集农林产品、养鱼、打鱼、养牲畜以及去市场销售自家生产的农产品等；但不包括在外地的农业打工）

**FL002.** In which year and month did you start working at that job? 您从哪年哪月开始做这份工作的？

______1900..2011 (FL002_1) Year年_______0..12 (FL002_2) Month 月

[IWER: Mark the year using four digits. Take down the month as its actual number. For example, write January as “1” not “01”, December as “12”. If do not remember month, fill ‘0’. 访员注意: 用4位数表示年，按照实际的月份填写月。例：1月写作“1”，而不是“01”,12月写作“12”。如果记不住月份，请填入’0’。]

**Soft Check: Prompt for Verification/Clarification if the Respondent was Less than 16 at time of starting this job, e.g. Prompt if (FL002_1+FL002_2 /12)-(CV009_a+CV009_b/12)<16**

**[Replace 16 in the above check with FB001 . FL002_1 < FB001_2 or FL002_1 < (CV009_a + FB001_1 )]**

**FL003.** In which year and month did you stop working at that job? 您什么时候停止做这份工作的？

______1900..2011 (FL003_1) Year年_______0..12 (FL003_2) Month 月

[IWER: Mark the year using four digits. Take down the month as its actual number. For example, write January as “1” not “01”, December as “12”. If do not remember month, fill ‘0’. 访员注意: 用4位数表示年，按照实际的月份填写月。例：1月写作“1”，而不是“01”,12月写作“12”。如果记不住月份，请填入’0’。]

**Soft Check: Prompt for Verification/clarification if End Date is Before the Start Date, e.g. FL003_1 <FL002_1**

**FL004.** Where was the job located?　工作地点在哪里？

(1)this village/community 本村/社区

(2)other village/community in this county/city 本县/市的其他村/社区

(3)Another county/city in this province在本省的其他县/市，___________(FL004_1)city市___ ________ (FL004_2)county县

(4)Another province 外省：________(FL004_3)province 省/自治区/直辖市，___________(FL004_4)city市___________(FL004_5)county县00000000000000000000000000000000000000000000000000000000000000000000000000000000000000000000000000000000000000000000000000000000 136___别是？0000000000000000000000000000000000000000000000000000000000000000000000000000000000000000000000000000000000000000000000

[IWER: Mark 0 if there is no fixed address. 访员注意：如果没有具体地址，请标明“0”。]

[IWER: Choose from the list of provinces，see appendix 2 访员注意:请选择省份，见附表二]

**If FL001 =4 Skip to FL018 如果FL001 =4，跳至FL018**

**[PROCEDURE:If non-farmer（FL001 =1or 2 or 3）, ask FL005 to FL008 . 程序：如果不是农民（FL001 =1或者2或者3），问FL005 至FL008]**

**FL005.** What was the name of your workplace/employer? Please state specifically the name of your company or business. 您的工作单位或者雇主是什么名称？（请详细标明名称）______________________________________________________________

**FL006.** What kind of business or industry was it—that is, what did they make or do at the place where you worked? 该单位主要是做什么的，也就是，他们制造什么产品或者从事什么活动？ _____________________________________________________________

[IWER: Type of business访员注意:产业或行业类型]

**FL007.** Is this employer still in existence? 这个单位还存在吗？

1. Yes 是
2. No 否

**FL008.** How many hours a week did you usually work [for this employer/in this business]? 您当时一般每周工作几个小时？[做这份工作]_____ 0..168hours per week小时/每周

**Soft Check: Prompt for Verification if FL008 >80**

**Ask only if employed.如果是受雇（FL001 =1），问：**

**FL009.** What were the monthly wages, bonuses, and subsidies from this job，before you stopped working at this job? 您停止做这份工作前，平均每月总共挣多少钱（包括工资、奖金和补贴）？______Yuan 元

[IWER: Mark 0 if there is no net income, and mark 999997 if running a deficit.访员注意：如果没有净收入请标注0，如果是亏损请标注999997.]

**Soft Check: Prompt for Verification if FL009 < 100**

[IWER: If R is unwilling to answer or does not remember, ask unfolding bracket questions here. 访员注意：如果受访者不愿回答或者忘记了，请回答：]

**FL010.** [IWER: If R is unwilling to answer or does not remember, ask unfolding bracket questions here. 访员注意：如果受访者不愿回答或者忘记了，在此处分级展开提问]: 500 /1,000/2,500/5,000/10,000 yuan 元

**FL011.**What was the value of other bonuses not paid with regular wages each year? 您从单位拿到的其他所有奖金（不和薪酬一起按期支付，比如年终奖等）每年一共有多少钱？______yuan 元

**Skip to FL013 跳至FL013**

**Ask if self-employed 如果是自雇（FL001 =2），问:**

**FL012.** Do you have employees? 您有雇员吗？

1. Self-employed with employees 有
2. Self-employed without employees 没有
3. Family business worker without pay 家里人一起做，不付工资

**Skip to FL018 跳至FL018**

**Ask the following only if employed（FL001=1）:对于问题FL013-FL017，只向受雇人群提问**

**FL013.** Were you a regular worker, a temporary worker, or a casual worker? 您是正式员工、合同制员工、临时工，还是小时工？

1. Regular wage worker 正式员工
2. Contract worker 合同制员工
3. Temporary wage worker 临时工
4. Casual wage worker 小时工

**FL014.** Did you work for the government, institution, firm, NGO, individual farmer or a resident household? 您是为政府机构、事业单位、企业单位、个体户、非赢利机构（如社团、协会、学会等）、农户，还是居民户工作？

1. Government 政府部门
2. Institutions 事业单位 →Skip to FL017 跳至FL017
3. NGO 非营利机构（如社团、协会、学会等）→Skip to FL017 跳至FL017
4. Firm 企业 →Skip to FL016 跳至FL016
5. Individual firm 个体户 Skip to FL017 跳至FL017
6. Individual farmer农户 →Skip to FL017 跳至FL017
7. Individual household 居民户 →Skip to FL017 跳至FL017
8. Other其他 →Skip to FL017 跳至FL017

If government employee [FL014 =1], ask: 如果受访者是政府雇员[FL014=1], 问：

**FL015.** Were you a civil servant? 您是公务员吗？

1. Yes 是
2. No 否

Skip to FL017 请跳至FL017

If firm [FL014 ＝4], ask: 如果在企业工作[FL014 ＝4], 问：

**[Show Card 出示卡片20]**

**FL016.** What was the ownership type of the business? 您的企业的所有制类型是？

1. 100% State owned firm 国有企业
2. State-controlled firm国有控股企业
3. 100% Collective-owned firm 集体所有制企业
4. Collective-controlled firm集体控股企业
5. 100% Private firm 私营/个体
6. Private-controlled firm私人控股企业
7. 100% foreign-owned 外商独资
8. Joint venture中外合资
9. Other joint-ownership 其他联营企业
10. Other 其他

**FL017.** What sort of work did you do? (E.g., cleaner, accountant, etc.) 您自己具体从事什么工作？(例如清洁工、会计、出纳等) ____________________________________

[IWER: Ask the specific work. 访问员注意：询问具体工作内容。 ]

[ IWER: If R has processed retirement [FB011=1], ask : 访员注意：如果受访者办理了退休手续，请回答:]

**FL018.** Is this the business/organization where you processed retirement? 您是在这个单位办的退休手续吗？

1. Yes 是 Skip to FL021 跳至FL021
2. No 否 Skip to FL020 跳至FL020

[IWER: If R has not processed retirement [FB011 =2], ask: 访员注意：如果受访者没有办理退休手续，请回答:]

**FL019.** Do you expect to process retirement from this business/organization? 您预期将来从这个单位或以自营职业的身份办理退休手续吗？

1. Yes 是
2. No 否

**FL020.** Why did you leave that employer? 您为什么离开这个工作单位？或者您为什么不继续您的生意或务农了，或者您为什么不再为家庭经营活动帮工了呢？

[IWER: Do not probe but check all that apply. 访员注意：不用深究，请选择全部适用项。]

1. Business closed 单位破产／倒闭了(FL020_1)
2. Quit 辞职(FL020_2)
3. I was laid off 下岗(FL020_3)
4. I was fired 被辞退(FL020_4)
5. I went to school 我上学了(FL020_5)
6. I went abroad 我出国了(FL020_6)
7. I stopped working for health reasons 因为健康原因中断工作(FL020_7)
8. I stopped working for family reasons 因为家庭原因中断工作(FL020_8)
9. I was transferred to another job 工作调动(FL020_9)
10. I was sent down to the countryside to do manual labor 上山下乡(FL020_10)
11. I started working off-farm locally 我在本地从事非农工作了(FL020_11)
12. I went to work away from home 我外出打工了(FL020_12)
13. Better job in local area 本地更好的新工作(FL020_13)
14. Better job in another location 外地更好的新工作(FL020_14)
15. I retired 我退休了(FL020_15)
16. Other 其他(FL020_16)

**FL021.** Did you receive any payments other than the legal retirement allowance upon leaving your last job? (For example, condolence payment，workers compensation, etc.) 上一份工作结束时，除去退休工资，您是否还得到了其他补偿（如买断工龄，伤残津贴等）？

1. Yes 是
2. No 否

F1：“买断工龄”指改革开放初期我国一些国有企业在改革过程中安置富余人员的一种办法，即参照员工在企业的工作年限、工资水平、工作岗位等条件，结合企业的实际情况，经企业与员工双方协商，报有关部门批准，由企业一次性支付给员工一定数额的货币，从而解除企业和富余员工之间的劳动关系，把员工推向社会的一种形式

If FL021 =1 ask:如果FL021.回答是，问：

**FL022**. How much was the compensation and for how many years of work?________ Yuan元____ years 您得到了多少补偿?_______ (FL022_1)元；是按照多少工龄算的？_______0..120(FL022_2 )年

**FL022_bracket.** [IWER: If R is unwilling to answer or does not remember, ask unfolding bracket questions here. 访员注意：如果受访者不愿回答或者忘记了，在此处分级展开提问]: 1000 /2000/5000/10,000/20,000 yuan 元

**End of Last Job 上一份工作部分结束**

## FM RETIREMENT 退休与退职

[IWER: If R has processed retirement [FB011 or FB012 =1], ask: FM001 –FM059. 访员注意：如果受访者已经办了退休手续[FB011=1]或者退职手续[FB012=1]，提示受访者，您刚才告诉我们您已经办理了退休/退职手续，下面是关于您的退休/退职手续的一些问题，并提问FM001 –FM059]

**FM001.** Is the work unit that processed your [preload: retirement /receding position] the one you told us about (preload: name of current employer if employed EP027, name of last employer if non-employed EP146)? 给您办理[退休/退职]手续的单位是[preload work unit name, 请下载单位名称]吗？

[IWER: If R has no work unit (no answer to FD003 or FL005 ), please choose “(2) No”. 访员注意：如果此受访者没有工作单位，即FD003 和FL005 都没有回答，请选择“（2）不是”，并继续]

1. Yes 是 Skip to procedure before FM005 请跳至FM005前的程序控制
2. No 不是

**If answer to FM001 =2 is no, then ask FM002 -FM004 :如果FM001 =2回答不是，问FM002 -FM004 ：**

**FM002.** What is the name of the employer that processed your [preload: retirement /receding position]? 给您办理[退休/退职]手续的单位的名称是什么？______________

**FM003.** What was the type of your work unit at [preload: retirement /receding position]? 您办理[退休/退职]的单位是哪种类型的？

1. Government 政府部门
2. Institutions 事业单位
3. NGO 非营利机构（如社团、协会、学会等）
4. Firm 企业
5. Individual个体或者私营企业主
6. Other 其他

**FM004.**  Where is this work unit located? 这个单位的地址是？

(1)this village/community 本村/社区

(2)other village/community in this county/city 本县/市的其他村/社区

(3)Another county/city in this province在本省的其他县/市，_________(FM004_1)city市_________ (FM004_2) county县

(4)Another province 外省：______(FM004_3)province 省/自治区/直辖市，_________(FM004_4)city市_______(FM004_5) county县00000000000000000000000000000000000000000000000000000000000000000000000000000000000000000000000000000000000000000000000000000000 136___别是？0000000000000000000000000000000000000000000000000000000000000000000000000000000000000000000000000000000000000000000000

[IWER: Mark 0 if there is no fixed address. 如果没有具体地址，请标明“0”。]

[IWER: Choose from the list of provinces，see appendix 2 请选择省份，见附表二]

If FB012 =1（Receding, ask FM005 -FM009 .如果FB012 =1（受访者退职），询问FM005-FM009

**FM005.** In what month and year did you recede from your position? 您是在哪年哪月办理的退职？______ (FM005_1)1900..2011Year年_______(FM005_2)0..12 Month 月

[IWER: Mark the year using four digits. Take down the month as its actual number. For example, write January as “1” not “01”, December as “12”. If do not remember month, fill ‘0’. 访员注意: 用4位数表示年，按照实际的月份填写月。例：1月写作“1”，而不是“01”,12月写作“12”。如果记不住月份，请填入’0’。]

**FM006.** What was the main reason you receded from your position?  您办退职的主要原因是？

- - 1. Due to poor health, I couldn't continue my work any more, at the same time I wasn't eligible for retirement 身体原因不能继续工作，而又不符合退休条件
    2. Years of eligible work are less than three，and time of stopping work due to diseases or injures not related to work are more than one year 连续工龄不满三年，因病或非因工负伤而停止工作的时间满一年
    3. I'm recruit worker within 6 months, but I had serious chronic disease once and can't tstick to work any more 录用后在六个月以内，发现原来有严重慢性疾病，不能坚持工作的
    4. I receded from my position voluntary自愿退职
    5. Reach retirement age, but not eligible working age. 到了退休年龄，但工龄不够
    6. Other 其他

**FM007.** Your pre-receding total salary was _______Yuan a month (including basic wage, bonus, et. al).

您退职前的总工资是每月多少钱？________元/月, 包括基本工资和奖金等。

**FM008.** Did you receive any payments for leaving your job？您是否得到了退职补偿？

（1）Yes 是

（2）No 否

**FM009.** If FM008 =1 ask: 如果FM008， 回答是，问：

How much was the compensation?_ _______ Yuan元

您得到了多少补偿?_______元；

**FM010.**  Are you currently receiving pension? 您现在领取养老金（退休金）吗？

（1）Yes 是 skip to FM018 跳至FM018

（2）No 否

**Skip to** **FM042 跳至FM042**

**If FB011 =1（Retirement）, ask FM011- FM041：如果FB011 =1（受访者退休），询问FM011- FM041**

**FM011.** Was your retirement normal retirement; early retirement; or internal retirement initially, followed by regular retirement? 您办的是正常退休, 提前退休还是先内退然后办的正式退休或者将来再办正式退休？

(1) Normal retirement 正常退休

(2) Early retirement 提前退休

(3) Internal retirement first, then regular retirement 先内退然后又正式退休

(4) Internal retirement, but not yet regular retirement内退但目前还没有办理正式退休

**FM012.** Did you retire as a worker or as a cadre? 您退休时的身份是干部还是工人？

(1) Worker 工人

(2) Cadre 干部

[IWER: If year of first job was before 1952 (compute from FB001 ), then ask: 访员注意：如果第一份工作是在1952年之前，问：]

**FM013.** Are you an ordinary retiree or revolutionary retiree? 您现在是退休还是离休？

(1) Ordinary retiree 退休

(2) Revolutionary retiree离休

F1：“离休”指对建国前参加中国共产党所领导的革命战争、脱产享受供给制待遇的和从事地下革命工作的老干部，达到离职休养年龄的 ，实行离职休养的制度。已经退休的干部，符合上述条件的，应当该为离休。

**If FM011 =1 or 2, ask FM014 -FM024 :如果FM011 =1 or 2，问FM014 -FM024 ;**

**FM014.** In what month and year did you take [preload: normal/early] retirement? 您办理[正式/提前]退休手续是在哪年哪月？______ 1900..2011 (FM014_1)Year年_______0..12 (FM014_2) Month 月

[IWER: Mark the year using four digits. Take down the month as its actual number. For example, write January as “1” not “01”, December as “12”. If do not remember month, fill ‘0’. 访员注意: 用4位数表示年，按照实际的月份填写月。例：1月写作“1”，而不是“01”,12月写作“12”。如果记不住月份，请填入’0’。]

**Soft Check: Prompt for Verification/Correction if Age of Early Retirement is Young, e.g.**

**Prompt if ((FM014_1 +FM014_2 /12)-(CV009_a+CV009_b/12) <45 & CV004==2) | ((FM014_1 +FM014_1 /12)-(CV009_a+CV009_b/12) <50 & CV004==1)**

**Ask if FM011 =2 (early retirement)如果提前退休，问：**

**FM015.** What was the main reason you processed early retirement?  您办提前退休的主要原因是？

1. I have 30 years’ of job experience, which is enough for early retirement. 我的工龄达到了30年，可以提前退休。
2. My work unit belonged to the category of high-risk and hard manual labor and thus was eligible for offering early retirement单位是高风险、高强度的工作，允许提前退休
3. My work unit was restructuring／bankrupt, so it offered early retirement我的工作单位要破产、已经破产或者正在重组因此允许提前退休
4. Due to poor health 健康原因
5. Due to family reason 家庭原因
6. Other其他

**FM016.** Your pre-retirement salary was _______Yuan a month (including bonus and subsidy, et. al).

您退休前的工资，包括奖金和各种补贴每月多少钱？________元/月。

**FM017.** [IWER: If R is unwilling to answer or does not remember, ask unfolding bracket questions here. 访员注意：如果受访者不愿回答或者忘记了，在此处分级展开提问]: 500 /1,000/2,500/5,000/10,000 yuan 元

**FM018.** In what month and year did you start to receive you pension benefits? 您从什么哪年哪月开始领取养老金(退休金)的？_____(FM018_1 )1900..2011Year年_____(FM018_2)0..12月

[IWER: Mark the year using four digits. Take down the month as its actual number. For example, write January as “1” not “01”, December as “12”. If do not remember month, fill ‘0’. 访员注意: 用4位数表示年，按照实际的月份填写月。例：1月写作“1”，而不是“01”,12月写作“12”。如果记不住月份，请填入’0’。]

**FM019.** Are you receiving pension benefits from the government, a previous work unit, or a social insurance agency? (Choose all that apply) 您的养老金（退休金）是从哪里领，政府、单位还是社会保险机构？(可多选)

1. Government 政府
2. work unit 工作单位
3. Social insurance agency 社会保险机构
4. Commercial insurance company 商业保险机构
5. Other 其他

**FM020.** How much were the benefits (including subsidy) when you retired? 您退休的时候，退休金(包括各种补贴)是多少？

____________ Yuan per month元每月

**FM021.** [IWER: If R is unwilling to answer or does not remember, ask unfolding bracket questions here. 访员注意：如果受访者不愿回答或者忘记了，在此处分级展开提问]: 500 /1,000/2,000/3,500/5,000 yuan 元

**FM022.** What is your monthly pension (including subsidy)? 您现在每月领多少退休金(包括各种补贴)？

____________ Yuan 元

**Soft Check: Verify if monthly benefits are low or high, e.g., prompt if FM022< 200 yuan/month**

**FM023.** [IWER: If R is unwilling to answer or does not remember, ask unfolding bracket questions here. 访员注意：如果受访者不愿回答或者忘记了，在此处分级展开提问]: 500 /1,000/2,000/3,500/5,000 yuan 元

**FM024.** How many years of eligible work did you have at the time of retirement? 您退休时按多少年工龄计算？___________0.00..100.00Years年

**Skip to** **FM042 跳至FM042**

**[PROCEDURE: Ask (FM025 – FM029) if FM011 =3 or 4 (internal retirement). 程序：如果FM011 =3 or 4，如果内退，问FM025– FM029]**

**FM025** In what month and year did you take internal retirement? 您是在哪年哪月办理的内退？______ (FM025_1)1900..2011Year年_______(FM025_2) 0..12Month 月

[IWER: Mark the year using four digits. Take down the month as its actual number. For example, write January as “1” not “01”, December as “12”. If do not remember month, fill ‘0’. 访员注意: 用4位数表示年，按照实际的月份填写月。例：1月写作“1”，而不是“01”,12月写作“12”。如果记不住月份，请填入’0’。]

**Soft Check: Prompt for Verification/Correction if Age of Retirement is Young, e.g.**

**Prompt if (FM030_1 +FM030_2 /12)-(CV009_a+CV009_b/12) <45 & CV004==2) | ((FM030_1 +FM030_2 /12)-(CV009_a+CV009_b/12) <50 & CV004==1)**

**FM026.** What was the main reason you processed internal retirement?  您办理内退的主要原因是？

1. 5years less than the legal retirement age 距法定退休年龄不足5年。
2. My work unit was restructuring／bankrupt我的工作单位要破产、已经破产或者正在重组
3. Due to poor health 健康原因
4. Due to family reason 家庭原因
5. Other 其他

**FM027.** Your pre-internal retirement salary was _______Yuan a month everything included. 您内退前的工资、奖金全部加起来是平均每月多少钱?_______元/月

**FM028.** How much was the internal retirement wage (everything included) when you processed internal retirement? 您内退的时候，工资、奖金全部加起来每个月平均能拿到多少？

____________ Yuan per month 元/月

**FM029.** [IWER: If R is unwilling to answer or does not remember, ask unfolding bracket questions here. 访员注意：如果受访者不愿回答或者忘记了，在此处分级展开提问]: 500 /1,000/2,000/3,500 /5,000 yuan 元

**Ask(FM030– FM036) if FM011 =3:如果FM011 =3，问(FM030 – FM036 )：**

**FM030.** In what month and year did you process formal retirement? 您在哪年哪月办理的正式退休手续？______ 1900..2011 (FM030_1)Year年_______0..12 (FM030_2) Month 月

[IWER: Mark the year using four digits. Take down the month as its actual number. For example, write January as “1” not “01”, December as “12”. If do not remember month, fill ‘0’. 访员注意: 用4位数表示年，按照实际的月份填写月。例：1月写作“1”，而不是“01”,12月写作“12”。如果记不住月份，请填入’0’。]

**Soft Check: Prompt for Verification/Correction if Age of Retirement is Young, e.g.**

**Prompt if ((FM030_1 +FM030_2 /12)-(CV009_a+CV009_b/12) <45 & CV004==2) | ((FM030_1 +FM030_2 /12)-(CV009_a+CV009_b/12) <50 & CV004==1)**

**FM031.** How much were the benefits (including subsidy) when you formally retired? 您正式退休的时候，退休金（包括各种补贴）是多少？___________Yuan per month 元/月

**FM032.**  [IWER: If R is unwilling to answer or does not remember, ask unfolding bracket questions here. 访员注意：如果受访者不愿回答或者忘记了，在此处分级展开提问]: 500 /1,000 /2,000/3,500/5,000 yuan 元

**FM033.** Are you receiving pension benefits from the government, a previous work unit, or a social insurance agency? 您的退休金是从哪里领的，政府、单位还是社会保险机构？

- - - 1. Government 政府
      2. Work unit 工作单位
      3. Social insurance agency 社会保险机构
      4. Commercial insurance company 商业保险机构
      5. Other 其他

**FM034.** What is your monthly pension? 您现在每月领多少退休金？

____________ Yuan 元

**Soft Check: Verify if monthly benefits are low or high, e.g., prompt if FM034 < 200 yuan/month or FM034 >5000**

**FM035.** [IWER: If R is unwilling to answer or does not remember, ask unfolding bracket questions here. 访员注意：如果受访者不愿回答或者忘记了，在此处分级展开提问]: 500 /1,000/2,000/3,500/5,000 yuan 元

**FM036.** How many years of eligible work did you have at the time of formal retirement? 您正式退休时计算了多少年工龄？______________ 0.00..100.00Years 年

**Skip to FM042 跳至FM042**

**Ask(FM037 – FM040 ) if FM011=4:如果FM011=4，问 FM037- FM040：**

**FM037.** In what month and year are you going to process formal retirement? 您将会在哪年哪月办理正式退休手续？______ 1900..2011 (FM037_1)Year年_______ 0..12 (FM037_2)Month 月

[IWER: Mark the year using four digits. Take down the month as its actual number. For example, write January as “1” not “01”, December as “12”. If do not remember month, fill ‘0’. 访员注意: 用4位数表示年，按照实际的月份填写月。例：1月写作“1”，而不是“01”,12月写作“12”。如果记不住月份，请填入’0’。]

**Soft Check: Verify if Age of Respondent will be outside the legal retirement range, e.g.,**

**Prompt for verification if ((FM037_1 +FM037_2 /12)-(CV009_a+CV009_b/12)<50 | (FM037_1 +FM037_2 /12)-(CV009_a+CV009_b/12)>55) & CV004==2) | (FM037_1+FM037_2 /12)-(CV009_a+CV009_b/12)<55 | (FM037_1 +FM037_2 /12)-(CV009_a+CV009_b/12)>60) & CV004==1)**

**FM038**  How much are the benefits going to be when you formally retire? 您预计正式退休的第一年，退休金是多少？____________(FM038_1) Yuan per month 元/月 or _________0.00..100.00 (FM038_2) % of salary before retirement 或者退休前工资的_____％

**Soft Check: Verify if monthly benefits are low or high, e.g., prompt if FM038_1 < 200 yuan/month or FM038_2 >5000**

**[Also compare with current wage]**

**FM039.** [IWER: If R is unwilling to answer or does not remember, ask unfolding bracket questions here. 访员注意：如果受访者不愿回答或者忘记了，在此处分级展开提问]: 500 /1,000/2,000/3,500/5,000 yuan 元

**FM040.** How many years of eligible work will you have at the time of retirement? 您正式退休时预计将计算多少年工龄？_____________0.00..100.00Years 年

**FM041.** How many years of eligible work do you currently have? 您现在已经有多少年工龄了？__________ 0.00..100.00years 年

**[ FM041 > FM040 ]**

**FM042.** Did you have a spouse when you processed [preload: normal retirement / early retirement / internal retirement/receding position]? 您办理[正式退休/提前退休/内退/退职]手续时是否有配偶？

1. Yes 是
2. No 否                         → Skip to to FM047 请跳至 FM047

**FM043.** How was your health at the time of your [preload: normal retirement / early retirement / internal retirement/receding position], excellent, very good, good, fair or poor? 您[正式退休/提前退休/内退/退职]时您的身体状况如何？是极好、很好、好、一般还是不好?

1. Excellent 极好
2. Very good 很好
3. Good 好
4. Fair 一般
5. Poor 不好

**FM044.** Had your spouse already processed retirement when you processed [preload: normal retirement / early retirement / internal retirement/receding position]? 您办理[正式退休/提前退休/内退/退职]时配偶是否已经办理了退休手续？

1. Yes 是
2. No 否

**FM045.** What kind of economic activities was your spouse engaged in at the time of your [preload: retirement /receding position]? 您[退休/退职]时配偶在从事哪种经济活动？

1. Employed by another person or company and received a wage 受雇于某公司或个人，领取工资
2. Ran own business 从事个体或者私营经济活动
3. Non-employed and looking for a job 没有工作但是在找工作
4. Non-employed and not looking for a job，or only doing household work 没有工作也没有找工作,或仅仅在家作家务
5. Farming 务农

**FM046.** How was your spouse’s health at the time of your [preload: normal retirement / early retirement / internal retirement/receding position], excellent, very good, good, fair or poor? 您[正式退休/提前退休/内退/退职]时您配偶的身体状况如何？是极好、很好、好、一般还是不好?

1. Excellent 极好
2. Very good 很好
3. Good 好
4. Fair 一般
5. Poor 不好

**FM047.** Was your father alive at the time of your [preload: normal retirement / early retirement / internal retirement/receding position]? 您[正式退休/提前退休/内退/退职]时，您父亲还健在吗？

1. Yes 是
2. No 否 Skip to FM049 请跳至FM049

**FM048.** How about the health of your father at the time of your [preload: normal retirement / early retirement / internal retirement/receding position], excellent, very good, good, fair or poor? 您[正式退休/提前退休/内退/退职]时您父亲的身体状况如何？是极好、很好、好、一般还是不好?

1. Excellent 极好
2. Very good 很好
3. Good 好
4. Fair 一般
5. Poor 不好

**FM049.** Did your mother alive when your [preload: normal retirement / early retirement / internal retirement/receding position]? 您[正式退休/提前退休/内退/退职]时，您母亲还健在吗？

1. Yes 是
2. No 否 Skip to FM051 请跳至FM051

**FM050.** How about the health of your mother at the time of your [preload: normal retirement / early retirement / internal retirement/receding position], excellent, very good, good, fair or poor? 您[正式退休/提前退休/内退/退职]时您母亲的身体状况如何？是极好、很好、好、一般还是不好?

1. Excellent 极好
2. Very good 很好
3. Good 好
4. Fair 一般
5. Poor 不好

**FM051.** How many grandchildren below age 6 did you have at the time of your [preload: normal retirement / early retirement / internal retirement/receding position]? 您[正式退休/提前退休/内退/退职]时有几个六岁以下的孙子女或者外孙子女？_____ __0..50persons 人

[IWER: if none, fill '0'. 访员注意：如果没有请填“0”]

**[PROCEDURE: If R is currently not working（FA001 =2 & FA002 =2 &FA003=2）, ask FM052 . 程序：如果受访者现在不在工作（FA001 =2 & FA002 =2 & FA003 =2），问：FM052**

**FM052.** Did you work after you processed [preload: normal retirement / early retirement / internal retirement/receding position]? We consider any of the following activities to be work: agricultural work, earn a wage, run your own business and unpaid family business work, et. al. Work does not include doing your own housework or doing activities without pay, such as voluntary work.在您办理了[正式退休/提前退休/内退/退职]之后，您是否工作过? 务农、挣工资工作、从事个体、私营经济活动或不拿工资为家庭经营活动帮工都算是工作，但不包括家务劳动、义务的志愿劳动。

- 1. Yes是
  2. No否

**[PROCEDURE: If FM052=1 or If R is currently working, ask FM053). 程序：如果受访者退休后工作过或者现在仍在工作（FM052 =1 or FA001 =1 or FA002 =1or FA003 =1），问：FM053 ]**

**FM053.** After you processed [preload: normal retirement / early retirement / internal retirement/receding position] How long did you start to work again? 在您办理了[正式退休/提前退休/内退/退职]之后，您过了多长时间又开始工作的？

____ 0.00..100.00Years 年 (allow for decimal points允许小数点）

**Skip to FN001 跳至FN001**

［IWER: If R is not working [FA001 =2 & FA002 =2 & FA003 =2], ask the following(FM054 – FM059 )］如果被访者之前回答目前没有工作，提问：

**FM054.** Are you currently engaged in paid small pastime work? 您目前是否从事小的消遣性的工作但是也有收入？

1. Yes 是
2. No 否        → Skip to FN001 请跳至FN001

**FM055.** What kind of pastime job are you engaged in? 您从事哪种消遣性的工作?

_______________________________________________________

**FM056.** When did you start this job? 您从什么时候开始做这项工作的？

___1900..2011（FM056_1） Year年___ 0..12（FM056_2）Month月

[IWER: Mark the year using four digits. Take down the month as its actual number. For example, write January as “1” not “01”, December as “12”. If do not remember month, fill ‘0’. 访员注意: 用4位数表示年，按照实际的月份填写月。例：1月写作“1”，而不是“01”,12月写作“12”。如果记不住月份，请填入’0’。]

**FM057.** How many days per week do you usually work for your pastime job? 您一般每周做几天？An average of _____ days per week 平均每周____ 0..7天

**FM058.** How many hours per week do you usually work at your pastime job? 您一般每周做几个小时？An average of _____ hours per week 平均每周___0.00..168.00小时

**FM059.** What is your monthly income from the pastime work? 您每月能挣多少钱？

______ YUAN 元

[IWER: Mark 0 if there is no net income, and mark 999997 if running a deficit. 访员注意：如果没有净收入填0，如果每月亏损填999997。]

**Skip to FN001 跳至FN001**

**End of Retirement 退休部分结束**

## FN PENSION INSURANCE 养老保险

**[Intro: Next we’ll ask you some questions about your pension insurance. It’s important to access existing pension policy and revise it in the future. ]**

**[引语:下面我们将问一些有关您的养老保险的问题，这些问题对于评估现有的养老政策以及制定将来的养老政策有着重要意义。]**

**FN001.** Are you currently receiving at least one kind of pension as followings? Pension here refers to income from such pension programs as supplemental pension insurance of the firms, residents'pension insurance, rural pension insurance, Urban residents’ pension and commercial pension insurance, and pension subsidy for the oldest old and so on，excluding wages from government and institutions，basic pension insurance provided by the firms. 您现在领取以下一种或几种养老金吗？企业补充养老保险、城乡居民养老保险、农村养老保险（指老农保）、城镇居民养老保险、商业养老保险等项目的养老金，以及高龄老人养老补助（补贴）等，不包括政府和事业单位、企业提供的退休工资。

- 1. Yes 是的，正在领取
  2. No 不是，没有领取 Skip to procedure before FN024 请跳至FN024前的程序控制

F1：（1）“企业补充养老保险”也叫企业年金，是指企业及其职工在依法参加基本养老保险的基础上，自愿建立的补充养老保险制度。

（2）“商业养老保险”是指受访者直接向商业保险公司投保，定期缴纳保险费，从合同约定年龄开始持续、定期地领取养老金的人寿保险，能有效地满足客户的养老需要。

（3）“农村养老保险”是指农村社会养老保险。1992年民政部颁布了《县级农村社会养老保险基本方案》，确定以县为基本单位开展农村社会养老保险。

（4）“城乡居民社会养老保险”指为推进城乡一体化，某些地区将城镇居民社会养老保险和新型农村社会养老保险统筹安排、合并实施，称为“城乡居民社会养老保障制度”。

（5）“城镇居民养老保险”指通过建立城镇居民养老保险个人账户，实行个人缴费和财政补贴相结合的筹资模式，为目前未享受定期养老待遇或其他相关定期养老待遇的城镇居民提供的养老保险。

（6）“高龄老人养老补助（补贴）”是指60岁以上老人享有政策性的优惠，如乘车免费、挂号费免费、参观等免费、部分社区活动免费，如注射流感疫苗等,百岁以上老人才享有月补助金。

**[Show Card 出示卡片23]**

**FN002.** What type(s) of pension do you receive? (choose all that apply) 您的养老金（退休金）来自于以下哪一种或哪几种养老保险项目？（可多选）

[IWER: Read out all the choices. 访员注意：读出所有选项。]

1. Supplemental pension insurance of the firm 企业补充养老保险 Skip to FN003 请跳至FN003
2. Commercial pension 商业养老保险 →Skip to FN006 请跳至FN006
3. Rural pension 农村养老保险 →Skip to FN009 请跳至FN009
4. Residents’ pension 城乡居民社会养老保险 →Skip to FN009 请跳至FN009
5. Urban residents’ pension 城镇居民养老保险→Skip to FN009 请跳至FN009
6. Pension subsidy to the oldest old 高龄老人养老补助（补贴） Skip to FN017 请跳至FN017
7. Other 其他 (FN002_1) Skip to FN020 请跳至FN020

F1：（1）“企业补充养老保险”也叫企业年金，是指企业及其职工在依法参加基本养老保险的基础上，自愿建立的补充养老保险制度。

（2）“商业养老保险”是指受访者直接向商业保险公司投保，定期缴纳保险费，从合同约定年龄开始持续、定期地领取养老金的人寿保险，能有效地满足客户的养老需要。

（3）“农村养老保险”是指农村社会养老保险。1992年民政部颁布了《县级农村社会养老保险基本方案》，确定以县为基本单位开展农村社会养老保险。

（4）“城乡居民社会养老保险”指为推进城乡一体化，某些地区将城镇居民社会养老保险和新型农村社会养老保险统筹安排、合并实施，称为“城乡居民社会养老保障制度”。

（5）“城镇居民养老保险”指通过建立城镇居民养老保险个人账户，实行个人缴费和财政补贴相结合的筹资模式，为目前未享受定期养老待遇或其他相关定期养老待遇的城镇居民提供的养老保险。

（6）“高龄老人养老补助（补贴）”是指60岁以上老人享有政策性的优惠，如乘车免费、挂号费免费、参观等免费、部分社区活动免费，如注射流感疫苗等,百岁以上老人才享有月补助金。

**[PROCEDURE: Ask FN003-FN005 if the respondent is receiving pension benefits from supplemental pension insurance of the firms FN002 =1. 程序：如果受访者正在领取企业补充养老保险金，请问FN003 -FN005 .]**

**FN003.** In what month and year did you start to receive pension benefits from supplement pension insurance of the firms?　您是从哪年哪月开始领企业补充养老保险金的？______(FN003_1)1900..2011Year年_______(FN003_2) 0..12Month 月

F1：“企业补充养老保险”也叫企业年金，是指企业及其职工在依法参加基本养老保险的基础上，自愿建立的补充养老保险制度。

[IWER: Mark the year using four digits. Take down the month as its actual number. For example, write January as “1” not “01”, December as “12”. If do not remember month, fill ‘0’. 访员注意: 用4位数表示年，按照实际的月份填写月。例：1月写作“1”，而不是“01”,12月写作“12”。如果记不住月份，请填入’0’。]

**FN004.** What are your monthly benefits? 您每月领多少钱？

___________ Yuan per month元/月

**FN005.** [IWER: If R is unwilling to answer or does not remember, ask unfolding bracket questions here. 访员注意：如果受访者不愿回答或者忘记了，在此处分级展开提问]: 500 /1,000/2,000/3,500/5,000 yuan 元

**Skip to FN024 if the respondent does not receive money fromother pension programs（FN002_2 =.& FN002_3 =.& FN002_4 =.&FN002_5 =.&FN002_6 =. FN002_7 =.&）如果受访者没有从其他养老保险项目领取养老金（ FN002_2 =.& FN002_3 =.& FN002_4 =.&FN002_5 =.&FN002_6 =. FN002_7 =.&），请跳至FN024 .**

**Ask FN006 -FN008 if the respondent is receiving commercial pension benefits FN002 =2. 如果受访者正在领取商业养老金FN002 =2，问FN006 -FN008 ：**

**FN006.** In what month and year did you start to receive commercial pension benefits?　您是从哪年哪月开始领商业养老金的？______1900..2011（FN006_1） Year年_______ 0..12（FN006_2）Month 月

[IWER: Mark the year using four digits. Take down the month as its actual number. For example, write January as “1” not “01”, December as “12”. If do not remember month, fill ‘0’. 访员注意: 用4位数表示年，按照实际的月份填写月。例：1月写作“1”，而不是“01”,12月写作“12”。如果记不住月份，请填入’0’。]

**FN007.** What is your monthly benefit? 您每月领多少钱？

____________ Yuan per month元/月

**FN008.** [IWER: If R is unwilling to answer or does not remember, ask unfolding bracket questions here. 访员注意：如果受访者不愿回答或者忘记了，在此处分级展开提问]: 500 /1,000/2,000/3,500/5,000 yuan 元

**Skip to FN024 if the respondent does not receive money from other pension programs（FN002_3 =.& FN002_4 =.&FN002_5 =.&FN002_6 =. FN002_7 =.&）如果受访者没有从其他养老保险项目领取养老金（FN002_3 =.& FN002_4 =.&FN002_5 =.&FN002_6 =. FN002_7 =.&），请跳至FN024**

**Ask FN009 -FN014 if the respondent is receiving rural pension benefits FN002 =3 or 4 or 5 如果受访者领取了农村养老保险或城乡居民社会养老保险或城镇居民养老保险FN002 =3 或 4 或 5，请问FN009 – FN014：**

**FN009.** Have you ever contributed to your [preload FN002 ]? 您参加[下载FN002 的答案]缴过费吗？[CAPI: If FN002 =4, prompt: If your residents’ pension was transferred from other pension programs, your contribution to these other programs also counts.如果FN002 =4，提示：如果您的城乡居民养老保险是从其他保险转过来的，为其他养老保险缴的费用也算]

1. Yes 是，缴过
2. No 否，没有 Skip to FN012 请跳至FN012

**FN010.** In what month and year did you start to contribute to your rural pension? 您是从哪年哪月开始缴费的？______(FN010_1) 1900..2011Year年______(FN010_2)0..12Month月

[IWER: Mark the year using four digits. Take down the month as its actual number. For example, write January as “1” not “01”, December as “12”. If do not remember month, fill ‘0’. 访员注意: 用4位数表示年，按照实际的月份填写月。例：1月写作“1”，而不是“01”,12月写作“12”。如果记不住月份，请填入’0’。]

**FN011.** Your annual contribution was____Yuan, annual subsidy from the collective was____yuan, annual subsidy from the government was____Yuan; or your lump sum contribution was____Yuan, lump sum subsidy from the collective was_____Yuan, lump sum subsidy from the government was_____Yuan 您每年缴费____(FN011_1)元，集体每年补贴_____(FN011_2)元，政府每年补贴______(FN011_3)元。或者您总共缴费____(FN011_4)元，集体总共补贴______(FN011_5)元，政府总共补贴_____(FN011_6)元。

**FN012.** In what month and year did you start to receive your [preload FN002]? 您是从哪年哪月开始领取[下载F228 ]？________ (FN012_1)1900..2011Year 年_____(FN012_2)0..12Month 月

[IWER: Mark the year using four digits. Take down the month as its actual number. For example, write January as “1” not “01”, December as “12”. If do not remember month, fill ‘0’. 访员注意: 用4位数表示年，按照实际的月份填写月。例：1月写作“1”，而不是“01”,12月写作“12”。如果记不住月份，请填入’0’。]

**FN013.** How much do you receive now? (as an amount per month?) 您现在每月领多少钱？__________ Yuan per month 元/月

**FN014.** [IWER: If R is unwilling to answer or does not remember, ask unfolding bracket questions here. 访员注意：如果受访者不愿回答或者忘记了，在此处分级展开提问]: 500 /1,000/2,000/3,500/5,000 yuan 元

**Ask FN015 -FN016 if the respondent is receiving residents’ pension FN002 =4.如果受访者参加了城乡居民养老保险FN002 =4，请问FN015 – FN016 ：**

**FN015.** Was your residents’ pension transferred from other pension insurances like rural pension and basic pension of the firms? 您参加的城乡居民养老保险是从农村养老保险、企业基本养老保险或其他养老保险转过来的吗？

（1）Yes 是的

（2）No 不是 Skip FN016 跳过FN016

F1：（1）“农村养老保险”是指农村社会养老保险。1992年民政部颁布了《县级农村社会养老保险基本方案》，确定以县为基本单位开展农村社会养老保险。

（2）“企业基本养老保险”：是指受访者参加社会基本养老保险制度，达到退休年龄并符合领取条件后，领取到社会养老保险金。

**FN016.** Which of the following pension program was your residents’ pension transferred from? 您参加的城乡居民养老保险是从以下哪种养老保险转过来的？

1. Rural pension 农村养老保险
2. Basic pension of the firms 企业基本养老保险
3. Other 其他_________(FN016_1)

F1：（1）“农村养老保险”是指农村社会养老保险。1992年民政部颁布了《县级农村社会养老保险基本方案》，确定以县为基本单位开展农村社会养老保险。

（2）“企业基本养老保险”：是指受访者参加社会基本养老保险制度，达到退休年龄并符合领取条件后，领取到社会养老保险金。

**[PROCEDURE: Skip to FN024 if the respondent does not receive money fromother pension programs（FN002_6 =. FN002_7 =.&）. 程序：如果受访者没有从其他养老保险项目领取养老金（FN002_6 =.& FN002_7 =.），请跳至FN024 ]**

**[PROCEDURE:Ask FN017 -FN019 if the respondent is receiving pension subsidy for the oldest old FN002 =6. 程序：如果受访者正在领取高龄老人养老补贴FN002 =6，请问FN017 – FN019 ]**

**FN017.** In what month and year did you start to receive the pension subsidy for the oldest old? 您是从哪年哪月开始领取高龄老人养老补贴的？________ (FN017_1)1900..2011Year 年_____(FN017_2 )0..12Month 月

F1：“高龄老人养老补助（补贴）”指60岁以上老人享有政策性的优惠，如乘车免费、挂号费免费、参观等免费、部分社区活动免费，如注射流感疫苗等,百岁以上老人才享有月补助金。

[IWER: Mark the year using four digits. Take down the month as its actual number. For example, write January as “1” not “01”, December as “12”. If do not remember month, fill ‘0’. 访员注意: 用4位数表示年，按照实际的月份填写月。例：1月写作“1”，而不是“01”,12月写作“12”。如果记不住月份，请填入’0’。]

**FN018.** How much do you receive now? 您现在每月领多少钱？____ ______Yuan per month 元/月

**FN019.** [IWER: If R is unwilling to answer or does not remember, ask unfolding bracket questions here. 访员注意：如果受访者不愿回答或者忘记了，在此处分级展开提问]: 500 /1,000/2,000/3,500/5,000 yuan 元

**[PROCEDURE:Skip to FN024 if the respondent does not receive money fromother pension programs（FN002_7 =.）. 程序：如果受访者没有从其他养老保险项目领取养老金（FN002_7 =.），请跳至FN024 ]**

**[PROCEDURE: Ask FN020 -FN023 if the respondent is receiving other pension benefits FN002 =7 . 程序：如果受访者参加了其他养老保险项目FN002=7，询问FN020 – FN023 ]**

**FN020.** You just told us you are receiving benefits from other pension program, what is the name of this pension program? 您刚才告诉我们说您正在从其他养老保险项目中领取养老金（退休金），这个项目的名称是__ ______

**FN021.** In what month and year did you start to receive this pension benefits? 您是从哪年哪月开始领取这份养老金（退休金）的？________(FN021_1) 1900..2011Year 年____(FN021_2)0..12Month 月

[IWER: Mark the year using four digits. Take down the month as its actual number. For example, write January as “1” not “01”, December as “12”. If do not remember month, fill ‘0’. 访员注意: 用4位数表示年，按照实际的月份填写月。例：1月写作“1”，而不是“01”,12月写作“12”。如果记不住月份，请填入’0’。]

**FN022.** How much do you receive now? 您现在每月领多少钱？________Yuan per month 元/月

**FN023.** [IWER: If R is unwilling to answer or does not remember, ask unfolding bracket questions here. 访员注意：如果受访者不愿回答或者忘记了，在此处分级展开提问]: 500 /1,000/2,000/3,500/5,000 yuan 元

**Skip to FN024 请跳至FN024**

**[PROCEDURE: If FB011 =2，ask FN024 -FN037 , 程序：如果FB011 =2 （受访者没有退休），询问FN024-FN037 ]**

**FN024.** Are you enrolled in pension program of the government and institutions or basic pension of the firms? 您是否参加了政府和事业单位的养老保险或者企业基本养老保险？

(1) Yes, pension program of the government and institutions 是的，参加了政府和事业单位的养老保险

(2) Yes, basic pension insurance of the firms 是的，参加了企业基本养老保险

(3) No 否，都没有参加 Skip to FN038 请跳至FN038

F1：“政府和事业单位的养老保险”指受访者从政府和事业单位领取的退休金。目前，我国大部分政府机构和事业单位，都还没有实行社会养老保险制度，仍实行退休养老制度。

**FN025.** From which of the following work units did you get the pension insurance you just told us[preload “pension program of government and institutions” if FN024 =1; or preload “Basic pension insurance of the firms” if FN024 =2]?给您提供这份养老保险[如果FN024 =1,下载“政府和事业单位养老保险”；如果FN024 =2,下载“企业基本养老保险”]的单位是下面的哪一个？

[[IWER: Choose “(4) None of the above” if the respondent has no work unit 访员注意：如果受访者没有单位，请选择“（4）都不是”并继续]

1. Current work unit [preload FD003 ] FD003 中提到的现在的工作单位[下载FD003 ] Skip to FN029 请跳至FN029
2. Last work unit [preload FL005 ] FL005 中提到的上一份工作的单位[下载FL005 ] Skip to FN029 请跳至FN029
3. The work unit that processed retirement for the respondent [preload FM002 ] FM002 中提到的办理退休手续的单位[下载FM002 ] Skip to FN029 请跳至FN029
4. None of the above 都不是

**If answer to FN025 =4 is no, then ask FN026 – FN028: 如果FN025 =4回答不是，请问FN026 -FN028 ：**

**FN026.** What is the name of the unit that provides you this pension insurance? 给您提供这份养老保险的单位的名称是什么?____ __________________________________

**FN027.** What was the type of the unit that provides you the pension insurance? 这个给您提供养老保险的单位是哪种类型的？

(1) Government 政府部门

(2) Institutions 事业单位

(3) NGO 非营利机构（如社团、协会、学会等）

(4) Firm 企业

(5) Individual个体或者私营企业主

(6) Other 其他

**FN028.** In which province and county/city is this work unit located? 这个给您提供养老保险的单位在哪个省？_______(FN028_1)Province省/自治区/直辖市_______(FN028_2) city市_______(FN028_3) county县

**Ask FN029 -FN031 only if the respondent have participated in basic pension insurance of the firms (FN024 =2)** 如果参加了企业基本养老保险，请回答下列问题FN029 -FN031

**FN029.** In what month and year did you start to participate in the basic pension insurance of the firms through this work unit? 您是从哪年哪月开始在这个单位参加企业基本养老保险的？_____(FN029_1)1900..2011Year年_____(FN029_2)0..12Month月

F1：“企业基本养老保险”是指受访者参加社会基本养老保险制度，达到退休年龄并符合领取条件后，领取到社会养老保险金。

[IWER: Mark the year using four digits. Take down the month as its actual number. For example, write January as “1” not “01”, December as “12”. If do not remember month, fill ‘0’. 访员注意: 用4位数表示年，按照实际的月份填写月。例：1月写作“1”，而不是“01”,12月写作“12”。如果记不住月份，请填入’0’。]

**FN030.** Before the above mentioned time, have you ever participated in basic pension insurance of the firms through other work unit for more than ten years? 在此之前，您有没有在其他地方参加企业基本养老保险超过10年？

（1）Yes 有

（2）No 没有 Skip to FN032 请跳至FN032

F1：“企业基本养老保险”是指受访者参加社会基本养老保险制度，达到退休年龄并符合领取条件后，领取到社会养老保险金。

**FN031.** Among all of work units that you have participated in basic pension insurance of the firms for more than 10 years, in which province was the lastest work unit located? 在您之前参加的所有超过10年的企业基本养老保险中，最近的一次是在哪个省参加的？_____ Province省/自治区/直辖市

F1：“企业基本养老保险”是指受访者参加社会基本养老保险制度，达到退休年龄并符合领取条件后，领取到社会养老保险金。

**FN032.** For how many years altogether have you been included in this program? [Include years with other employers if the same plan.] 到现在为止您可以用来计算养老金（退休金）的工龄一共有多少年？[不管雇主是否相同，只要能够累积计算工龄，都应算进去。]_______ 0.00..100.00Years 年

**FN033.** For how many years altogether will you have been included in this program when you retire? [Include years with other employers if the same plan.] 您估计，到您退休时您可以用来计算养老金（退休金）的工龄一共有多少年？[不管雇主是否相同，只要能够累积计算工龄，都应算进去。] ________0.00..100.00Years 年

**FN034.** Will this be enough years to receive pension? 这工龄足够您从上述养老保险项目中领取养老金吗？

1. Yes 是 →Skip to FN036 请跳至FN036
2. No 否 →Skip to FN035 请跳至FN035

**FN035.** What do you plan to do? 那您计划怎么办？

1. I will pay the remaining premiums all in one payment at retirement to qualify for pension 退休的时候，我会一次性支付剩余年限的保险金，这样就能领取养老金了。 →Skip to FN036 请跳至FN036
2. I will receive a one-time payment at retirement and not get pension 退休的时候，我会得到一次性的补偿，以后就没有养老金了。
3. I will not receive pension 我将拿不到养老金。

If R answered yes to pension [FN034 =1 or FN035 =1], ask:如果被访者退休的时候，能领养老金，请回答：

**FN036.** About how much do you expect your benefits to be? (as a percentage of your pay at retirement, or as an amount per month or year?) 您预期领多少钱的养老金？ 可以是退休前工资的百分比，也可以是每月多少钱。

_________ （FN036_1）Yuan per month元/月

Or 或_________ 0.00..100.00（FN036_2）% of final pay 退休前工资

**FN037.** [IWER: If R is unwilling to answer or does not remember, ask unfolding bracket questions here. 访员注意：如果受访者不愿回答或者忘记了，在此处分级展开提问]: 500 /1,000 /2,000/3,500/5,000 yuan 元

**Ask all respondents, 询问所有受访者**

**FN038.** Are you enrolled in at least one kind of pension programs as lists? Pension programmes here includes supplemental pension of the firms, commercial pension insurance, rural pension, residents’ pension and so on，excluding pension insurance of government and institutions, basic pension of the firms.

您是否参加了以下一种或几种养老保险项目？包括企业补充养老保险，商业养老保险，农村养老保险（指老农保），城乡居民养老保险等，不包括府和事业单位的养老保险，企业基本养老保险。

(1) Yes 是的，参加了

(2) No 否，没有参加 Skip to FN071 请跳至FN071

F1：（1）“企业补充养老保险”也叫企业年金，是指企业及其职工在依法参加基本养老保险的基础上，自愿建立的补充养老保险制度。

（2）“商业养老保险”是指受访者直接向商业保险公司投保，定期缴纳保险费，从合同约定年龄开始持续、定期地领取养老金的人寿保险，能有效地满足客户的养老需要。

（3）“农村养老保险”是指农村社会养老保险。1992年民政部颁布了《县级农村社会养老保险基本方案》，确定以县为基本单位开展农村社会养老保险。

（4）“企业基本养老保险”是指受访者参加社会基本养老保险制度，达到退休年龄并符合领取条件后，领取到社会养老保险金。

**FN039.** What type(s) of pension insurance do you have? (Choose all that apply) 您参加了以下哪一种或者哪几种养老保险？（可多选）

[IWER: Read out all the choices. 访员注意：读出所有选项。]

1. Supplemental pension insurance of the firm 企业补充养老保险Skip to FN040 请跳至FN040
2. Commercial pension 商业养老保险Skip to FN050 跳至FN050
3. Rural pension 农村养老保险 Skip to FN060 跳至FN060
4. Residents’ pension 城乡居民养老保险Skip to FN060 跳至FN060
5. Urban residents’ pension 城镇居民养老保险Skip to FN060 跳至FN060
6. Other pension 其他保险 Skip to FN067 请跳至FN067

F1：（1）“企业补充养老保险”也叫企业年金，是指企业及其职工在依法参加基本养老保险的基础上，自愿建立的补充养老保险制度。

（2）“商业养老保险”是指受访者直接向商业保险公司投保，定期缴纳保险费，从合同约定年龄开始持续、定期地领取养老金的人寿保险，能有效地满足客户的养老需要。

（3）“农村养老保险”是指农村社会养老保险。1992年民政部颁布了《县级农村社会养老保险基本方案》，确定以县为基本单位开展农村社会养老保险。

（4）“城乡居民社会养老保险”指为推进城乡一体化，某些地区将城镇居民社会养老保险和新型农村社会养老保险统筹安排、合并实施，称为“城乡居民社会养老保障制度”。

（5）“城镇居民养老保险”指通过建立城镇居民养老保险个人账户，实行个人缴费和财政补贴相结合的筹资模式，为目前未享受定期养老待遇或其他相关定期养老待遇的城镇居民提供的养老保险。

**Supplementary enterprise pension 企业补充养老金**

**[PROCEDURE: If FN039 =1 ask FN040 –FN049. 程序：如果FN039 =1，提问FN040 –FN049 。]**

**FN040 .** Next are questions about your supplementary enterprise pension. What type of retirement pension plan is/was your employer’s supplement pension? 下面是关于您的企业补充养老保险的一些问题。您的单位选择的补充养老金属于哪种类型？

1. Defined Benefit (DB) Retirement Pension 收益确定型退休金
2. Defined Contribution (DC) Retirement Pension 缴费确定型退休金

F1：“企业补充养老保险”也叫企业年金，是指企业及其职工在依法参加基本养老保险的基础上，自愿建立的补充养老保险制度。

| DB Retirement Pension Plan: A worker's retirement pension is determined in advance and the amount paid by the user shall change based on how well the savings are managed. 收益确定性退休金计划：预先决定员工的养老金，使用者所支付的金额根据储蓄管理情况而定。 |
| --- |
| DC Retirement Pension Plan: The amount paid by the user is determined in advance and the retirement pension paid to a worker shall change based on how well the savings are managed. 缴费确定性退休金计划：预先决定使用者支付的金额，员工领取的退休金根据储蓄管理情况而定。 |

**FN041.** For how many years altogether have you been included in this plan? [Include years with other employers if the same plan.] 您参加该保障制度多少年了？[包括受雇不同雇主但享受同一计划的年度。]______ 0.00..100.00 Years 年

**FN042.** At what age do you expect to start receiving benefits from this plan? 您预期什么时候开始从这份保险中领取保险金？

1. At age在 _______0..120 (FN042_1)岁 or in 或在______0..120 (FN042_2) years 年之后
2. I do not expect receiving these benefits because I have received cash settlements 领取不到保险金，因为已被买断 → Skip to FN049 请跳至FN049
3. I do not expect receiving these benefits because I have lost benefits 领取不到保险金，因为保险已经中断。 → Skip to FN050 请跳至FN050
4. Other 其他 → Skip to FN050 请跳至FN050

**FN043.** What is the combined monthly contribution from you and your employer? Of which, how much do you pay? 每个月您和雇主一共缴纳多少钱？_______ （FN043_1）Yuan 元；

其中，您个人支付部分是多少？_____ 0.00..100.00（FN043_2）%

**If FN040 =1ask:如果FN040 =1，问：**

**FN044.** For a DB plan, do you know how much you are entitled to at (age in EP226)? 如果是收益确定性退休金计划，[开始领退休金的年龄，preload EP226]岁时，您知道每月可以领取多少钱？

_________(FN044_1) Yuan per month 元/月

Or 或_______0.00..100.00 (FN044_2) % of retirement wage %退休时的工资

Or 或_________ (FN044_3)Yuan元 （Lump sum amount 一次结清）

**FN045.** [IWER: If R is unwilling to answer or does not remember, ask unfolding bracket questions here. 访员注意：如果受访者不愿回答或者忘记了，在此处分级展开提问]: 500 /1,000/2,000/3,500/5,000 yuan 元

**Skip to FN050 . 跳至FN050 .**

**If FN040 =2 ask: 如果FN040 =2，问：**

**FN046.** For a DC plan, have you ever checked your account balance? 如果是缴费确定型退休金计划，您有没有查看过账户余额？

1. Yes 有， ______ （FN046_1）Yuan元 in ______ 1900..2011（FN046_2）Year 年_______0..12（FN046_3） Month月

[IWER: Mark the year using four digits. Take down the month as its actual number. For example, write January as “1” not “01”, December as “12”. If do not remember month, fill ‘0’. 访员注意: 用4位数表示年，按照实际的月份填写月。例：1月写作“1”，而不是“01”,12月写作“12”。如果记不住月份，请填入’0’。]

1. No 没有

**FN047.** What is the earliest age at which you could leave this employer and start to receive pension benefits? 您最早可以在多大年纪时离开这家单位，并开始领取这项养老金？ ________ 45..120Years old 岁

**FN048 .** By how much would your pension be reduced from full benefits if you left this job at (AGE IN FN047 )? 如果您[preload FN047 ]岁时离开现在的工作，您可领取的养老金会比预期的全额养老金减少多少？By ________ 0.00..100.00（FN048_1）% or 或 _______ （FN048_2）Yuan 元

**Skip to FN050 请跳至FN050**

**FN049.** How much cash settlements did you receive? 您买断时拿了多少钱？

_______________ Yuan 元

**Skip to FN080 if the respondent participates in no other pension programs (FN039_2 =.&FN039_3 =.&FN039_4 =.&FN039_5 =.&FN039_6 =.)如果受访者没有参加其他养老保险(FN039_2 =.&FN039_3 =.&FN039_4 =.&FN039_5 =.&FN039_6 =.)，请跳至FN080**

**Commercial Pension 商业养老保险**

**[PROCEDURE: If FN039 =2 ask FN050 -FN059 . 如果FN039 =2，提问FN050 -FN059]**

**FN050 .** Next are questions about your Commercial Pension. Who paid for the commercial pension insurance? 下面是关于您的商业养老保险的一些问题。谁给您买的商业养老保险？

1. Myself 我自己
2. My employer 我的雇主
3. My family or relative 我的家人或亲戚
4. Other person 其他人

F1：“商业养老保险”是指受访者直接向商业保险公司投保，定期缴纳保险费，从合同约定年龄开始持续、定期地领取养老金的人寿保险，能有效地满足客户的养老需要。

**FN051.** When did you start paying for the commercial pension? 您什么时候开始购买这商业养老保险的？

___（FN051_1） 1900..2011Year年___0..12（FN051_2） Month月

F1：“商业养老保险”是指受访者直接向商业保险公司投保，定期缴纳保险费，从合同约定年龄开始持续、定期地领取养老金的人寿保险，能有效地满足客户的养老需要。

[IWER: Mark the year using four digits. Take down the month as its actual number. For example, write January as “1” not “01”, December as “12”. If do not remember month, fill ‘0’. 访员注意: 用4位数表示年，按照实际的月份填写月。例：1月写作“1”，而不是“01”,12月写作“12”。如果记不住月份，请填入’0’。]

**FN052.** How do you contribute to the commercial pension? 以什么样的形式交纳保险费?

(1) Annual payment 按年

(2) Lump sum amount一次性缴费 Skip to FN055 请跳至FN055

**FN053.** You contribute _______yuan/ year to the commercial insurance ？您每年需要交纳多少保险费？_______Yuan/ year元/年

**FN054.** How many years do you need to pay? 您需要缴几年保险费?_________Years年

**Soft Check: Prompt for verification if greater than a legal maximum**

**[PROCEDURE: Skip FN055 if FN052 =1. 程序：如果FN052 =1, 跳过FN055]**

**FN055.** How much premium do you need to pay in total? 您一共需要交纳多少保险费？_________Yuan元

**FN056.** How do you receive the pension? 保险费的领取方式？

1. Lump sum amount一次性领取 SkipFN057 and FN058 请跳过FN057 -FN058
2. Yearly 年领
3. Monthly 月领

**If FN056 =2 ask FN057**

**FN057.** How much do you expect to receive _______ yuan/ year after your retirement? 您预期退休后每年能领取多少保险费？ _________ Yuan/year元/年

**Soft Check: Prompt for verification if low or high, e.g. FN057 < 1200 per year or FN057 > 60000 per year**

**If FN056 =3 ask FN058**

**FN058.** How much do you expect to receive _______ yuan/month in the future? 您预期以后每月能领取多少养老金？ _________ Yuan/ month元/月

**Soft Check: Prompt for verification if low or high, e.g. FN058 < 100 per month or FN058 > 5000 per year**

**FN059.** How much do you expect to receive _______ yuan in total? 您预期领取的养老金总额能达到多少？ ________ Yuan元

**Skip to FN080 if the respondent participates in no other pension programs (FN039_3 =.&FN039_4 =.&FN039_5 =.&FN039_6 =.)如果受访者没有参加其他养老保险(FN039_3 =.&FN039_4 =.&FN039_5 =.&FN039_6 =.)，请跳至FN080**

**[PROCEDURE: If FN039 =3 or 4 or 5 ask FN060 -FN064. 如果FN039 =3或4或5，提问FN060-FN064 ]**

**FN060.** In what month and year did you start to pay for your [preload FN039]? 您是从哪年哪月开始为[下载FN039的答案]缴费的？________(FN060_1)1900..2011 Year 年____(FN060_2)0..12Month月

[IWER: Mark the year using four digits. Take down the month as its actual number. For example, write January as “1” not “01”, December as “12”. If do not remember month, fill ‘0’. 访员注意: 用4位数表示年，按照实际的月份填写月。例：1月写作“1”，而不是“01”,12月写作“12”。如果记不住月份，请填入’0’。]

**FN061.** Your annual contribution is _____ Yuan, annual subsidy from the collective is ______ Yuan, annual subsidy from the government is ____ Yuan您参加这份养老保险每年自己缴费 （FN061_1 ）元，集体每年补贴 （FN061_2）元；政府每年补贴___(FN061_3 )元

**FN062.** When do you expect to receive pension？ 您预期在多大年龄享用该保险？

At age 在 _______45..120（FN062_1）years old岁 or in 或在______0.00..100.00（FN062_2） years 年之后

**Soft Check: Prompt or Verify if FN062_1 <50 or FN062_2 >60**

**FN063.** About how much do you expect your benefits to be? (as an amount per month or year or a lump sum?) 您预期自己能领多少钱？

____________ （FN063_1）Yuan per month元/月

Or 或_________ （FN063_2）Yuan 元 （Lump sum amount 一次结清）

**[Soft Check: Prompt or Verify if These Benefits are Low or High, e.g., FN063_1 <100 | FN063_1 >5000 per month.]**

**FN064.** [IWER: If R is unwilling to answer or does not know, ask unfolding bracket questions here. 访员注意：如果受访者不愿回答或者不知道，请回答：] 250 / 500/ 1000/ 2000

**Ask FN065 and FN066 if the respondent is enrolled in residents’ pension FN039 =4 如果受访者参加了城乡居民养老保险FN039 =4，请回答FN065 和FN066**

**FN065.** Was your residents’ pension transferred from other pension insurances like rural pension and basic pension of the firms? 您参加的城乡居民养老保险是从农村养老保险、企业基本养老保险或其他养老保险转过来的吗？

(1) Yes 是的

(2) No 不是 Skip FN066 跳过FN066

F1：（1）“农村养老保险”是指农村社会养老保险。1992年民政部颁布了《县级农村社会养老保险基本方案》，确定以县为基本单位开展农村社会养老保险。

（2）“企业基本养老保险”：是指受访者参加社会基本养老保险制度，达到退休年龄并符合领取条件后，领取到社会养老保险金。

**FN066.** Which of the following pension program was your residents’ pension transferred from? 您参加的城乡居民养老保险是从以下哪种养老保险转过来的？

(1) Rural pension 农村养老保险

(2) Basic pension insurance of the firms 企业基本养老保险

(3) Other 其他__________(FN066_1)

F1：（1）“农村养老保险”是指农村社会养老保险。1992年民政部颁布了《县级农村社会养老保险基本方案》，确定以县为基本单位开展农村社会养老保险。

（2）“企业基本养老保险”：是指受访者参加社会基本养老保险制度，达到退休年龄并符合领取条件后，领取到社会养老保险金。

**Skip to FN080 if the respondent participates in no other pension programs (FN039_6 =.) 如果受访者没有参加其他养老保险 (FN039_6 =.)，请跳至FN080**

**Ask FN067 -FN068 if the respondent is enrolled in other pension program FN039 =6 如果受访者参加了其他养老保险FN039 =6，请回答FN067 -FN068**

**FN067 .** You just told us you are receiving benefits from other pension program, what is the name of this pension program? 您刚才告诉我们说您参加了其他养老保险项目，这个项目的名称是________

**FN068.** When do you expect to receive pension 您预期在多大年龄享用该保险？

At age 在 _______(FN068_1)45..120岁 or in 或在______(FN068_2)years 年之后。

**FN069.** About how much do you expect your benefits to be? (as an amount per month or year or a lump sum?) 您预期自己能领多少钱？

____________(FN069_1) Yuan per month元/月

Or 或_________ (FN069_2)Yuan 元 （Lump sum amount 一次结清）

**FN070.** [IWER: If R is unwilling to answer or does not know, ask unfolding bracket questions here. 访员注意：如果受访者不愿回答或者不知道，请回答：] 250 / 500/ 1000/ 2000

**New Rural Social Pension Insurance 新型农村社会养老保险**

**FN071.** Do you participate in the New Rural Social Pension Insurance program? 您是否参加了新型农村社会养老保险？

1. Yes是 →Skip to FN073 请跳至FN073

（2） No否

**[Procedure: Ask FN072 if FN071 =2, and then end this part 程序：如果FN071=2，请提问FN072，然后结束新型农村社会养老保险部分]**

**FN072.** Why don’t you participate in the New Rural Social Pension Insurance program? (Circle all that applies) 您没有参加新型农村社会养老保险的原因是？（可多选）

1. The New Rural Social Pension has not been introduced in my local area本地还没有开展新型农村社会养老保险(FN072_1)
2. I lack money没有钱(FN072_2)
3. I am not satisfied with the benefits because it isn’t cost-effective. 我对待遇水平不满意，缴费参加该保险是不划算的。(FN072_3)
4. The benefits are poor, and mean nothing to my life. 待遇水平低，对我的生活没有什么意义(FN072_4)
5. Application and payment arrangements make it inconvenient to participate 申请办理或缴费不方便(FN072_5)
6. The mechanism for making contributions is not justifiable. 缴费设计不合理(FN072_6)
7. I do not have a local Hukou. 我没有本地户口(FN072_7)
8. I am already covered by other social pension insurance. 我已经参加了其他社会养老保险项目，不能重复参加(FN072_8)
9. Other 其他(FN072_9)

**→Skip to FN080 请跳至FN080**

**FN073.** In what month and year were you first covered by New Rural Social Pension Insurance? 您是从什么时候加入新型农村社会养老保险（新农保）的？___(FN073_1)2008..2011Year 年___(FN073_2)0..12Month 月

[IWER: Mark the year using four digits. Take down the month as its actual number. For example, write January as “1” not “01”, December as “12”. If do not remember month, fill ‘0’. 访员注意: 用4位数表示年，按照实际的月份填写月。例：1月写作“1”，而不是“01”,12月写作“12”。如果记不住月份，请填入’0’。]

**FN074.** How do you contribute to New Rural Pension Insurance? 您是以什么方式缴费的？

（1） Annual payment 按年 ___(FN074_1) Yuan/Year 元/年

（2） Lump sum amount 一次性缴费___(FN074_2)Yuan元，equals to annual payment等同于缴费FN074_3___ Yuan/Year元/年 **→** Skip to FN076 ，请跳至FN076

（3） I don’t need to pay myself. I am over 60 years old, and I am covered through my children’s participation in the New Rural Social Pension Insurance. 自己超过60岁不用缴费，但符合条件的子女已纳入新型农村社会养老保险 **→**Skip to FN077 ，请跳至FN077

**FN075.**  How many years do you need to pay to receive benefits? 您需要缴几年保险费? __Years年

**FN076.** Who pays for your participation in New Rural Social Pension Insurance? 谁为您的新农保缴费的?

(1) Myself 我自己

(2) My children 我的孩子

(3) Other family member or relative 其他家人或亲戚

(4) Others其他人

**FN077.** Did you start to receive pension benefit? 您是否已经领取了新型农村社会养老保险（新农保）发放的养老金？

1. Yes 是

2. No 否 →Skip to FN080 ，请跳至FN080

**FN078.** In what month and year did you start to receive pension benefits? 您是从哪年哪月开始领取领取新型农村社会养老保险待遇的？___(FN078_1)2008..2011Year 年___(FN078_2)0..12Month 月

[IWER: Mark the year using four digits. Take down the month as its actual number. For example, write January as “1” not “01”, December as “12”. If do not remember month, fill ‘0’. 访员注意: 用4位数表示年，按照实际的月份填写月。例：1月写作“1”，而不是“01”,12月写作“12”。如果记不住月份，请填入’0’。]

**FN079.** How much do you receive every month? 您每月能领取多少养老金？ ____ Yuan/ month元/月

**Ask all R 所有人回答：**

**FN080.** Whom do you think you can rely on for old-age support？如果您将来老了干不动工作了，您认为您最主要依靠什么养老？

1. Children 子女
2. Savings 储蓄
3. Pension or retirement salary 养老金或退休金
4. Commercial pension insurance 商业养老保险
5. Other 其他

F1：“商业养老保险”是指受访者直接向商业保险公司投保，定期缴纳保险费，从合同约定年龄开始持续、定期地领取养老金的人寿保险，能有效地满足客户的养老需要。

**End of Pension 养老金部分结束**

**FN081.** IWER: How often did the respondent receive assistance in answering section Section E－Work? [IWER: If it is answered by a proxy, please record the respondent’s reaction. ]受访者填写E部分问卷时是否求助？[访员注意：如果是协助回答，请记录受访者的反应。]

(1) Never 从未

(2) A few times 偶尔几次

(3) Most or all of the time 大多数或者全部时间

(4) The section was completed by a proxy respondent（the respondent is absent） 受访者不在场，完全请人代填 → Skip to FN082 请跳至FN082

**FN082.** [IWER: What is the proxy’s relationship to R? If unknown, please ask the proxy. 访员注意：代填问卷的人和受访者是什么关系。如果不清楚，请问代填者。]

What is your relationship to R? 您和受访者是什么关系？

(1) Spouse 配偶

(2) Mother 母亲

(3) Father 父亲

(4) Mother-in-law 岳母/婆婆

(5) Father-in-law 岳父/公公

(6) Sibling 兄弟姐妹

(7) Brother-in-law, sister-in-law 姐夫妹夫/嫂子弟媳

(8) Child 孩子

(9) Spouse of child 孩子的配偶

(10) Grandchild 孙子女

(11) Other relative 其他亲戚

(12) Helper or other non-relative 帮忙的人或者其他非亲属

**FN083.**[IWER: Please record the reason for proxy 访员注意：请记录代答的原因。]

What is the main reason for proxy（the respondent is absent）？受访者不在场，完全请人代填的主要原因是什么？

（1）The respondent has serious physical handicaps 受访者有严重身体障碍

（2）The respondent has serious mental handicaps 受访者有严重精神障碍

（3）The respondent has rejected this interview. 受访者拒访

（4）Other . 其他 (FN083_1)

# **G &H INCOME, EXPENDITRUES AND ASSETS 收入、支出与资产**

## G1 VIGNETTS ON INCOME 情景选择题

**[For main respondent only FA006---FA008 ] The following vignette questions are on subjective inequality [仅针对主要受访者询问G001 ---G003 ]下面是关于主观判断偏差的一些问题:**

**[Show Card 出示卡片24]**

**G001.** Mr. Wang owns an apartment in a large city. He lives with his wife and father, who is retired. He and his wife earn 3000 yuan per month. Overall, how would you rate the standard of living of Mr. Wang's family?  Is it very high, relatively high, average, relatively poor or poor? 王先生在大城市有一套房子。他和妻子以及退休的父亲一起住。他和妻子每月挣3000元。总体来说，您怎么评价王先生家的生活水平？是非常高、偏上、中等、偏下还是贫困？

1. Very high 非常高
2. Relatively high 偏上
3. Average 中等
4. Relatively poor 偏下
5. Poor 贫困

**G002.** Mr. Zhang works as a migrant and earns 1000 yuan per month.His wife and father live in a rural village. Their land can produce 2000kg of grain each year, and they also raise 2 pigs.

Overall, how would you rate the standard of living of Mr. Wang's family? Is it very high, relatively high, average, relatively poor or poor? 张先生是个外来务工人员，每月挣1000元。他的妻子和父亲都在农村住。他们家的地每年能产2000公斤粮食，他们还养了两头猪。总体来说，您怎么评价王先生家的生活水平？是非常高、偏上、中等、偏下还是贫困？

1. Very high 非常高
2. Relatively high 偏上
3. Average 中等
4. Relatively poor 偏下
5. Poor 贫困

**G003.** Overall, how would you rate your own standard of living? Is it very high, relatively high, average, relatively poor or poor? 总体来说，您怎么评价您自己家的生活水平？是非常高、偏上、中等、偏下还是贫困？

1. Very high 非常高
2. Relatively high 偏上
3. Average 中等
4. Relatively poor 偏下
5. Poor 贫困

## G2 HOUSEHOLD INCOME AND EXPENDITURES 家户收入与支出

[IWER: Except for Part 1_1, this section is asked of the financial respondent, from questionCV031 Do not allow a proxy respondent to answer the entire section. 除了Part 1_1，其余部分要问财务受访者，不允许完全请别人代答。财务受访者是最了解家庭财务（收入、支出和资产）状况的人。其姓名和编码可以从CV031 题中获得。]

### Part 1: Household Wage Income and Individual-based transfers 家户工资收入和个人获得的转移收入

**Part 1_1: Main Respondent and Spouse’s Wage Income and Individual-based transfers 主要受访者及其配偶的工资收入和个人获得的转移收入**

**[IWER: Please conduct Part 1_1 when the main respondent and spouse are at home. Don’t allow a proxy to complete the part. 访员注意：当主要受访者及其配偶在场时，提问Part 1_1问卷。这部分不允许请人完全代填。]**

**GA001 .** Did you receive any wage and bonus income in the past year? 过去一年，您有没有领工资（包括奖金，不包括退休工资）？

1. Yes 有

（2）No 没有 skip to GA003 _跳到GA003

**GA002.**  How much did you receive last year? ____yuan or F001_b_1 ____yuan/month

过去一年一共领了多少钱？______元 或每个月领多少钱？__ ____ (GA002_1)元

Wage income: yuan [soft check >200,000] or yuan/month [soft check>15,000]

**GA002_bracket.** [IWER: If R is unwilling to answer or does not remember, ask unfolding bracket questions here. 访员注意：如果受访者不愿回答或者忘记了，在此处分级展开提问]: 10,000/30,000/50,000/100,000/200,000 yuan 元

**[Show Card 出示卡片25]**

**GA003.** Did you receive any of the following types of individual income in the past year? (check all that apply) 过去一年，您有没有领到下列的哪种收入？（可多选）

1. Pensions (including wages from government institutions and firms, supplemental pension of the firms,and income from such programs as rural pension insurance, Urban residents’ pension and commercial pension insurance, and pension subsidy for the oldest old) 退休金或养老金（包括政府和事业单位、企业基本养老金，企业补充养老金、农村、城乡、城镇居民养老金、商业养老金、高龄老人养老补助等）
2. unemployment compensation 失业补助
3. pension subsidy 无保障老人生活补贴
4. Workers’ compensation from Industrial Accident Compensation Insurance includes wage-replacement benefits, disability benefits, and survivors' benefits工伤保险金包括误工补贴、伤残补助、丧葬费等
5. elderly family planning subsidies 独生子女老年补助
6. medical aid 医疗救助
7. other government subsidies政府给您个人的其他补助
8. social assistance 社会捐助
9. other income sources (including alimony, child support) 其他收入（离婚后的赡养费、子女抚养费等）
10. None of the above 以上均没有 Skip GA004 跳过GA004

F1：“医疗救助”在部分区县试点，救助标准是由各区县根据自身经济情况制定，导致救助标准有高有低。主要三种，一是提供社会医疗救助金，给救助对象以经济补偿；二是给医疗机构一定的经济补贴，使后者直接减免救助对象的部分医疗费；三是由社会医疗救助机构举办专门医疗机构，免费为救助对象提供医疗服务。

**GA004.** How much did you receive last year? ____yuan or ___yuan/month

过去一年一共领了多少钱？______(GA004_1)元 或每个月领多少钱？_______(GA004_2 )元

If GA003=1 and GA004=DK, 如果领取退休金或养老金，但是回答不知道领了多少钱：

**GA004_bracket.** [IWER: If R is unwilling to answer or does not remember, ask unfolding bracket questions here. 访员注意：如果受访者不愿回答或者忘记了，在此处分级展开提问]: 10,000/30,000/50,000/100,000/200,000 yuan 元

**Part 1_2: Other Household Member’s Wage Income and Individual-based transfers 其他家户成员的工资收入和个人获得的转移收入**

[IWER reminder: make sure others are not present. 访员注意：请确保没有其他人在场。]

**[Intro: We’d like to ask you some questions about the income and assets of OTHER members of your household. 下面我们想问问您家其他家户成员的收入情况。我们将会对您的回答严格保密，并且仅用于学术研究。]**

**[IWER: The names of other household members are preloaded from the cover screen information. For each member（excluding main respondent and spouse）, answer the following questions:] [访员注意：其他家户成员名单要从过滤问卷获得。对于每一个家户成员（不包括主要受访者及其配偶），分别问下面的问题：]**

**GA005.** Did [preload household member name] receive any wage and bonus income in the past year? 过去一年， [preload household member name]有没有领工资（包括奖金，不包括退休工资）？

1. Yes 有

（2）No 没有 skip to GA007 _跳到GA007

**GA006.**  How much did he/she receive last year? ____yuan or ____yuan/month

过去一年一共领了多少钱？______(GA006_1)元 或每个月领多少钱？_______(GA006_2)元

Wage income: yuan [soft check >200,000] or yuan/month [soft check>15,000]

**GA006_bracket.** [IWER: If R is unwilling to answer or does not remember, ask unfolding bracket questions here. 访员注意：如果受访者不愿回答或者忘记了，在此处分级展开提问]: 10,000/30,000/50,000/100,000/200,000 yuan 元

**[Show Card 出示卡片25]**

**GA007.** Did [preload household member name] receive any of the following types of individual income in the past year? (check all that apply) 过去一年，[preload household member name] 有没有领到下列的哪种收入？（可多选）

1. Pensions (including wages from government institutions and firms, supplemental pension of the firms,and income from such programs as rural pension insurance, Urban residents’ pension and commercial pension insurance, and pension subsidy for the oldest old) 退休金或养老金（包括政府和事业单位、企业基本养老金，企业补充养老金、农村、城乡、城镇居民养老金、商业养老金、高龄老人养老补助等）
2. unemployment compensation 失业补助
3. pension subsidy 无保障老人生活补贴
4. Workers’ compensation from Industrial Accident Compensation Insurance includes wage-replacement benefits, disability benefits, and survivors' benefits工伤保险金包括误工补贴、伤残补助、丧葬费等
5. elderly family planning subsidies 独生子女老年补助
6. medical aid 医疗救助
7. other government subsidies政府给您个人的其他补助
8. social assistance 社会捐助
9. other income sources (including alimony, child support) 其他收入（离婚后的赡养费、子女抚养费等）
10. None of the above 以上均没有 Skip GA008 跳过GA008

F1：“医疗救助”在部分区县试点，救助标准是由各区县根据自身经济情况制定，导致救助标准有高有低。主要三种，一是提供社会医疗救助金，给救助对象以经济补偿；二是给医疗机构一定的经济补贴，使后者直接减免救助对象的部分医疗费；三是由社会医疗救助机构举办专门医疗机构，免费为救助对象提供医疗服务。

**GA008.** How much did he/she receive last year? ____yuan or ____yuan/month

过去一年一共领了多少钱？______(GA008_1)元 [soft check >10000] 或每个月领多少钱？_______ (GA008_2)元 [soft check >3000]

If GA007=1 and GA008=DK, 如果领取退休金或养老金，但是回答不知道领了多少钱：

**GA008_bracket.** [IWER: If R is unwilling to answer or does not remember, ask unfolding bracket questions here. 访员注意：如果受访者不愿回答或者忘记了，在此处分级展开提问]: 10,000/30,000/50,000/100,000/200,000 yuan 元

**[PROCEDURE: Skip to next person 程序： 请询问下一个人]**

### PART 2 HOUSEHOLD AGRICULTURAL INCOME AND EXPENDITURE 家户农业收入与支出

**[Intro: Next we will ask some questions about your household agricultural income and expenditure.**

**下面，我们将问一些有关您家农业收入与支出的问题。]**

**GB001.** Did your household engage in agricultural work (including cropping, forestry, livestock, and fish) last year? 过去一年，您家有没有从事农业方面的工作（包括种地、管理果树、采集农林产品、养鱼、打鱼、养牲畜以及去市场销售农产品等）？

(1) Yes 有

(2) No 没有 skip to GC001 请跳至GC001

**GB002.** [IWER: The names of other household members not including respondent and spouse are preloaded from the cover screen information. [访员注意：其他家户成员不包括主要受访者及其配偶，其他家户成员的名单从过滤问卷获得。] [preload other household member name] Did [name] engage in agricultural work (including cropping, forestry, livestock, and fish) last year? 过去一年，[name]有没有从事农业方面的工作（包括种地、管理果树、采集农林产品、养鱼、打鱼、养牲畜以及去市场销售农产品等）？

(1) Yes 有

(2) No 没有

#### Crops and forestry products 农林产品:

**GB003.** Did your household engage in cropping or forestry last year? 您家过去一年是否从事了种植业或林业（包括花木种植）？

1. Yes 是
2. No 否  Skip to GB007 请跳至GB007

**GB004.** When was the most recent harvest? 最近的一次收成是在什么时候？

_______2009..2011 (GB004_1)year 年________0..12 (GB004_2)month 月

[IWER: Mark the year using four digits. Take down the month as its actual number. For example, write January as “1” not “01”, December as “12”. If do not remember month, fill ‘0’. 访员注意: 用4位数表示年，按照实际的月份填写月。例：1月写作“1”，而不是“01”,12月写作“12”。如果记不住月份，请填入’0’。]

**GB005.** What is the value of all crops and forestry products (including sold lumber) produced in the past year? 过去一年，您家生产的农产品和林产品（包括所有卖出去的和自己消费的）加起来值多少钱？ ___ Yuan 元 [soft check: 75000 yuan]

**GB005_bracket.** [IWER: If R is unwilling to answer or does not remember, ask unfolding bracket questions here. 访员注意：如果受访者不愿回答或者忘记了，在此处分级展开提问]: 1,000/3,000/5,000/7,000/10,000 yuan 元

**GB006.** What was the total cost of producing crops (including vegetables and Chinese herbs) and forestry products in the past year? (including Seeds (including home produced), Fertilizer, Organic fertilizer, Pesticide, Plastic sheets, Hiring labor (including with machine or animals), Land rents, Rents (excluding land rents), Irrigation, Fuel,Transportation, Processing, Marketing (including packaging, management fee) )

过去一年，您家为了农业生产（包括种植粮食作物、蔬菜和中药材等）和林业生产（包括种植蘑菇、木耳等林下作物、种植茶叶），总共投入了多少钱？[包括种子(包括自家留种的价值）、化肥、农家肥、农药、塑料薄膜、雇工费（包括其使用的机器和役畜）、土地租金、除地租以外的其他租金（例如：租用收割机）、灌溉费、燃料、运输费、加工费、市场费用（包括包装费，管理费等）) ______ Yuan 元 [soft check: 50,000 yuan]

**GB006_bracket.** [IWER: If R is unwilling to answer or does not remember, ask unfolding bracket questions here. 访员注意：如果受访者不愿回答或者忘记了，在此处分级展开提问]: 3,00/6,00/1,000/2000/5,000 yuan 元

**Livestock and fisheries 牲畜和水产品：**

**GB007.** Did your household grow any livestock or aquatic life last year? 您家过去一年养过牲畜或者水产品吗？

1. Yes 是
2. No 否 Skip to GC001 请跳至GC001

**GB008.** What is the current value of all livestock（including chicken, duck, cattle, pig, sheep, etc.）and aquatic life? 现在，您家所有的家禽、家畜（包括鸡、鸭、牛、猪、羊等），水产品加起来一共值多少钱？____Yuan 元 [soft check: 100,000 yuan]

**GB008_bracket.** [IWER: If R is unwilling to answer or does not remember, ask unfolding bracket questions here. 访员注意：如果受访者不愿回答或者忘记了，在此处分级展开提问]: 5,00/1,500/3,000/4,500/9,000 yuan 元

**GB009.** What was the value of all livestock and aquatic life at this time last year? 去年这个时候，您家拥有的牲畜及水产品一共值多少钱？______Yuan 元[soft check: 100,000 yuan]

**GB009_bracket.** [IWER: If R is unwilling to answer or does not remember, ask unfolding bracket questions here. 访员注意：如果受访者不愿回答或者忘记了，在此处分级展开提问]: 5,00/1,500/2,500/4,000/8,000 yuan 元

**GB010.** How much did you spend purchasing new livestock and aquatic life in the past year?

过去一年，你们家买牲畜及水产品一共花了多少钱？______( Yuan 元 [soft check: 50,000]

**GB011.** What was the value of all livestock and aquatic life that were sold or consumed in the past year? 过去一年，你们家卖出去的和自家消费的牲畜及水产品加起来一共值多少钱? ______Yuan 元 [soft check: 100,000 yuan]

**GB011_bracket.** [IWER: If R is unwilling to answer or does not remember, ask unfolding bracket questions here. 访员注意：如果受访者不愿回答或者忘记了，在此处分级展开提问]: 2,00/9,00/1,500/2,500/5,000 yuan 元

**GB012.** What was the value of all livestock products produced（including the self consumption value） in the past year, including milk, wool (including cashmere, sheep or goat skin), and eggs) 过去一年，你们家养的牲畜生产出来的副产品（比如鸡蛋、牛奶、羊毛、羊绒、羊皮），加起来一共值多少钱（包括所有卖出去的和自家消费的）？______ Yuan 元 [soft check: 50,000 yuan]

**GB012_bracket.** [IWER: If R is unwilling to answer or does not remember, ask unfolding bracket questions here. 访员注意：如果受访者不愿回答或者忘记了，在此处分级展开提问]: 100/200/300/500/1000 yuan 元

**GB013.** What was the cost of producing livestock and aquatic life in the past year, including the value of all feed, medicine, pasture fees, animal pens, wages, etc.) 过去一年，养这些牲畜及水产品一共花了多少钱，包括喂养费、医药费、放牧费、畜舍栅栏费、雇工费等？ ______ Yuan 元 [soft check: 50,000 yuan]

[soft check: “reported raising livestock but no evidence of such activity” if GB008 =0 and GB009 ==0 如果受访者饲养过牲畜或水产品，但GB008 =0 且 GB009 =0，则进行检查]

### PART 3. Self-employed Activities 个体经营或开办私营企业

**GC001.** Did your household members engage in any self-employed activities last year? 过去一年，您家是否有家户成员从事某些个体经营或开办私营企业？

1. Yes 是
2. No 否   Skip to GD001 请跳至GD001

**GC002.** How many types of activities did your household members participate in the last year? _____ Activities. 过去一年，您家家户成员从事几项个体经营活动或开办几家私营企业？

**For each each self-employed activity in GC002 , ask GC003 - GC005 .**

**针对每一项个体经营活动或私营企业，询问GC003 - GC005**

**GC003.** Who engage in this self-employment businesse in the past year? 过去一年，从事这项个体经营或私营企业的家户成员有哪些？

[IWER: All the names of household members are preloaded from the cover screen information 访员注意：所有家户成员名单（包括主要受访者及其配偶）要从过滤问卷获得] (可多选)

**[Show Card 出示卡片26]**

**GC004 .** Which types of activities? 这项活动属于什么行业？（可多选）

- 1. Services (cooking, sewing, private clinic etc.) 服务业（烹饪、缝纫、私人诊所等）(GC004_1 )
  2. Transportation 交通运输(GC004_2)
  3. Construction 建筑业(GC004_3 )
  4. Mining 采掘(GC004_4 )
  5. Processing production 加工生产业(GC004_5 )
  6. Business 商业(GC004_6)
  7. Others 其他(GC004_7)

**GC005.** Not including fixed capital costs, what is your best estimate of the net income earned from this activity by your household members last year?[ If the activity was conducted jointly with non-household members, report only the net income earned by household members. Remember to consider the following types of costs: energy, housing or equipment rental, raw materials, transportation, marketing, wages, taxes or fees.] 不包括固定资本成本，您能否精确估计一下您家过去一年从这项活动净赚多少钱？如果此项经营活动有非家户成员参与，只计算家户成员的净收入。别忘了考虑下列各种成本：能源、住房和设备租用费、原材料、交通费、营销、工资、税收和杂费。_______ Yuan元 [soft check: 500,000 yuan]

**GC005_bracket.** [IWER: If R is unwilling to answer or does not remember, ask unfolding bracket questions here. 访员注意：如果受访者不愿回答或者忘记了，在此处分级展开提问]: 5,000 /10,000/50,000/100,000/200,000 yuan 元

### PART 4 HOUSEHOLD PUBLIC TRANSFER INCOME 家户政府转移支付收入

**We ask the public transfers received by the households (with household as the unit). Public transfers have characteristic of welfare, such as Wubaohu Subsidy and Tekunhu Subsidy gived by government.**

**这部分询问家户从政府所得的各种转移支付收入。政府转移支付具有福利支出的性质，如政府给五保户和特困户的补助金等。这里询问的是以家户为单位获得的转移支付。**

**GD001.** How much Dibao assistance did your household receive last year? (if not applicable, fill in 0 yuan).

您家过去一年得到低保了吗？得到多少？（如果没有，请填0）

_______________Yuan元

F1：“低保”居民最低生活保障的简称。是在城市已经建立了国有企业下岗职工基本生活保障、失业保险和城市居民最低生活保障等“三条保障线”制度的基础上，建立实行最低生活保障的制度。

**[Show Card 出示卡片27]**

**GD002.** Did your household receive any of the following government subsidies in the past year? (check all that apply) 您家过去一年有没有收到下列政府补助？(可多选) [soft checks for each category: 20,000 yuan对每项进行检查：20,000元。]

1. Reforestation 退耕还林: how much? 有多少？______(GD002_1 )Yuan元
2. Agricultural subsidies 农业补助: how much? 有多少？______(GD002_2 )Yuan 元
3. Wubaohu ( targets low-income, blind, disabled, aged persons, and young persons that have no means to support themselves.五保户补助金（用来补助那些无法自力更生的低收入者、盲人、身体残疾者、老人和小孩: how much?有多少？______(GD002_3)Yuan 元
4. Tekunhu 特困户补助: how much? 有多少？______(GD002_4 )Yuan 元
5. Work injury subsidies to the immediate family members 工伤人员供养直系亲属抚恤金how much? 有多少？______(GD002_5 )Yuan 元
6. Emergency or disaster relief (jiujikuan, jiuzaikuan) last year? 突发事件或重大灾难之后的补助（即救济金、赈灾款）（包括实物形式）: how much? 加上实物救助的价值一共有多少？______(GD002_6 )Yuan 元
7. Other 其他补助: how much? 有多少？______(GD002_7)Yuan 元

(8) 2 Did your household receive 00000000000000000000000000000000000000000000000000000000000000000000000000000000000000000000000(8) None 没有收到任何政府补助 **Skip to GD003 请跳至GD003**

**[Show Card 出示卡片28]**

**GD003.** Did your household receive any income from the following sources in the past year? (check all that apply) 您家过去一年有没有收到下列哪种捐助或补偿？（可多选）

1. Donations from the society (including cash, and items like food, clothing, etc.)社会捐助（包括现金，食品、衣服等）: how much? 加上实物的价值一共有多少？_____(GD003_1 )Yuan 元 [soft check: 20,000 yuan]
2. Compensation for land seizure last? 征地补偿金: how much? 有多少？_______(GD003_2 )Yuan 元 [soft check: 100,000 yuan]
3. Compensation to pulling down your house or apartment last year? 住房拆迁补偿(FD003S3)_: how much? 有多少？______(GD003_3 )Yuan 元 [soft check: 100,000 yuan]
4. None 没有收到任何捐助或补偿

### PART 5 HOUSEHOLD LIVING EXPENDITURE 家户生活支出

**GE001.** We wish to know your family food expenditure for the last week. Are you the primary person who purchases food for the household? 我们想知道您家最近一周的食品支出。您负责为家里购买食品吗？

1. Yes 是 Skip to GE004 请跳至GE004
2. No 否

**GE002.** Who is the primary person purchasing food for the household? 谁负责为您家购买食品？[CAPI: Preload all the HHmember list 列出所有家户成员的名单]

[IWER: If possible, the primary person who purchases food for the household should answer the questions about expenditures FE003-FE009][访员注意：如果可能的话，由负责为该家庭购买食品的人来回答生活支出问题]

**GE003.** [IWER：Please record who answered these questions. 访员注意：请记录回答收入支出的人]

(1)Primary person who purchases food for the household （not Financial respondent）负责为该家庭购买食品的人（非财务受访者）

(2)Financial respondent 财务受访者

(3)Other其他

**GE004.** In the past week, how many people usually ate meals together in your household (not including guests)? 最近一周，您家里一般有几口人吃饭（不包括客人）？______Persons 人 [soft check: 10]

**GE005.**. Last week how many meals did you provide to guests? 最近一周，您家的客人在您家吃了几顿饭（按人次计算）？________ meals 人次 [soft check: 100]

**Food** **食品:**

**[Intro: The next questions are about your household living expenditure, including your household members ‘(preloaded names of household members) living expenditure. If one attends school/work outside and comes home almost every week, GE006 –GE008 includes his/her expenditureon food and meals outside. If one attends school/work outside but not come home every week, GE006 –GE008 excludes his/her expenditureon food and meals outside.]**

**[引语：下面问题是关于您家的生活支出的。这些支出包括所有家户成员（加载家户成员的名单）的生活支出。如果有人在外上学/上班，但周末回家，他们在外上学/上班发生的开支算在内；如果有人在外打工等，周末不回家，他们在外的生活开支不算在内。]**

**GE006.**In the past week, what was the value of household consumption of food, including both food purchased and food eaten from your own production (excluding eating out expenditure, alcohol, Cigarettes, cigars and tobacco expenditure) ? 最近一周，您家花了多少钱购买食品，包括自家生产和消费的农产品，不包括外出就餐、购买香烟、酒水等？_______ Yuan元 [soft check: 6000 yuan]

**GE007.** Among it, how much did your household spend on eating out? 最近一周，您家花了多少钱外出就餐等？______Yuan元 [soft check: 3000 yuan]

**GE008** Among it, how much did your household spend on alcohol, Cigarettes, cigars and tobacco? 最近一周，您家花了多少钱购买香烟、酒水等？_____ _Yuan元 [soft check: 3000 yuan]

**[Show Card 出示卡片29]**

**GE009.** Please tell me the expenditure last month for your household for the following items. 下面我们想了解您家过去一个月在以下各项消费中的支出。[soft check for each category: 5000 yuan]

[IWER: fill in 0 if no corresponding expenditure; fill in -9999 if the respondent cannot recall the expenditure.访员注意: 没有相应项支出用0元表示，记不清该支出用-9999表示。]

|  | Item  项目 | Expenditure (Yuan)  支出（元） |
| --- | --- | --- |
| 1 | Communication fees (including post, internet usage, telephone and cell phone usage) 邮电、通讯支出（包括电话、手机、上网、邮寄等） | (GE009_1 ) |
| 2 | Utilities: Water and electricity 水费、电费 | (GE009_2 ) |
| 3 | Fuels (including gas, coal, etc.) 燃料费（包括煤炭、煤制品、柴草、木炭、液化气等） | (GE009_3 ) |
| 4 | Fees for Matron, housekeepers and servants 保姆、小时工、佣人等的支出 | (GE009_4) |
| 5 | Local Transportation 在当地的交通费 | (GE009_5) |
| 6 | Household items and personal toiletries that are used daily plus beauty treatments (e.g., detergent, soap, toothpaste, toothbrush, cosmetics, beauty salon, etc.) 日用品包括美容化妆品（如洗衣粉、香皂、肥皂、牙膏、牙刷、美容化妆品等） | (GE009_6 ) |
| 7 | Entertainment (including fees to buy books, newspapers, VCCs, DVDs, going to cinema and bars) 文化娱乐支出（包括书报杂志、光盘、影剧票、歌舞厅和网吧等） | (GE009_7) |

**[Show Card 出示卡片30]**

**GE010.** In the last year how much did your household spend on the following items? 下面我们想了解您家过去一年在以下各项消费中的支出。

[IWER: fill in 0 if no corresponding expenditure; fill in -9999 if the respondent cannot recall the expenditure. 访员注意: 没有相应项支出用0元表示，记不清该支出用-9999表示。] [soft check: >=100,000 yuan]

|  | Item  项目 | Expenditure (Yuan)  支出（元） |
| --- | --- | --- |
| 1 | Clothing and bedding 衣着消费 | (GE010_1 ) |
| 2 | Long distance traveling expenses (including travel fees through train, car, bus, plane and ship) 家庭的旅游支出（包括旅行时坐火车、汽车、飞机、轮船的费用） | (GE010_2) |
| 3 | Heating(centrally heated) 家庭的取暖费支出(指集中供暖) | (GE010_3) |
| 4 | Furniture and consumption of durable goods, includes refrigerator, washing machine, TV and expensive instruments like pianio. 家具和耐用消费品的支出，包括电冰箱、洗衣机、电视和钢琴等高档乐器 | GE010_4) |
| 5 | Education and training(including tuition, training fees, etc.) 教育和培训支出（包括学杂费、培训费等） | (GE010_9) |
| 6 | Medical expenditure 医疗支出（包括直接或间接） | (GE010_10) |
| 7 | Fitness expenditures 保健费用（包括健身锻炼及产品器械、保健品等） | (GE010_5) |
| 8 | Beauty (including make-ups, facials, massages, etc.)美容支出（包括化妆品、美容护理、按摩等） | (GE010_6) |
| 9 | Purchase, Maintenance and repair (of transportation vehicles, appliances, communication products, etc.) 各种交通通讯工具的购买（如自行车、电动自行车和手机）、维修及配件费用 | (GE010_7) |
| 10 | Taxes and fees turned over to the government 上交给政府相关部门的税费和杂费（不包括所得税） | （GE010_8） |
| 11 | Automobiles 购买汽车 | (GE010_11) |
| 12 | Electronics (laptops, computers and accessories, vedio games, etc.)电器（包括笔记本电脑、台式电脑和配件等） | (GE010_12) |
| 13 | Property management fees (including parking fee) 物业费（包括车位费） | (GE010_13) |
| 14 | Donations to the society (including cash, and items like food, clothing, etc.)社会捐助支出（包括现金，食品、衣服等） | (GE010_14) |

**GE011.** How often did the respondent receive assistance in answering section Household income and expenditure?受访者填写该部分问卷时是否求助？

[IWER: If it is answered by a proxy, please record the respondent’s reaction. 访员注意：如果是协助回答，请记录受访者的反应。]

1. Never 从未
2. A few times 偶尔几次
3. Most or all of the time 大多数时间

## HA HOUSEHOLD ASSETS 家户资产

[IWER: This section is asked of the financial respondent, from question CV025. Do not allow a proxy respondent to answer the entire section. 访员注意：这部分要问财务受访者，不允许完全请别人代答。]

**PART 1 Current Residence 第一部分：现有住宅**

**The following questions pertain to your current residence. 下面是关于您现住宅的问题。**

**HA001.** When did your household start to live at your current residence? 您家何时开始住进现在的房子？ _________ 1900..2011Year 年

[IWER: Mark the year using four digits. 访员注意: 用4位数表示年。]

**HA002.** Do you pay rent for your current residence? 您家现在住的房子要付租金吗？

- 1. Yes 要
  2. No 不要 Skip to HA007 请跳至HA007

**HA003.** How much rent do you pay each month? 如果要付租金，一个月付多少钱？______ Yuan/month元/月 [soft check: <100, >10,000 yuan]

**HA004.** Did you pay less than the market rental value? 您家支付的租金是否低于市场价？

1. Yes 是
2. No 否 Skip to HA007 请跳至HA007

**HA005.** If you rented the same housing unit from the market, what is the rent per month you would have to pay? 如果从市场上租同样的房子，每月的租金会是多少？______________ Yuan/Month 元/月[soft check: <100, >10,000 yuan]

**HA006.** How much of the rent was paid by a housing subsidy from the employer of a household member? ____yuan [soft check >10,000 yuan], which household member?_____[preloaded list] 您家户成员的单位提供多少住房补贴来支付租金？_______(HA006_1)元，哪一个家户成员？_______(HA006_2) [从前面提取家户成员名单]

**HA007.** Who owns your current residence? 您家现在的房子归谁所有？

(1) 完全归家户成员所有

(2) 部分归家户成员所有

(3) 完全不归家户成员所有 Skip to HA010 请跳至HA010

**HA008.** Which household member(s) own the house? (preloaded names of household members)哪一个/些家户成员拥有这处房子？_______ [从前面提取家户成员名单]

**HA009.** What share of the house is owned by (preloaded names of household members)? （加载HA008 是答案）拥有产权的比重是？ ________ 0.00..100.00% [hard check: range 0-100]

**Skip to HA011 请跳至HA011**

**HA010.** What non-household members own all or part of your current residence? (circle all that apply) 您家现在的房子是哪些非家户成员的？（可多选）

1. working unit of household member, 家户成员单位的，which household member?_____ (HA010_1)哪一个家户成员的单位 [preloaded list][从前面提取家户成员名单]
2. government indemnificatory housing 政府保障性住房
3. child(non- household member) of main respondent or spouse 主要受访者（或其配偶）的非家户成员的孩子, which child?哪个孩子？___(HA010_2) [preloaded list]从前面提取孩子的名单
4. parent(non- household member) of main respondent or spouse 主要受访者（或其配偶）的非家户成员的父母___________(HA010_3 ) [preload list] 从前面提取父母的名单
5. nonresident other relatives 没在一起居住的其他亲戚
6. friends 朋友
7. other 其他人

F1：“政府保障性住房”指政府在对中低收入家庭实行分类保障过程中所提供的限定供应对象、建设标准、销售价格或租金标准，具有社会保障性质的住房，包括两限商品住房、经济适用住房、政策性租赁住房以及廉租房。

**HA011.** What is the present market value of your house? Or, what is the present market value of a similar housing unit within its neighborhood? 这所房子当前的市场价是多少？或者说，这房子附近类似的房子当前的市场价是多少？

Total price 总价格_______________(HA011_1) 10000Yuan 万元 [soft check <0.1, >500] or unit price或单位价格_____________(HA011_2)1000Yuan/m^2^ 千元/平方米 [soft check: <0.1, >25]

※IWER: skip to HA013 if R answered HA011. If not, ask unfolding brackets. 如果答卷人回答了HA011，跳至HA013，否则，提问分级展开问题。

[PROCEDURE: If HA011_1>=100,ask HA011_check][程序：如果HA011_1大于等于100，询问HA011_check]

**HA011_check.** Are you sure the present market value of your house is [preload HA011_1] 10000Yuan? 这所房子的市场价确定是[加载HA011_1的答案]万元吗？

- 1. Yes 是
  2. No 否 go back HA011 返回到HA011

**HA012.** [IWER: If R is unwilling to answer or does not remember, ask unfolding bracket questions here. 访员注意：如果受访者不愿回答或者忘记了，在此处分级 展开提问] 20,000/50,000/100,000/200,000/500,000 yuan元

**[If HA007 =2 or 3, then skip to HA027 如果HA007 =2 or 3，那么请跳至HA027]**

**HA013.** Do you or other household members take out a bank loan to finance the purchase, construction, or decoration of your house now? 您家是否正在用按揭贷款来购买、建造或装修您的房子？（已还清贷款不算）

- 1. Yes 是
  2. No 否 → Skip to HA016 请跳至 HA016

F1：“按揭贷款”指购房者以所购住房做抵押并由其所购买住房的房地产企业提供阶段性担保的个人住房贷款业务。房子指现在的住宅。

**HA014.** What is the outstanding amount of the loans? 还有多少贷款没还清？ _____________ Yuan 元 [soft check: >500,000 yuan]

**HA015.** What is the monthly mortgage payment? 每月要还多少贷款？___Yuan 元 [soft check: >10000]

**HA016.** How was this housing unit obtained? 房子的来源是什么？

1. Purchased from market 是从市场买来的
2. Purchased from working unit of household member(s) 从家户成员的单位买来的，哪一个家户成员的单位______？(HA016_1 ) [preloaded list]从前面提取家户成员名单
3. Purchased from child(non-household member) of main respondent or spouse, which child? 从主要受访者（或其配偶）的非家户成员的孩子那里购得，哪个孩子？___(HA016_2 ) [preloaded list]从前面提取名单
4. Purchased from Parents(non- household member), of who（main respondent or spouse）? 从主要受访者（或其配偶）的非家户成员的父母那里购得，谁的（主要受访者还是其配偶的）？______(HA016_3)
5. Purchased from Other relatives 从其他亲戚那里购得
6. Self-built 自建 → Skip toHA025 请跳至 HA025
7. Inherited, bequeathed, or given 继承（如祖业）或受赠与 → Skip to HA020 请跳至HA020
8. Received home as compensation for demolition of old home 拆迁换房 Skip to HA021
9. Other 其他 → Skip to HA020 请跳至 HA020

**HA017.** When did you purchase it? 这房子什么时候买的？__________ 1900..2011 Year 年Skip to HA018 请跳至HA018

[IWER: Mark the year using four digits. 访员注意: 用4位数表示年。]

**HA018.** How much of your own money did you spend on the new house? 您家自己付了多少钱？

______________10000Yuan 万元 [soft check >500]

If HA016 = 8, skip to HA022 如果HA016 = 8，跳至 HA022

**HA019.** Was it purchased at market price, subsidized by working unit, or as purchased as economical housing? 这房子是以市场价购买、单位优惠价格买的、或者是经济适用房？

- 1. Market price 以市场价购买 → Skip to HA025 请跳至 HA025
  2. Subsidized by working unit 单位优惠价格买的
  3. Economic housing 经济适用房
  4. Other 其他

F1：“经济适用房”指已经列入国家计划，由城市政府组织房地产开发企业或者集资建房单位建造，以微利价向城镇中低收入家庭出售的住房，具有社会保障性质的商品住宅。具有经济性和适用性的特点。

**HA020.** What would you have to pay if you had paid a market-set price for the same housing? 同样房子当时的市场价格是多少？

Total price 总价格_______________(HA020_1)10000Yuan 万元 [soft check <0.1, >500] or unit price或单位价格_____________(HA020_2)1000Yuan/m^2^ 千元/平方米 [soft check: <0.1, >25]

Skip to HA025 if HA016 not equal to (8) 如果HA016 不等于（8），请跳至HA025

[PROCEDURE: If HA020_1>=100,ask HA020_check][程序：如果HA020_1大于等于100，询问HA020_check]

**HA020_check.** Are you sure you had paid a market-set price [preload HA020_1] 10000Yuan for the same housing? 同样房子在当时的市场价确定是[加载HA020_1的答案]万元吗？

(1)Yes 是

(2)No 否 go back HA020 返回到HA020

**HA021.** When did you receive the housing? 什么时候换的这套房子？________ 1900..2011year 年 [IWER: Mark the year using four digits. 访员注意: 用4位数表示年]

**HA022.** How much was the market value of the old house at that time? 您家的老房子当时的市场价格是多少？

Total price 总价格_______________ 10000Yuan 万元 [soft check: <0.1, >500]

[PROCEDURE: If HA022_1>=100,ask HA022_check][程序：如果HA022_1大于等于100，询问HA022_check]

**HA022_check.** Are you sure you had paid a market-set price [preload HA022 _1] 10000Yuan for the old house? 您确定您家的老房子当时的市场价格是[加载HA022_1的答案]万元吗？

(1)Yes 是

(2)No 否 go back HA022 返回到HA022

**HA023.** How much was the market value of the new house at that time? 换的新房子当时的市场价格是多少？

Total price 总价格_______________(HA023_1) 10000Yuan 万元 [soft check: <0.1, >500] or unit price或单位价格_____________(HA023_2)1000Yuan/m^2^ 千元/平方米 [soft check: <0.1, >25]

[PROCEDURE: If HA023_1>=100,ask HA023_check][程序：如果HA023_1大于等于100，询问HA023_check]

**HA023_check.** Are you sure the market value of the new house is [preload HA023 _1] 10000Yuan at that time? 您确定换的新房子当时的市场价格是[加载HA023_1的答案]万元吗？

(1)Yes 是

(2)No 否 go back HA023 返回到HA023

**HA024.** Can you sell the house freely? 你家是不是可以自由卖房？

- - 1. Yes 是的
    2. No, restricted by work unit 不是，受到单位的限制

**HA025.**  How much did you spend on decorating or renovating your house (exclude expenditures on furniture)? 您装修房屋花了多少钱（不包括家具开销）？__________________ Yuan 元 [soft check: >500,000 yuan]

**HA026.** When was it decorated/renovated (if more than once, choose time of greatest expendithure)? 是在哪年装修的（如果有多次装修，问花费最多的一次）？

__________ 1900..2011year 年

[IWER: Mark the year using four digits. 访员注意: 用4位数表示年。]

**HA027.** Excluding the house in which you live, do you or members of your household own any other residential properties? 除了您家现在的住房以外，您或其他家户成员是否拥有其他房产（是否拥有以您或其他家户成员是否是房主为准）？

1. Yes 是
2. No 否 → Skip to HA054 请跳至 HA054

**HA028.** How many other housing units do you or members of your household currently own? 您或其他家户成员还有几处其他的房产？ ____________ 0..10处

**PART 2 Other Residences 第二部分：其他房产**

**For every other housing unit owned by house members, ask the following questions,**

**对所有家户成员拥有的其他的每处房产，询问以下的问题：**

**HA029. Now we want to know other housing unit owned by house members, 现在我们想了解有关你第n【n从GA036 load】处房产的情况**

Where is this house located? _______Province__________District, Zip Code_____________

该房的地址是 _______(HA029_1)省__________(HA029_2)市或区， 邮编是____________ (HA029_3)

**HA030.** Who owns this residence? 这所房子归谁所有？

(1) Owned completely by your household member(s). 完全归家户成员所有

(2) Owned partly by your household member(s). 部分归家户成员所有

**HA031.** Which household member(s) own the house? (preloaded names of household members)哪一个/些家户成员拥有这处房子？_______ [从前面提取家户成员名单]

**HA032.** What share of the house is owned by (preloaded names of household members)? (加载GA042_2的答案)拥有产权的比重是？ _______0.00..100.00% [hard check: range 0-100]

**[If HA030 =1, skip to the procedure before HA034. 如果HA030 =1，跳至HA034前面的程序控制]**

**HA033.** What non-household members own all or part of your current residence? (circle all that apply) 您家现在的房子是哪些非家户成员的？（可多选）

1. working unit of household member, 家户成员单位的，which household member?_____ (HA033_1 )哪一个家户成员的单位______[preloaded list][从前面提取家户成员名单]
2. government indemnificatory housing 政府保障性住房
3. child(non- household member) of main respondent or spouse 主要受访者（或其配偶）的非家户成员的孩子, which child?哪个孩子？___(HA033_2) [preloaded list]从前面提取孩子的名单
4. parent(non- household member) of main respondent or spouse 主要受访者（或其配偶）的非家户成员的父母___________(HA033_3 ) [preload list] 从前面提取父母的名单
5. nonresident other relatives 没在一起居住的其他亲戚
6. friends 朋友
7. other 其他人

**[PROCEDURE: If HA031 does not include respondent or spouse, SKIP TO Next house. 如果HA031 答案不包括受访者及其配偶，请跳至下一处房产。 ]**

**HA034.** What is the present market value of your house? Or, what is the present market value of a similar housing unit within its neighborhood? 当前的市场价是多少？或者说，这房子附近类似的房子当前的市场价是多少？

Total price 总价格_______________(HA034_1)10000Yuan 万元[soft check <0.1, >500] or unit price或单位价格_____________(HA034_2)1000Yuan/m^2^ 千元/平方米 [<0.1, >30]

[CAPI Skip to HA036 if R answered HA034. If not, ask unfolding brackets. 如果答卷人回答了HA034，跳至HA036，否则，提问分级展开问题]

[PROCEDURE: If HA034_1>=100,ask HA034_check][程序：如果HA034_1大于等于100，询问HA034_check]

**HA034_check.** Are you sure your present market value of your house is [preload HA034_1] 10000Yuan? 您的房子的市场价确定是[加载HA034_1的答案]万元吗？

（1）Yes 是

（2）No 否 go back HA034 返回到HA034

**HA035.** [IWER: If R is unwilling to answer or does not remember, ask unfolding bracket questions here. 访员注意：如果受访者不愿回答或者忘记了，在此处分级展开提问] 20,000/50,000/100,000/200,000/500,000 yuan元

**HA036.** Does your household members take out a bank loan to finance the purchase, construct, or decorate your house? 您家是否正在用按揭贷款来购买、建造或装修房屋？（如曾按揭贷款买房，但已还清不算）

1. Yes 是
2. No 否 → Skip to HA039 请跳至 HA039

F1：“按揭贷款”指购房者以所购住房做抵押并由其所购买住房的房地产企业提供阶段性担保的个人住房贷款业务。房子指现在的住宅。

**HA037.** What is the outstanding amount of the loans? 还有多少贷款没还清？ _______________ 10,000 Yuan 万元 [soft check >500]

**HA038.** What is the monthly mortgage payment? 每月要还多少贷款？_____ Yuan 元 soft check >20,000]

**HA039.** How was this housing unit obtained? 房子的来源是什么？

1. Purchased from market 是从市场买来的
2. Purchased from working unit of respondent or spouse 从受访者或其配偶的单位买来的，谁的单位______(HA039_1 )？ [preloaded list]从前面提取受访者或其配偶的名单
3. Purchased from child of main respondent or spouse, which one? 从主要受访者（或其配偶）的孩子那里买的，哪一个_____(HA039_2 )preload[提取孩子名单]
4. Purchased from parents of main respondent or spouse, of who（main respondent or spouse）? 从主要受访者（或其配偶）的父母那里买的，谁的（主要受访者还是其配偶的）？______ (HA039_3 )
5. Purchased from Other relatives 从其他亲戚那里买的
6. Self-built 自建 → Skip to HA041 请跳至 HA041
7. Inherited, bequeathed, or given 继承（如祖业）或受赠与 → Skip to HA045 请跳至 HA045
8. Received home as compensation for demolition of old home, Skip to HA046 拆迁换房 Skip to HA046
9. Other 其他 → Skip to HA045 请跳至HA045

**HA040.** Was it purchased at market price, subsidized by working unit, or as purchased as economical housing? 这房子是以市场价买、单位优惠价格买的、或者是经济适用房？

1. Market price 以市场价买 → Skip to HA045 请跳至 HA045
2. Subsidized by working unit 单位优惠价格买的
3. Economic housing 经济适用房
4. Other 其他

F1：“经济适用房”指已经列入国家计划，由城市政府组织房地产开发企业或者集资建房单位建造，以微利价向城镇中低收入家庭出售的住房，具有社会保障性质的商品住宅。具有经济性和适用性的特点。

**HA041.** When did you purchase/build it? 这所房子是什么时候买/建的？ _________ 1900..2011Year 年

[IWER: Mark the year using four digits. 访员注意: 用4位数表示年。]

Skip to HA043 请跳至HA043

**HA042.** Can you sell the house freely if you want? 如果想卖的话，您家是不是可以自由卖房？

(1) Yes 是的

(2) No, restricted by work unit 不是，受到单位的限制

**HA043.** How much of your own money did you spend on the house (including loan-financed)? 买/建/换这所房子你家花了多少钱？_____________ 10000Yuan 万元 [soft check<0.1, >500]

[IWER: Skip to HA045 if R answered HA043 . If not, ask unfolding brackets. 如果答卷人回答了HA043 ，跳至HA045 ，否则，提问分级展开问题。]

[PROCEDURE: If HA043_1>=100,ask HA043_check][程序：如果HA043_1大于等于100，询问HA043_check]

**HA043_check.** Are you sure you had spend [preload HA043_1] 10000Yuan on the house? 您确定您买/建/换这所房子时花了[加载HA043_1的答案]万元吗？

(1)Yes 是

(2)No 否 go back HA043 返回到HA043

**HA044.** [IWER: If R is unwilling to answer or does not remember, ask unfolding bracket questions here. 访员注意：如果受访者不愿回答或者忘记了，在此处分级展开提问] 10,000/20,000/50,000/100,000/200,000 yuan元

If HA039 = 8, Skip to HA046. 如果HA039 = 8 跳至HA046

CAPI：If HA039 = 6, Skip to HA049

**HA045.** What would you have to pay if you had paid a market-set price for the same housing? 同样房子当时的市场价格是多少？

Total price 总价格_______________(HA045_1) 10000Yuan 万元[soft check >500] or unit price或单位价格_____________(HA045_2) 1000Yuan/m^2^ 千元/平方米 [soft check >30]

Skip to HA049 请跳至HA049 if HA039 does not equal to (8)

[PROCEDURE: If HA045_1>=100,ask HA045_check][程序：如果HA045_1大于等于100，询问HA045_check]

**HA045_check.** Are you sure you had paid a market-set price [preload HA045_1] 10000Yuan for the same housing? 同样房子在当时的市场价确定是[加载HA045_1的答案]万元吗？

(1)Yes 是

(2)No 否 go back HA045 返回到HA045

**HA046.** When did you receive the new housing? 什么时候换的这所房子？_____ 1900..2011Year 年 [IWER: Mark the year using four digits. 访员注意: 用4位数表示年。]

**HA047.** How much was the market value of the old house at that time? 您家的老房子当时的市场价格是多少？

Total price 总价格_____________ 10000Yuan 万元 [soft check <0.1, >500]

[PROCEDURE: If HA047_1>=100,ask HA047_check][程序：如果HA047_1大于等于100，询问HA047_check]

**HA047_check.** Are you sure the market-set price of your old house is [preload HA047_1] 10000Yuan? 您确定您家的老房子当时的市场价格是[加载HA047_1的答案]万元吗？

(1)Yes 是

(2)No 否 go back HA047 返回到HA047

**HA048.** How much was the market value of the new house at that time? 换的新房子当时的市场价格是多少？

Total price 总价格_______________(HA048_1) 10000Yuan 万元 [soft check<0.1, >500] or unit price或单位价格_____________(HA048_2) 1000Yuan/m^2^ 千元/平方米

[PROCEDURE: If HA048_1>=100,ask HA048_check][程序：如果HA048_1大于等于100，询问HA048_check]

**HA048_check.** Are you sure the market value of the new house at that time is [preload HA048_1] 10000Yuan? 您确定换的新房子当时的市场价格是[加载HA048_1的答案]万元吗？

(1)Yes 是

(2)No 否 go back HA048 返回到HA048

**HA049.** How much did you spend on decorating or renovating your house (exclude expenditures on furniture)? 您装修房屋花了多少钱（不包括家具开销）？________________ Yuan 元 [soft check <1000, >500,000]

**HA050.** When was it decorated/renovated (if more than once, choose time of greatest expendithure)? 是在哪年装修的（如果有多次装修，问花费最多的一次）？

_________ 1900..2011Year 年

[IWER: Mark the year using four digits. 访员注意: 用4位数表示年。]

**HA051.** What is the construction area of the house? 这房子的建筑面积是多大？___________ m^2^ 平方米 [soft check<10 or >500]

**[Skip to next house 跳至下一处房产]**

**HA052.** What is the monthly rental income for all room or houses owned by main respondent or spouse, that you are currently leasing? 主要受访者（或其配偶）拥有的房屋，正在出租的，每月一共能收取多少房租？

1. _______________ (HA052_1)Yuan/month 元/月 [soft check >20,000]
2. Not applicable 没有出租

**HA053.** What is the monthly rental income for all room or houses owned by other household members that are currently being leased? 其他家户成员拥有的房屋，正在出租的，每月一共能收取多少房租？

1. _______________ (HA053_1)Yuan/month 元/月
2. Not applicable 没有出租

**PART 3 Land 第三部分：土地**

**The following questions pertain to your land. 下面是关于土地的问题.**

**HA054.** Does your household have any collective distributing or rent cultivated land, forest land, pasture and/or pond? (Choose all that apply) 您家是否有集体分配的耕地、林地、牧场或水塘，或者从别人那里租用了耕地、林地、牧场或水塘？（可多选）

1. Cultivated land 耕地(HA054_1)
2. Forest land 林地(HA054_2)
3. Pasture 牧场(HA054_3)
4. Pond 水塘(HA054_4)
5. None 没有(HA054_5) Skip to HA064 请跳至HA064

[PROCEDURE: According to all options choosed in HA054 , ask HA055 -HA063 in loop] [程序：针对HA054 的所有选择，循环询问HA055 - HA063 题]

**HA055.** How many mu of [preload answer from HA054] do you have? 您家从集体分配到的[加载HA054的答案] 有多少亩？________ Mu亩 [soft check>50]

[PROCEDURE: If HA054 =1,ask HA056 ] [程序：如果HA054 =1，询问HA056 ]

**HA056.** How many mu of them are irrigable? 其中可灌溉面积是多少亩？_______Mu 亩 [hard check cannot be >HA055 ]

**HA057.** What is the rent per mu per year you would get if you rent out all your [preload answer from HA054]? 如果出租的话，每亩[加载HA054的答案]每年的租金会是多少？[soft check <10, >4000]

________ Yuan per mu per year 元每年每亩

**HA058.** Did you rent out any of your [preload answer from HA054] in the past year? 过去一年您家是否将[加载HA054的答案]出租给了其他人？

1. Yes 是
2. No 否 >>Skip to HA061 请跳至HA061

**HA059.** How much [preload answer from HA054 did you rent out the past year? 过去一年您家出租了多少亩[加载HA054的答案]？_____ Mu亩 [soft check >50] [hard check, cannot be >HA055]

**HA060.** How much rental income did you earn in the past year? 过去一年您家出租[加载HA054 的答案]，赚了多少钱？____ Yuan元

**HA061.** Did you rent in any [preload answer from HA054] from others (including the collective) in the past year? 过去一年您家是否从别人（包括集体）那租用了[加载HA054的答案]？

1. Yes 是
2. No 否 >>Skip to HA064 请跳至HA064

**HA062.** How much did you rent in the past year? 过去一年您家租用了多少亩[加载HA054 的答案]？_____ Mu亩 [soft check >100]

**HA063.** How much rent did you pay in the past year? 过去一年您家租用[加载HA054的答案]地花了多少钱？____ Yuan元 [soft check >20000]

**HA064.** How much rental income did you earn for any other household assets other than housing or land? (trees, use of fixed capital, durables, or livestock)? 除了出租房屋或者土地，过去一年您家出租其他家庭资产（如树木、固定资本的使用、耐用品或者牲畜）赚了多少钱？

1. _______(HA064_1)Yuan元
2. Not applicable 没有出租

F1：“耐用品”是指使用时间较长，至少在1年以上的物品，如电冰箱、汽车、电视机、机械设备等。

**PART 4 Equipments, Consumption durables, and Valuables.下面是关于家用设备、耐用消费品和其他贵重物品的问题。**

**[Show Card 出示卡片31]**

**HA065.** Do members of your household own the following assets? (Choose all that apply) 您家有下列物品吗？（可多选）

| For all categories, add [soft check <100 or >30,000] unless other check is written | [For each asset owned by the household] what is the asset’s current value? (Yuan)针对这家有的每一项物品提问：当前值多少？（元） |
| --- | --- |
| Automobile 汽车[soft check <3000, >500,000] | (HA065_1[1]) |
| Electric Bicycle 电动自行车 | (HA065_1[2]) |
| Motorcycle 摩托车 | (HA065_1[3]) |
| Refrigerator 电冰箱、冰柜 | (HA065_1[4]) |
| Washing machine 洗衣机 | (HA065_1[5]) |
| TV 电视机 | (HA065_1[6]) |
| Computer 家用电脑 | (HA065_1[7]) |
| Stereo system 组合音响 | (HA065_1[8]) |
| Video camera 摄像机 | (HA065_1[9]) |
| Camera 照相机 | (HA065_1[10]) |
| Air conditioner 空调 | (HA065_1[11]) |
| Mobile phone 手机 | (HA065_1[12]) |
| Furniture 值钱家具 | (HA065_1[13]) |
| Music instrument 高档乐器 | (HA065_1[14]) |
| Valuable decorations, ornaments, vases  昂贵的装饰、物品、花瓶 | (HA065_1[15]) |
| Treasures and precious metal (such as gold) you own? 您拥有的珠宝和贵重金属（如黄金等） | (HA065_1[16]) |
| Antiques, valuable paintings and calligraphic work, and other artistic work you own? 您拥有的古董、字画及其他艺术品 | (HA065_1[17]) |
| None 没有以上物品 |  |

F1：（1）“值钱家具”比如红木家具等三、四级家具。

（2）“"""" "

1保障性住房：549）：帮助在精神、情绪、体能或智能上有障碍的000000000000000000000000000000000000000000000000000000000000000000000000000000000000000000000000高档乐器”如钢琴、提琴、萨克斯等。

（3）“昂贵的装饰”物品、花瓶：进口地板、大理石装饰、昂贵的屏风等（非古董）。

**HA066.** Do members of your household own the following fixed capital assets? How much are the assets worth? (check all that apply) [ask same questions as in GA063] 您家有下列固定资产吗？现在值多少钱？（可多选）[问与GA063同样的问题]

1. Tractor, current value_______Yuan拖拉机，现在值______(HA066_1)元 [soft check <1000, >30,000]
2. Thresher, current value_______Yuan脱粒机(包括打稻机) ，现在值______(HA066_2)元[soft check <100 or >10,000]
3. Tractor tools, current value_______Yuan机引农具，现在值______(HA066_3 )元 [soft check <100 or >10,000]
4. Water pump, current value_______Yuan抽水机 (包括水泵)，现在值_____(HA066_4)元[soft check <100 or >10,000]
5. Processing equipment, current value_______Yuan加工机械，现在值_____(HA066_5 )元[soft check <100 or >10,000]
6. None 没有以上固定资产(HA066_6 )

F1：（1）“机引农具”例如机引犁、机引耙、机引播种机、旋耕机等。农产品加工机械：包括对收获后的农产品或采集的禽、畜产品进行初步加工，以及某些以农产品为原料进行深度加工的机械设备。

（2）“固定资产”是指企业使用期限超过1年的房屋、建筑物、机器、机械、运输工具以及其他与生产、经营有关的设备、器具、工具等。不属于生产经营主要设备的物品，单位价值在2000元以上，并且使用年限超过2年的，也应当作为固定资产。

**HA067.** What is the current value of other fixed capital assets used in household production or self-employed activities? 用于家庭生产、个体经营或开办私营企业的其他固定资产现在值多少钱？______ Yuan 元

F1：“固定资产”是指企业使用期限超过1年的房屋、建筑物、机器、机械、运输工具以及其他与生产、经营有关的设备、器具、工具等。不属于生产经营主要设备的物品，单位价值在2000元以上，并且使用年限超过2年的，也应当作为固定资产。

[IWER: Be sure to ask about fixed capital assets used in all self-employment activities, do not count assets already reported above. 访员注意：务必要询问用于所有家庭生产、个体经营或开办私营企业活动的固定资产，不包括前面问过了的固定资产。]

**HA068.** Does your household have any other durable or fixed assets worth 500 yuan or more? 您家有其他500元及以上的其他耐用品或固定资产吗？

1. Yes 有 How much are the assets worth? 现在值多少？___(HA068_1)Yuan元 [hard check>500] [soft check>50,000]
2. No 没有

F1：（1）“固定资产”是指企业使用期限超过1年的房屋、建筑物、机器、机械、运输工具以及其他与生产、经营有关的设备、器具、工具等。不属于生产经营主要设备的物品，单位价值在2000元以上，并且使用年限超过2年的，也应当作为固定资产。

（2）“耐用品”是指使用时间较长，至少在1年以上的物品，如电冰箱、汽车、电视机、机械设备等。

**HA069.** Have you lent to other families or individuals and not been repaid by them? 现在还有没有人或单位欠您家钱没还的？

1. Yes 有
2. No 没有 Skip to HA071 请跳至HA071

**HA070.** What is the total amount of the loans? 他们一共还欠您家多少钱？___________________ Yuan 元 [soft check>500,000]

**HA071.** How much interest income from what you lent to others in past year? 过去一年您家借出去的钱一共得到多少利息？ __________ Yuan  元 [soft check >50000]

**HA072.** What is the total amount of loans that you are still owing to other families, individuals, or your work unit? (not including mortgage loans) 您家还欠其他家庭、个人或单位的钱总数是多少？（不包括抵押贷款）__________________ Yuan 元 [soft check >500,000]

[IWER: Skip to HA074 if R answered HA072 If not, ask unfolding brackets HA073. 访员注意：如果受访者回答了HA072，跳至HA074，否则，提问分级展开问题HA073]

**HA073.** [IWER: If R is unwilling to answer or does not remember, ask unfolding bracket questions here. 访员注意：如果受访者不愿回答或者忘记了，在此处分级展开提问]5,000/10,000 /50,000 /100,000 /200,000 /500,000 yuan 元

**[Intro: Next we will ask your household members, other than the main respondent and spouse, some financial questions. 下面，我们将问除主要受访者及其配偶外的家户成员一些财产问题。**

[IWER: The names of other household members not including respondent and spouse are preloaded from the cover screen information. For each member, answer the following questions:] [访员注意：其他家户成员不包括主要受访者及其配偶，其他家户成员的名单要从过滤问卷获得。对于每一个其他家户成员，分别问下面的问题：]

**HA074.** What is the value of all financial assets of [preload household member name] (includes cash, savings, stocks, funds)? [preload household member name]拥有多少金融资产（包括现金、存款、股票、基金）？____Yuan 元 [soft check >1,000,000]

[PROCEDURE: If HA074_1>=1000000,ask HA074_check][程序：如果HA074_1大于等于100万元，询问HA074_check]

**HA074_check.** Are you sure the value of all financial assets of [preload household member name] (includes cash, savings, stocks, funds) is [preload HA074_1] Yuan? 您确定[加载家户名字]拥有[加载HA074_1的答案]元金融资产（包括现金、存款、股票、基金）吗？

(1)Yes 是

(2)No 否 go back HA074 返回到HA074

**HA075.** What is the value of all outstanding (unpaid) loans from banks or financial institutions (not including mortgages) of [name]? [name]还欠银行或其他金融机构的大笔贷款（不包括住房抵押贷款）有多少？__________ Yuan 元

[soft check >500,000]

**HA076.** How often did the respondent receive assistance in answering section Household assets? 受访者填写该部分问卷时是否求助？

[IWER: If it is answered by a proxy, please record the respondent’s reaction. 访员注意：如果是协助回答，请记录受访者的反应。]

1. Never 从未
2. A few times 偶尔几次
3. Most or all of the time 大多数时间

## HB INDIVIDUAL ASSETS 个人资产

[IWER: Please conduct sections HB and HC when the main respondent and his/her spouse are at home. Don’t allow a proxy to complete the entire sections. 访员注意：当主要受访者和其配偶在场时，才提问HB和HC 部分问卷。这部分不允许请人完全代填。]

**PART 1 Housing Reform 第一部分：房改**

**The following questions pertain to the housing reform. 下面是关于房改的问题。**

**HB001.** Have you ever purchase d a housing unit from your work unit? 您从单位买过房子吗？

- 1. Yes 是
  2. No 否 → Skip to HB018 请跳至HB018

**HB002.** Did you live in the housing unit before you purchased it from your work unit? 在从单位购买之前您就住在单位这房子里吗？

- 1. Yes 是
  2. No 否

**HB003.** Did you obtain the deed of the housing unit that you purchased from your work unit? 从单位买的这处房子，您有产权证吗？

- 1. Yes 是
  2. No 否

F1：“产权证”包括《房屋所有权证》和《土地使用权证》，但有些地方也可能是由房屋管理部门和土地管理部门统一开据的《房地产权证书》。

**HB004.** Is this your current residence? 您现在还住在单位这房子里吗？

1. Yes 是
2. No 否

**HB005 .** Do you still own the house that you purchased from your work unit? 您从单位买的房子，现在还是您的吗？

- 1. Yes 是 Skip to HC001 请跳至HC001
  2. No 否

**HB006.** When did you purchase the housing unit from your work unit? 您哪年从单位买的房子？

_____ 1949..2011Year 年

[IWER: Mark the year using four digits. 访员注意:用四位数字表示年份。]

**HB007 .** At what price did you purchase the housing unit? 当时您从单位买房的价格是？

Total price总价格____________(HB007_1)10000Yuan 万元[soft check: <0.1, >500] or unit price或单位价格________(HB007_2 )1000Yuan/m^2^ 千元/平方米[soft check: <0.1, >25]

[IWER: Skip to HB009 if R answered HB007. If not, ask HB008 如果受访者回答了HB007，跳至HB009 ，否则，HB008]

[PROCEDURE: If HB007_1>=100,ask HB007_check][程序：如果HB007_1大于等于100，询问HB007_check]

**HB007_check.** Are you sure you had paid [preload HB007_1] 10000Yuan for the housing unit? 您确定当时您从单位买房的价格是[加载HB007_1的答案]万元吗？

(1)Yes 是

(2)No 否 go back HB007 返回到HB007

**HB008.** Which of the following is close to the amount you spent on purchasing the house form your work unit then? 您当时买房的总开支大概在以下哪个档次内？

(1) Less than 5000 少于五千

(2) 5,000 to 10,000 五千到一万

(3) 10,000 to 20,000 一万到两万

(4) 20,000 to 50,000 两万到五万

(5) 50,000 to 100,000 五万到十万

(6) More than 100,000 十万以上

**HB009.** Was it purchased at market price, subsidized by working unit, or as purchased as economical housing? 这房子是以市场价买的、单位优惠价格买的、或者是经济适用房？

- 1. Market price 以市场价买 → Skip toHB011 请跳至 HB011
  2. Subsidized by working unit单位优惠价格买的
  3. Economic housing 经济适用房
  4. Other 其他

F1：“经济适用房”指已经列入国家计划，由城市政府组织房地产开发企业或者集资建房单位建造，以微利价向城镇中低收入家庭出售的住房，具有社会保障性质的商品住宅。具有经济性和适用性的特点。

**HB010.** What would you have had to pay if you had paid a market-set price for the same housing? 同样房子当时的市场价格是多少？

Total price 总价格__________(HB010_1)10000Yuan 万元[soft check <0.1, >500] or unit price或单位价格_________(HB010_2)1000Yuan/m^2^ 千元/平方米[soft check <0.1, >25]

[PROCEDURE: If HB010_1>=100,ask HB010_check][程序：如果HB010_1大于等于100，询问HB010_check]

**HB010_check.** Are you sure the market-set price of the same housing was [preload HB010_1] 10000Yuan at that time? 同样房子在当时的市场价确定是[加载HB010_1的答案]万元吗？

(1)Yes 是

(2)No 否 go back HB010 返回到HB010

**HB011.** What was the construction area of the housing unit? 房子的建筑面积是？_________ m^2^  平方米 [soft check <10, >500]

**HB012.** When did you sell or give away the housing unit? 何时售出或赠与他人？_______ 1949..2011Year 年

[IWER: Mark the year using four digits. 访员注意: 用四位数字表示年份。]

**HB013.** At what price did you sell it （0 if gave away）? 以什么样的价格售出(如果免费赠予，则填0)？

Total price 总价格_________ (HB013_1)10000Yuan 万元[soft check <0.1, >500] or unit price 或单位价格__________(HB013_2)1000Yuan /m^2^ 千元/平方米 [soft check <0.1, >25]

[PROCEDURE: If HB013_1>=100,ask HB013_check][程序：如果HB013_1大于等于100，询问HB013_check]

**HB013_check.** Are you sure you sold it at a price of [preload HB013_1] 10000Yuan? 您确定您以[加载HB013_1的答案]万元将其售出吗？

(1)Yes 是

(2)No 否 go back HB013 返回到HB013

[IWER: Skip to HB015 if R answered HB013. If not, ask HB014. 访员注意：如果受访者回答了HB013 ，跳至HB015，否则，提问HB014 题]

**HB014.** Which of the following is close to the total price you sold? 您当时售房的总收入大概在以下哪个档次内？

(1) Less than 20,000 两万以下

(2) 20,000 to 50,000 两万到五万

(3) 50,000 to 100,000 五万到十万

(4) 100,000 to 200,000 十万到二十万

(5) 200,000 to 500,000 二十万到五十万

(6) More than 500,000 五十万以上

**HB015.** Who did you sell or give away the housing unit to? 你把房子卖或赠给谁了？

1. Back to work unit还给单位
2. Commercial market市场上卖了
3. Child, which one? 给孩子，哪一个_____(HB015_1 )preload[提取孩子名单]
4. Parents, of who（respondent or spouse）? 给父母，谁的（受访者还是其配偶的）？______ (HB015_2 )
5. Other relative, specify其他亲戚，注明______(HB015_3 )
6. Other其他人

**HB016.** Did you sell at the market price or at a price below the market price? 这房子是以市场价还是低于市场价卖出的？

1. Market price 市场价 Skip to HC001 跳到 HC001
2. Below market price低于市场价

**HB017.** What was the market value of the housing unit when you sold it? 卖这房子时的市场价是多少？

Total price 总价格__________(HB017_1 )10000Yuan 万元[soft check <0.1, >500] or unit price或单位价格_________(HB017_2 )1000Yuan/m^2^ 千元/平方米 [soft check <0.1, >25]

[PROCEDURE: If HB017_1>=100,ask HB017_check][程序：如果HB017_1大于等于100，询问HB017_check]

**HB017_check.** Are you sure the market value of the housing unit was [preload HB017_1] 10000Yuan when you sold it? 您卖这个房子的时候市场价确定是[加载HB017_1的答案]万元吗？

(1)Yes 是

(2)No 否 go back HB017 返回到HB017

**Skip to HC001 请跳至HC001**

**HB018.** Why didn’t you purchase housing unit from your work unit? 您为什么没从单位买房？

- 1. I have no work unit 没有工作单位
  2. Not eligible to purchase 没有资格买
  3. Eligible but no housing unit was made available 有资格买，但没房
  4. Eligible but could not afford有资格买，但没钱买
  5. Eligible but decided not to purchase 有资格买，但不想买
  6. Other 其他

**PART 2 Financial Assets 第二部分：金融资产**

**The following questions pertain to your financial asset. 下面是关于金融资产的问题。**

[IWER reminder: make sure others are not present, IWER read following instructions: The following questions pertain to your financial asset. The answers to these questions will be kept strictly confidential and will be used for research purposes only. 访员注意：请确保没有其他人在场。请调查员念“下面是关于您的金融资产问题。我们将会对您的回答严格保密，并且仅用于学术研究。”]

**HC001.** How much cash is held by you and your spouse at home? 您和您爱人现在在家里有多少现金（随身携带以及放在家里的）？__________ Yuan 元 [soft check >50,000]

[IWER: Skip to HC003 if R answered HC001. If not, ask unfolding brackets. 访员注意：如果受访者回答了HC001 ，跳至HC003，否则，提问分级展开问题]

**HC002.** [IWER: If R is unwilling to answer or does not remember, ask unfolding bracket questions here. 访员注意：如果受访者不愿回答或者忘记了，在此处分级展开提问]500 /1,000/2,000/5,000/10,000 yuan 元

If married ( = 1 or 2), ask: 如果已婚(BE001 = 1 or 2)，提问：

**HC003.** Of which, how much do you own (if jointly owned with spouse then count 50%)? 其中，有多少是您名下的（如果和爱人共有，那么算50%）？

________(HC003_1)yuan 元[hard check <GM019] [soft check > 50,000] or或者 _______(HC003_2) % [range 0-100]

[CAPI: prompt for HC004 -HC019 IWER: for deposit, bonds，stocks, and funds, only include assets legally in his/her name.对HC004 -HC013提示：访员注意：对于存款、政府债券、股票和基金，只计算法定意义上在受访者名下的财产]

**HC004.** Are you currently holding any deposits in financial institutions in your name? 您目前在金融机构有（记在您名下的）存款吗？

1. Yes 有
2. No 没有 Skip to HC007 跳到HC007

**HC005.** What is the total amount of deposits you are currently holding in financial institutions（eg:bank）? 您现在在金融机构(如银行等)存了多少钱？_______________ Yuan元 [soft check >500,000]

[IWER: Skip to HC007 if R answered HC005 . If not, ask unfolding brackets. 访员注意：如果受访者回答了HC005，跳至HC007，否则，提问分级展开问题。]

**HC006.** [IWER: If R is unwilling to answer or does not remember, ask unfolding bracket questions here. 访员注意：如果受访者不愿回答或者忘记了，在此处分级展开提问] 2,000/5,000/10,000/50,000/100,000/200,000/500,000 yuan 元

**HC007.**  Do you have any government bonds (e.g.Treasury bills) in your name? 您有（记在您名下的）政府债券（如国库券）吗？

1. Yes 有
2. No 没有 -〉Skip to HC010 　请跳至HC010

**HC008.** What is the total face value of government bonds that you are currently holding? 您现有的所有政府债券(如国库券等)总共面值多少? __________________ Yuan 元 [soft check >50,000]

[IWER: Skip to HC010 if R answered HC008. If not, ask unfolding brackets HC009. 访员注意：如果受访者回答了HC008，跳至HC010，否则，提问分级展开问题HC009]

**HC009.** [IWER: If R is unwilling to answer or does not remember, ask unfolding bracket questions here. 访员注意：如果受访者不愿回答或者忘记了，在此处分级展开提问] 10,000 /50,000/100,000/200,000/500,000 yuan 元

**HC010.** Have you held any stocks in the past year in your name, excluding the equity or stock of your work unit? 不包括您在单位持有的股份，过去一年，您持有过（记在您名下的）股票吗？

1. Yes 有
2. No 没有 -〉Skip to HC015 　请跳至HC015

**HC011.** During the past year, overall did you earn money from stock dividends and increases in the value of your stocks or did you lose money? 过去一年，总的来说您在股票上赚钱还是赔钱了？

1. Earned 赚钱
2. Lost 赔钱-〉Skip to HC012_2 　请跳至HC012_2
3. Unchanged 不赔不赚 -〉Skip to HC013 　请跳至HC013

**HC012.** How much did you earn? 赚了多少？__Yuan 元 [soft check >200,000]

**HC012_2.** How much did you lose? 赔了多少？__Yuan 元 [soft check >200,000

**HC013.** What is the present market value of all the stocks you are currently holding? 您现在持有的股票当前价值多少？_________________ Yuan 元 [soft check >200,000]

[IWER: Skip to HC015 if R answered HC013. If not, ask unfolding brackets. 访员注意：如果受访者回答了HC013，跳至HC015，否则，提问分级展开问题。]

**HC014.** [IWER: If R is unwilling to answer or does not remember, ask unfolding bracket questions here. 访员注意：如果受访者不愿回答或者忘记了，在此处分级展开提问] 10,000 /50,000/100,000/200,000/500,000 yuan 元

**HC015.** Have you held any funds in your name in the past year? 过去一年，您持有过（记在您名下的）基金吗？

1. Yes 有
2. No 没有 -〉Go to PROGRAM before HC020 请跳至HC020 前面的程序控制

**HC016.** During the past year, overall did you earn money from holding funds or did you lose money? 过去一年，总的来说您在基金上赚钱还是赔钱了？

1. Earned 赚钱
2. Lost 赔钱
3. Unchanged 不赔不赚 -〉Skip to HC018 　请跳至HC018

**HC017.** How much did you earn or lose? 赚/赔了多少？____ Yuan 元 [soft check >200,000]

**HC018.** What is the present market value of all the mutual funds you are currently holding? 您现在持有的基金当前价值多少？___ _______________ Yuan 元 [soft check >200,000]

[IWER: Skip to HC020 if R answered HC018. If not, ask unfolding brackets. 访员注意：如果受访者回答了HC018，跳至HC020，否则，提问分级展开问题。]

**HC019.** [IWER: If R is unwilling to answer or does not remember, ask unfolding bracket questions here. 访员注意：如果受访者不愿回答或者忘记了，在此处分级展开提问]10,000 /50,000 /100,000 /200,000 /500,000 yuan 元]

[PROGRAM: If HC004 =1 or HC007 =1 or HC010 =1 or HC015 =1, go to HC020; if HC004 =2 and HC007 =2 and HC010 =2 and HC015 =2, then go to HC021. 程序：如果HC004 =1 or HC007 =1 or HC010 =1 or HC015 =1，询问HC020；如果HC004，HC007， HC010， HC015 答案都为2，那么请跳至HC021]

**HC020.** What percentage of the deposits, bonds, stocks, and funds held in your name is fully controlled by you and not your spouse? (%) 您名下的存款、政府债券、股票和基金有百分之多少是完全由你支配的？__ _____0..100％ [hard check >=0, <=100]

**HC021.** Do you have any other deposits, bonds, stocks, or funds that belong to you but which are held in a person’s name other than you or your spouse?您是否还有其他存款、政府债券、股票和基金没有记在您或您爱人的名下？

(1) Yes 是

(2) No 否 Skip to HC023 请跳至HC023

**HC022.** What is the value of such assets? 这些资产的总价值是多少？ ____yuan 元[soft check >200,000]

**HC023.** Other than income you have already told me about, did you receive any other income from other investments in past year? 除了上述提到的收入，过去一年您还有其他投资收入吗？

- 1. Yes 是
  2. No 否 → Skip to HC027 请跳至HC027

**HC024.** How much did you receive altogether from other investments in the past year? 您过去一年从其他投资中总共获得多少钱？ ________ Yuan 元[soft check >200,000]

[IWER: Skip to HC027 .if R answered HC024. If not, ask unfolding brackets HC025. 访员注意：如果受访者回答了HC024，跳至HC027，否则，提问分级展开问题HC025]

**HC025.** [IWER: If R is unwilling to answer or does not remember, ask unfolding bracket questions here. 访员注意：如果受访者不愿回答或者忘记了，在此处分级展开提问]1,000 /5,000/10,000/20,000/50,000 yuan 元]

**HC026.** What percentage of the other investments is owned jointly with your spouse? 其他投资，有百分之多少是与您爱人共有的？________0..100% [hard check <=0, >=100]

**HC027.** Do you have public housing funding? 您有住房公积金吗？

1. Yes 有
2. No 没有 Skip to HC030 请跳至HC030

F1：“住房公积金”指单位及其在职职工缴存的长期住房储金，是住房分配货币化、社会化和法制化的主要形式。

**HC028.** What is the total amount of money in your public housing fund? 您现在住房公积金账户上一共有多少钱？________________ Yuan 元 [soft check >100,000]

F1：“住房公积金”指单位及其在职职工缴存的长期住房储金，是住房分配货币化、社会化和法制化的主要形式。

[IWER: Skip to HC030 .if R answered HC028. If not, ask unfolding brackets HC029. 访员注意：如果受访者回答了HC028，跳至HC030 ，否则，提问分级展开问题HC029]

**HC029.** [IWER: If R is unwilling to answer or does not remember, ask unfolding bracket questions here. 访员注意：如果受访者不愿回答或者忘记了，在此处分级展开提问]5,000/10,000 /50,000/100,000/200,000/500,000 yuan 元

**HC030.** Do you have Jizikuan that your work unit or other work units have collected from you and are still holding (Jizikuan is fund individuals provided to the work unit for the purpose of investment, building apartments, etc.)? 您有没有交给自己单位或其他单位的集资款还未返还？

1. Yes 有
2. No 没有 Skip to HC033 请跳至HC033

F1：“集资”是从企业内部获得资金,让自己下属拿出钱来入股就是集资，这些入股的资金就是集资款。包括交给受访者单位或其他单位的所有集资款。

**HC031.** What is the amount of your jizikuan? 您的集资款总金额是多少？_________________ Yuan 元 [soft check >200,000]

F1：“集资”是从企业内部获得资金,让自己下属拿出钱来入股就是集资，这些入股的资金就是集资款。包括交给受访者单位或其他单位的所有集资款。

[IWER: Skip to HC033 .if R answered HC031. If not, ask unfolding brackets HC032. 访员注意：如果受访者回答了HC031，跳至HC033，否则，提问分级展开问题HC032]

**HC032.** [IWER: If R is unwilling to answer or does not remember, ask unfolding bracket questions here. 访员注意：如果受访者不愿回答或者忘记了，在此处分级展开提问]5,000/10,000 /50,000 /100,000 /200,000 /500,000 yuan 元

**HC033.** Do you have any unpaid salary that your work unit still owes you? 目前，有单位拖欠您的工资吗？

1. Yes 有
2. No 没有 Skip to HC036 请跳至HC036

**HC034.** What is the amount of your unpaid salary? 一共拖欠您多少工资？______________ Yuan 元

**[soft check >100,000]**

[IWER: Skip to HC036.if R answered HC034. If not, ask unfolding brackets HC035. 访员注意：如果受访者回答了HC034，跳至HC036，否则，提问分级展开问题HC035]

**HC035.** [IWER: If R is unwilling to answer or does not remember, ask unfolding bracket questions here. 访员注意：如果受访者不愿回答或者忘记了，在此处分级展开提问]5,000/10,000 /50,000 /100,000 /200,000 yuan 元

**HC036.** Have you participated in any rotating savings and credit association during the past year? 您在过去一年里有没有参加过标会?

1. Yes 有
2. No 没有 🡪 HD001 请跳至🡪 HD001

F1：“标会”民间标会又称互助会或跟会、搭会，具有赚取利息及筹措资金的双重功能,是民间重要的理财工具。

**HC037.** What is the total amount of funds that you are still obligated to pay to the rotating savings and credit association？您还需要支付给标会多少钱？ ____ yuan 元

**PART 3 DEBTS 第三部分：债务**

**The following questions pertain to your debt. 下面是关于您债务的问题。**

**HD001.** What is the total amount of loan that you haven’t repaid yet (not including loans for house)? 您尚未还清的贷款总额(不包括买房、建房、装修的贷款)是多少？ ________________ Yuan 元 [soft check>500,000]

[IWER: Skip to HD003 if R answered HD001. If not, ask unfolding brackets HD002. 访员注意：如果受访者回答了HD001 ，跳至HD003 ，否则，提问分级展开问题HD002]

**HD002.**  [IWER: If R is unwilling to answer or does not remember, ask unfolding bracket questions here. 访员注意：如果受访者不愿回答或者忘记了，在此处分级展开提问]5,000/10,000 /50,000 /100,000 /200,000 /500,000 yuan 元

**HD003.** What is the amount of your credit card balance? 现在您的信用卡所欠金额是多少？________________ Yuan 元 [soft check >50,000]

[IWER: Skip toHD005 if R answered HD003, If not, ask unfolding brackets HD004. 访员注意：如果受访者回答了HD003 ，跳至HD005 ，否则，提问分级展开问题HD004]

**HD004 .** [IWER: If R is unwilling to answer or does not remember, ask unfolding bracket questions here. 访员注意：如果受访者不愿回答或者忘记了，在此处分级展开提问]5,000/10,000 /50,000 /100,000 /200,000 /500,000 yuan 元

**HD005.** Have you ever inherited anything？您是否继承过遗产？

- 1. Yes 是
  2. No 否 → Skip to HD012 请跳至 HD012

**HD006.** How much in total have you inherited ？您继承的遗产总共值多少钱？ ________ Yuan 元 （按继承时的价值来算）[soft check >200,000]

[CAPI: Skip to HD008 if R answered HD006 If not, ask unfolding brackets HD007. 如果受访者回答了HD006 ，跳至HD008 ，否则，提问分级展开问题HD007 。]

**HD007.** [IWER: If R is unwilling to answer or does not remember, ask unfolding bracket questions here. 访员注意：如果受访者不愿回答或者忘记了，在此处分级展开提问]5,000/10,000 /50,000 /100,000 /200,000 /500,000 yuan 元

**HD008.** From whom you inherited？(Choose all that apply) 您从谁那里继承了遗产？（可多选）

(1)Parents 父母

(2)Parents-in-law 岳父母

(3)Children 子女

(4)Relatives 亲戚

(5)Others 其它

**HD009.** When did the largest inheritance occur？您继承遗产最多的一次发生在哪一年？_______ 1900..2011 Year 年

[IWER: Mark the year using four digits. 访员注意: 用四位数字表示年份。]

**HD010.** What was the value of that inheritance? 当时这笔遗产值多少钱？_______________ Yuan 元

[CAPI: Skip to HD012 if R answered HD010. If not, ask unfolding brackets HD011..如果受访者回答了HD010，跳至HD012，否则，提问分级展开问题HD011 ] [soft check >200,000]

**HD011.** [IWER: If R is unwilling to answer or does not remember, ask unfolding bracket questions here. 访员注意：如果受访者不愿回答或者忘记了，在此处分级展开提问]5,000/10,000 /50,000 /100,000 /200,000 /500,000 yuan 元

**HD012.**  How often did the respondent receive assistance in answering section G ASSETS? 受访者填写该部分问卷时是否求助？

[IWER: If it is answered by a proxy, please record the respondent’s reaction.访员注意：如果是协助回答，请记录受访者的反应。]

- 1. Never 从未
  2. A few times 偶尔几次
  3. Most or all of the time 大多数时间

## HOUSING CHARACTERISTICS 住房情况

**[PROCEDURE: For main respondent only I001 --- I026 ] [程序：仅主要受访者回答I001 --- I026 ]**

**I001.** What is the construction area of your residence? 您现在住房的建筑面积是多大？______m^2^ 平方米

[soft check: <10 or >500]

**I002.** [What is the total housing land area (*zhaijidi,* including both the house construction area and the yard area) 宅基地的面积有多大?（宅基地，包括房屋的建筑面积和院落的面积） __m^2^平方米 [soft check <10, >1000]

**I003.** Is your residence used for business as well? 这个住房兼作生产经营用房吗？

(1) 是

(2) 否

**I004.** What type of structure is this building? Is it concrete and steel, mixed structure, bricks and wood, wood, bamboo, grass or other? 本建筑是什么结构？

1. Concrete and steel 钢筋混凝土结构
2. Bricks and wood 砖木结构
3. Mixed structure 混合结构
4. Wood, bamboo, grass 木、竹、草结构
5. Woolen felt 毛毡
6. Sheet iron 简易铁皮房
7. Cave dwelling 窑洞
8. Tent 帐篷
9. Adobe 土坯房
10. Other 其它结构

**I005.** When was this house built? 这个房子是什么时候建成的？

____year 年

If R is unclear about year, choose: 如果不清楚具体年份，选：

（1）0-5 years 0-5年

（2）5-10 years 5-10年

（3）10-20 years 10-20年

（4）20-30 years 20-30年

（5）30-40 years 30-40年

（6）more than 40 years 40年以上

**I006.** Is the building one story or multi-level building, how many stories? 住房所在建筑是平房还是楼房，多少层的？

1. One-story building 平房 skip to I007
2. Multi-story building 楼房 skip to I008

**I007.** Is the story independent or compound? 是独立的平房，还是大杂院，

(1) Independent story 独立的平房

(2) Compound 大杂院

**I008.** Which story is this building on? 该住房在第几层？

**[PROCEDURE: If I008 >1, ask I009.] [程序：如果I008 >1，询问I009 .]**

**I009.** Does it has elevator？有电梯吗？

（1）Yes 是

（2）No 否

**I010.** Are there any handicapped facilities (e.g., non-stair ramp)? 住宅是否有无障碍通道？（例如：没有台阶的斜坡等）

(1) Yes 是

(2) No 否

**[PROCEDURE: If I010 =2, ask I011.] [程序：如果I010 =2，询问I011 ]**

**I011.** How many steps had to be climbed to get to the main entrance of the household's flat? 从外面回来要爬多少个阶梯才能到家门口？

[IWER: Do not count steps if an elevator is available. 访员注意：可以坐电梯而不用爬的阶梯忽略不计。]

(1) 1- 5 1-5个阶梯

(2) 6 to 15 6-15个

(3) 16 to 25 16-25个

(4) More than 25 25个以上

**I012.** How many bedrooms, living rooms, bathrooms, and kitchens are there in your residence? 您家现在的房屋有_________(I012_1 )bedrooms室________( I012_2 )living rooms厅_________( I012_3 )toilets卫生间（厕所）________(I012_4)kitchens厨房________( I012_5 )balcony阳台 [soft check: >20]

**[PROCEDURE: Ask I013 if no toilets in answer to I012.] [如果I012 答案中没有卫生间，则提问I013]**

**I013.** How far is the nearest toilet to your house? 离您家最近的厕所多远？___ meters 米 [soft check: >500]

**I014.** What is the type of toilet? 厕所是什么样的？是蹲坑式还是坐式？

1. Toilet without a seat 蹲坑式
2. Toilet with a seat 坐式

**I015.** Is the toilet flushable? 厕所能冲水吗？

1. 是
2. 否

**I016.** Does your residence have electricity? 是否有电？

- 1. Yes 是
  2. No 否

**I017.** Does your residence have running water? 是否有自来水？

- 1. Yes 是
  2. No 否

**I018.** Is there in-house shower or bath facility? What type? 住房内有无洗澡设施？是什么样的？

(1) Hot water provided 统一供热水

(2) Water heater installed by the household 家庭自装热水器

(3) No 无

**I019.** Does your residence have coal gas or natural gas supply? 是否有管道煤气或天然气？

- 1. Yes是
  2. No 否

**I020.** Does your residence have heating?　是否带供暖设施（不包括土暖气和可制暖的空调）？

- 1. Yes是 → Skip to I022 请跳至I022
  2. No 否

**I021.** What is the main heating energy source? 供暖所用的主要能源是什么？

1. Solar 太阳能
2. Coal 煤炭、蜂窝煤
3. Natural gas 管道天然气或煤气
4. Liquefied Petroleum Gas 液化石油气
5. Electric 电
6. Crop residue/Wood buring 秸秆、柴火
7. Other 其他

**I022.** What is the main source of cooking fuel? 做饭用的主要燃料是什么？

1. Coal 煤炭、蜂窝煤
2. Natural gas 管道天然气或煤气
3. Marsh gas 沼气
4. Liquefied Petroleum Gas 液化石油气
5. Electric 电
6. crop residue/Wood burning 秸秆、柴火
7. other 其他

**I023.** Does your residence have a telephone connection?　您家装电话了吗？

- 1. Yes 是
  2. No 否

**I024.** Does your residence have broad-band internet connection? 您家可以宽带上网吗？

- 1. Yes 是
  2. No 否

**I025. [Interviewer records it] [访员自己记录]** How clear and tity is in this household？这户人家的室内整洁度如何？

(1) Excellent 非常整洁

(2) Very clear 很整洁

(3) Clear 整洁

(4) Fair 一般

(5) Poor 不整洁

**I026. [Interviewer records it] [访员自己记录]**[How](http://www.iciba.com/how/) is the [temperature](http://www.iciba.com/temperature/) in this household？这户人家的室内温度如何？

(1) Very hot 很热

(2) Hot 比较热

(3) Bearable 还可以

(4) Cold 比较冷

(5)Very cold 很冷

## HOUSEHOLD CONTACTS 家户联系信息表

| **INTRO: We will inform you blood tests report. And in the future, we will visit this household again.Your continue participation is very important to our studay. In order to find you in the future survey, we have to get your and your friend、family member’s contact information. All the contact information will be kept strictly confidential.Thanks for your understanding and help.过些天会有国家疾控中心的人通知您参加血检，为了通知您时间及血检结果，我们希望记下您和您朋友或家人的电话和地址。请您放心，我们会对您留下的所有联系方式严格保密。**  **AIK1.**Could you please tell me your contact information? 请把您的联系方式告诉我。  Name [preload main respondent name] 姓名:[加载主要受访者的姓名]  Home 家庭电话**└─┴─┴─┴─┘** (AIK1_2_1)**-└─┴─┴─┴─┴─┴─┴─┴─┘** (AIK1_2_2)  **INTRO: I make a call to your home telephone to check whether it is right.我拨一下您家的电话，看我是不是写对了。**  [IWER:Call the number to check.If the number is right, then continue,if wrong, then go back and correct.访员注意：拨一下受访者留的电话号码，检查是否记录正确。如果不对，请返回修改。]  **AIK2.** IWER:Record whether the home telephone number is right.访员填写：记录电话号码是否正确  (1)right 正确  (2)wrong 错误  10000000000.0..19999999999.0**Cell phone number 手机: └─┴─┴─┴─┴─┴─┴─┴─┴─┴─┴─┘**  (AIK1_3)  **Intro: I make a call to your cell phone to check whether it is right.我拨一下您的手机，看我是不是写对了。**  [IWER:Call the number to check.If the number is right, then continue,if wrong, then go back and correct.访员注意：拨一下受访者留的电话号码，检查是否记录正确。如果不对，请返回修改。]  **AIK3.** WER:Record whether the cell phone number is right.访员填写：记录手机号码是否正确  (1)right 正确  (2)wrong 错误  **AIK4. Email address 电子邮箱:_______________________________________________**  **AIK5.** Could you give us your mailing address, so we can send you blood test result later？ 能否告诉我们您家的邮寄地址，这样我们能够将血检结果寄给您。  Mailing Address 邮寄地址: ______________________________________________________________________________________ Zip Code 邮政编码____________________ (AIK1_7) | |
| --- | --- |
| **INTRO: We will call on you two years later, could you give us the name and contact information of a friend/family member who can tell us where you are? 我们这个调查追踪调查，下一次全国调查是2013年。能否告诉我们一个您本村/社区的朋友或者亲戚的姓名及联系方式，这样我们可以在两年后联系到你。**  **AIK6.**Name 姓名:____________(AIK6_1)What is his relationship to you? 他是您的什么人？___________________________________________________________(AIK6_1_0)  Telephone number 电话号码: Home 家庭电话**└─┴─┴─┴─┘** (AIK6_2_1)**-└─┴─┴─┴─┴─┴─┴─┴─┘** (AIK6_2_2)  10000000000.0..19999999999.0Cellphone number 手机: **└─┴─┴─┴─┴─┴─┴─┴─┴─┴─┴─┘**(AIK6_3)  **Email address 电子邮箱:_______________________________________________**(AIK6_3_1)  Mailing Address 邮寄地址: _____________________________________________________________________________(AIK6_4)_ Zip Code 邮政编码____________________ (AIK6_7)  AIK7. Could you give us the name and contact information of a second friend/family member who can tell us where you are? 能否告诉我们您另一个不住在本村/社区的朋友或者亲戚的姓名及联系方式，这样我们可以通过他们联系到您。  Name 姓名:_______________________________(AIK7_1) What is his relationship with you? 他是您的什么人？________________________________________________(AIK7_0)  Telephone number 电话号码: Home 家庭电话**└─┴─┴─┴─┘** (AIK7_2_1)**-└─┴─┴─┴─┴─┴─┴─┴─┘** (AIK7_2_2)  10000000000.0..19999999999.0 Cellphone number 手机: **└─┴─┴─┴─┴─┴─┴─┴─┴─┴─┴─┘**(AIK7_3)  **Email address 电子邮箱:_______________________________________________**(AIK7_3_1)  Mailing Address 邮寄地址: ______________________________________________________________________________ (AIK7_4) Zip Code 邮政编码____________________ (AIK7_7) | |
| **AIK8a.** Did you move within last two years? 在过去的两年内，您搬过家吗？  1.Yes 搬过 2.No 没搬过 skip AIK8b 跳过 AIK8b  **AIK8b.** Did you move here because of demolition, stay here temporary or just move back because of demolition? 你是因为拆迁搬到目前住处，临时住这里，还是因为拆迁刚搬回来？   - - - 1. move here because of demolition 拆迁搬到目前住处       2. stay here temporary 临时住这里       3. move back because of demolition 拆迁刚搬回来       4. other 其他   **AIK8.** Do you intend to move within the next two years? 最近两年内，您是否会搬家或拆迁？  **AIK9.** If you move, where will you move? 如果搬家/拆迁，您会搬去哪儿？  Country 国家:Province 省:  City, district/county 市、区/县：  Street/Township 街道/乡镇：  Community/Village 小区/村:  **AIK10.** Could you please tell us a contact information for us to contact you in the future if you move, the phone number of your hometown is the best.您能否再告诉我们一个到时能联系到您的，比如您老家的电话号码以便我们以后回访？  Name联系人姓名：_____________ (AIK10_1)  what is his/her relationship with you与受访者的关系：_______________________ (AIK10_2)  phone number固定电话：_____________ (AIK10_3) | **1. Move 会搬 2. NOT MOVE 不会搬🡺next section 请跳至下一部分 8. DON’T KNOW 不知道**  🡻 **↓**  **Skip to the next section 请跳至下一部分**   1. **Not same 与现在住址不同**   🡻  1、___________________________________ 2、same 本国 **🡺next section 请跳至下一部分**  8、 DON’T KNOW 不知道  1、___________________________________ 2、same 本省 **🡺next section 请跳至下一部分**  8、 DON’T KNOW 不知道  1、___________________________________ 2、same 本市、区/县 **🡺next section 请跳至下一部分**  8、 DON’T KNOW 不知道  1、___________________________________ 2、same 本街道/乡镇 **🡺next section 请跳至下一部分**  8、 DON’T KNOW 不知道  1、___________________________________ 2、same 本小区/村 **🡺next section 请跳至下一部分** |

## J. INTERVIEWER OBSERVATION 访问员观察

**[IWER. This section is about your observations during the interview and should be filled out after each completed individual interview.] [访员注意：这部分是您在调查时所做的观察，访问结束后，请填写这部分内容.]**

**J001.** Were any third persons, except proxy respondents, present during (parts of) the interview with ^FL Respondent Name? (circle all that apply) 当您访问主要受访者时，除代理回答人外，是否还有其他人在场？（可多选）

(1) Nobody 没有其他人 Skip to J003 跳至J003

(2) Spouse or partner 受访者的配偶

(3) Parent or parents 受访者的父母亲

(4) Child or children 受访者的孩子

(5) Other relatives 其他亲戚

(6) Other persons present 还有其他的人在场

**J002.** Did these persons intervene during the interview? 调查时，这人/这些人有没有干预？

(1) Yes, often 有，经常干预

(2) Yes, occasionally 有，偶尔干预

(3) No 没有

**J003.** How would you describe the willingness of ^FLRespondentName to answer? 受访者回答问卷时配合程度如何？

(1) Very good 非常配合 Skip to J005 跳过J005

(2) Good 比较配合 Skip to J005 跳过J005

(3) Fair 一般 Skip to J005 跳过J005

(4) Bad 不太配合 Skip to J005 跳过J005

(5) Good in the beginning, got worse during the interview 刚开始配合，后来就不太配合

(6) Bad in the beginning, got better during the interview 刚开始不太配合，后来态度慢慢变好了 Skip OV005 跳过OV005

**J004.** Why did the respondent's willingness to answer get worse during the interview? (Choose all that apply)

受访者为什么后来不大愿意回答问卷了？（可多选）

(1) The respondent was losing interest 没有兴趣了

(2) The respondent was losing concentration or was getting tired 受访者注意力不能集中了，有些厌倦

(3) Other, please specify 其他，请注明_______

**J005.** Did ^FLRespondentName ask for clarification on any questions? 受访者回答问题时，需要您来解释吗？

(1) Never 根本不需要

(2) Almost never 基本上不需要

(3) Now and then 偶尔需要

(4) Often 经常需要

(5) Very often 频繁需要

(6) Always 一直需要

**J006.** Overall, did you feel that ^FLRespondentName understood the questions? 总的来说，您觉得受访者能听懂这些题目吗？

(1) Never 全部听不懂

(2) Almost never 几乎听不懂

(3) Now and then 偶尔能听懂

(4) Often 大部分能听懂

(5) Very often 绝大部分能听懂

(6) Always 全部能听懂

**J007.** Did the respondent need any help reading the showcards during the interview? 出示卡片时，受访者要您帮忙读卡片？

(1) Yes, due to sight problems 要，由于视力问题

(2) Yes, due to literacy problems 要，因为不识字

(3) No 不需要

# **APPENDIX 附表**

**Appendix 1: List of Chinese Zodiac signs 附表一: 属相列表**

| **1** | **Rat charm** | **鼠** |
| --- | --- | --- |
| **2** | **Ox patient** | **牛** |
| **3** | **Tiger sensitive** | **虎** |
| **4** | **Rabbit articulate** | **兔** |
| **5** | **Dragon healthy** | **龙** |
| **6** | **Snake deep** | **蛇** |
| **7** | **Horse popular** | **马** |
| **8** | **Goat elegant** | **羊** |
| **9** | **Monkey clever** | **猴** |
| **10** | **Rooster deep thinkers** | **鸡** |
| **11** | **Dog loyalty** | **狗** |
| **12** | **Pig chivalrous** | **猪** |

**Appendix 2: List of provinces 附表二：省份列表**

| **1** | **Anhui** | **安徽** |
| --- | --- | --- |
| **2** | **Beijing** | **北京** |
| **3** | **Chongqing** | **重庆** |
| **4** | **Fujian** | **福建** |
| **5** | **Gansu** | **甘肃** |
| **6** | **Guangdong** | **广东** |
| **7** | **Guangxi** | **广西** |
| **8** | **Guizhou** | **贵州** |
| **9** | **Hainan** | **海南** |
| **10** | **He’nan** | **河南** |
| **11** | **Hebei** | **河北** |
| **12** | **Heilongjiang** | **黑龙江** |
| **13** | **Hu’nan** | **湖南** |
| **14** | **Hubei** | **湖北** |
| **15** | **Inner mongolia** | **内蒙古** |
| **16** | **Jiangsu** | **江苏** |
| **17** | **Jiangxi** | **江西** |
| **18** | **Jilin** | **吉林** |
| **19** | **Liaoning** | **辽宁** |
| **20** | **Ningxia** | **宁夏** |
| **21** | **Qinghai** | **青海** |
| **22** | **Shandong** | **山东** |
| **23** | **Shanghai** | **上海** |
| **24** | **Shannxi** | **陕西** |
| **25** | **shanxi** | **山西** |
| **26** | **Sichuan** | **四川** |
| **27** | **Tianjin** | **天津** |
| **28** | **Tibet** | **西藏** |
| **29** | **Xinjiang** | **新疆** |
| **30** | **Yunnan** | **云南** |
| **31** | **Zhejiang** | **浙江** |
| **32** | **Hongkong** | **香港** |
| **33** | **Macao** | **澳门** |

**Appendix 3: List of Occupation 附表三：行业编码**

| **1** | **Agriculture, Forestry, herd, fishing** | **农、林、牧、渔业** |
| --- | --- | --- |
| **2** | **Mining** | **采矿业** |
| **3** | **Manufacture** | **制造业** |
| **4** | **Energy sector(including electricity, gas**  **and water)** | **电力、燃气及水的生产和供应业** |
| **5** | **Construction** | **建筑业** |
| **6** | **Transportation, warehousing industry**  **and postal industry** | **交通运输、仓储和邮政业** |
| **7** | **Information transmission , computer**  **service**  **and software industry** | **信息传输、计算机服务和软件业** |
| **8** | **Wholesale and Retail** | **批发和零售业** |
| **9** | **Lodging and catering sector** | **住宿和餐饮业** |
| **10** | **Finance** | **金融业** |
| **11** | **Real estate business** | **房地产业** |
| **12** | **Leasing industry and Business Service**  **Industry** | **租赁和商务服务业** |
| **13** | **Science research, technical service and geology prospecting industry** | **科学研究、技术服务和地质勘查业** |
| **14** | **Water conservancy , environments and**  **public facilities management** | **水利、环境和公共设施管理业** |
| **15** | **Personal Services and other service**  **industry** | **居民服务和其他服务业** |
| **16** | **Education** | **教育** |
| **17** | **Public healthcare, social security and public welfare** | **卫生、社会保障和社会福利业** |
| **18** | **Culture , sports and entertainment** | **文化、体育和娱乐业** |
| **19** | **Common administration and society**  **organize** | **公共管理与社会组织** |
| **20** | **International organization** | **国际组织** |

1. *****, Please refer to LOOP questionnaire **“**HMem_Roster.dta**”.** 所有标记*的题，请参见LOOP问卷“家户成员个人信息”。 [↑](#footnote-ref-2)
2. ******, Please refer to LOOP questionnaire **“**HHMem1.dta**”.** 所有标记**的题，请参见LOOP问卷“BA 家户登记表 家户成员信息”。 [↑](#footnote-ref-3)
3. *******, Please refer to LOOP questionnaire **“**Parents1.dta**”.** 所有标记***的题，请参见LOOP问卷“BA 家庭登记表 父母信息”。 [↑](#footnote-ref-4)
4. **********, Please refer to LOOP questionnaire **“**Parents2.dta**”.** 所有标记******的题，请参见LOOP问卷“BE 照顾父母 父母信息”。 [↑](#footnote-ref-5)
5. ************, Please refer to LOOP questionnaire **“**Dailycare.dta**”.** 所有标记********的题，请参见LOOP问卷“Bh 提供照料时间”。 [↑](#footnote-ref-6)
